# Identification of a Novel Pseudo‐Natural Product Type IV IDO1 Inhibitor Chemotype

**DOI:** 10.1002/anie.202209374

**Published:** 2022-08-29

**Authors:** Caitlin Davies, Lara Dötsch, Maria Gessica Ciulla, Elisabeth Hennes, Kei Yoshida, Raphael Gasper, Rebecca Scheel, Sonja Sievers, Carsten Strohmann, Kamal Kumar, Slava Ziegler, Herbert Waldmann

**Affiliations:** ^1^ Max Planck Institute of Molecular Physiology Department of Chemical Biology Otto-Hahn-Strasse 11 44227 Dortmund Germany; ^2^ Technical University of Dortmund Department of Chemical Biology Otto-Hahn-Strasse 6 44227 Dortmund Germany; ^3^ Technical University of Dortmund Department of Inorganic Chemistry Otto-Hahn-Strasse 6 44227 Dortmund Germany; ^4^ Compound Management and Screening Center (COMAS) Otto-Hahn-Strasse 11 44227 Dortmund Germany; ^5^ Current address: Institute for Stem-Cell Biology Regenerative Medicine and Innovative Therapies IRCCS Casa Sollievo della Sofferenza 71013 San Giovanni Rotondo Italy; ^6^ Center for Nanomedicine and Tissue Engineering (CNTE) ASST Grande Ospedale Metropolitano Niguarda 20162 Milan Italy; ^7^ Current address: AiCuris Anti-infective Cures AG Friedrich-Ebert-Str. 475 42117 Wuppertal Germany

**Keywords:** Cancer, Heme Proteins, Immunology, Natural Products, Structure-Activity Relationships

## Abstract

Natural product (NP)‐inspired design principles provide invaluable guidance for bioactive compound discovery. Pseudo‐natural products (PNPs) are de novo combinations of NP fragments to target biologically relevant chemical space not covered by NPs. We describe the design and synthesis of apoxidoles, a novel pseudo‐NP class, whereby indole‐ and tetrahydropyridine fragments are linked in monopodal connectivity not found in nature. Apoxidoles are efficiently accessible by an enantioselective [4+2] annulation reaction. Biological evaluation revealed that apoxidoles define a new potent type IV inhibitor chemotype of indoleamine 2,3‐dioxygenase 1 (IDO1), a heme‐containing enzyme considered a target for the treatment of neurodegeneration, autoimmunity and cancer. Apoxidoles target apo‐IDO1, prevent heme binding and induce unique amino acid positioning as revealed by crystal structure analysis. Novel type IV apo‐IDO1 inhibitors are in high demand, and apoxidoles may provide new opportunities for chemical biology and medicinal chemistry research.

## Graphical abstract

Pseudo‐natural products are de novo combinations of natural product (NP) fragments to target biologically relevant chemical space not covered by existing NPs. We describe the design and synthesis of the novel pseudo‐NP class termed apoxidoles. Biological investigation revealed potent reduction of cellular kynurenine levels by apoxidoles through selective targeting of apo‐indoleamine 2,3‐dioxygenase 1 (apo‐IDO1).

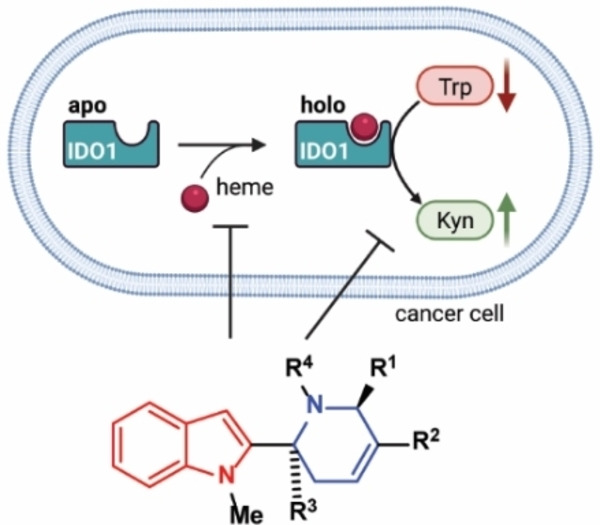

## Introduction

Natural products (NPs) are inherently diverse, frequently possess biological activity,^[1]^ and are valuable starting points for the design of novel, biologically relevant scaffolds. This relevance calls for new approaches in order to discover compound classes that can be considered NP‐inspired, but occupy unexplored areas of natural product‐like chemical space. The recently introduced pseudo‐natural product (PNP) approach^[2]^ combines natural product fragments de novo and through different arrangements to yield compound classes that structurally resemble NPs but are not found in nature.^[3]^ As a result, pseudo‐NPs inherit typical properties of NPs from their respective NP fragments,^[4]^ yet occupy biologically relevant chemical space not covered by existing NPs. In pseudo‐NP design and synthesis, variation of fragment ‐type, ‐combination and ‐connectivity may yield unique chemically and biologically diverse compound classes.^[5]^

**Figure 1:**
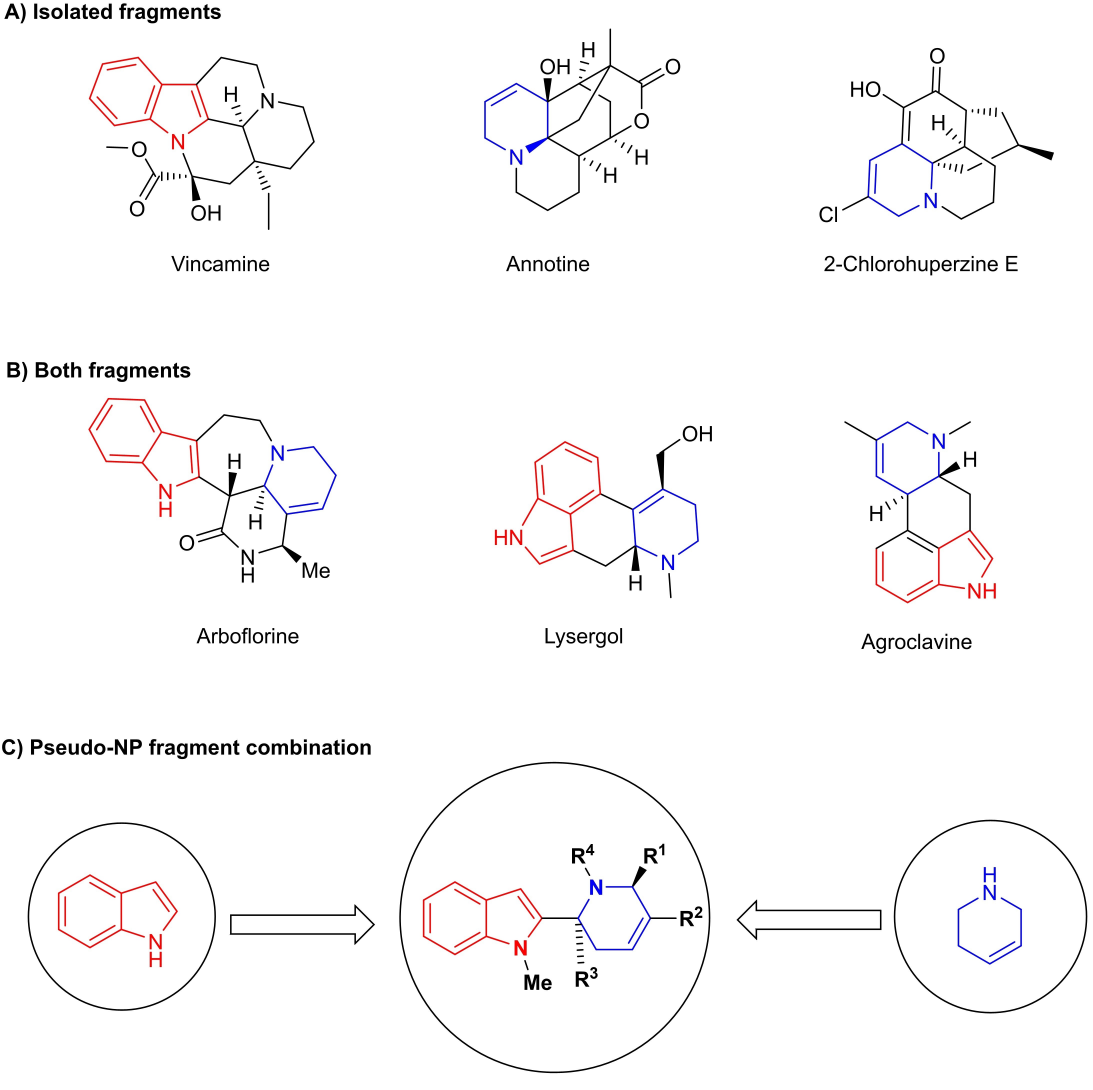
Design of the pseudo‐NP class based on the fragments derived from indole‐ (red) and THP (blue) natural products.

For the development of a new PNP class, we considered to combine the indole‐ and the tetrahydropyridine (THP) fragments. Both occur individually in numerous bioactive NPs^[6]^ such as vincamine and annotine (Figure 1A), and are also found together in a number of NPs with complex, fused ring systems e.g. lysergol and arboflorine (Figure 1B). However, analysis of the Dictionary of Natural Products‐ and Coconut databases^[7]^ indicated that a direct mono‐podal connection (i.e. direct connection, no intermediary ring) between indole‐ and THP‐fragments in NPs has not been found. Therefore, we aimed to combine these two fragments in a mono‐podal manner, that is biologically unexplored. Herein, we describe the synthesis of a novel pseudo‐NP class, combining indole and THP fragments (Figure 1C) via a complexity‐generating enantioselective [4+2] annulation reaction.^[8]^ Biological investigation revealed a new potent type IV indoleamine 2,3‐dioxygenase 1 (IDO1) inhibitor chemotype, that targets apo‐IDO1 and interferes with heme binding.^[9]^ Novel type IV apo‐IDO1 inhibitors are in high demand, since the prototypic type II holo‐IDO1 inhibitor epacadostat recently failed in clinical trials,^[10]^ while the type IV apo‐IDO1 inhibitor BMS‐986205 is still actively investigated in late stage.^[11]^ The novel inhibitor class, termed apoxidoles, has a distinct chemotype and induces unprecedented structural changes not observed for other type IV inhibitors, as revealed by a crystal structure analysis.^[12]^

## Results and Discussion

### Synthesis of Apoxidoles

For the synthesis of a new indole‐THP PNP class in which the two fragments would be connected via C2 of the indole and C6 of the THP, we turned to the seminal work of Kwon et al. who described a [4+2] annulation of *N*‐nosyl‐indole‐2‐aldimines with allenoates.^[8]^ An asymmetric version has previously been shown to yield moderate to good diastereo‐ and high enantioselectivity when catalysed with a binaphthyl‐based C_2_‐symmetric monophosphine.^[13]^

**Scheme 1:**
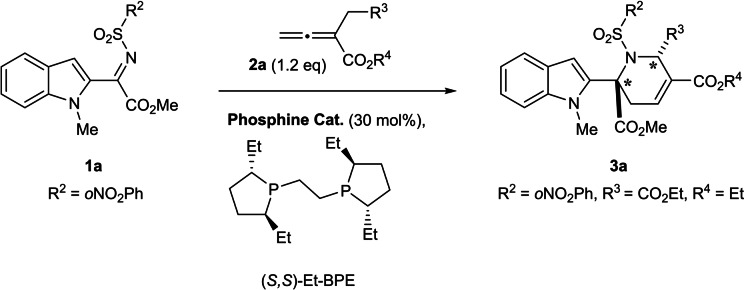
Asymmetric synthesis of the apoxidole pseudo‐NP class; optimised conditions: Allenoate (1.2 equiv), (*R*,*R*) or (*S*,*S*)‐Et‐BPE (30 mol %), THF [0.05 M], 0 °C, rt, 12 h.

In order to advance this chemistry and enrich the stereogenic content of the formed tetrahydropyridine, we employed N‐nosyl ketimine **1** **a** (leading to formation of a quaternary carbon atom at C6 of the THP) and allenoate **2** **a** (R^3^=CO_2_Et, R^4^=Et) for the development of reaction conditions (see Supporting Information for details). Already in orienting reactions it was found that desired product **3** **a** was formed as a single diastereoisomer. Subsequent screening of chiral phosphines and solvents revealed the use of (*S,S*)‐Et‐BPE in THF as the most advantageous, affording the pseudo‐NP in 71 % yield with 88 % *ee* (Scheme 1).

With optimised conditions in hand, we explored the scope of the transformation and found that, gratifyingly, substrates bearing various substitutions were tolerated and allowed for the facile synthesis of a collection of apoxidole pseudo‐NPs (Table 1).

**Table 1:**
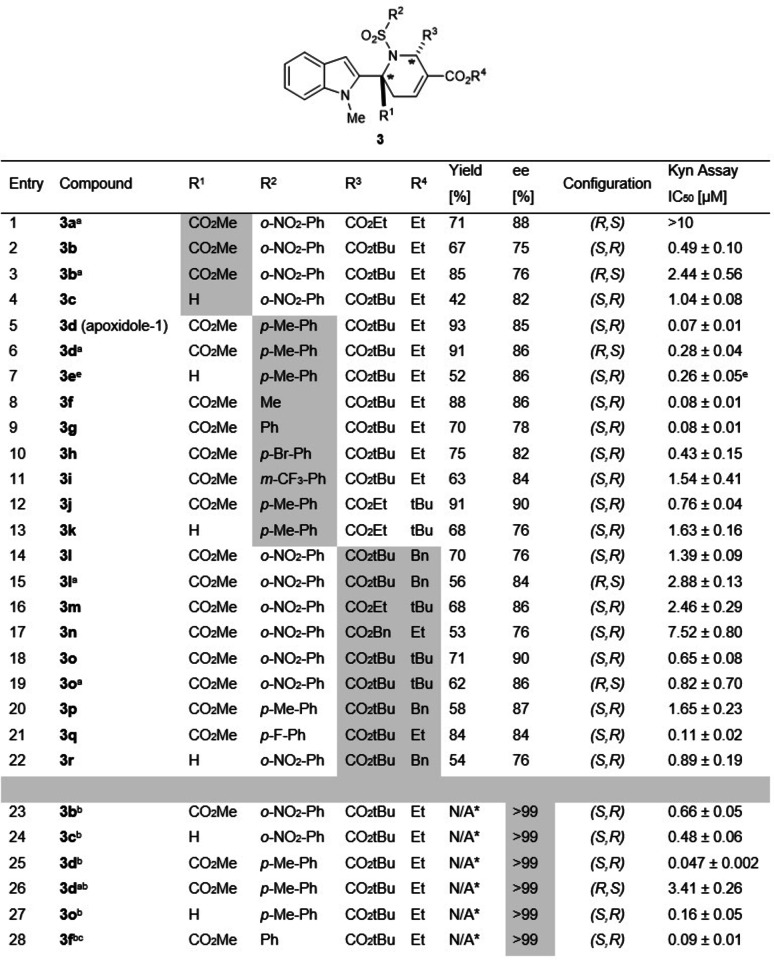
Results for the enantioselective synthesis of apoxidole pseudo‐NPs and structure activity relationship (SAR) for reduction of Kyn levels (See Supporting Information for further details). Kyn levels were determined in HeLa cells using the Kyn assay. Data are mean values (*n*=3) ±SD.

[a] Acquired using (*S*,*S*)‐Et‐BPE, [b] Purified on chiral preparative HPLC, IC column, DCM : EtOH (100 : 2)/*iso*‐hexane=30/70, flow rate=3 mL min^−1^. [c] Enantiomer purified using DCM:MeOH (100 : 5)/*iso*‐hexane=30/70. [d] Single enantiomer yields detailed in Supporting Information. [e] IC_50_ value obtained from automated Kyn Assay.

Pleasingly the reaction conditions also proceeded smoothly with the aldimine (Table 1, entry 4) providing moderate *ee*, albeit with lower yield. Exchange of the nosyl group with other sulphonamides gave high yields and good enantioselectivity (Table 1, entries 5–12. but the reaction failed with *N*‐*o‐*nitrophenyl ketimine. Various allenoates reacted successfully with ketimine **1** **a** and provided the desired products in moderate to high yields with good enantioselectivity (Table 1, entries 13–18). However, attempts to displace esters at R^3^ with H, an alkyl moiety, e.g. Et, or charge stabilising groups, e.g Ph, CN and COPh, resulted in trace or no product formation. The structure and stereochemistry were confirmed by X‐Ray analysis based on the major isomer (*R,S*)‐**3** **b** (Table 1, entry 3, see the Supporting Information). Yet, pure enantiomers of selected compounds were readily obtained by preparative chiral HPLC (Table 1, entries 20–25).

The pseudo‐NP collection was investigated in different cell‐based assays to evaluate their influence on various biological pathways, e.g. Wnt,^[14]^ Notch and Hedgehog (Hh) signalling,^[15]^ autophagy,[^3b^, ^16^] and modulation of kynurenine (Kyn) levels.^[9c]^ Apoxidole derivative (*S,R*)‐**3** **b** (Table 1, entry 2) selectively decreased cellular kynurenine (Kyn) levels in BxPC‐3 cells (Figure S1) upon stimulation with the cytokine Interferon‐γ (IFN‐γ). Imbalances in Kyn pathway metabolites such as Kyn itself induce immune‐suppression and can lead to diseases like neurodegeneration,^[17]^ autoimmune diseases^[18]^ and cancer.^[19]^ The three tryptophan‐catabolizing enzymes indoleamine 2,3‐dioxygenase 1 and 2 (IDO1 and IDO2) and tryptophan 2,3‐dioxygenase (TDO) catalyze the first and rate‐limiting step of the Kyn pathway.^[20]^ IDO1 is expressed in immune‐privileged organs such as the placenta, whereas expression of the *IDO1* gene can be induced by IFN‐γ in inflammatory and tumoral tissues.^[21]^ In contrast, TDO and IDO2 are mainly constitutively expressed in the liver or in immune cells, respectively,[^18a^, ^22^] and their expression cannot be induced by cytokines in cancer cells.[^18a^, ^23^] Therefore, compounds that modulate Kyn levels in the employed screening assay are most likely to interfere with IDO1 activity. Kyn levels can be reduced by small molecules that e.g. inhibit IDO1 directly,^[24]^ redox‐cycling compounds,^[25]^ decrease IDO1 expression, e.g. via inferring with JAK/STAT signalling,^[26]^ inactivate IDO1 via post‐translational modifications^[27]^ or prevent the uptake of the IDO1 substrate L‐tryptophan (L‐Trp).^[28]^

**Figure 2:**
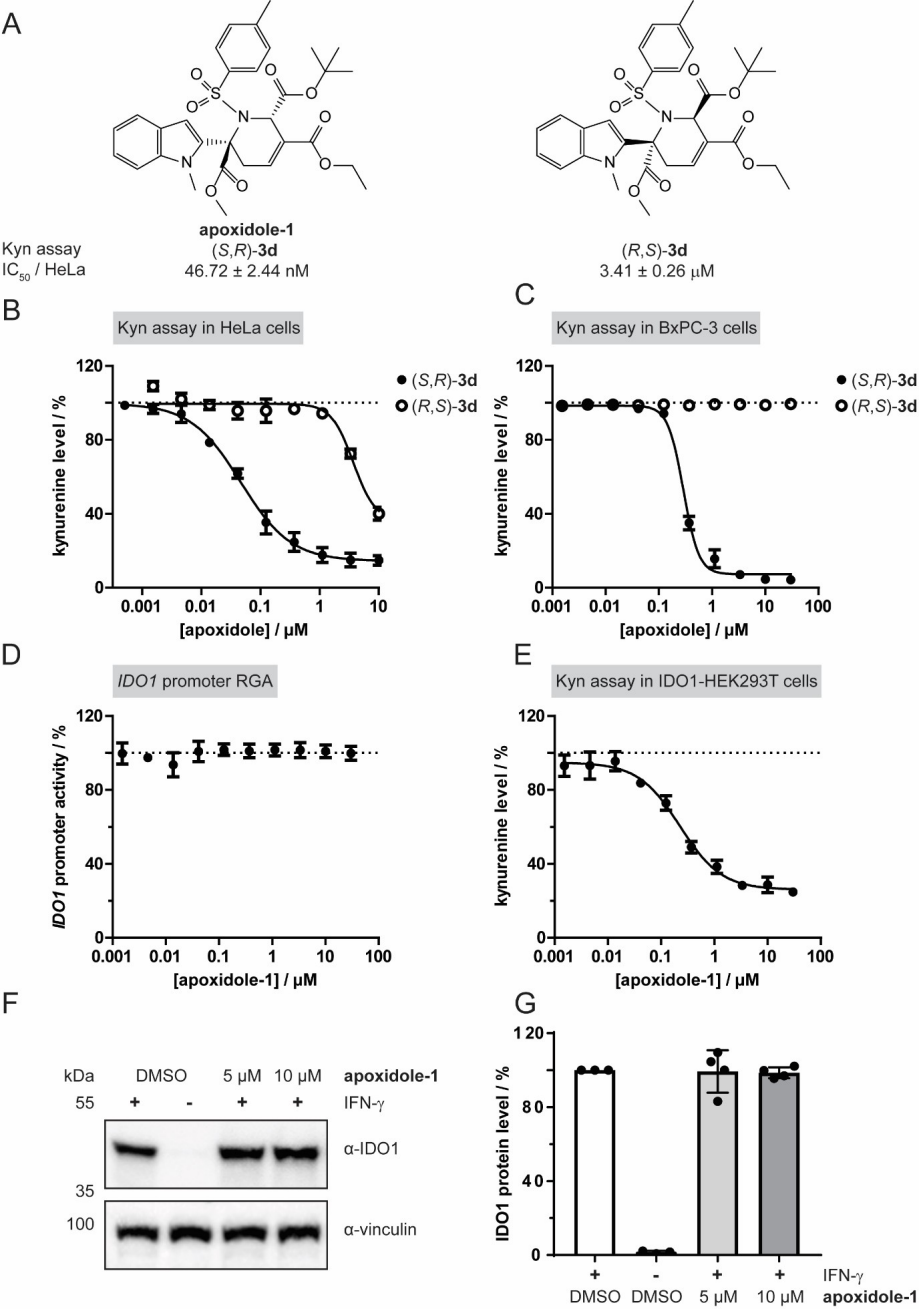
The pseudo‐NP apoxidoles reduce cellular kynurenine (Kyn) levels, but do not inhibit IDO1 expression. A) Structures and IC_50_ values of (*S*,*R*)‐**3** **d** (apoxidole‐1) and (*R*,*S*)‐**3** **d** (mean±SD, *n*=3). B) Determination of Kyn levels in HeLa cells upon treatment with IFN‐γ, L‐Trp and compounds for 48 h and detection of Kyn using *p*‐DMAB (mean±SD, *n*=3). C) Determination of Kyn levels in BxPC‐3 cells upon treatment with IFN‐γ, L‐Trp and compounds for 48 h and detection of Kyn levels using *p*‐DMAB (mean±SD, *n*≥3). D) Reporter gene assay (RGA) in HEK293T cells expressing firefly luciferase (Fluc) under the control of the *IDO1* promoter and constitutive *Renilla* luciferase expression. Expression of Fluc was induced by IFN‐γ with simultaneous treatment with apoxidole‐1 for 48 h (mean±SD, *n*=3). E) Kyn assay in HEK293T cells transiently expressing human IDO1 under the control of a CMV promoter. Cells were treated with L‐Trp and apoxidole‐1 for 24 h prior measuring Kyn levels with *p*‐DMAB (mean±SD, *n*=3). F, G) IDO1 protein levels in HeLa cells that were treated with IFN‐γ and apoxidole‐1 or DMSO for 24 h. Representative immunoblot shown in C (*n*=3), see also Figure S2. Quantification of IDO1 band intensities from C normalized to the loading control vinculin shown in D (mean±SD, *n*=3). Dotted lines in B, C, D, E) indicate the signal levels of cell that were treated with DMSO+IFN‐γ.

To gain an overview of an initial SAR, activities of enantioenriched compounds **3** **a‐3** **c** were determined. The initially determined activity of (*S,R*)‐apoxidole **3** **b** was confirmed in manual Kyn assays in BxPC‐3 and HeLa cells, using *p*‐DMAB instead of the Kyn sensor (Figure S1). Subsequent exploration of the structure‐activity‐relationship revealed that the mixture with the predominating enantiomer (*R,S*)*‐* **3** **b** was substantially less active with IC_50_=2.44±0.56 μM (entry 3). Interestingly, potency of the cycloadduct from the aldimine substrate lacking the methyl ester i.e. (*S,R*)‐**3** **c** (R^1^=H, entry 4) decreased by more than half, demonstrating the importance of the stereogenic quaternary centre (compare entries 2 and 4). Replacement of the nosyl group with other sulphonamide moieties at R^2^ (Table 1, entries 5–13) generally providing sub‐micromolar activity, in particular lipophilic structures such as the tosyl‐, methyl‐ and phenyl moieties (Table 1, entries 5–8). Tosylated apoxidole (*S,R*)‐**3** **d** (apoxidole‐1) displayed nanomolar activity, however, substitution with other electron‐poor aryl rings (Br and CF_3_, entries 9 and 10) led to diminished activity. Varying ester moieties on the allene core at R^3^ and R^4^ (Table 1, entries 14–19) revealed the optimal combination to be tertiary butyl and ethyl ester respectively. Finally, enantiomeric purification of selected compounds (Table 1, entries 23–28) demonstrated the substantial influence of the minor enantiomer. The enantiopure apoxidole (*S,R*)‐**3** **d** (apoxidole‐1) was found to be the most potent compound (Table 1, entry 25 and Figure S4) with an IC_50_ value of 46.7±2.4 nM, while its enantiomer was 72‐fold less active (Table 1, entry 26) in HeLa cells (Figure 2A, B) and completely inactive in BxPC‐3 cells (Figure 2C). The integrity of the compound under these conditions was confirmed by a serum stability analysis (Figure S16).

To explore if (*S,R*)*‐* **3** **d** (apoxidole‐1) reduces cellular Kyn levels by decreasing IDO1 expression, its impact on the *IDO1* promoter was analyzed by means of a reporter gene assay (Figure 2D). Apoxidole‐1 did not reduce the expression of the reporter, indicating that *IDO1* gene expression is not inhibited. Furthermore, apoxidole‐1 inhibited Kyn production in HEK293T cells in the absence of IFN‐γ when cells transiently expressed IDO1 under the control of a CMV promoter (Figure 2E). In line with these findings, *IDO1* mRNA levels were not reduced upon treatment with initially synthesized (*S,R*)*‐* **3** **d** (Figure S3A). Apoxidole‐1 did not alter the level of the IDO1 protein as demonstrated by means of immunoblotting and in‐Cell western (Figure 2F, G and Figure S3B and S3C). Thus, apoxidole‐1 decreases Kyn levels with and without stimulation with IFN‐γ, but does not reduce IDO1 expression and, thus, protein levels.

The IDO1 substrate L‐Trp can be transported into the cell by two different mechanisms. System L‐type amino acid transporters (LAT) serve as import route for large essential amino acids such as L‐Trp. LATs are responsible for the majority of L‐leucine (L‐Leu) uptake into cells and can be inhibited by saturating concentrations of L‐Leu (Figure S5A).[^28c^, ^29^] In addition, L‐Trp can be taken up by tryptophanyl‐tRNA synthetases (TrpRS) which are highly expressed upon treatment with IFN‐γ.^[30]^ TrpRS can be inhibited by the L‐Trp analogue and IDO1 inhibitor 1‐methyl‐L‐tryptophan (1‐MT, Figure S5A).[^28a^, ^28b^, ^30^] To analyze if apoxidole‐1 interferes with one of the uptake mechanisms, we starved BxPC‐3 cells for L‐Trp for 72 h in the absence or presence of IFN‐γ prior to detecting the uptake of supplemented L‐Trp by HPLC‐MS/MS. We observed higher L‐Trp uptake upon treatment with IFN‐γ (Figure S5B). This is due to the fact that protein levels of IFN‐γ‐inducible TrpRS are upregulated and, moreover, IDO1 expression leads to a higher demand for its substrate. While both L‐Leu and 1‐MT inhibit the uptake of L‐Trp, apoxidole‐1 does not interfere with the import of L‐Trp (Figure S5B).

**Figure 3:**
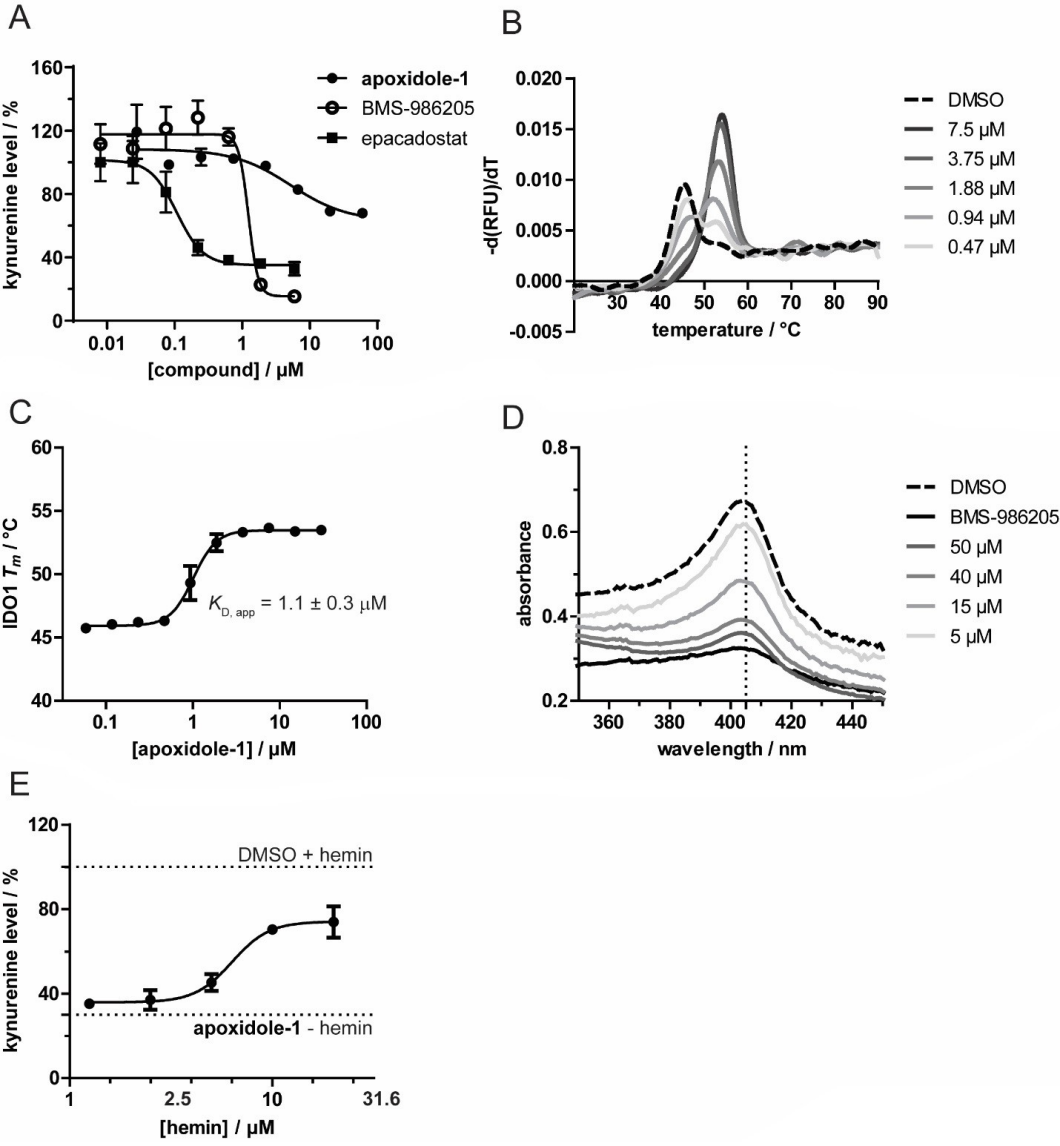
Apoxidole‐1 inhibits IDO1 by binding to apo‐IDO1 and releasing heme. A) Purified holo‐IDO1 protein was treated with apoxidole‐1, BMS‐986205 or epacadostat for 40 min at 37 °C prior to detection of Kyn levels using *p*‐DMAB (mean±SD, *n*=2). B) Dose‐dependent influence of apoxidole‐1 on the melting temperature of IDO1. Purified holo‐IDO1 was treated with apoxidole‐1 or DMSO for 60 min at 37 °C prior to detection of the intrinsic tryptophan/tyrosine fluorescence upon melting. Representative melting curves are shown (*n*=3, see also Figure S6–S9. C) Apparent *K*_D, app_ value for apoxidole‐1=1.09±0.31 μM, as determined using the data from B (mean±SD, *n*=3). D) UV/Vis spectra of IDO1. Purified holo‐IDO1 was treated with apoxidole**‐1**, BMS‐986205 (20 μM) or DMSO for 2 h at 37 °C prior to acquiring UV/Vis spectra. Representative spectra shown (*n*=3), see also Figure S10. The dotted line indicates the Soret peak at 404 nm. E) Determination of Kyn levels in presence of hemin. BxPC‐3 cells were treated with IFN‐ γ, L‐Trp, hemin and 600 nM apoxidole**‐1** for 48 h prior to measuring Kyn levels with *p*‐DMAB (mean±SD, *n*=3).

Subsequently, apoxidole‐1 was tested for direct inhibition of IDO1 in vitro (Figure 3A). Whereas the compound was inactive in the IDO1 enzymatic assay at 25 °C, after pre‐incubation at 37 °C apoxidole‐1 dose‐dependently decreased IDO1 activity. The observed inhibition is characteristic for small molecules that bind apo‐IDO and prevent binding of the cofactor heme such as BMS‐986205.[^9a^, ^9c^] Pre‐incubation of IDO1 with the compounds at 37 °C is required to identify apo‐IDO1 inhibitors as heme dissociation from holo‐IDO1 is a slow and reversible process. This explains the lower potency of this type of inhibitors in in vitro assays compared to in cellulo assays, e.g. BMS‐986205 has an IC_50_ value of 4.2 nM^[9a]^ in HeLa cells and 1.13±0.31 μM in the biochemical assay (Figure 3A). To demonstrate binding of apoxidole‐1 to IDO1, the thermal denaturation of IDO1 was analyzed using nano differential scanning fluorimetry (nanoDSF, Figure 3B and Figure S6). Treatment of holo‐IDO1 with apoxidole‐1 shifted the melting temperature *T*_m_ by 7.7±0.3 °C from 45.7±0.2 °C to 53.5±0.2 °C, showing that apoxidole‐1 directly binds to IDO1. In addition, treatment with different concentrations of apoxidole‐1 allowed determination of an apparent dissociation constant (*K*_D,app_) of 1.1±0.3 μM (Figure 3C). Furthermore, we performed an isothermal analysis^[31]^ of the nanoDSF melting temperature data to obtain a *K*_D_ value of 20.7±0.01 nM at 49 °C (Figure S7–S9). To investigate if binding of apoxidole‐1 induces heme loss, we employed UV/Vis spectroscopy to monitor the so‐called Soret peak (Figure 3D). The Soret peak describes the characteristic maximum in absorbance of heme‐containing enzymes at 404 nm. A red‐shift of the maximum indicates ligand coordination to the heme iron, while a decrease in the signal is indicative for heme loss.^[32]^ Apoxidole‐1 dose‐dependently reduced the Soret peak, proving that compound binding to IDO1 releases heme. In line with these findings, increasing concentrations of free hemin reduced the potency of apoxidole‐1 in the Kyn assay (Figure 3E).

Röhrig et al.^[24]^ described four different types of direct IDO1 inhibitors: I) tryptophan‐competitive inhibitors, binding to oxygen‐bound holo‐IDO1, II) oxygen‐competitive inhibitors, binding to free ferrous holo‐IDO1, III) inhibitors binding to free ferric holo‐IDO1 and IV) inhibitors binding to apo‐IDO1. In contrast to inhibitor types I–III, type IV inhibitors do not coordinate to the heme in the IDO1 active site, but target IDO1 exclusively in its heme‐free form. The failure in clinical trials of type II inhibitor epacadostat has halted clinical investigations of several other IDO1 inhibitor programmes,^[10]^ whilst the type IV inhibitor BMS‐986205 remains in late stage clinical trials (e.g. NCT03661320, NCT04106414, NCT03854032).^[11]^ Moreover, apo‐IDO1 inhibitors bind to a different physiologically relevant state compared to type I–III. Thus, novel type IV inhibitors are in high demand.

**Figure 4:**
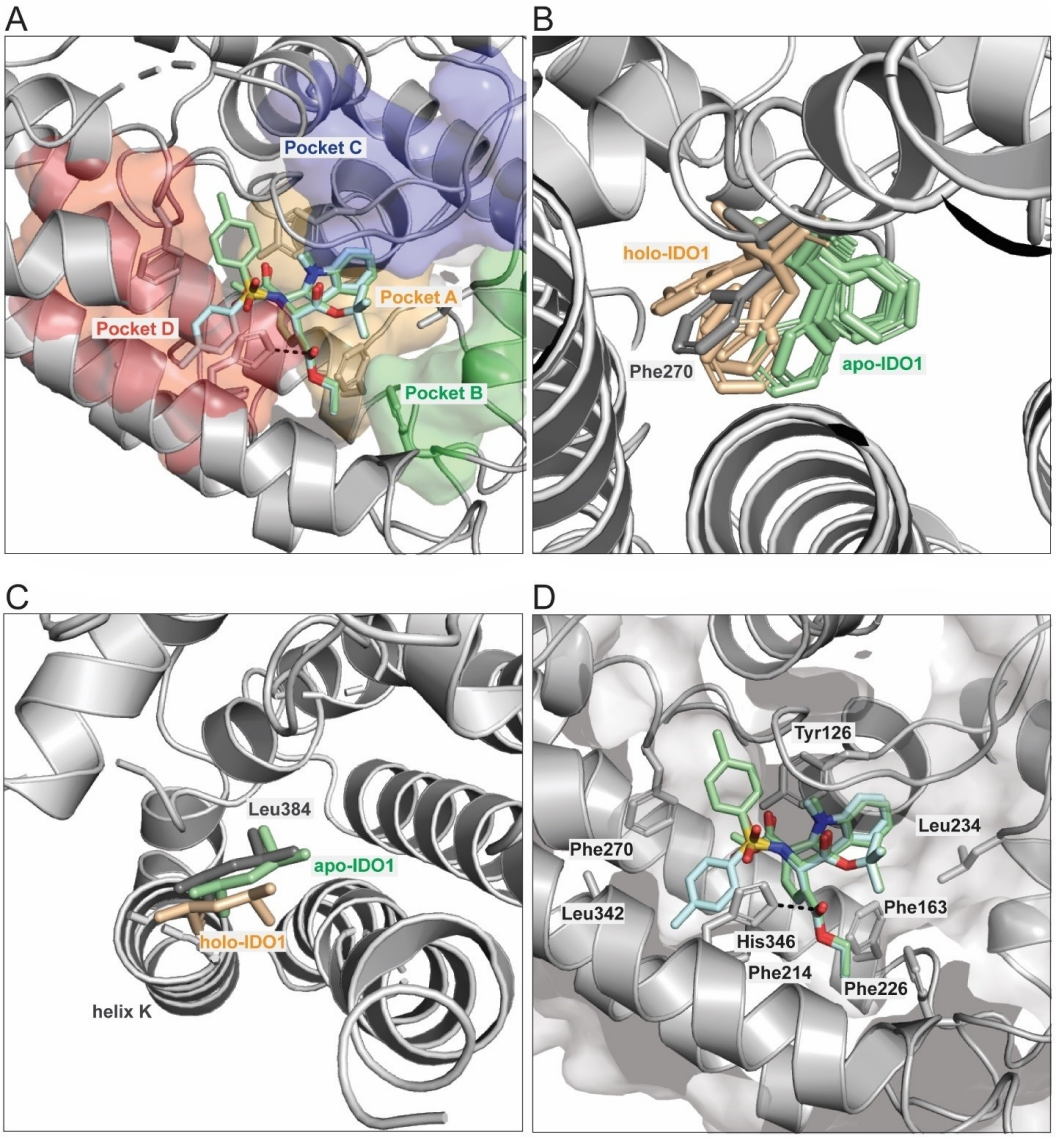
Crystal structure of apoxidole‐1 bound to apo‐IDO1 (pdb 8abx). A) Apoxidole‐1 occupies pockets A and D of apo‐IDO1. The IDO1 active site consists of four sub‐pockets: Pockets A (orange), B (green), C (blue) and D (red). Apoxidole‐1 displaces the heme cofactor and binds to pockets A and D. B) Conformation of Phe270. In apo‐IDO1 (green sticks, pdbs 6dpq, 6azv, 6azw, 6e43, 6v52, 6wpe, 6wjy, 6x5y, 7m63), Phe270 covers pocket D; whereas Phe270 is in an open conformation in holo‐IDO1 (orange sticks, pdbs 2d0t, 6e42, 6f0a, 6kw7, 7ah6) and the here described co‐crystal structure (grey sticks). C) Conformation of Leu384. In the here described crystal structure and apo‐IDO1 (gray and green sticks, pdb 6e43), Leu384 moves into the free heme‐binding pocket. In holo‐IDO1 (orange sticks, pdb 7ah6), Leu384 points out of the pocket. D) Secondary structure elements stabilizing apoxidole‐1 in the IDO1 active site. Apoxidole‐1 binds to the hydrophobic pocket of apo‐IDO1 (gray cartoons) in two different conformations (green and cyan sticks, see also Figure S14). The amino acids in the active site are labeled with the three‐letter code. The dotted black line indicates a hydrogen bond between His346 and the carbonyl oxygen of the ethyl ester of apoxidole‐1. Heteroatoms of the ligand are depicted in red (oxygen), blue (nitrogen) and yellow (sulfur). Amino acids 383–389 are omitted for clarity.

A co‐crystal structure of apoxidole‐1 with apo‐IDO1 was solved to determine the binding mode of the compound (Figure 4). The IDO1 active site consists of four sub‐pockets A to D, of which pockets A, B and C are normally separated from pocket D by the cofactor heme.[^12^, ^24^, ^33^] While pocket A is a binding site for ligands on the distal heme site of holo‐IDO1 (e.g. oxygen),^[24]^ pocket D is located on the proximal heme site.^[12]^ The crystal structure revealed that apoxidole‐1 binds to pockets A and D, occupying the heme‐binding site spanning both pockets (Figure 4A). Residues 360–383 belonging to the JK‐loop^[12]^ are not resolved in this structure, thus indicating a flexible structural element. In comparison to holo‐IDO1, in apo‐IDO1 residue Phe270 covers pocket D in absence of heme.[^12^, ^24^] Interestingly, when apoxidole‐1 is bound to apo‐IDO1, Phe270 has a different conformation compared to previously published co‐crystal structures of apo‐IDO1 inhibitors (Figure 4B, pdbs 6dpq, 6azv, 6azw, 6e43, 6v52, 6wpe, 6wjy, 6x5y, 7m63). Residue Phe270 adopts a conformation similar to structures of holo‐IDO1 without a proximal pocket D ligand (Figure 4B). Consequently, pocket D is in an open conformation and allows for solvent exchange between helices E and F.^[12]^ Furthermore, Leu384 on the N‐terminal side of helix K points into the free heme‐binding pocket as typically observed for structures of apo‐IDO1 (Figure 4C).^[12]^ The ligand is framed by the hydrophobic amino acids Tyr126, Phe163, Phe214, Phe226, Leu234, Phe270, and Leu342 and is additionally stabilized by polar interactions between the carbonyl oxygen of the ethyl ester of apoxidole‐1 and His346 which is normally coordinated to the proximal site of heme in holo‐IDO1 (Figure 4D).^[12]^ Similar to other co‐crystal structures,^[12]^ we observed unexplained electron density in close proximity to the tosyl in pocket D. In contrast to the ligand core, the tosyl appendage of apoxidole‐1 is flexible (Figure 4D, see also Figure S14). Hence, we modelled two different conformations of the ligand with the tosyl pointing in different directions (green and cyan sticks, Figure 4D, see also Figure S14). Considering this and the large free space in pocket D, this part of the ligand structure could be further derivatized to achieve higher occupancies, possibly improving the affinity to IDO1.

**Figure 5:**
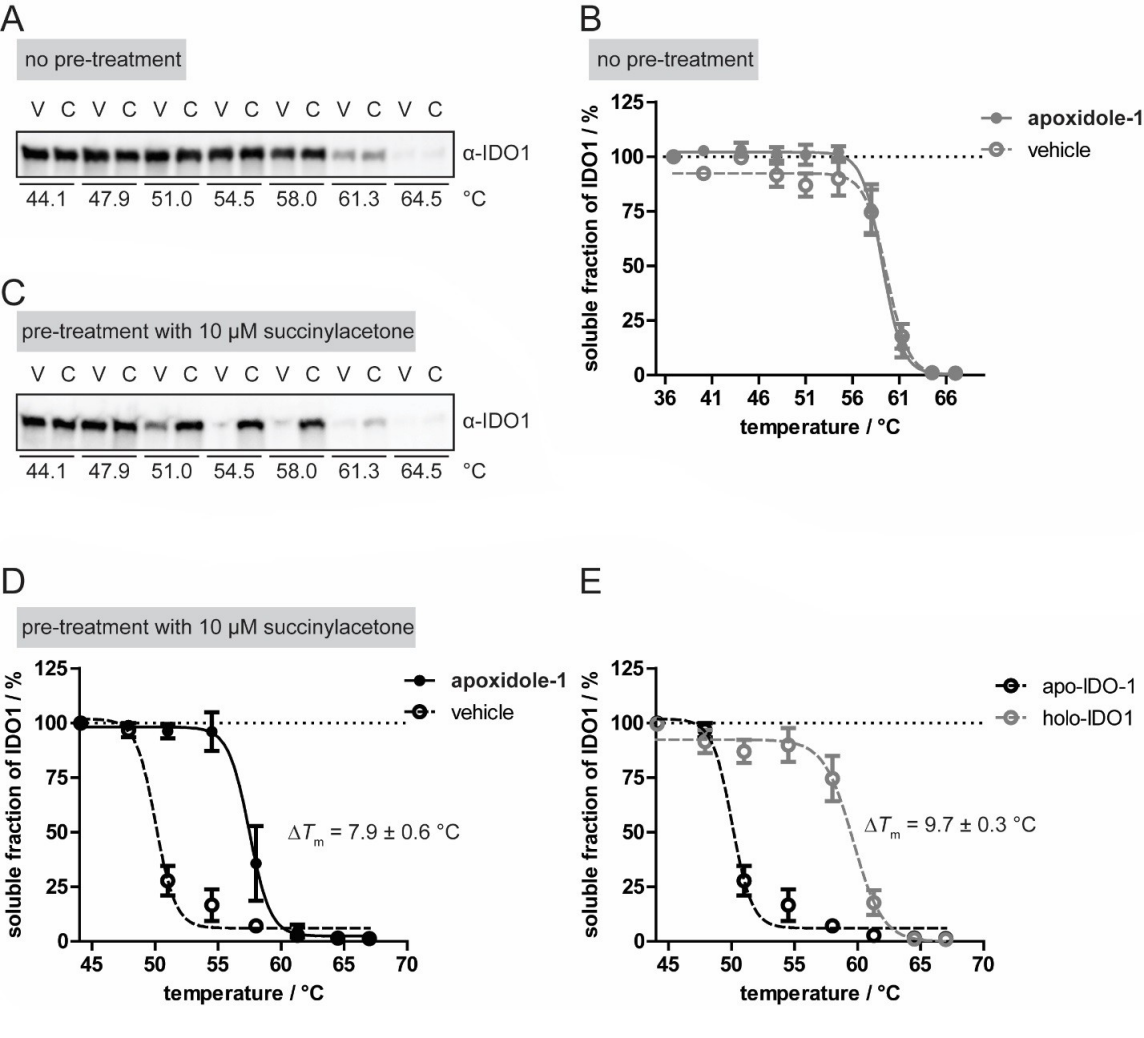
Apoxidole‐1 binds to apo‐IDO1 in cells. A) Cellular thermal shift assay (CETSA) for IDO1 in SKOV‐3 cells. Cells were treated with IFN‐γ for 24 h prior to addition of 50 μM apoxidole‐1 or DMSO for 15 min. Representative immunoblot shown (*n*=4), see also Figure S11. B) Thermal profiles of IDO1 upon compound treatment. Quantification of IDO1 band intensities from A shown in B (mean±SD, *n*=4). C) CETSA for apo‐IDO1 in SKOV‐3 cells. Cells were treated with IFN‐γ and 10 μM succinylacetone (heme synthesis inhibitor, SA) for 24 h prior to addition of 30 μM apoxidole‐1 or DMSO for 15 min. Representative immunoblot shown (*n*=3), see also Figure S12. D) Thermal profiles of IDO1 upon compound treatment. Quantification of IDO1 band intensities from C shown in D (mean±SD, *n*=3). E) Thermal profiles of holo‐ (in absence of SA) and apo‐IDO1 (in presence of SA). V: vehicle. C: compound. See Figure S11 and S12 for uncropped blots.

To prove target engagement in cells, we investigated the thermal stability of IDO1 in a cellular thermal shift assay (CETSA, Figure 5). Treatment of SKOV‐3 cells with apoxidole‐1 for 15 min did not lead to a shift in *T*_m_ compared to vehicle‐treated cells (Figure 5A and B). On the contrary, treatment with BMS‐986205 caused a clear stabilization of IDO1 with a Δ*T*_m_ of 5.6±1.0 °C (Figure S13). Considering the higher affinity of BMS‐986205 to IDO1, most likely, the treatment time was too short to allow heme displacement by apoxidole‐1 and subsequent binding to apo‐IDO. Therefore, cells were pre‐treated with the heme synthesis inhibitor succinylacetone (SA) 24 h prior to CETSA to ensure that apo‐IDO1 is the predominant form during compound treatment. Upon treatment with apoxidole‐1, a shift in *T*_m_ of 7.9±0.6 °C was observed, which correlates with the detected Δ*T*_m_ from the nanoDSF experiment (Figure 5C and D). Moreover, we detected different melting behaviour for holo‐ and apo‐IDO1 with an increase in *T*_m_ from 50.2±0.2 °C for apo‐IDO1 (i.e. in the presence of the heme synthesis inhibitor) to 59.9±0.2 °C for holo‐IDO (i.e. in the absence of the heme synthesis inhibitor, Figure 5E). This finding indicates that holo‐IDO1 exhibits higher thermal stability than apo‐IDO1 and binding of heme or apoxidole‐1 to apo‐IDO1 stabilizes the protein.

**Figure 6:**
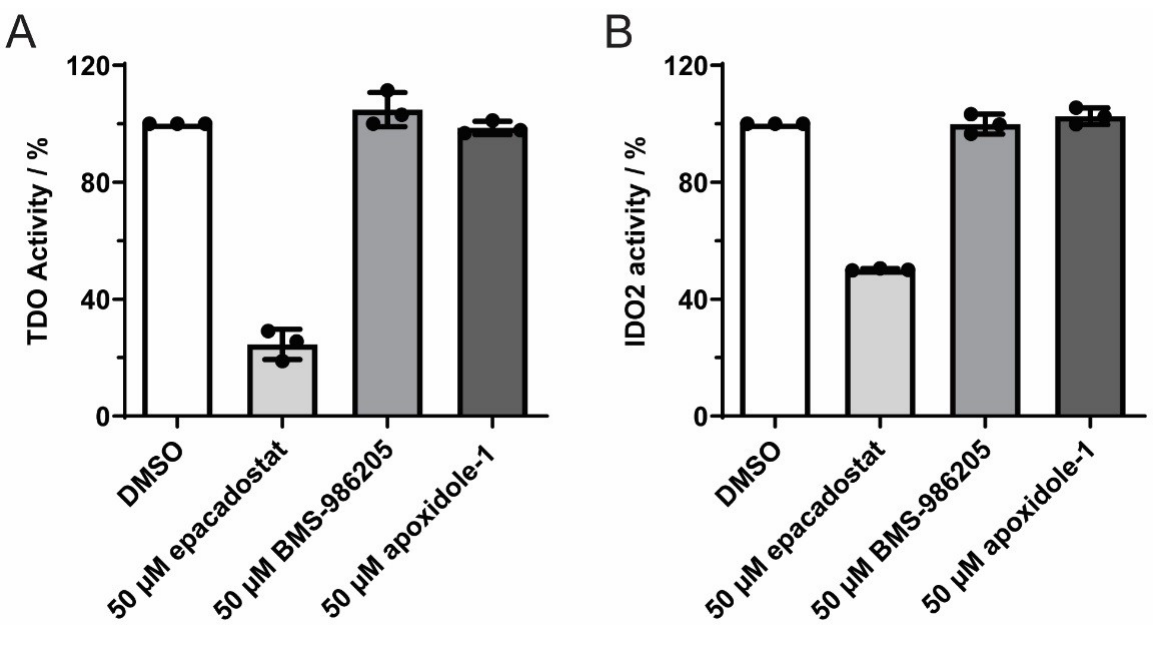
Apoxidole‐1 does not inhibit TDO and IDO2. A) Purified TDO protein was treated with epacadostat, BMS‐986205 or apoxidole‐1 for 120 min at 37 °C prior to detection of the reaction product (mean±SD, *n*=3). B) Purified IDO2 protein was treated with epacadostat, BMS‐986205 or apoxidole**‐1** for 90 min at 30 °C prior to addition of the IDO2 substrate. Samples were incubated for another 120 min at 30 °C prior to detection of the reaction product (mean±SD, *n*=3).

To investigate if apoxidole‐1 selectively targets IDO1, we investigated inhibition of the two other tryptophan‐catabolizing heme‐containing enzymes TDO and IDO2 (Figure 6).^[34]^ The development of dual and pan inhibitors may overcome resistance to immunotherapy,^[35]^ however, the highly potent compounds epacadostat and BMS‐986205 show high selectivity for IDO1^[36]^ (IDO1 and IDO2 share 44 % sequence homology^[20]^). The type II IDO1 inhibitor epacadostat competes with oxygen for free ferrous holo‐IDO1^[24]^ and inhibits purified IDO1 with an IC_50_ of 73 nM,^[36b]^ showing a >1000‐fold selectivity over TDO and IDO2.^[36b]^ In line with these findings, we observed that 50 μM epacadostat reduces TDO and IDO2 activity by 75.5±4.3 % and 49.9±0.3 %, respectively. On the contrary, the apo‐IDO1 inhibitors BMS‐986205 and apoxidole‐1 did not inhibit TDO or IDO2, suggesting that type IV inhibitors selectively bind to and inhibit IDO1.

## Conclusion

In conclusion, we designed and developed a novel pseudo‐NP class, termed apoxidoles. Apoxidoles are efficiently accessible by means of a stereoselective [4+2] annulation reaction combining indole‐ and tetrahydropyridine fragments in a mono‐podal manner. Initial biological evaluation in various cell‐based assays showed that the potent apoxidole‐1 reduces cellular Kyn levels. An in‐depth analysis revealed that apoxidole‐1 defines a novel type IV IDO1 inhibitor chemotype, selectively targeting apo‐IDO1. Since the type IV inhibitor BMS‐986205 persists under active late stage clinical investigation, whereas other types of IDO1 modulators have either failed or are currently not investigated anymore, novel type IV ligands, like apoxidole‐1, are in high demand.

## Conflict of interest

The authors declare no conflict of interest.

1

## Supporting information

As a service to our authors and readers, this journal provides supporting information supplied by the authors. Such materials are peer reviewed and may be re‐organized for online delivery, but are not copy‐edited or typeset. Technical support issues arising from supporting information (other than missing files) should be addressed to the authors.

Supporting Information

## Data Availability

The data that support the findings of this study are available from the corresponding author upon reasonable request.
