# Supplementary material for: Identification of a Novel Pseudo‐Natural Product Type IV IDO1 Inhibitor Chemotype
Source: Angew Chem Int Ed Engl. 2022 Aug 29;61(40):e202209374. doi: 10.1002/anie.202209374 (PMC9804781; doi:10.1002/anie.202209374)
Supplement: Supplementary file 1 — Supporting Information [file ANIE-61-0-s001.pdf]

Angewandte Chemie
Eine Zeitschrift der Gesellschaft Deutscher Chemiker

Supporting Information

Identification of a Novel Pseudo-Natural Product Type IV IDO1 Inhibitor Chemotype

*C. Davies, L. Dötsch, M. G. Ciulla, E. Hennes, K. Yoshida, R. Gasper, R. Scheel, S. Sievers, C. Strohmann, K. Kumar, S. Ziegler, H. Waldmann**

Table of Contents

Table of Contents 1
1. Supplementary Figures 2
2. Chemical Synthesis Methods 23
2.1. General Information 23
2.2 Reaction Optimization 24
2.2.1 *N*-Substituted Indole Racemic Reaction Screen 24
2.2.2 Chiral Phosphine Catalyst Screen 24
2.2.3 Solvent Screen 26
2.3 General Procedures and Analytical Data 26
2.3.1 General Procedure for the Synthesis of α-Ketoester 26
2.3.2 General procedure for the Synthesis of Ketimines from Precursor α-Ketoester 27
2.3.3 General Procedure for the Synthesis of Aldimines from Aldehyde 31
2.3.4 General Procedure for the Synthesis of Allenoates 33
2.3.5. General Procedure for the Synthesis of Apoxidoles 36
3. X-Ray Structure Analyses 75
4. Biological Methods 78
5. Author Contributions 85
6. Supplementary References 86
7. Publication Licenses 88
8. NMR Spectra 91

1. Supplementary Figures

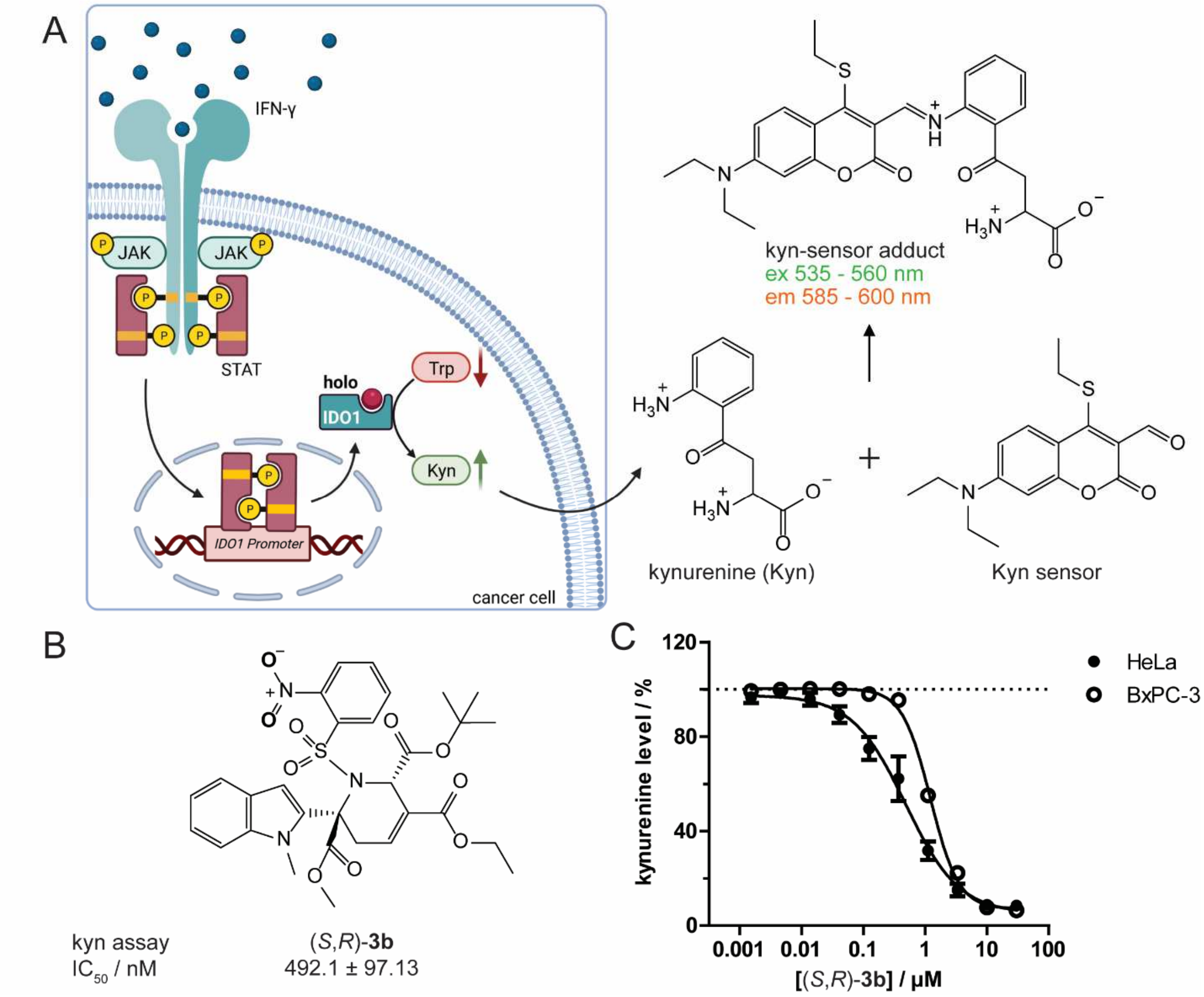

Figure S1. The pseudo-NP (*S,R*)-**3b** reduces cellular Kyn levels in an automated cellular assay. Principle of the assay. A) Determination of Kyn levels in BxPC-3 cells upon treatment with IFN-γ, L-Trp and compounds for 48 h and detection of Kyn levels with a fluorescence-based sensor. Created with BioRender.com. B) Structure of (*S,R*)-**3b** and IC_{50} ± SD value (n = 3) in HeLa cells. C) Determination of Kyn levels in HeLa and BxPC-3 cells upon treatment with IFN-γ, L-Trp and (*S,R*)-**3b** for 48 h and detection of Kyn levels using *para*-dimethylaminobenzaldehyde (*p*-DMAB) (mean ± SD, n = 3). Dotted line indicates Kyn levels in cells that were treated with DMSO+IFN-γ.

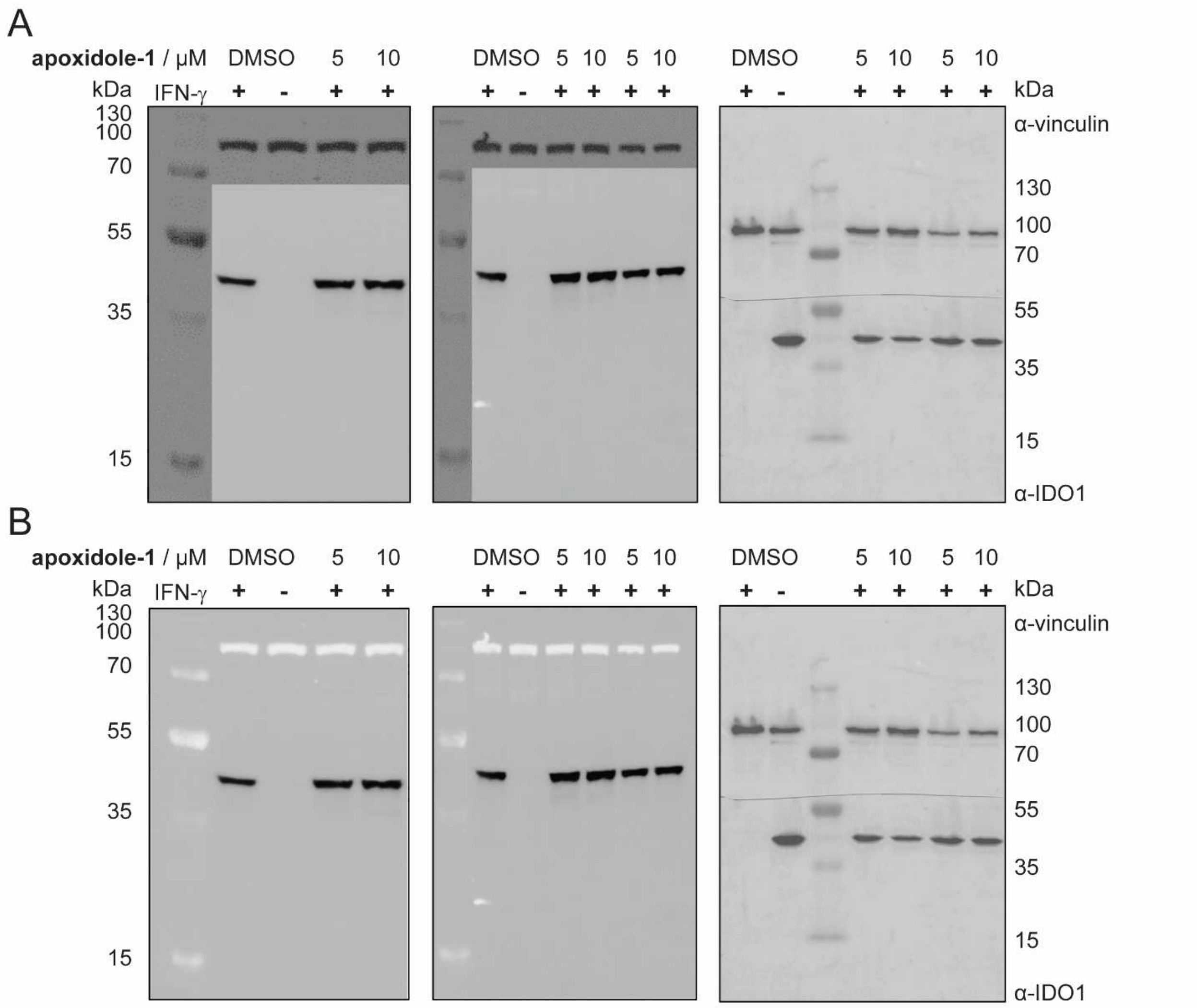

Figure S2. Influence of apoxidole-1 on IDO1 expression. Related to Figure 2. A-B) IDO1 protein levels in HeLa cells that were treated with IFN-γ and apoxidole-1 or DMSO for 24 h. Immunoblots of all biological replicates are shown. Unmodified immunoblots from A) are shown in B).

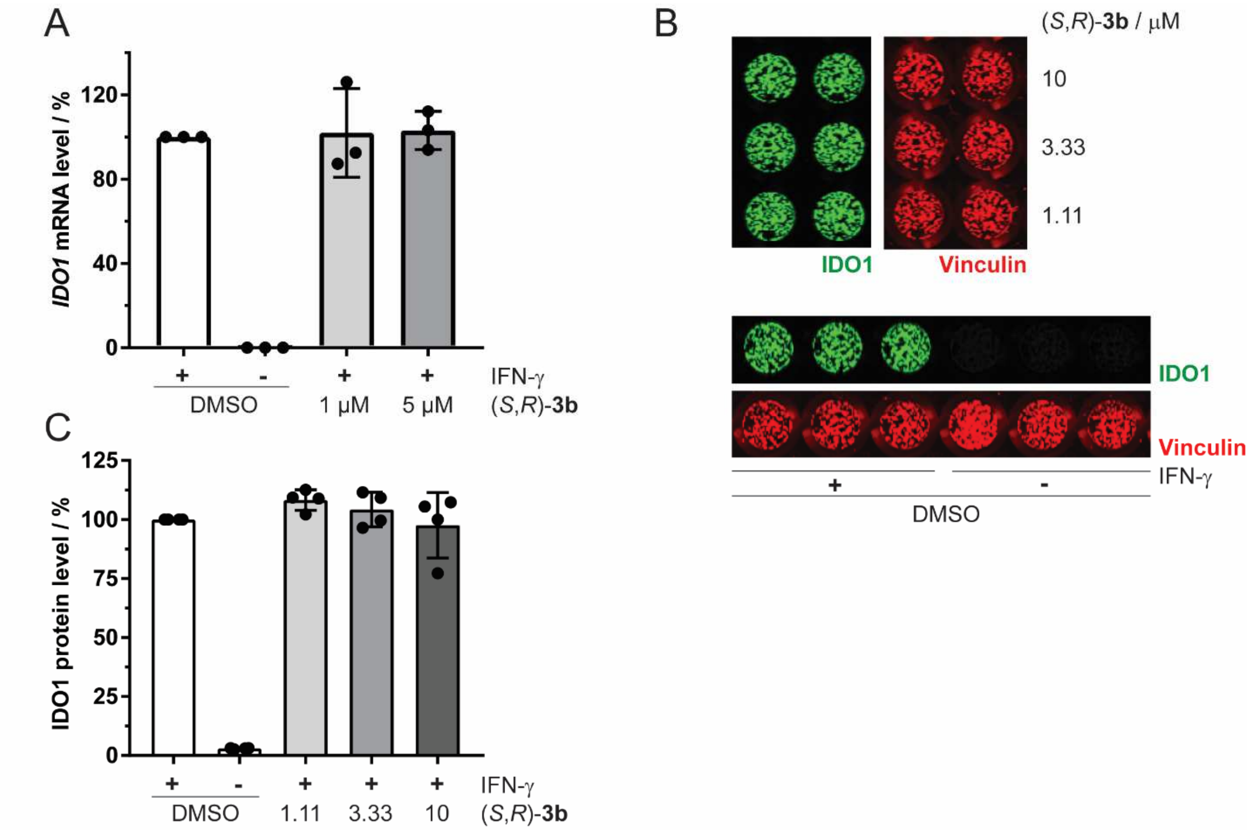

Figure S3. Influence of (*S,R*)-**3b** on IDO1 expression. A) *IDO1* mRNA expression in HeLa cells upon treatment with IFN-γ and (*S,R*)-**3b** or DMSO for 24 h (mean ± SD, n = 3). B and C) In-Cell Western of IDO1 protein levels in BxPC-3 cells upon treatment with IFN-γ and (*S,R*)-**3b** or DMSO for 24 h. Representative images shown in B (n = 4). Quantification of IDO1 signal from B normalized to the reference protein vinculin is shown in C (mean ± SD, n = 4).

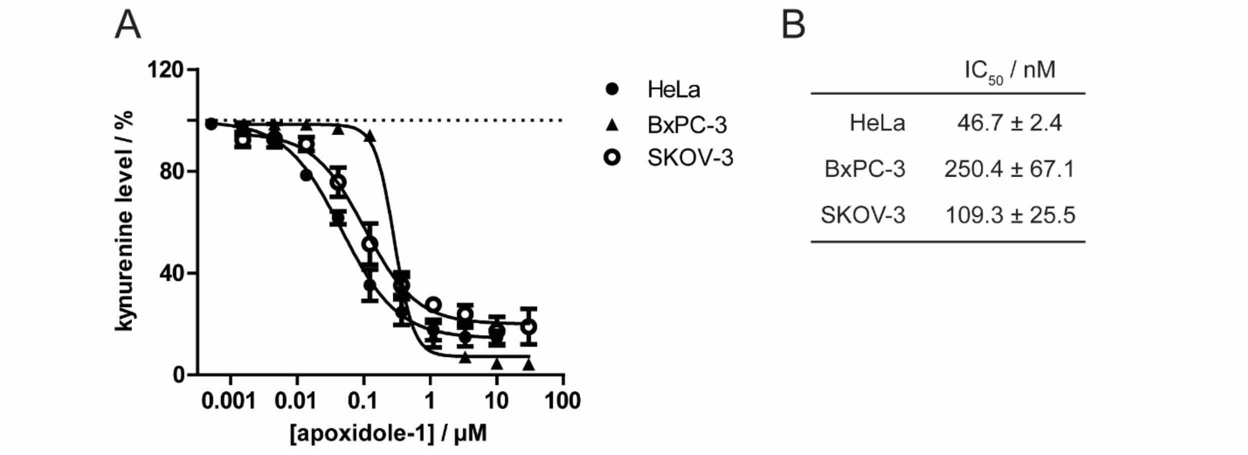

Figure S4. Apoxidole-1 reduces Kyn levels *in cellulo*. A) Determination of Kyn levels in HeLa, BxPC-3 and SKOV-3 cells upon treatment with IFN-γ, L-Trp and compound apoxidole-1 for 48 h and detection of Kyn levels using *p*-DMAB (mean ± SD, n ≥ 3). Dotted line indicates Kyn levels of the DMSO+IFN-γ control. B) IC50 values for apoxidole-1 obtained from A (mean ± SD, n ≥ 3).

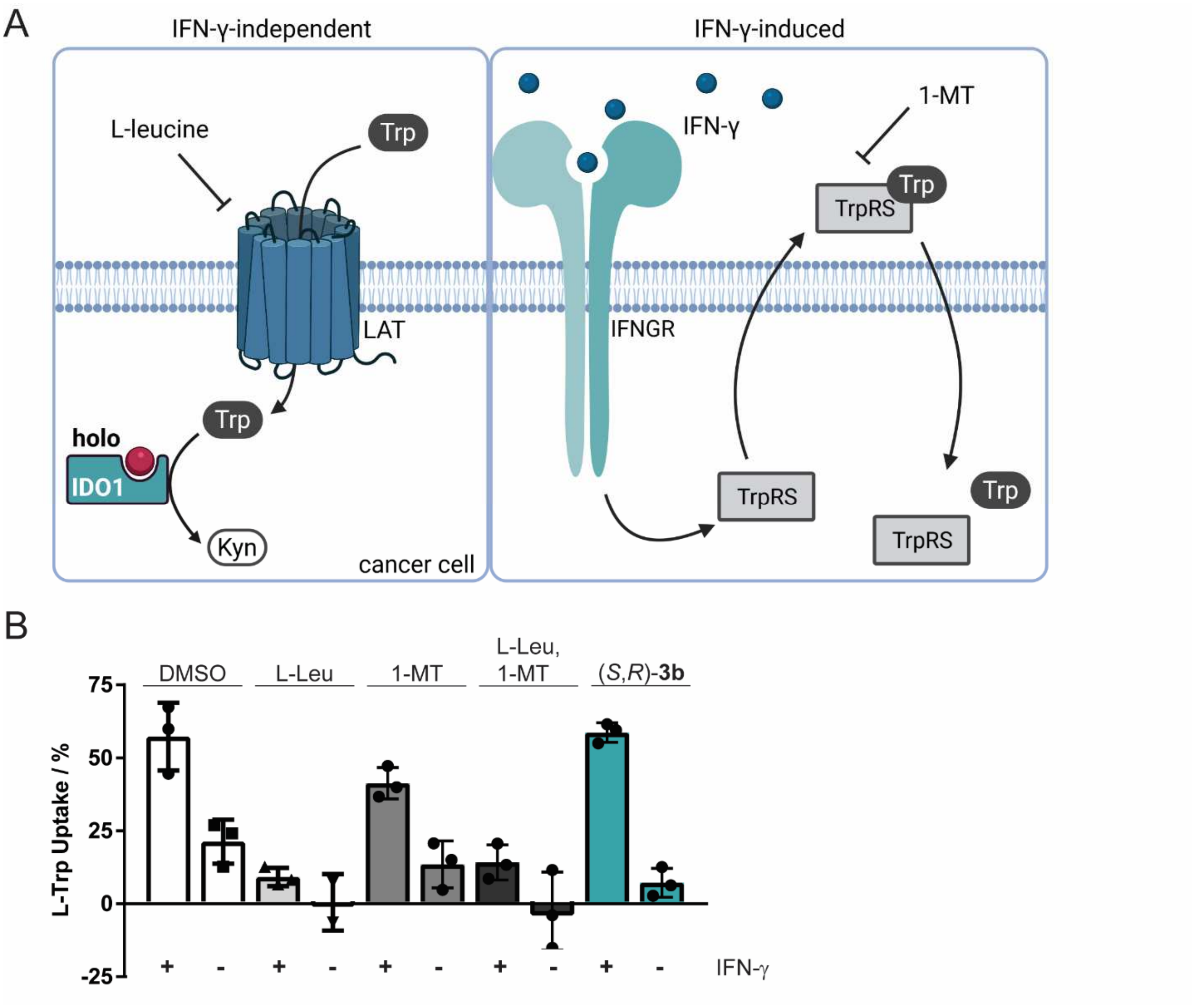

Figure S5. Influence of (*S,R*)-**3b** on L-Trp uptake. A) Two different L-Trp uptake mechanisms. L-Trp can be taken up IFN-γ-independently via amino acid transporters of the LAT family which can be inhibited by addition of saturating concentrations of L-Leu (left). Secondly, L-Trp can be taken up by TrpRS which is highly expressed upon treatment with IFN-γ and can be inhibited by the L-Trp analogue 1-MT. Created with BioRender.com. B) BxPC-3 cells were starved for L-Trp for 72 h and treated with IFN-γ for 24 h prior to addition of control inhibitors (5 mM L-Leu, 1 mM 1-MT) or 5 µM (*S,R*)-**3b** for 30 min. Afterwards, 50 µM L-Trp was added and L-Trp uptake was quantified with HPLC-MS/MS (mean ± SD, n = 3). IFNGR: Interferon-gamma receptor. LAT: system L-amino acid transporter. L-Leu: L-leucine. 1-MT: 1-methyl-L-tryptophan. TrpRS: tryptophanyl-tRNA synthetase.

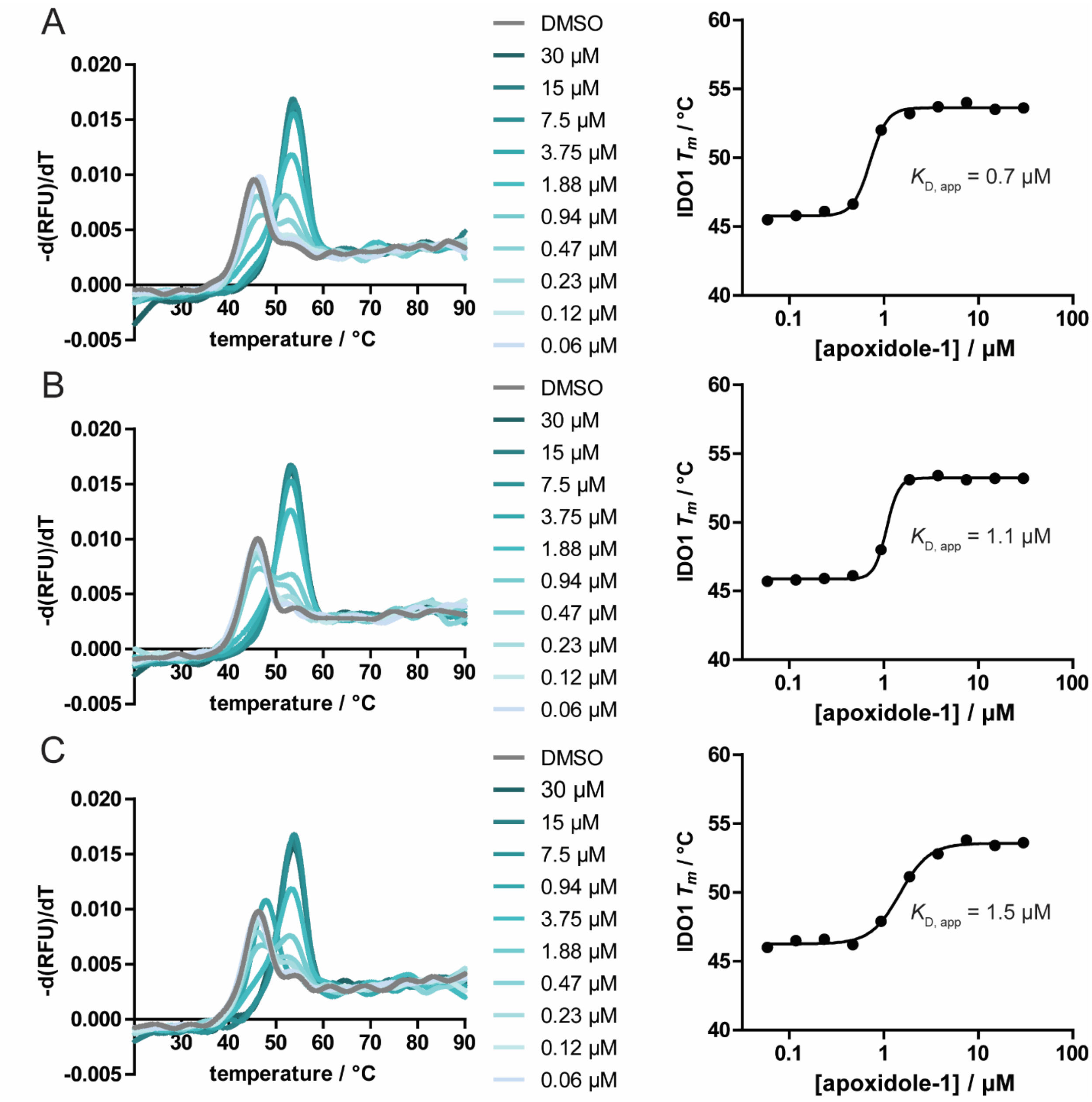

Figure S6. Influence of compounds on the IDO1 thermal denaturation temperature. Related to Figure 3. A-C) Dose-dependent influence of apoxidole-1 on the melting temperature of IDO1. Purified holo-IDO1 was treated with apoxidole-1 or DMSO for 60 min at 37°C prior to detection of the intrinsic tryptophan/tyrosine fluorescence upon melting. Three biological replicates of nanoDSF experiment (left) with respective apparent K_D value determination (right) are shown. $K_{D,\ app}$(apoxidole-1) = 1.1 ± 0.3 µM.

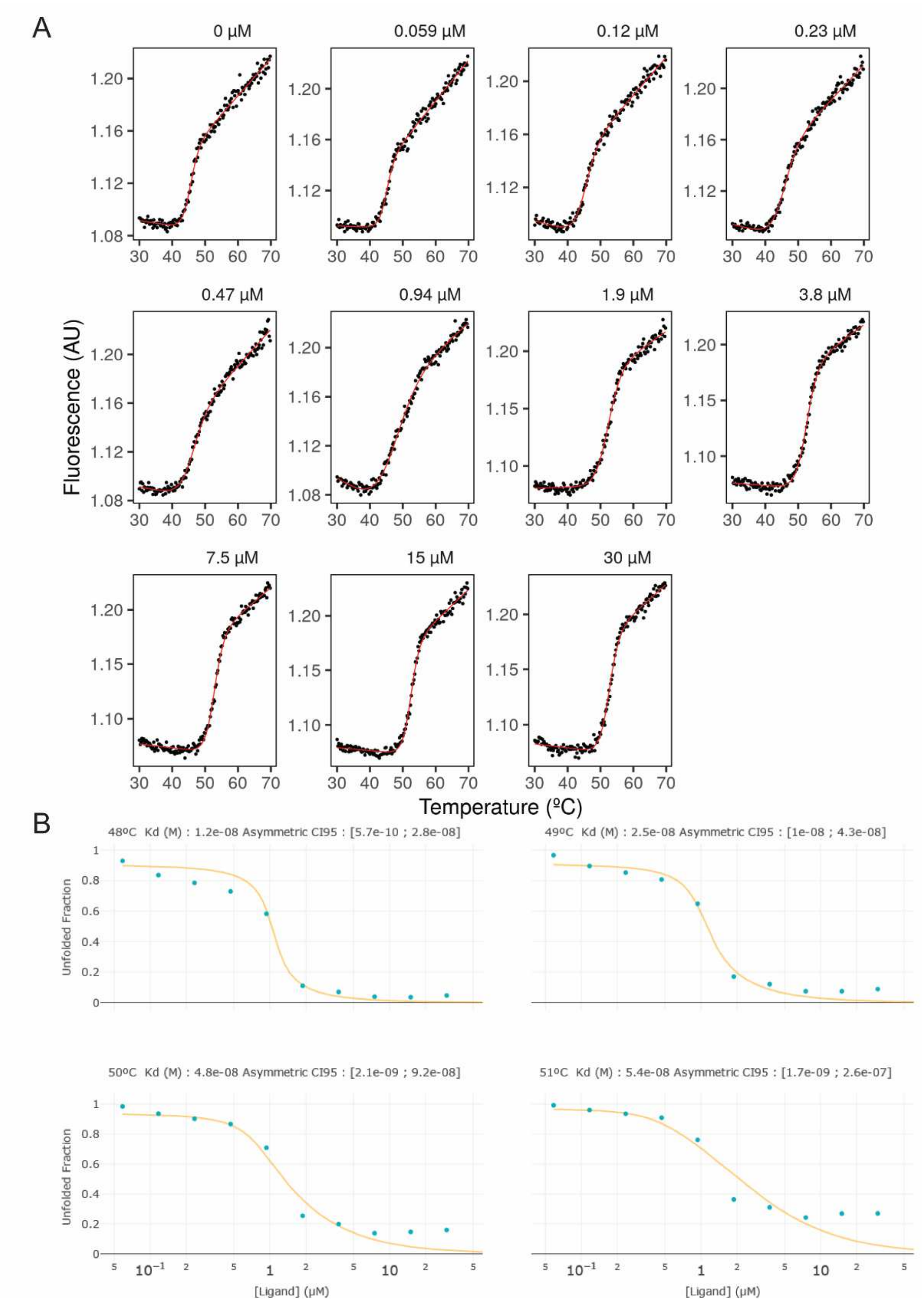

Figure S7. Isothermal analysis of the first replicate of the nanoDSF data from figure S6. A) Fitting of the fluorescence-based thermal denaturation curves of IDO1 at different concentrations of apoxidole-1. B) Isothermal analysis of the nanoDSF data from figure S6 A) at 48-51°C (2.3°C above the protein T_m). Plots created by FoldAffinity[1] (spc.embl-hamburg.de). $K_{D,\ 48°C}$ = 11.6 nM; $K_{D,\ 49°C}$ = 24.7 nM; $K_{D,\ 50°C}$ = 48.4 nM; $K_{D,\ 51°C}$ = 54.4 nM.

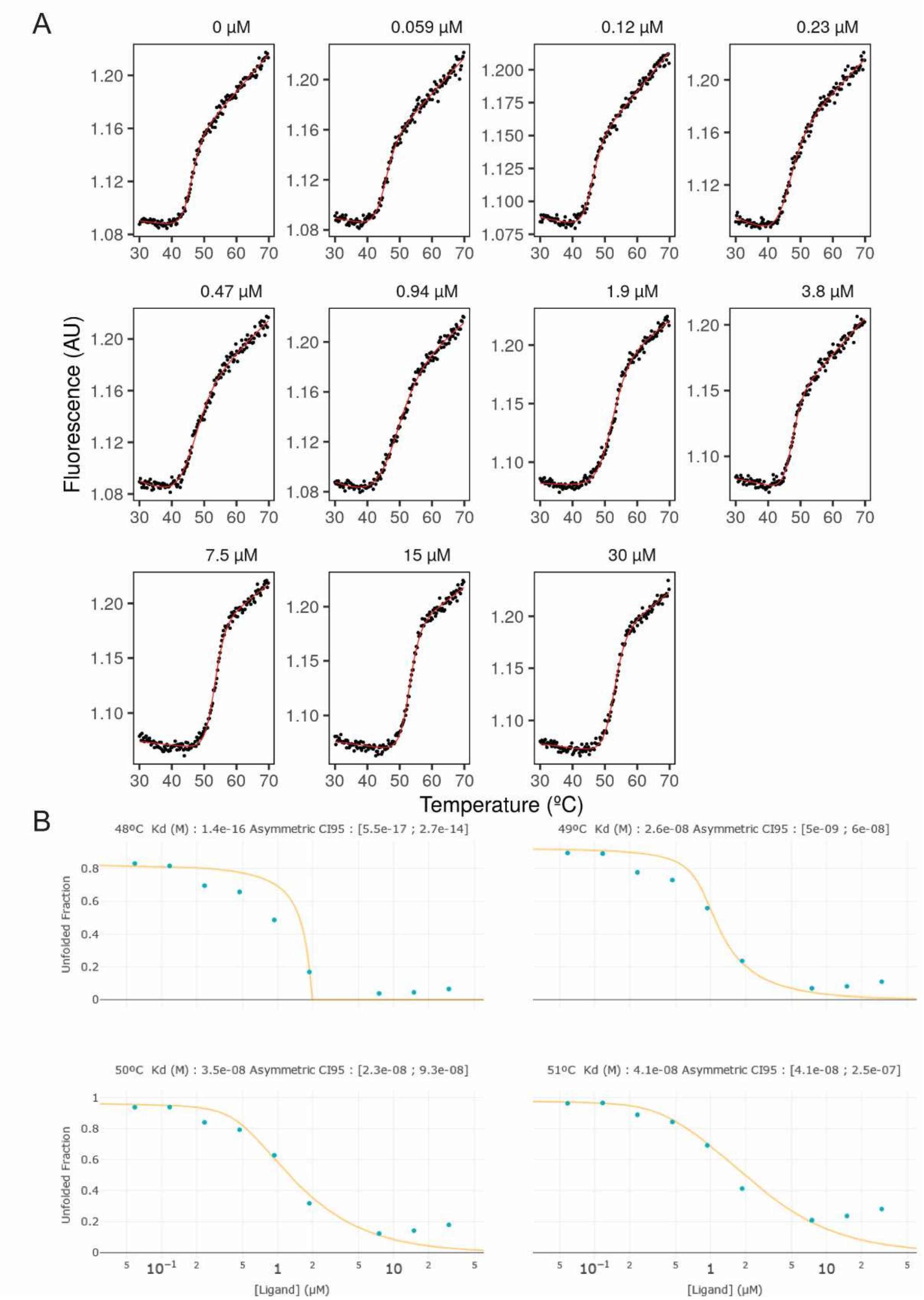

Figure S8. Isothermal analysis of the second replicate of the nanoDSF data from figure S6. A) Fitting of the fluorescence-based thermal denaturation curves of IDO1 at different concentrations of apoxidole-1. B) Isothermal analysis of the nanoDSF data from figure S6 B) at 48-51°C (2.3°C above the protein T_m). Plots created by FoldAffinity[1] (spc.embl-hamburg.de). $K_{D,\,48°C}$ = 142.3 aM; $K_{D,\,49°C}$ = 26.0 nM; $K_{D,\,50°C}$ = 34.7 nM; $K_{D,\,51°C}$ = 41.3 nM.

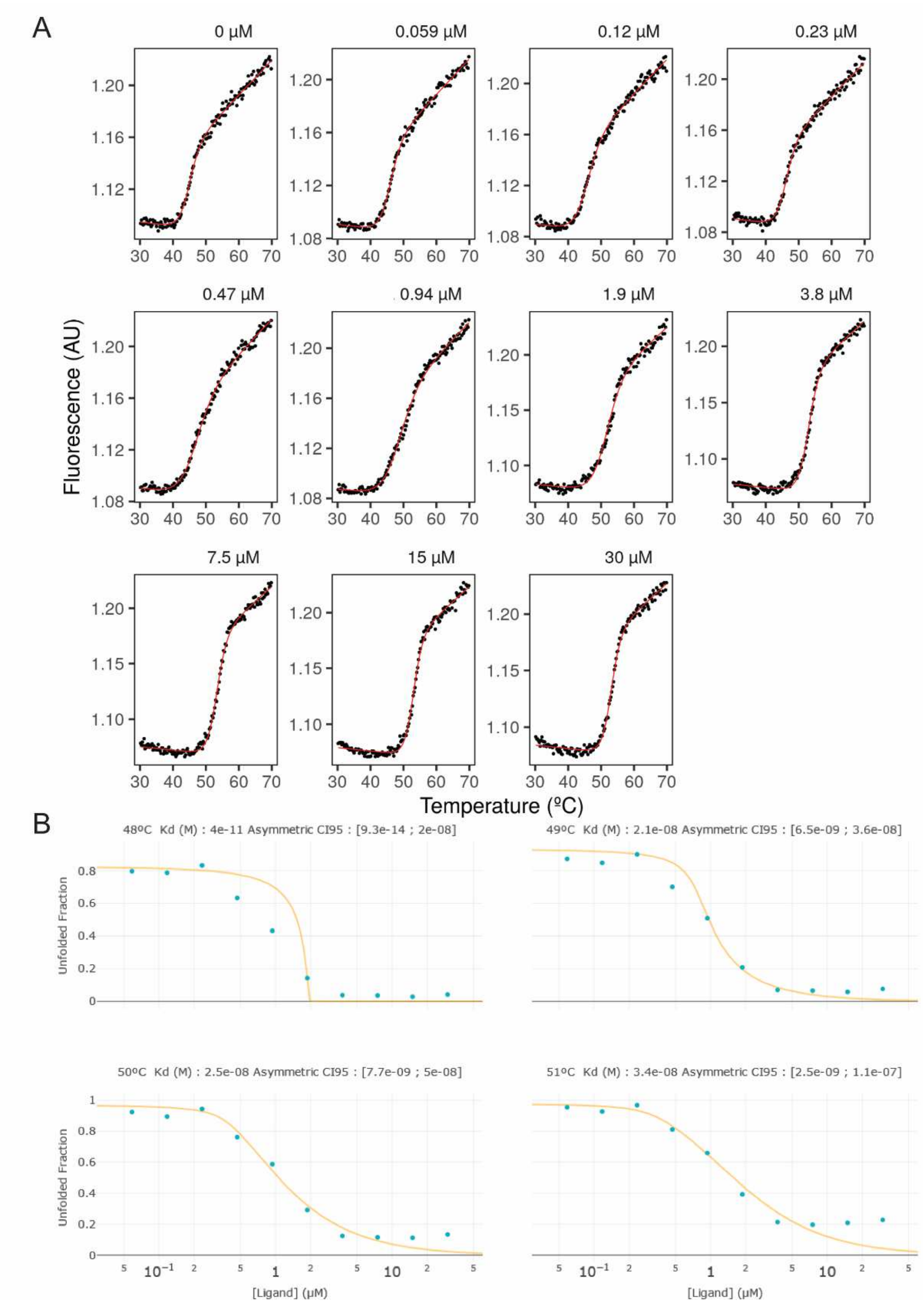

Figure S9. Isothermal analysis of the third replicate of the nanoDSF data from figure S6. A) Fitting of the fluorescence-based thermal denaturation curves of IDO1 at different concentrations of apoxidole-1. B) Isothermal analysis of the nanoDSF data from figure S6 C) at 48-51°C (2.3°C above the protein T_m). Plots created by FoldAffinity[1] (spc.embl-hamburg.de). $K_{D,\ 48°C}$ = 39.7 pM; $K_{D,\ 49°C}$ = 20.7 nM; $K_{D,\ 50°C}$ = 24.8 nM; $K_{D,\ 51°C}$ = 33.6 nM.

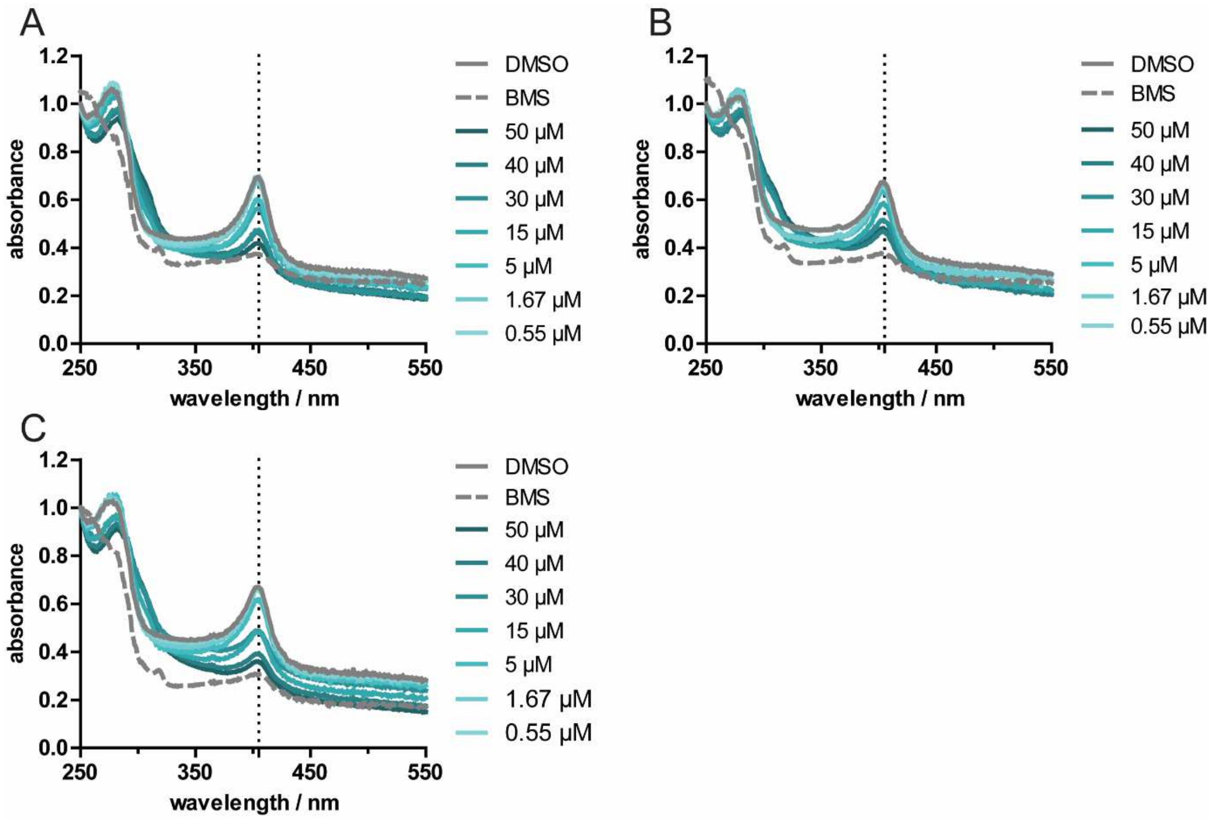

Figure S10. UV/Vis spectra of IDO1. Related to figure 5. Purified holo-IDO1 was treated with apoxidole-1, BMS-986205 (20 µM) or DMSO for 2 h at 37°C prior to detecting UV/Vis spectra (n = 3). Complete spectra of all three biological replicates are shown in A)-C). Dotted lines indicate the Soret peak at 404 nm. BMS: BMS-986205.

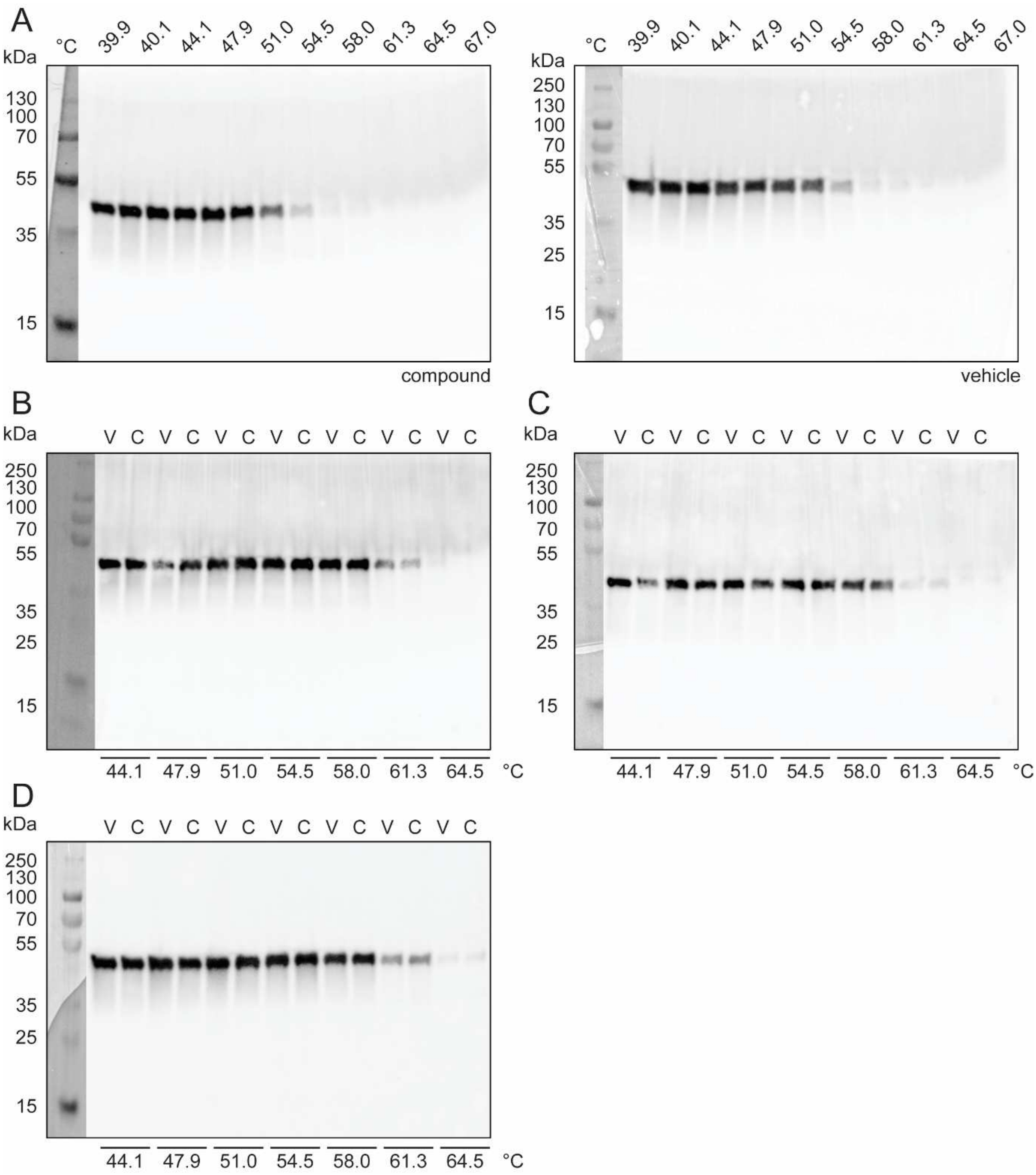

Figure S11. Cellular thermal shift assay (CETSA) for IDO1 in SKOV-3 cells. Related to Figure 4. Cells were treated with IFN-γ for 24 h prior to addition of 50 µM apoxidole-1 or DMSO for 15 min. Uncropped immunoblots of all four biological replicates shown. V: vehicle. C: compound.

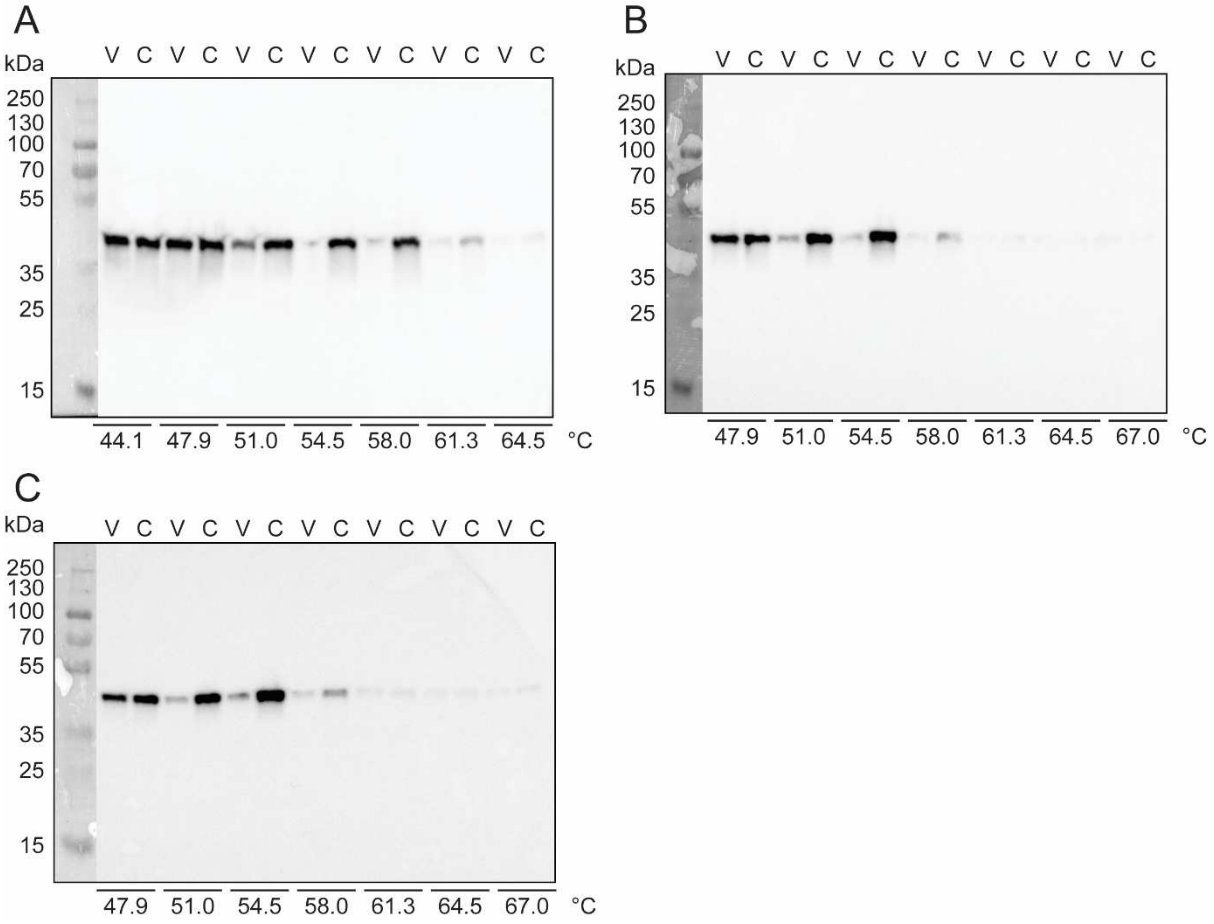

Figure S12. Cellular thermal shift assay (CETSA) for apo-IDO1 in SKOV-3 cells. Related to figure 4. Cells were treated with IFN-γ and 10 µM succinylacetone (heme synthesis inhibitor) for 24 h prior to addition of 30 µM apoxidole-1 or DMSO for 15 min. Uncropped immunoblots of all three biological replicates shown. V: vehicle. C: compound.

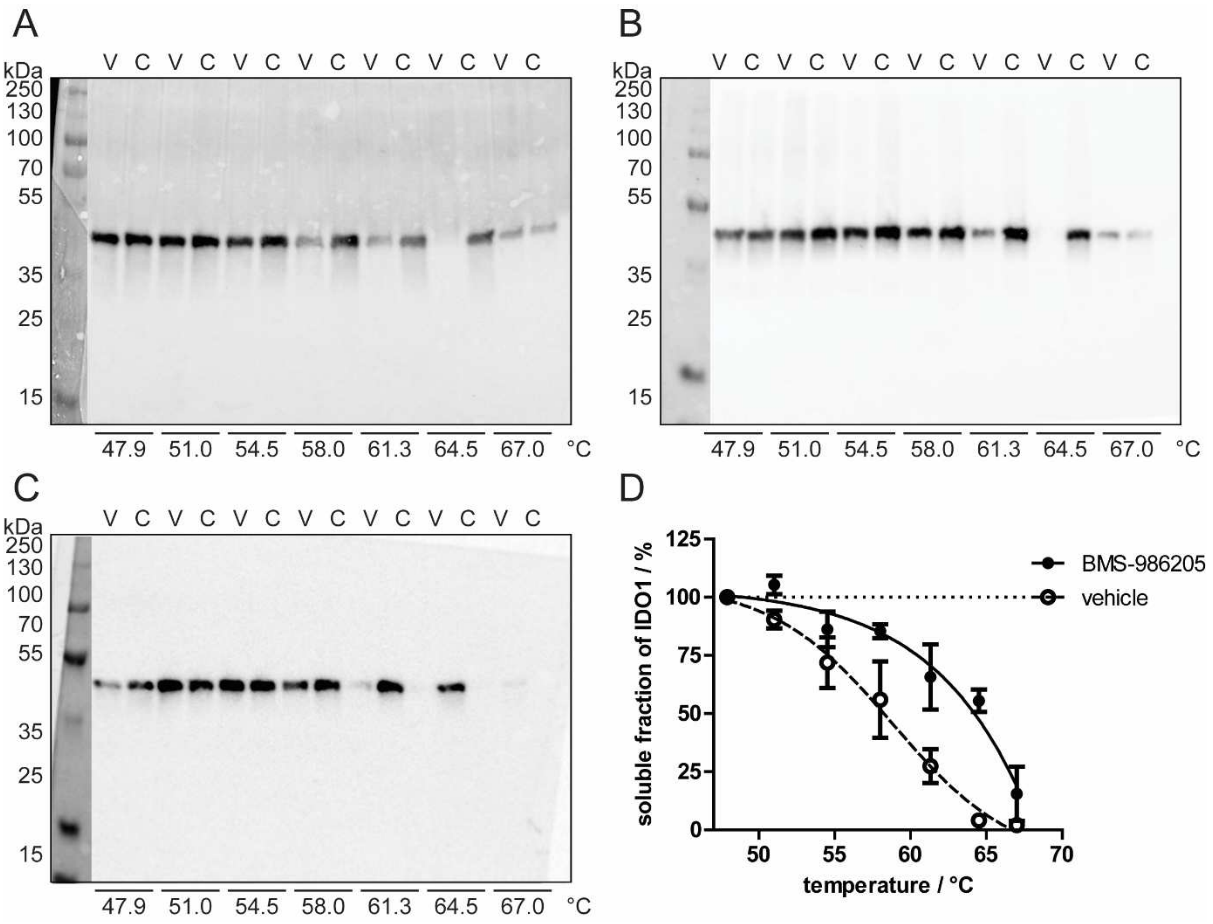

Figure S13. Cellular thermal shift assay (CETSA) for IDO1 in SKOV-3 cells. A-C) Cells were treated with IFN-γ for 24 h prior to addition of 20 µM BMS-986205 or DMSO for 15 min. Uncropped immunoblots of all three biological replicates are shown. D) Thermal profiles of IDO1 upon compound treatment. Quantification of IDO1 band intensities from A, B and C shown in D (mean ± SD, n = 3). V: vehicle. C: compound.

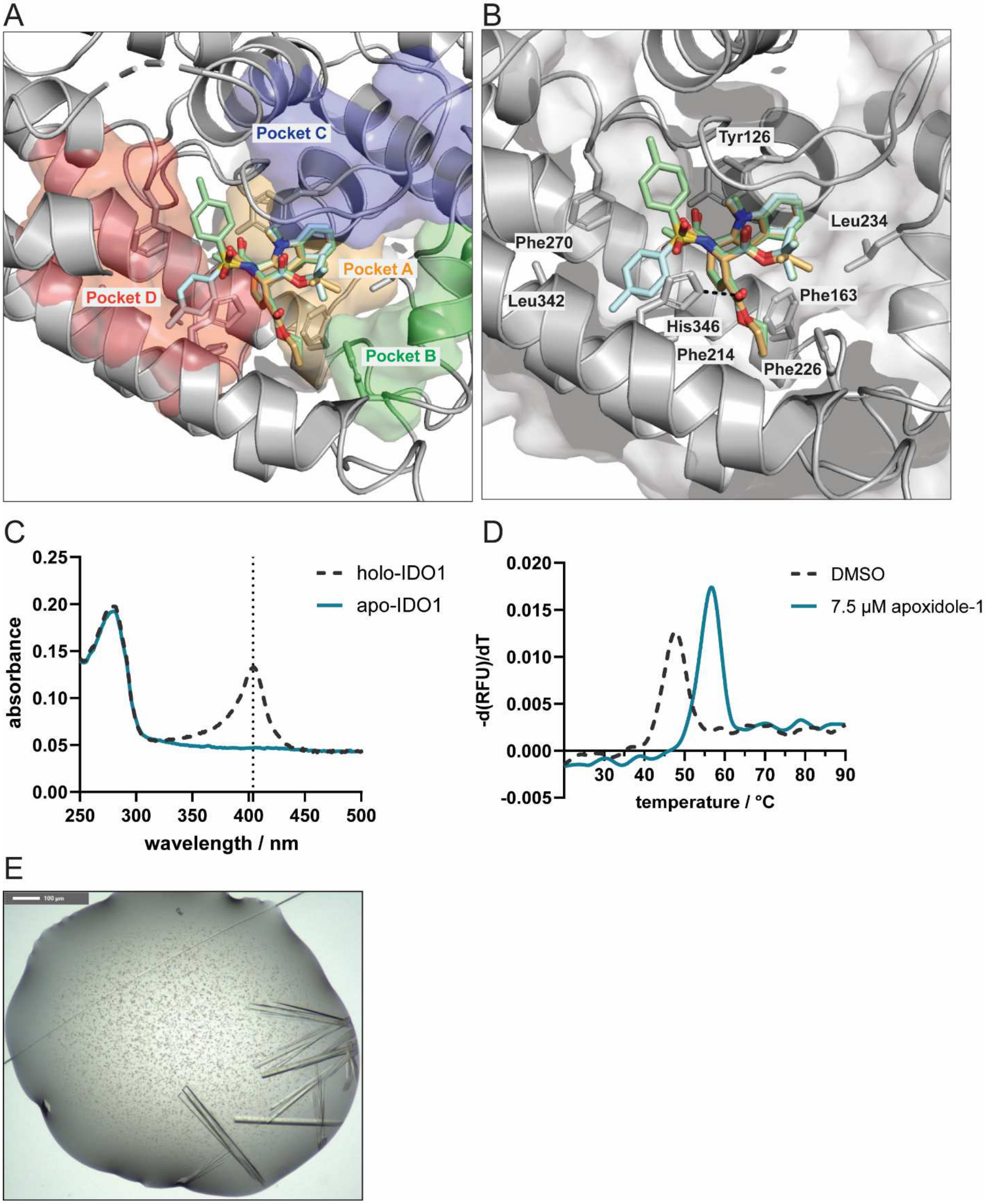

Figure S14. Purification and crystallisation of apo-IDO1 with its bound ligand apoxidole-1 (pdb 8abx). A) Apoxidole-1 occupies pockets A and D of apo-IDO1. The IDO1 active site consists of four sub-pockets: Pockets A (orange), B (green), C (blue) and D (red). Apoxidole-1 displaces the heme cofactor and binds to

pockets A and D. B) Secondary structure elements stabilizing apoxidole-1 in the IDO1 active site. Apoxidole-1 binds to the hydrophobic pocket of apo-IDO1 (gray cartoons) in different conformations (green, cyan and orange sticks). Besides the two confirmations with the flexible tosyl group (green and cyan sticks), we modelled a third confirmation with the tosyl group removed (orange sticks). This explains the significantly weaker density at the otherwise electron-rich sulfonamide. We presumed that radiation damage may lead to hydrolysis at the sulfonamide. HPLC-ESI-MS was utilized to determine the ligand integrity 63 days after the crystallization setup and showed the ligand is still intact, while the ligand without the tosyl was not identified (Figure S12). The amino acids in the active site are labeled with the three-letter code. The dotted black line indicates a hydrogen bond between His346 and the carbonyl oxygen of the ethyl ester of apoxidole-1. Heteroatoms of the ligand are depicted in red (oxygen), blue (nitrogen) and yellow (sulfur). Amino acids 383-389 are omitted for clarity. C). The heme occupancy of purified rhIDO1 was determined by UV/Vis spectrophotometry. The dotted line indicates the Soret peak at 404 nm. D) Purified apo-IDO1 was incubated with apoxidole-1 at 20°C for 15 min prior to detection of the intrinsic tryptophan/tyrosine fluorescence upon thermal denaturation. Representative melting curves are shown (n = 3). E) Purified apo-IDO1 was incubated with apoxidole-1 at 42°C for 2 h prior to setting up a crystallization experiment in a sitting drop setup. Protein crystals were obtained from mixing the protein solution with the reservoir solution (40% (v/v) PEG200 in 100 mM MES, pH 6.5) in a 1:1 ratio and incubation at 20°C.

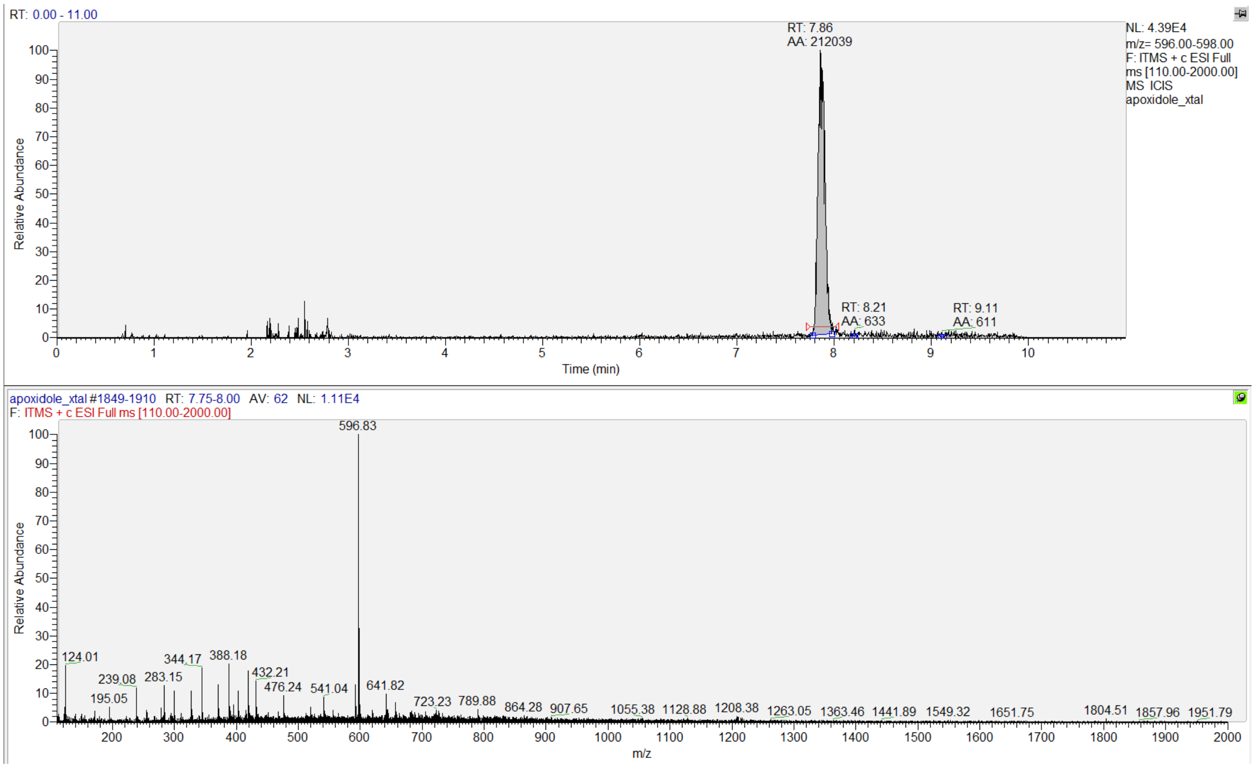

Figure S15. Integrity of apoxidole-1 in the crystallization setup. 63 days after the crystallization setup, the integrity of apoxidole-1 in the protein solution was confirmed by mixing the crystallization drop with 100 µL of DCM prior to recording HPLC-ESI-MS spectra.

HPLC method

time [min]	%A	%B
0	90	10
0.5	90	10
7.5	5	95
9.0	5	95
11	90	10

A: ddH_2O + 0.1% (v/v) formic acid; B: acetonitrile + 0.1% (v/v) formic acid; flow rate:0.4 mL/min.

Instruments: LTQ Fleet (Thermo Fisher Scientific, US), Ultimate 3000 HPLC (Thermo Fisher Scientific, US).

A

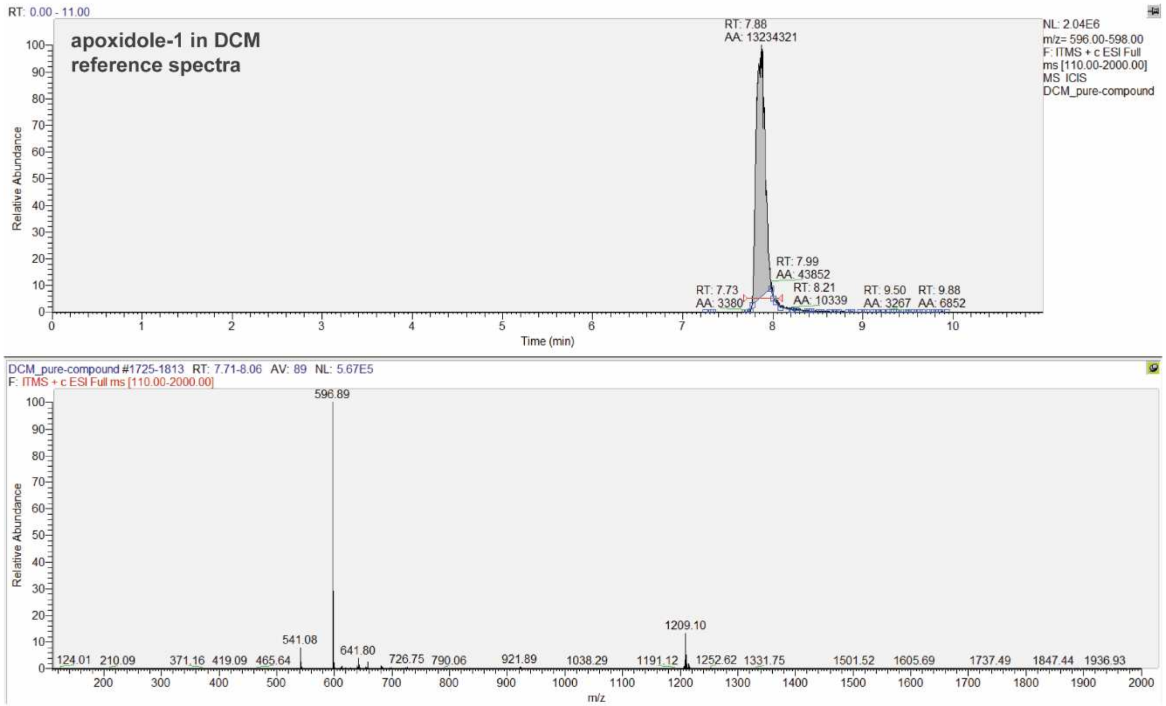
RT: 0.00 - 11.00
apoxidole-1 in DCM
reference spectra
RT: 7.88
AA: 13234321
NL: 2.04E6
m/z= 596.00-598.00
F: ITMS + c ESI Full
ms [110.00-2000.00]
MS ICIS
DCM_pure-compound
RT: 7.73
AA: 3380
RT: 7.99
AA: 43852
RT: 8.21
AA: 10339
RT: 9.50
AA: 3267
RT: 9.88
AA: 6852
Relative Abundance
Time (min)
DCM_pure-compound #1725-1813 RT: 7.71-8.06 AV: 89 NL: 5.67E5
F: ITMS + c ESI Full ms [110.00-2000.00]
596.89
541.08
641.80
1209.10
124.01 210.09 371.16 419.09 465.64 726.75 790.06 921.89 1038.29 1191.12 1252.62 1331.75 1501.52 1605.69 1737.49 1847.44 1936.93
m/z

B

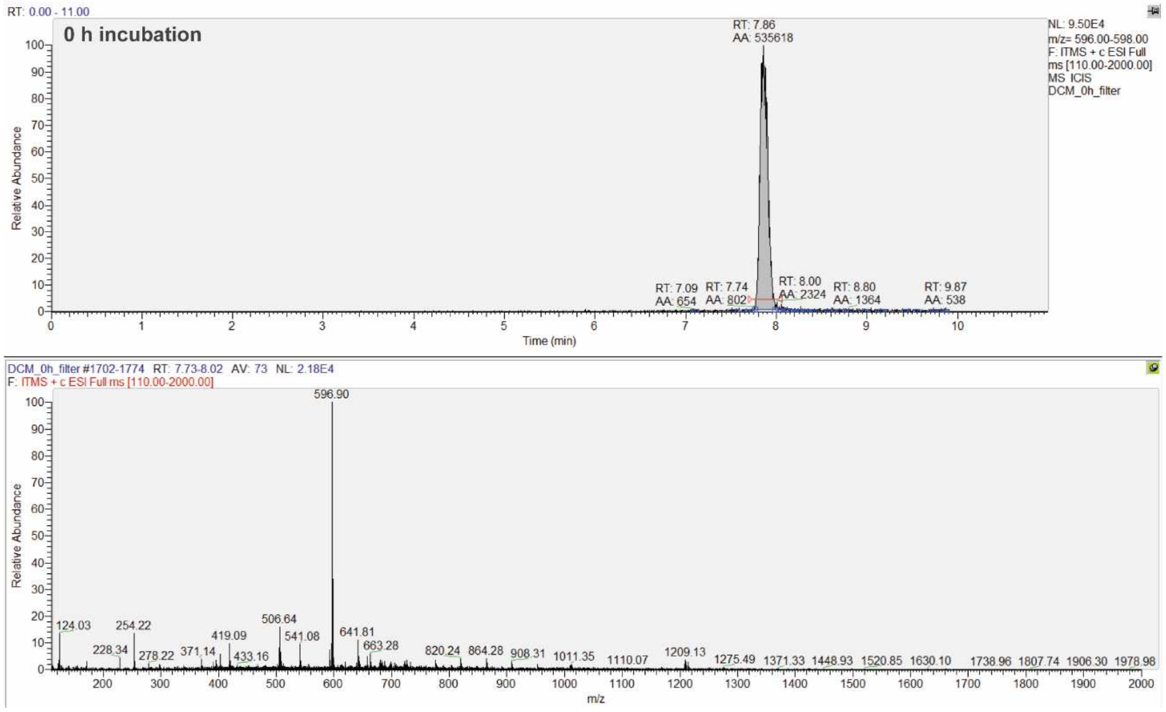
RT: 0.00 - 11.00
0 h incubation
RT: 7.86
AA: 535618
NL: 9.50E4
m/z= 596.00-598.00
F: ITMS + c ESI Full
ms [110.00-2000.00]
MS ICIS
DCM_0h_filter
RT: 7.09
AA: 654
RT: 7.74
AA: 802
RT: 8.00
AA: 2324
RT: 8.80
AA: 1364
RT: 9.87
AA: 538
Relative Abundance
Time (min)
DCM_0h_filter #1702-1774 RT: 7.73-8.02 AV: 73 NL: 2.18E4
F: ITMS + c ESI Full ms [110.00-2000.00]
596.90
124.03 254.22 228.34 278.22 371.14 419.09 433.16 506.64 541.08 641.81 663.28 820.24 864.28 908.31 1011.35 1110.07 1209.13 1275.49 1371.33 1448.93 1520.85 1630.10 1738.96 1807.74 1906.30 1978.98
m/z

C

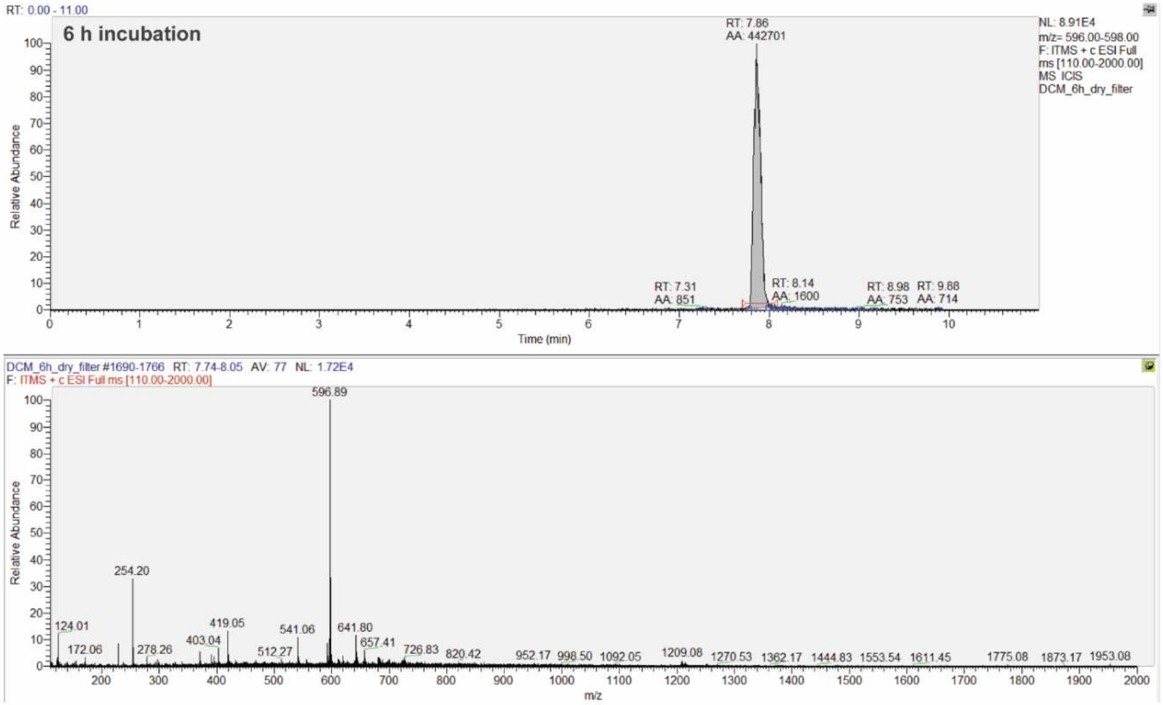

RT: 0.00 - 11.00
6 h incubation
RT: 7.86
AA: 442701
NL: 8.91E4
m/z= 596.00-598.00
F: ITMS + c ESI Full ms [110.00-2000.00] MS ICIS
DCM_6h_dry_filter
RT: 7.31
AA: 851
RT: 8.14
AA: 1600
RT: 8.98
AA: 753
RT: 9.88
AA: 714
Relative Abundance
Time (min)
DCM_6h_dry_filter #1690-1766 RT: 7.74-8.05 AV: 77 NL: 1.72E4
F: ITMS + c ESI Full ms [110.00-2000.00]
596.89
254.20
124.01
172.06
278.26
403.04
419.05
512.27
541.06
641.80
657.41
726.83
820.42
952.17
998.50
1092.05
1209.08
1270.53
1362.17
1444.83
1553.54
1611.45
1775.08
1873.17
1953.08
m/z

D

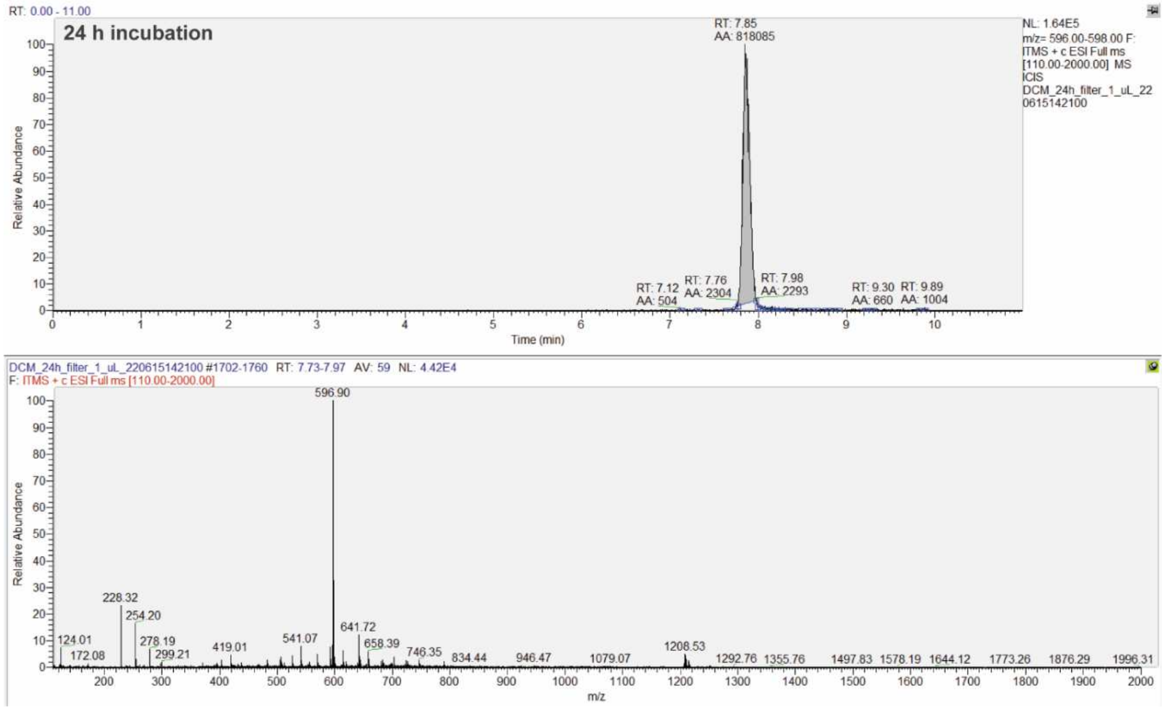

RT: 0.00 - 11.00
24 h incubation
RT: 7.85
AA: 818085
NL: 1.64E5
m/z= 596.00-598.00 F: ITMS + c ESI Full ms [110.00-2000.00] MS ICIS
DCM_24h_filter_1_uL_220615142100
RT: 7.12
AA: 504
RT: 7.76
AA: 2304
RT: 7.98
AA: 2293
RT: 9.30
AA: 660
RT: 9.89
AA: 1004
Relative Abundance
Time (min)
DCM_24h_filter_1_uL_220615142100 #1702-1760 RT: 7.73-7.97 AV: 59 NL: 4.42E4
F: ITMS + c ESI Full ms [110.00-2000.00]
596.90
228.32
254.20
124.01
172.08
278.19
299.21
419.01
541.07
641.72
658.39
746.35
834.44
946.47
1079.07
1208.53
1292.76
1355.76
1497.83
1578.19
1644.12
1773.26
1876.29
1996.31
m/z

E

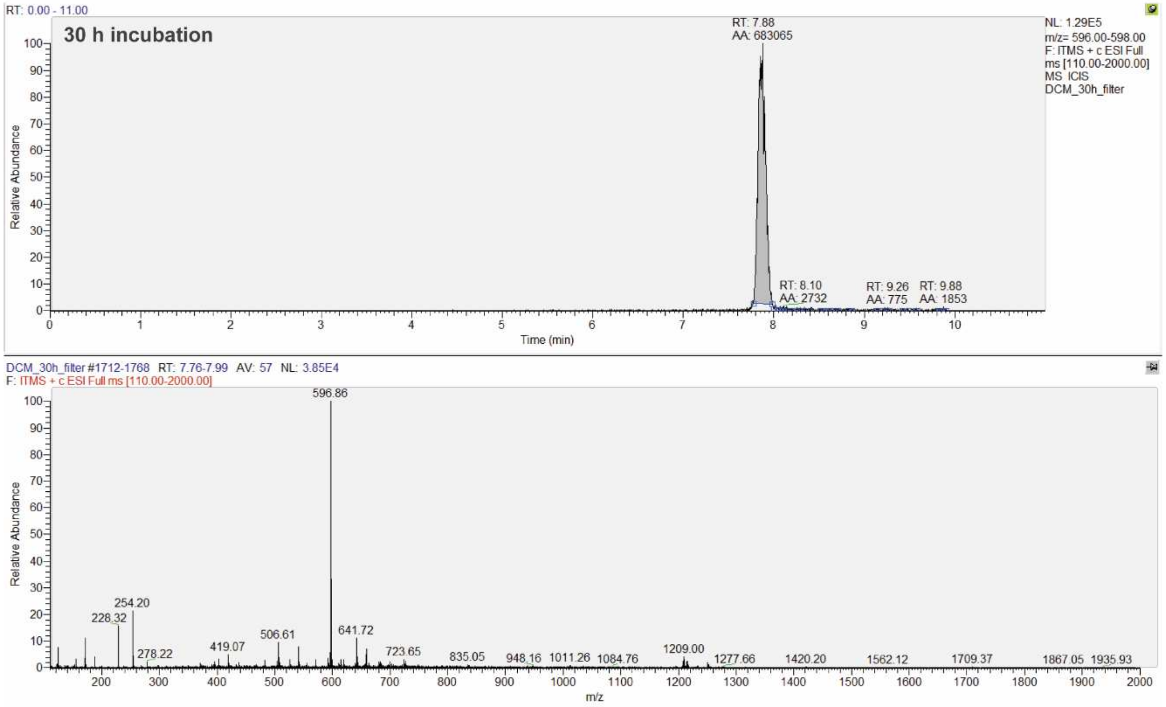

F

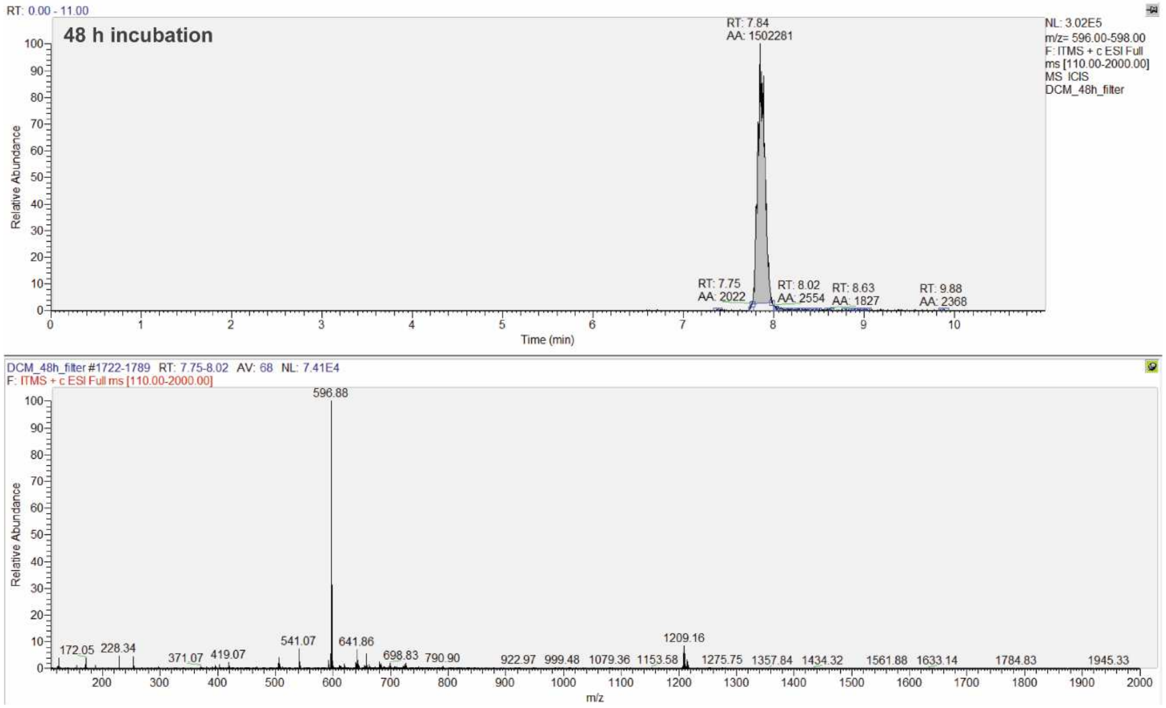

Figure S16. Stability of apoxidole-1 in serum. A) Reference HPLC-ESI-MS spectra of apoxidole-1 in DCM. B)-F) 400 µM Apoxidole-1 was incubated in fetal bovine serum (FBS) for up to 48 h at 37°C prior to extraction of the compound with DCM. The organic phase was dried and filtered, then subjected to HPLC-ESI-MS analysis. See biological methods for experimental details.

HPLC method

time [min]	%A	%B
0	90	10
0.5	90	10
7.5	5	95
9.0	5	95
11	90	10

A: ddH_2O + 0.1% (v/v) formic acid; B: acetonitrile + 0.1% (v/v) formic acid; flow rate:0.4 mL/min.

Instruments: LTQ Fleet (Thermo Fisher Scientific, US), Ultimate 3000 HPLC (Thermo Fisher Scientific, US).

Table S1. Activity of the pseudo-NP apoxidole (*S*,*R*)-3b in a selection of different automated screening assays.

	**inhibition**	**IC_{50}/EC_{50} [μM]**
kynurenine levels[2]	94.0 ± 1%	0.63 ± 0.32%
autophagy induction under fed condition[3]	0.33 ± 0.47%	inactive
autophagy inhibition in the presence of rapamycin[3]	10.50 ± 9.50%	
autophagy induction in the presence of chloroquine[3]	-3.67 ± 0.94%	inactive
hedgehog signaling[4]	73.0 ± 10.0%	6.33 ± 0.69
alkaline phosphatase[4b, 5]		inactive
BMP signaling[5]	37%	
Wnt signaling[6]	45.33 ± 1.89%	
glucose uptake[7]	17%	

2. Chemical Synthesis Methods

2.1. General Information

Unless otherwise noted, all commercially available compounds were used as provided without further purification. Solvents for chromatography were technical grade.

Analytical thin-layer chromatography (TLC) was performed on Merck silica gel aluminium plates with F-254 indicator. Compounds were visualized by irradiation with UV light or potassium permanganate staining. Column chromatography was performed using silica gel Merck 60 (particle size 0.040-0.063 mm).

^{1}H-NMR, ^{13}C-NMR and ^{19}F-NMR were recorded on a Bruker DRX400 (400 MHz), Bruker DRX500 (500 MHz), INOVA500 (500 MHz) and Bruker DRX700 using $CDCl_3$ or CD_2Cl_2 as solvent. Data are reported in the following order: chemical shift (δ) values are reported in ppm with the solvent resonance as internal standard ($CDCl_3$: δ = 7.26 ppm for ^{1}H, δ = 77.16 ppm for ^{13}C, CD_2Cl_2: δ = 5.32 ppm for ^{1}H, δ = 54.00 ppm for ^{13}C). Multiplicities are indicated br s (broad singlet), s (singlet), d (doublet), t (triplet), q (quartet), m (multiplet); coupling constants (J) are given in Hertz (Hz).

High resolution mass spectra (HR-MS) were recorded on an LTQ Orbitrap mass spectrometer coupled to an Accela HPLC-System (HPLC column: Hypersyl GOLD, 50 mm x 1 mm, particle size 1.9 µm, ionization method: electron spray ionization).

The enantiomeric excesses were determined by HPLC analysis using a chiral stationary phase column (CHIRALCEL IC, CHIRALCEL IA; eluent: (DCM/EtOH = 100/2) / *iso*-hexane or (DCM/MeOH = 100/5) / *iso*-hexane (4.6 mm x 250 mm, particle size 5 µm). The chiral HPLC methods were calibrated with the corresponding racemic mixtures.

2.2 Reaction Optimization

Screening of reaction conditions. Initial screening conditions were reported b Kwon *et al.*[8]

2.2.1 *N*-Substituted Indole Racemic Reaction Screen

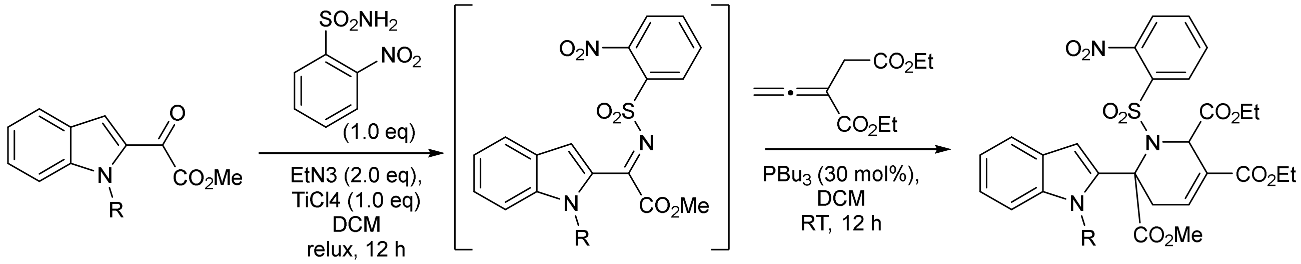

Entry	R	Ketimine	Isolated Yield [%]
**1**	All	Isolated	51
**2**	**Me**	**Isolated**	**63**
**3**	PMB	Not observed	-
**4**	SO_2Ph	Not observed	-
**5**	H	90% SM recovered	-
**6**	Boc	No reaction	-
**7**	Bn	90% SM recovered	-
**8**	MOM	90% SM recovered	-

Reactions initially performed with ketimine formation *in-situ*.

2.2.2 Chiral Phosphine Catalyst Screen

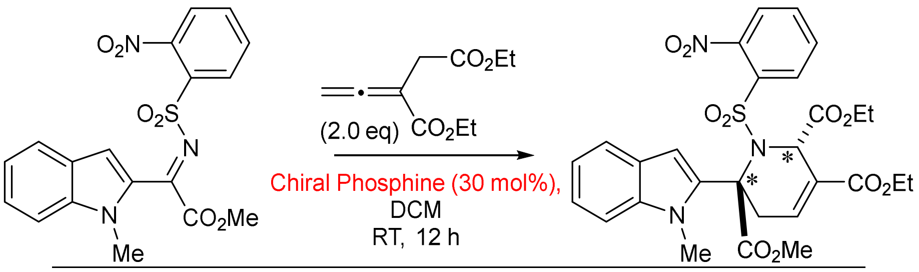

Entry	Catalyst (30 mol%)	Isolated Yield [%]	ee [%]
**1**	*(S,S)*-Ph-BPE	20	11
**2**	***(S,S)*-Et-BPE**	**96**	**74**
**3**	*(S,S)*-Me-BPE	81	54
**4**	*(R,R)*-Et-BPE	50	77
**5**	*(S,S)*-iPr-BPE	5	40
**6**	*(S)*-SITCP	42	40
**7**	*(R,R)*-Me-DUPHOS	26	18
**8**	**C1**[a]	23	80
**9**	**C2**	10	5
**10**	**C3**	0	0

**11**	**C4**	0	0

[a] Catalyst synthesised using literature procedure[9].

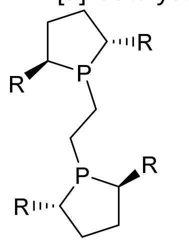

R = Me; (S,S)-Me-BPE
R = Et; (S,S)-Et-BPE
R = Ph; (S,S)-Ph-BPE
R = iPr; (S,S)-iPr-BPE

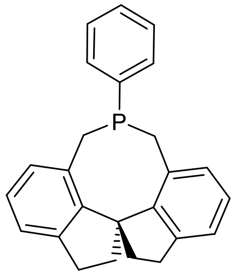

(S)-SITCP

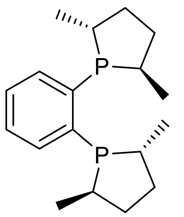

(R,R)-Me-DUPHOS

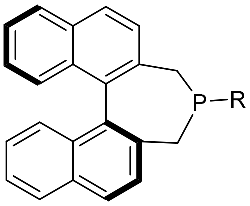

C1 R = tBu
C2 R = Ph

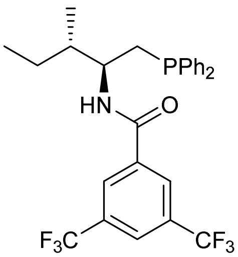

C3

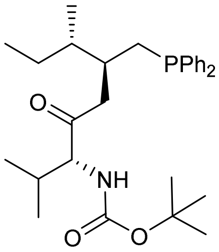

C4

2.2.3 Solvent Screen

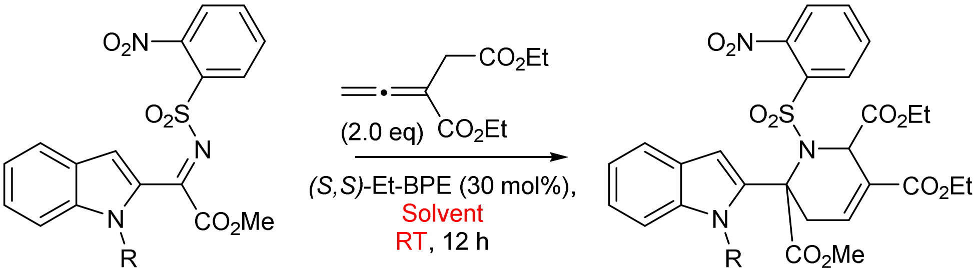

Entry	Solvent	Temperature	Isolated Yield [%]	ee [%]
**1**	Tol	RT	31	84
**2**	DCM	RT	96	74
**3**	DCE	RT	0	-
**4**	**THF**	**RT**	**71**	**88**
**5**	MeCN	RT	20	76
**6**	EtOAc	RT	53	82
**7**	DMF	RT	0	-
**8**	$CHCl_3$	RT	ND	-
**9**	Dioxane	RT	42	82
**10**	DCM	0 °C	18	79
**11**	DCM	-30 °C	7	40
**12**	Tol	0 °C	80	79
**13**	THF	0 °C	54	77

2.3 General Procedures and Analytical Data

2.3.1 General Procedure for the Synthesis of α-Ketoester

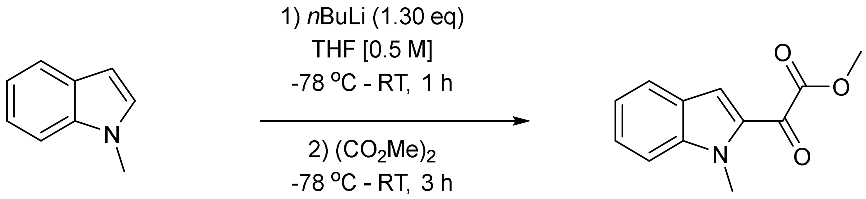

An oven-dried flask, under argon was charged with N-methyl indole (1.0 eq.) and THF (0.5M). The solution was then cooled to -78 °C and *n*BuLi (1.2 eq., 1.6 M solution in hexanes) was added dropwise. The reaction mixture was stirred for 10 min at -78°C and then 1 h at RT. The resulting solution was cooled to -78 °C then a solution of dimethyl oxalate (1.3 eq.) dissolved in THF was added. The reaction mixture was stirred for 1 h at -78°C and then for 3 h at room temperature. Then, H_2O was added and the mixture was extracted with Et_2O (5x 25 mL). The combined organic layers were then dried over $MgSO_4$, filtered and concentrated *in vacuo*. The crude material was

purified by silica gel column chromatography (Pent/EtOAc (14:1 to 12:1) to afford a yellow oil. Colourless oil, 27% yield. **^{1}H-NMR (400 MHz - $CDCl_3$):** δ 7.72 (dq, *J* = 8.2, 0.9 Hz, 1H), 7.62 (d, *J* = 0.8 Hz, 1H), 7.45 (ddt, *J* = 8.5, 6.7, 0.9 Hz, 1H), 7.39 (dt, *J* = 8.5, 1.0 Hz, 1H), 7.17 (ddt, *J* = 8.6, 7.0, 0.9 Hz, 1H), 4.10 (d, *J* = 0.6 Hz, 3H), 3.99 (d, *J* = 0.7 Hz, 3H); **^{13}C-NMR (101 MHz - $CDCl_3$):** δ 177.38, 163.40, 141.41, 131.14, 127.78, 126.06, 123.87, 121.31, 117.82, 110.45, 52.91, 32.20; **HR-MS:** calc. for $[M+H]^+$, $C_{12}H_{12}O_3N$ = 218.08117, found: 218.08081.

2.3.2 General procedure for the Synthesis of Ketimines from Precursor α-Ketoester

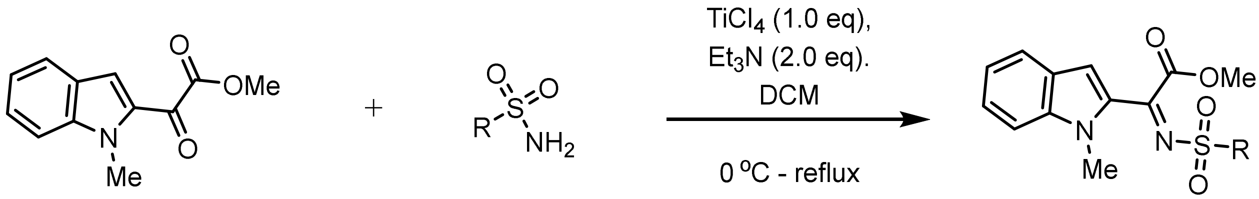

To a solution of 1,2-diketone (1.0 eq.) and sulfonamide (1.0 eq.) in DCM was added Et_3N (2.0 eq.). The solution was cooled to 0 °C and $TiCl_4$ (1.0 eq) was added. The reaction mixture was heated at reflux overnight. Then, the solution was cooled to RT, quenched with H_2O the resulting mixture was filtered through celite and washed with DCM. The filtrate was extracted with DCM (3 x 25mL) and the combined organic extracts were dried over $MgSO_4$ and concentrated under reduced pressure. The crude material was purified by silica gel column chromatography (Pent/EtOAc (4:1 to 2:1)) to afford the corresponding imino-ester.

Methyl (Z)-2-(1-methyl-1H-indol-2-yl)-2-(((2-nitrophenyl)sulfonyl)imino)acetate (1a)

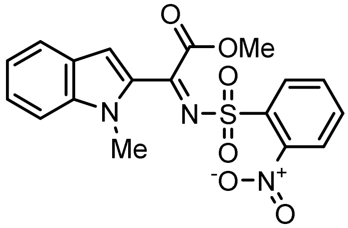

Yellow powder, 53%. **^{1}H-NMR (500 MHz – $CDCl_3$):** δ 8.28 – 8.23 (m, 1H), 7.83 – 7.73 (m, 3H), 7.65 (dt, *J* = 8.3, 1.0 Hz, 1H), 7.44 (m, *J* = 8.2, 6.9, 1.1 Hz, 1H), 7.35 (d, *J* = 8.8, 0.9 Hz, 1H), 7.31 (s, *J* = 0.8 Hz, 1H), 7.15 (ddd, *J* = 8.0, 6.9, 0.9 Hz, 1H), 4.09 (s, 3H), 4.05 (s, 3H).; **^{13}C-NMR (126 MHz – $CDCl_3$):** δ 164.16, 160.94, 148.46, 143.30, 134.51, 133.11, 132.44, 130.87, 129.57,

129.06, 126.70, 124.79, 123.68, 121.86, 120.66, 110.83, 54.08, 33.25; **HR-MS:** calc. for $[M+H]^+$, $C_{18}H_{16}O_6N_3S$ = 402.07543, found: 402.07529.

Methyl (Z)-2-(1-methyl-1H-indol-2-yl)-2-(tosylimino)acetate (1b)

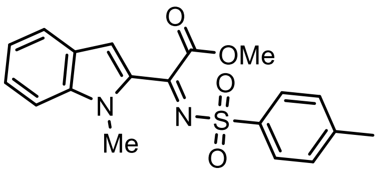

Dark yellow powder, 64%. **^{1}H-NMR (500 MHz – $CDCl_3$):** δ 7.95 – 7.89 (m, 2H), 7.64 (dt, *J* = 8.2, 1.0 Hz, 1H), 7.41 (ddd, *J* = 8.2, 6.8, 1.2 Hz, 1H), 7.38 – 7.30 (m, 3H), 7.19 (d, *J* = 0.9 Hz, 1H), 7.14 (ddd, *J* = 8.0, 6.9, 0.9 Hz, 1H), 4.11 (s, 3H), 3.99 (s, 3H), 2.44 (s, 3H); **^{13}C-NMR (126 MHz – $CDCl_3$):** δ 164.91, 159.49, 144.63, 142.65, 136.61, 129.89 (2C), 128.27, 127.89 (2C), 126.57, 123.41, 121.60, 118.84, 110.60, 53.88, 33.23, 21.83; **HR-MS:** calc. for $[M+H]^+$ $C_{19}H_{19}O_4N_2S$ = 371.10600, found: 371.10628.

Methyl (Z)-2-(1-methyl-1H-indol-2-yl)-2-((methylsulfonyl)imino)acetate (1c)

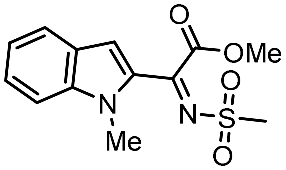

Dark yellow powder, 29%. **^{1}H-NMR (500 MHz – $CDCl_3$):** δ 7.67 (dt, *J* = 8.2, 1.0 Hz, 1H), 7.46 (ddd, *J* = 8.6, 6.8, 1.2 Hz, 1H), 7.39 (dq, *J* = 8.5, 0.9 Hz, 1H), 7.25 (d, *J* = 0.9 Hz, 1H), 7.17 (ddd, *J* = 7.9, 6.8, 1.0 Hz, 1H), 4.12 (s, 3H), 4.04 (s, 3H), 3.21 (s, 3H); **^{13}C-NMR (126 MHz – $CDCl_3$):** δ 164.45, 161.42, 143.04, 129.64, 128.77, 126.79, 123.75, 121.95, 119.76, 110.89, 54.11, 42.10, 33.51; **HR-MS:** calc. for $[M+H]^+$, $C_{13}H_{15}O_4N_2S$ = 295.07470, found: 295.07461.

Methyl (Z)-2-(1-methyl-1H-indol-2-yl)-2-((phenylsulfonyl)imino)acetate (1d)

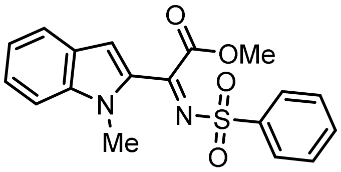

Dark yellow powder, 60%. **^{1}H-NMR (500 MHz – $CDCl_3$):** δ 8.08 – 8.02 (m, 2H), 7.62 (tt, *J* = 7.2, 1.2 Hz, 2H), 7.58 – 7.52 (m, 2H), 7.42 – 7.36 (m, 1H), 7.31 (dt, *J* = 8.6, 0.9 Hz, 1H), 7.20 (d, *J* = 0.9 Hz, 1H), 7.13 (ddd, *J* = 7.9, 6.8, 0.9 Hz, 1H), 4.11 (d, *J* = 0.8 Hz, 3H), 3.97 (s, 3H); **^{13}C-NMR**

(126 MHz – $CDCl_3$): δ 165.02, 160.03, 142.95, 139.81, 133.80, 129.46 (2C), 128.62, 128.01 (2C), 127.92, 126.78, 123.66, 121.86, 119.34, 110.78, 54.10, 33.44; **HR-MS:** calc. for $[M+H]^+$, $C_{18}H_{17}O_4N_2S$ = 357.09035, found: 357.09088.

Methyl (Z)-2-(((4-bromophenyl)sulfonyl)imino)-2-(1-methyl-1H-indol-2-yl)acetate (1e)

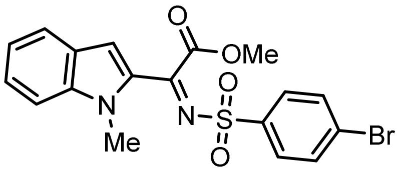

Dark yellow powder, 64%. **^{1}H-NMR (500 MHz – $CDCl_3$):** δ 7.93 – 7.87 (m, 2H), 7.70 – 7.67 (m, 2H), 7.66 – 7.63 (m, 1H), 7.44 – 7.40 (m, 1H), 7.33 (dd, *J* = 8.6, 1.0 Hz, 1H), 7.22 (d, *J* = 0.9 Hz, 1H), 7.15 (ddd, *J* = 8.0, 6.9, 0.9 Hz, 1H), 4.11 (s, 3H), 3.98 (s, 3H); **^{13}C-NMR (126 MHz – $CDCl_3$):** δ 164.76, 160.14, 142.94, 138.71, 132.60 (2C), 129.62, 129.37 (2C), 128.85, 128.71, 126.63, 123.56, 121.81, 119.67, 110.70, 54.00, 33.29; **HR-MS:** calc. for $[M+H]^+$, $C_{18}H_{16}O_4N_2BrS$ = 435.0008, found: 435.00098 and $C_{18}H_{16}O_4N_2{}^{81}BrS$ = 436.99882, found: 436.99895.

Methyl (Z)-2-(1-methyl-1H-indol-2-yl)-2-(((3-(trifluoromethyl)phenyl)sulfonyl)imino)acetate (1f)

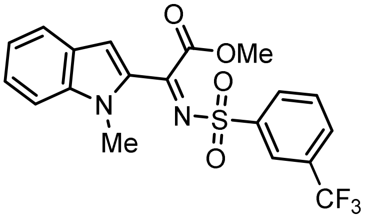

Dark yellow powder, 30%. **^{1}H-NMR (500 MHz – $CDCl_3$):** δ 8.34 – 8.31 (m, 1H), 8.24 (dtt, *J* = 7.5, 1.4, 0.7 Hz, 1H), 7.91 – 7.86 (m, 1H), 7.71 (ddt, *J* = 8.0, 7.1, 0.8 Hz, 1H), 7.65 (dq, *J* = 8.2, 1.2 Hz, 1H), 7.47 – 7.41 (m, 1H), 7.34 (dq, *J* = 8.6, 0.9 Hz, 1H), 7.26 (d, *J* = 1.0 Hz, 1H), 7.15 (ddd, *J* = 8.1, 6.9, 1.0 Hz, 1H), 4.12 (d, *J* = 1.4 Hz, 3H), 4.00 (d, *J* = 1.4 Hz, 3H); **^{13}C-NMR (151 MHz – $CDCl_3$):** δ 163.35, 159.45, 141.94, 139.83, 130.90, 130.67, 129.85, 128.97, 128.89, 128.40, 127.75, 123.81, 125.47, 122.45, 120.70, 118.96, 109.54, 52.87, 32.11; **^{19}F NMR (565 MHz, $CDCl_3$)** δ -62.83.; **HR-MS:** calc. for $[M+H]^+$, $C_{19}H_{16}O_4N_2F_3S$ = 425.07774, found: 425.07785.

Methyl (Z)-2-(((4-fluorophenyl)sulfonyl)imino)-2-(1-methyl-1H-indol-2-yl)acetate (1g)

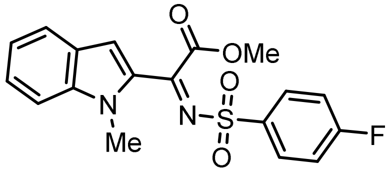

Green powder, 50%**^{1}H-NMR (500 MHz – $CDCl_3$):** δ 8.10 – 8.02 (m, 2H), 7.64 (d, *J* = 8.2 Hz, 1H), 7.42 (ddd, *J* = 8.1, 6.9, 1.1 Hz, 1H), 7.36 – 7.31 (dd, 1H), 7.24 – 7.18 (m, 3H), 7.14 (ddd, *J* = 7.9, 6.9, 0.8 Hz, 1H), 4.11 (s, 3H), 3.99 (s, 3H); **^{13}C-NMR (126 MHz – $CDCl_3$):** δ 166.67, 164.66, 159.79, 142.72, 135.56, 130.57 (d, *J* = 9.6 Hz, 2C), 129.49, 128.46, 126.47, 123.38, 121.63, 119.31, 116.46 (d, *J* = 22.7 Hz, 2C), 110.54, 53.85, 33.13; **^{19}F NMR (470 MHz, $CDCl_3$)** δ -103.74; **HR-MS:** calc. for $[M+H]^+$, $C_{18}H_{16}O_4N_2FS$ = 375.08093, found: 375.08132.

2.3.3 General Procedure for the Synthesis of Aldimines from Aldehyde

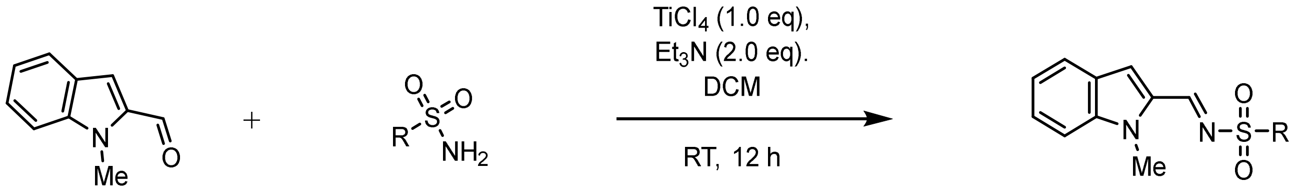

Following a modified procedure by Kwon *et al.*[8], to a suspension of N-methyl indole-2-carboxaldehyde (1.0 eq), sulfonamide (1.0 eq.), and triethylamine (2.0 eq) in DCM at 0 °C was added $TiCl_4$ (1.0 eq. After 12 h at RT, the mixture was filtered through a pad of Celite and washed with DCM. The filtrate was concentrated under reduced pressure. The crude material was purified by silica gel column chromatography (Pent/EtOAc (6:1 to 4:1)) to afford the corresponding imine.

(E)-N-((1-methyl-1H-indol-2-yl)methylene)-2-nitrobenzenesulfonamide (1h)

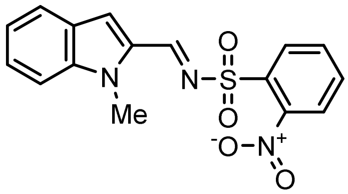

Yellow powder, 51%. **^{1}H-NMR (500 MHz – $CDCl_3$):** δ 9.01 (s, 1H), 8.40 – 8.34 (m, 1H), 7.82 – 7.74 (m, 3H), 7.72 (dt, *J* = 8.2, 1.0 Hz, 1H), 7.49 – 7.42 (m, 2H), 7.38 (dd, *J* = 8.6, 1.0 Hz, 1H), 7.18 (ddd, *J* = 8.0, 6.8, 0.9 Hz, 1H), 4.10 (s, 3H); **^{13}C-NMR (126 MHz – $CDCl_3$):** δ 163.00, 148.52, 143.01, 134.42, 132.61, 132.57, 131.66, 131.44, 128.51, 127.05, 124.79, 123.63, 121.94, 121.58, 110.70, 32.47; **HR-MS:** calc. for $[M+H]^+$, $C_{16}H_{14}O_4N_3S$ = 344.06995, found: 344.07004.

(E)-4-methyl-N-((1-methyl-1H-indol-2-yl)methylene)benzenesulfonamide (1i)

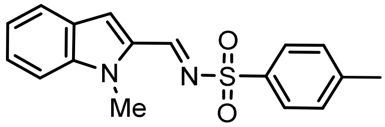

Dark yellow powder, 36%. **^{1}H-NMR (500 MHz – $CDCl_3$):** δ 8.99 (s, 1H), 7.91 – 7.86 (m, 2H), 7.69 (dt, *J* = 8.2, 1.0 Hz, 1H), 7.42 (ddd, *J* = 8.1, 6.8, 1.2 Hz, 1H), 7.37 – 7.33 (m, 3H), 7.30 (d, *J* = 0.9 Hz, 1H), 7.16 (ddd, *J* = 8.1, 6.8, 1.0 Hz, 1H), 4.08 (s, 3H), 2.44 (s, 3H); **^{13}C-NMR (126 MHz – $CDCl_3$):** δ 160.29, 144.40, 142.46, 136.06, 131.69, 129.92 (2C), 127.89 (2C), 127.83, 126.92,

123.30, 121.34, 120.37, 110.55, 32.51, 21.80; **HR-MS:** calc. for $[M+H]^+$, $C_{17}H_{17}O_2N_2S$ = 313.10053, found: 313.10057.

(E)-N-((1-methyl-1H-indol-2-yl)methylene)methanesulfonamide (1j)

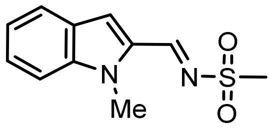

Dark yellow powder, 10%. **^{1}H-NMR (500 MHz – $CDCl_3$):** δ 8.98 (s, 1H), 7.72 (dt, *J* = 8.1 Hz, 1H), 7.45 (dd, *J* = 6.7, 1.2 Hz, 1H), 7.41 (dq, *J* = 8.6, 1.0 Hz, 1H), 7.34 (d, *J* = 1.0 Hz, 1H), 7.19 (ddd, *J* = 8.0, 6.8, 1.1 Hz, 1H), 4.15 (s, 4H), 3.14 (s, 3H).; **^{13}C-NMR (126 MHz – $CDCl_3$):** δ 161.43, 142.45, 131.25, 127.92, 126.80, 123.31, 121.34, 120.64, 110.50, 40.68, 32.39; **HR-MS:** calc. for $[M+H]^+$, $C_{11}H_{13}O_2N_2S$ = 237.06922, found: 237.06888.

2.3.4 General Procedure for the Synthesis of Allenoates

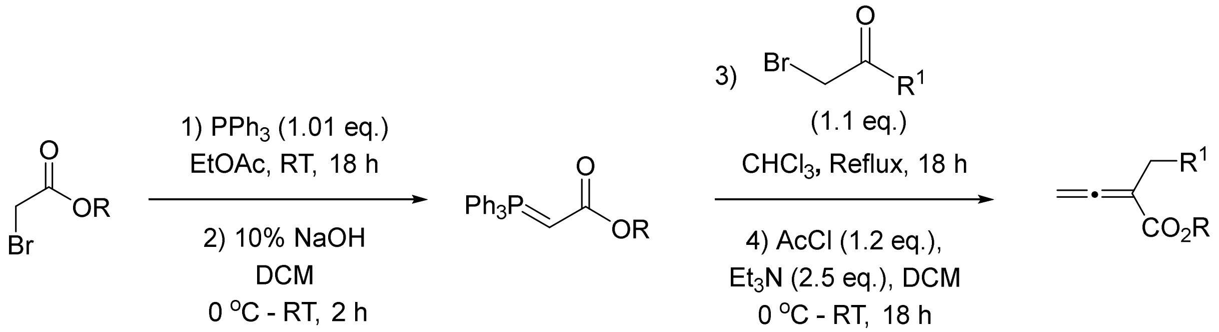

Following a modified procedure by Kwon *et al*.[8], to a stirred solution of triphenylphosphine (1.01 eq.) in EtOAc at RT, was added the corresponding bromoacetate (1.0 eq), slowly. After stirring overnight, the resulting precipitate was collected by vacuum filtration, washed with EtOAc and dried on the vacuum to provide the phosphonium salt as a white solid. The crude material was dissolved in DCM and sodium hydroxide in water (10% solution) was added at 0° C. After stirring for 2 h at room temperature the layers were allowed to separate and the aqueous phase was extracted with DCM (x3). The combined organic layers were dried over $MgSO_4$ and concentrated *in vacuo* to give the phosphorane as an off-white solid. To a solution of crude phosphorane (1.0 eq.) in $CHCl_3$ was added the corresponding bromoacetate (1.1 eq). After 18 h of reflux, the solvent was removed under reduced pressure. The crude product (1.0 eq) was dissolved in DCM and treated with Et_3N (2.5 eq). After stirring for 30 mins, the reaction was cooled 0 °C and acetyl chloride (1.2 eq.) was added dropwise. The reaction mixture was allowed to warm to RT and stirred overnight.

The solution was the concentrated to approximately 20% volume, and diluted with pentane. The triphenylphosphine oxide was removed by vacuum filtration, the filter cake was washed with 20% EtOAc/pentane. The filtrate was concentrated *in vacuo* and the resulting crude material was purified by silica gel column chromatography (Pent/EtOAc (20:1 to 15:1)) to afford the allenoate as an oil.

diethyl 2-vinylidenesuccinate (2a)

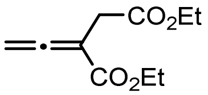

Yellow oil, 61%. **^{1}H-NMR (400 MHz – $CDCl_3$):** δ 5.16 (q, *J* = 2.1 Hz, 2H), 4.20 – 4.06 (m, 4H), 3.20 (q, *J* = 2.2 Hz, 2H), 1.21 (qd, *J* = 7.1, 2.1 Hz, 6H).; **^{13}C-NMR (126 MHz – $CDCl_3$):** δ 214.45, 170.37, 166.20, 94.58, 79.39, 61.23, 60.84, 34.72, 14.09, 14.08; **HR-MS:** calc. for $[M+H]^+$, $C_{10}H_{15}O_4$ $[M+H]^+$ 199.0970, found: 199.0967.

4-(tert-butyl) 1-ethyl 2-vinylidenesuccinate (2b)

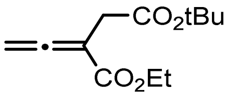

Yellow oil, 34%. **^{1}H-NMR (500 MHz – $CDCl_3$):** δ 5.20 (t, *J* = 2.2 Hz, 2H), 4.21 (q, *J* = 7.1 Hz, 2H), 3.17 (t, *J* = 2.2 Hz, 2H), 1.45 (s, 9H), 1.28 (t, *J* = 7.1 Hz, 3H); **^{13}C-NMR (126 MHz – $CDCl_3$):** δ 214.81, 170.06, 166.75, 95.36, 81.45, 79.68, 61.64, 36.23, 28.37 (3C, tBu), 14.56; **HR-MS:** calc. for $[M+H]^+$, $C_{12}H_{19}O_4$ = 227.1277, found: 227.12785.

1-(tert-butyl) 4-ethyl 2-vinylidenesuccinate (2c)

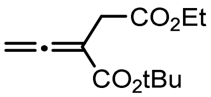

Colourless oil, 25%. **^{1}H-NMR (500 MHz – $CDCl_3$):** δ 5.16 (t, *J* = 2.2 Hz, 2H), 4.16 (q, *J* = 7.1 Hz, 2H), 3.21 (t, *J* = 2.2 Hz, 2H), 1.46 (s, 9H), 1.26 (t, *J* = 7.1 Hz, 3H); **^{13}C-NMR (126 MHz – $CDCl_3$):** δ 214.44, 170.79, 165.53, 96.11, 81.56, 79.24, 61.03, 35.13, 28.12 (3C, tBu), 14.34; **HR-MS:** calc. for $[M+H]^+$, C_{12} $H_{19}O_4$ = 227.12779, found: 227.12791.

1-benzyl 4-(tert-butyl) 2-vinylidenesuccinate (2d)

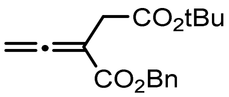

Colourless oil, 37%. **^{1}H-NMR (500 MHz – $CDCl_3$):** δ 7.38 – 7.27 (m, 5H), 5.23 (t, *J* = 2.2 Hz, 2H), 5.20 (s, 2H), 3.20 (t, *J* = 2.2 Hz, 2H), 1.42 (s, 9H); **^{13}C-NMR (126 MHz – $CDCl_3$):** δ 214.91, 169.77, 166.42, 136.08, 128.61 (2C), 128.22, 128.03 (2C), 95.01, 81.33, 79.70, 66.93, 36.00, 28.14 (3C); **HR-MS:** calc. for $[M+H]^+$, $C_{17}H_{21}O_4$ = 289.14344, found: 289.14363.

4-benzyl 1-ethyl 2-vinylidenesuccinate (2e)

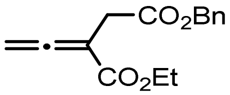

Yellow oil, 20%. **^{1}H-NMR (500 MHz – $CDCl_3$):** δ 7.39 – 7.26 (m, 5H), 5.21 (t, *J* = 2.2 Hz, 2H), 5.15 (s, 2H), 4.19 (q, *J* = 7.2 Hz, 2H), 3.33 (t, *J* = 2.2 Hz, 2H), 1.25 (t, *J* = 7.1 Hz, 3H); **^{13}C-NMR (126 MHz – $CDCl_3$):** δ 214.67, 170.51, 166.42, 135.87, 128.67 (2C), 128.62, 128.40 (2C), 94.66, 79.81, 66.89, 61.57, 34.94, 14.31; **HR-MS:** calc. for $[M+H]^+$, $C_{15}H_{17}O_4$ = 261.11214, found: 261.11229.

Di-tert-butyl 2-vinylidenesuccinate (2f)

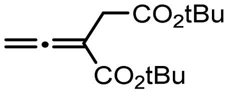

Orange oil, 30%. **^{1}H-NMR (500 MHz – $CDCl_3$):** δ 5.15 (t, *J* = 2.3 Hz, 2H), 3.12 (t, *J* = 2.3 Hz, 2H), 1.47 (s, 9H), 1.45 (s, 9H); **^{13}C-NMR (126 MHz – $CDCl_3$):** δ 214.43, 169.96, 165.62, 96.51, 81.38, 81.07, 79.05, 36.20, 28.18 (3C, tBu), 28.14 (3C, tBu); **HR-MS:** calc. for $[M+H]^+$, $C_{14}H_{23}O_4$ = 255.15909, found: 255.15921.

4-benzyl 1-(tert-butyl) 2-vinylidenesuccinate (2g)

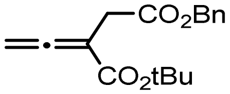

Colourless oil, 37%. **^{1}H-NMR (500 MHz – $CDCl_3$):** δ 7.39 – 7.29 (m, 5H), 5.16 – 5.14 (m, 4H, overlapped triplet and singlet), 3.28 (t, *J* = 2.2 Hz, 2H), 1.44 (s, 9H); **^{13}C-NMR (126 MHz – $CDCl_3$):** δ 214.48, 170.65, 165.47, 135.91, 128.66 (2C), 128.43 (2C), 128.37, 95.96, 81.62, 79.36, 66.82, 35.10, 28.10 (3C, tBu); **HR-MS:** calc. for $[M+H]^+$, $C_{17}H_{21}O_4$ = 289.14344, found: 289.14364.

tert-butyl 2-(cyanomethyl)buta-2,3-dienoate (2h)

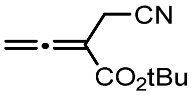

Orange oil, 15%. **^{1}H-NMR (500 MHz – $CDCl_3$):** δ 5.37 (t, *J* = 3.1 Hz, 2H), 3.29 (t, *J* = 3.1 Hz, 2H), 1.48 (s, 9H); **^{13}C-NMR (126 MHz – $CDCl_3$):** δ 213.01, 163.95, 116.82, 93.73, 82.55, 82.01, 27.98 (3C, tBu), 18.25; **HR-MS:** calc. for $[M+H]^+$, $C_{10}H_{14}O_2N$ = 180.10191, found: 180.10183.

2.3.5. General Procedure for the Synthesis of Apoxidoles

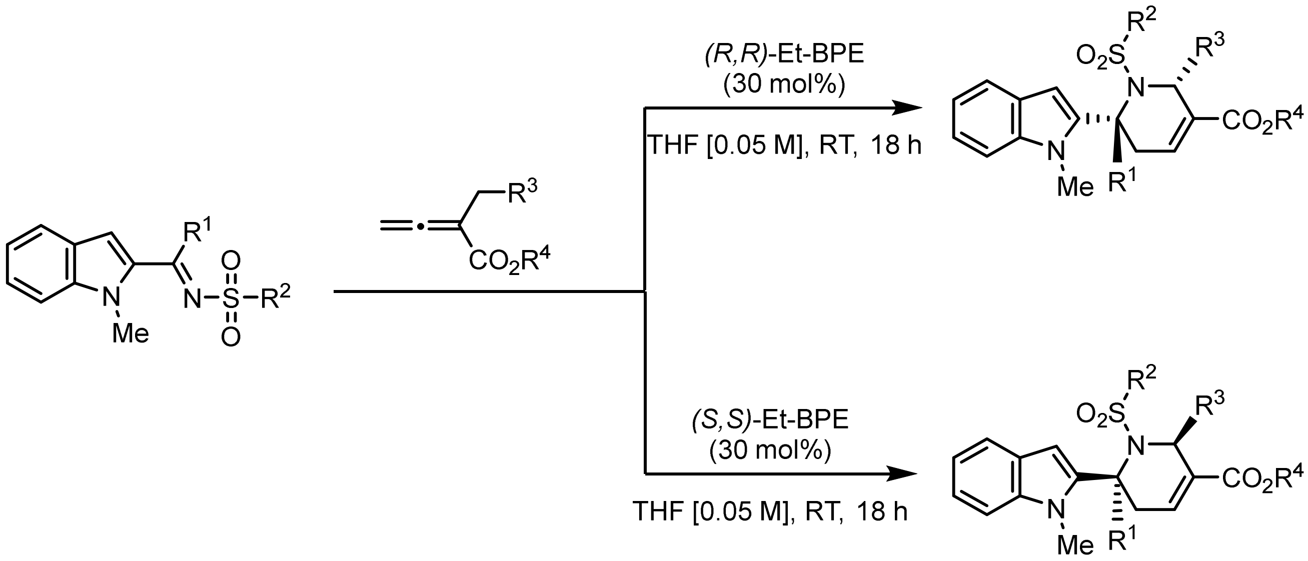

To a stirred solution of imino-ester (1.0 eq.) and allenoate (1.2 eq.) in THF [0.05M] was added the relevant chiral phosphine catalyst, Et-BPE (30 mol%). After 12 h, the reaction mixture was concentrated and purified by silica gel column chromatography (Pent/EtOAc) to afford the Apoxidole product.

2,3-diethyl 6-methyl (2R,6S)-6-(1-methyl-1H-indol-2-yl)-1-((2-nitrophenyl)sulfonyl)-1,2,5,6-tetrahydropyridine-2,3,6-tricarboxylate (3a)

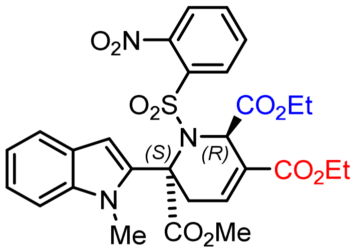

***(R,S)*-3a**

White solid, 71% yield. Pent/EtOAc (7:1 to 4:1) **^{1}H-NMR (600 MHz - CDCl$_3$):** δ 8.88 (bs, 1H), 7.79 (t, *J* = 7.6 Hz, 1H), 7.73 (td, *J* = 7.8, 1.2 Hz, 1H), 7.66 (dd, *J* = 7.9, 1.2 Hz, 1H), 7.52 (d, *J* = 7.9 Hz, 1H), 7.31 (t, *J* = 4.0 Hz, 1H), 7.25 – 7.16 (m, 2H), 7.09 – 7.04 (m, 1H), 6.48 (s, 1H), 6.04 (s, 1H), 4.24 – 4.13 (m, 2H), 3.62 (s, 3H), 3.60 - 3.51 (m, 4H), 3.39 (bs, 1H), 2.43 (bs, 2H), 1.26 (t, *J* = 1.1 Hz, 3H), 0.55 – 0.45 (m, 3H); **^{13}C-NMR (151 MHz - CDCl$_3$):** δ 168.62, 167.85, 164.47, 148.10, 139.49, 136.95, 135.74, 133.62, 133.49, 131.61, 131.57, 130.68, 130.21, 126.14, 123.70,

123.59, 121.32, 121.01, 120.39, 109.78, 107.70, 61.98, 61.31, 57.57, 53.79, 34.29, 32.87, 13.20.; **HR-MS:** calc. for $[M+H]^+$ $C_{28}H_{29}O_{10}N_3S$ = 600.1574, found: 600.1646. $[\alpha]^{RT}_{D}$ = + 59.0° (CH_2Cl_2, c = 1.00); HPLC conditions: CHIRAPAK IC column, DCM:EtOH (100:2)/*iso*-hexane = 40/60, flow rate = 0.5 mL min^{-1}, minor enantiomer: t_R= 15.85 min; major enantiomer: t_R= 24.42 min, 88% *ee*.

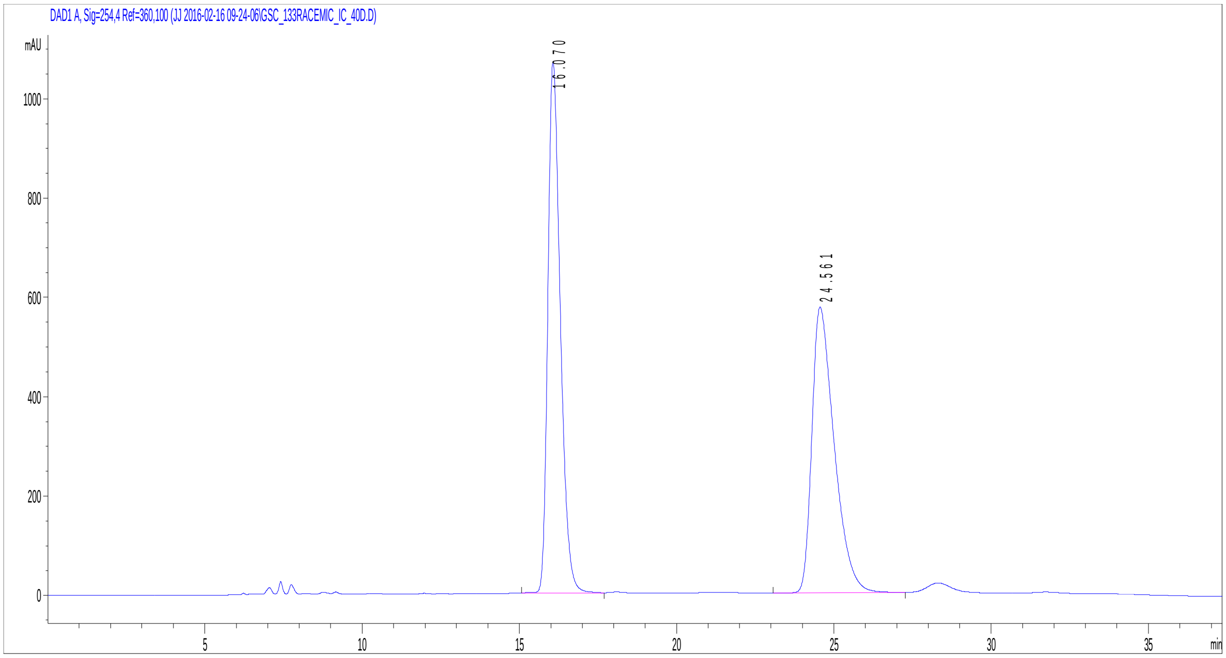

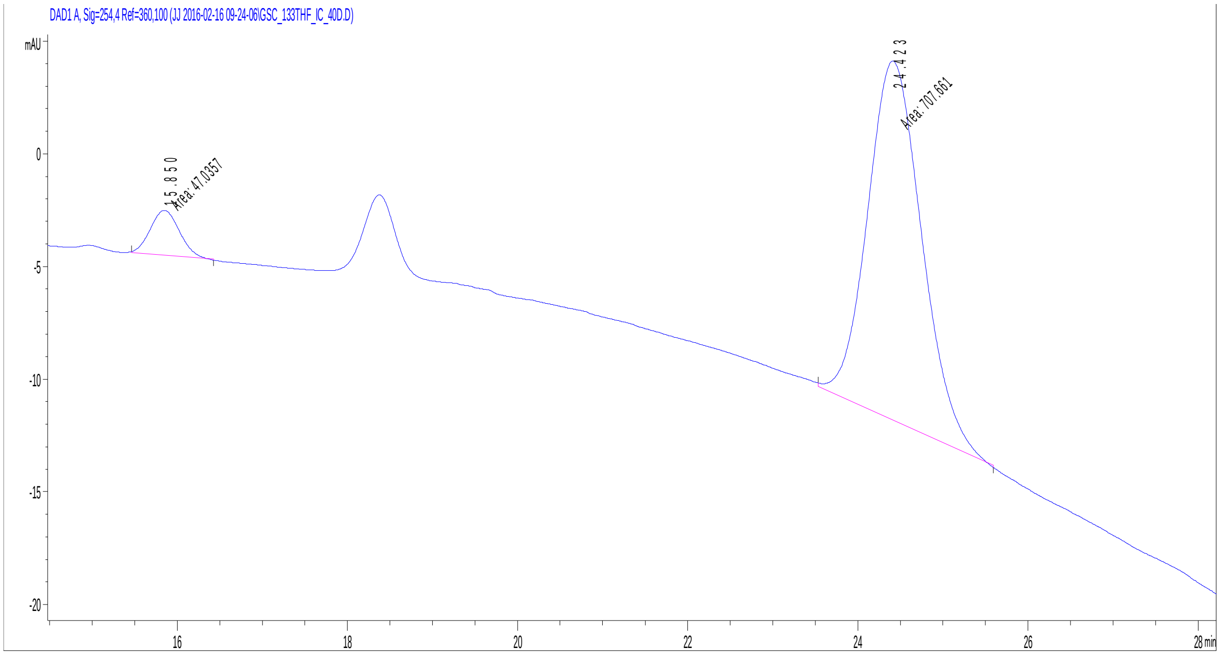

#	Time	Area	Height	Width	Area%	Symmetry
1	15.85	47	2	0.3943	6.232	0.885
2	24.423	707.7	15.9	0.7402	93.768	0.842

2-(tert-butyl) 3-ethyl 6-methyl (2R,6S)-6-(1-methyl-1H-indol-2-yl)-1-((2-nitrophenyl)sulfonyl)-1,2,5,6-tetrahydropyridine-2,3,6-tricarboxylate (3b)

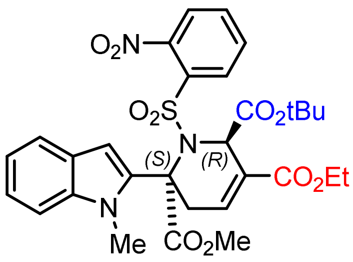

***(R,S)*-3b**

Off-white solid, 85% yield. Pent/EtOAc (5:1 to 4:1) **^{1}H-NMR (500 MHz - $CDCl_3$):** δ 8.88 (bs, 1H), 7.79 (t, *J* = 7.8 Hz, 1H), 7.71 (td, *J* = 7.6, 1.3 Hz, 1H), 7.64 (dd, *J* = 7.9, 1.4 Hz, 1H), 7.53 (dt, *J* = 7.9, 1.0 Hz, 1H), 7.24 – 7.15 (m, 3H), 7.09 – 7.04 (m, 1H), 6.50 (s, 1H), 6.05 (q, 1H), 4.32 – 4.22 (m, 1H), 4.21 – 4.11 (m, 1H), 3.59 (d, *J* = 5.5 Hz, 6H), 3.56 – 3.44 (m, 2H), 1.33 (t, *J* = 7.2 Hz, 3H), 0.72 (bs, 9H).; **^{13}C-NMR (126 MHz - $CDCl_3$):** δ 168.58, 166.83, 164.78, 147.83, 139.62, 135.79, 135.27, 133.11, 131.44, 130.10, 126.06, 123.30, 123.25, 120.96, 120.12, 110.12, 107.15, 107.13, 83.02, 66.34, 61.13, 58.04, 53.52, 34.04, 32.86, 27.04 (3C, tBu), 23.84, 14.29; **HR-MS:** calc. for $[M+H]^+$ $C_{30}H_{34}O_{10}N_3S$ = 628.1959, found: 628.1957

$[\alpha]^{RT}_{D}$ = + 87.0° (CH_2Cl_2, c = 1.00); HPLC conditions: CHIRAPAK IC column, DCM:EtOH (100:2)/*iso*-hexane = 40/60, flow rate = 0.5 mL min^{-1}, minor enantiomer: t_R= 24.24 min; major enantiomer: t_R= 14.99 min, 78% *ee*.

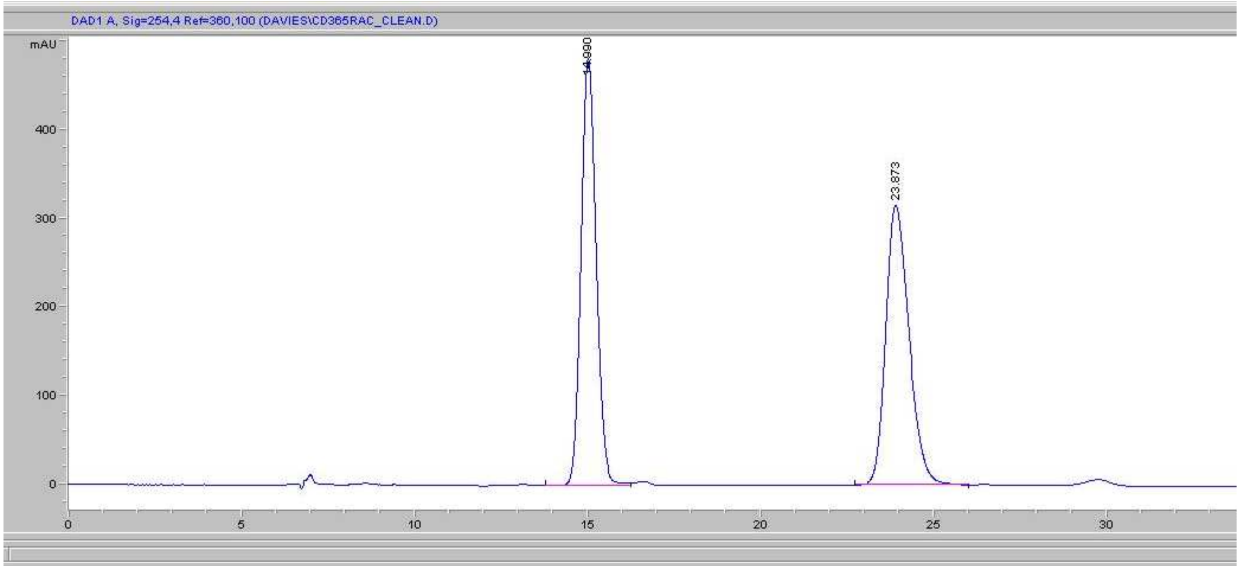

File Information	
LC-File	CD365RAC_CLEAN.D
File Path	C:\HPCHEM\1\DATA\DAVIES\
Date	26-May-21, 20:09:27

#	Time	Area	Height	Width	Area%	Symmetry
1	14.99	14391.2	481.2	0.4689	49.492	0.848
2	23.873	14686.3	315.6	0.7109	50.508	0.685

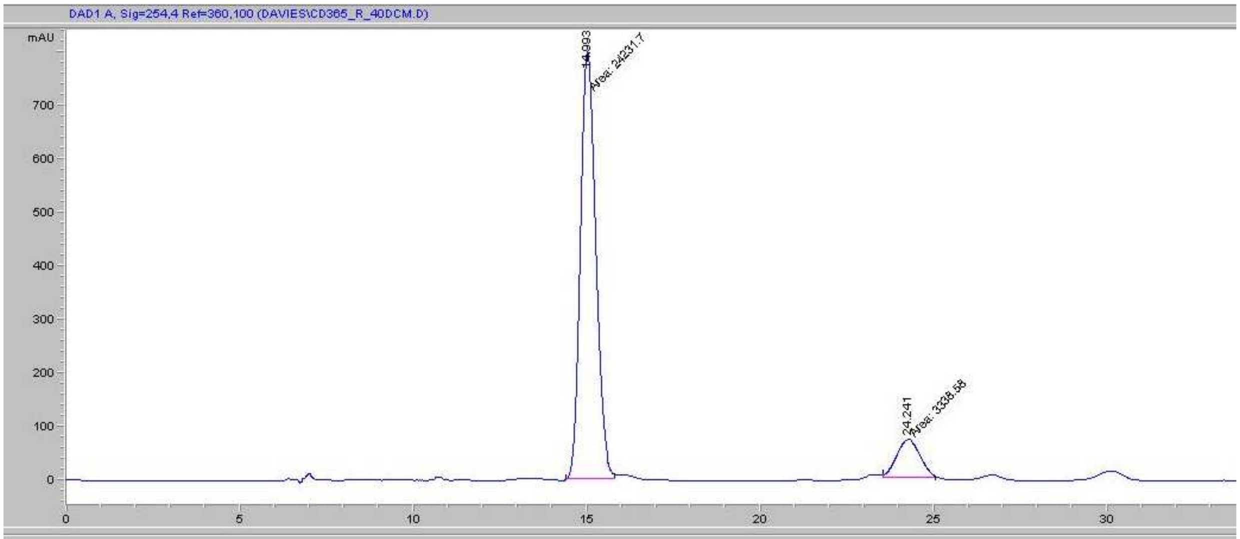

File Information	
LC-File	CD365_R_40DCM.D
File Path	C:\HPCHEM\1\DATA\DAVIES\
Date	21-May-21, 10:08:25

#	Time	Area	Height	Width	Area%	Symmetry
1	14.993	24231.7	800.7	0.5044	87.891	0.849
2	24.241	3338.6	71.2	0.7817	12.109	0.978

2-(tert-butyl) 3-ethyl 6-methyl (2S,6R)-6-(1-methyl-1H-indol-2-yl)-1-((2-nitrophenyl)sulfonyl)-1,2,5,6-tetrahydropyridine-2,3,6-tricarboxylate (3b)

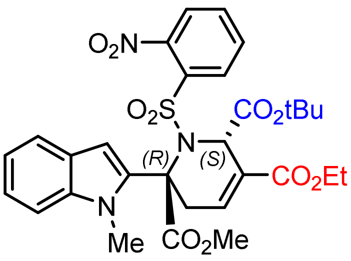

***(S,R)*-3b**

Off-white solid, 67% yield. Pent/EtOAc (6:1 to 4:1). **HR-MS:** calc. for $[M+H]^+$ $C_{30}H_{34}O_{10}N_3S$ = 628.1959, found: 628.1957. $[\alpha]^{RT}_{D}$ = - 97.0° (CH_2Cl_2, c = 1.00); HPLC conditions: CHIRAPAK IC column, DCM:EtOH (100:2)/ *iso*-hexane = 40/60, flow rate = 0.5 mL min^{-1}, minor enantiomer: t_R= 13.90 min; major enantiomer: t_R= 22.14 min, 76% *ee*.

Single enantiomer purified using CHIRAPAK IC column, DCM:EtOH (100:2)/*iso*-hexane = 30/70, flow rate = 3 mL min^{-1}. White foam, 47%.

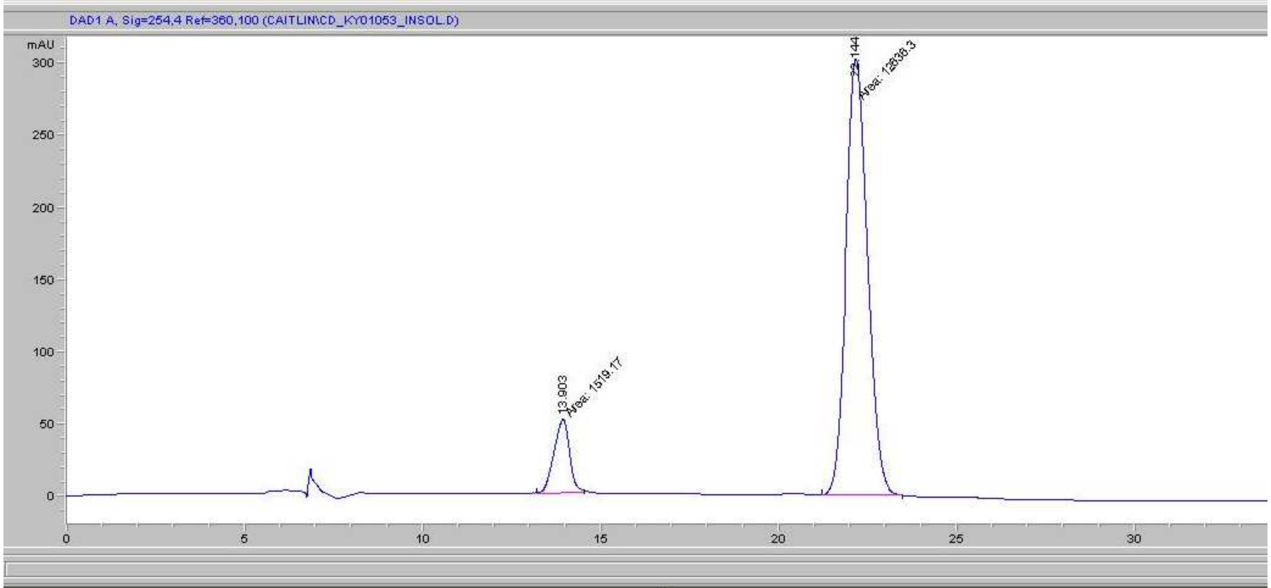

File Information	
LC-File	CD_KY01053_INSOL.D
File Path	C:\HPCHEM\1\DATA\CAITLIN\
Date	05-Oct-20, 10:34:25

#	Time	Area	Height	Width	Area%	Symmetry
1	13.903	1519.2	50.9	0.4979	10.732	1.261
2	22.144	12636.3	301.5	0.6985	89.268	0.882

2-(tert-butyl) 3-ethyl (2S,6R)-6-(1-methyl-1H-indol-2-yl)-1-((2-nitrophenyl)sulfonyl)-1,2,5,6-tetrahydropyridine-2,3-dicarboxylate (3c)

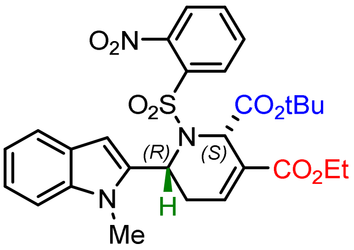

***(S,R)*-3c**

Off-white foam, 42% yield. Pent/DCM (1:5) **^{1}H-NMR (500 MHz - $CDCl_3$):** δ 7.99 (dd, *J* = 8.0, 1.4 Hz, 1H), 7.73 (td, *J* = 7.8, 1.4 Hz, 1H), 7.66 (td, *J* = 7.7, 1.3 Hz, 1H), 7.56 (dd, *J* = 7.9, 1.3 Hz, 1H), 7.45 (dt, *J* = 7.8, 1.0 Hz, 1H), 7.29 (dd, *J* = 8.4, 0.9 Hz, 1H), 7.17 (ddd, *J* = 8.3, 7.0, 1.1 Hz, 1H), 7.01 (ddd, *J* = 7.9, 7.0, 1.0 Hz, 1H), 6.97 – 6.93 (m, 1H), 6.33 (d, *J* = 0.9 Hz, 1H), 5.65 (d, *J* = 7.2 Hz, 1H), 5.59 (dd, *J* = 2.4, 1.3 Hz, 1H), 4.32 – 4.16 (m, 2H), 3.87 (s, 3H), 2.84 (dd, *J* = 20.0, 5.4 Hz, 1H), 2.70 – 2.60 (m, 1H), 1.33 (t, *J* = 7.1 Hz, 3H), 0.77 (s, 9H); **^{13}C-NMR (126 MHz - $CDCl_3$):** δ 165.53, 165.28, 148.34, 138.09, 134.65, 134.19, 133.88, 132.79, 131.69, 130.47, 127.79, 126.64, 123.83, 122.40, 120.79, 119.51, 109.45, 103.40, 82.74, 61.11, 54.08, 46.77, 30.16, 27.46, 27.14 (3C, tBu), 14.29; **HR-MS:** calc. for $[M+H]^+$, $C_{28}H_{32}O_8N_3S$ = 570.19046, found: 570.19016. $[\alpha]^{RT}_{D}$ = - 82.0° (CH_2Cl_2, c = 1.00); HPLC conditions: CHIRAPAK IC column, DCM:EtOH (100:2)/ *iso*-hexane = 40/60, flow rate = 0.5 mL min^{-1}, minor enantiomer: t_R= 15.19 min; major enantiomer: t_R= 17.46 min, 81% *ee*

Single enantiomer purified using CHIRAPAK IC column, DCM:EtOH (100:3)/*iso*-hexane = 40/60, flow rate = 3 mL min^{-1}. Yellow foam.

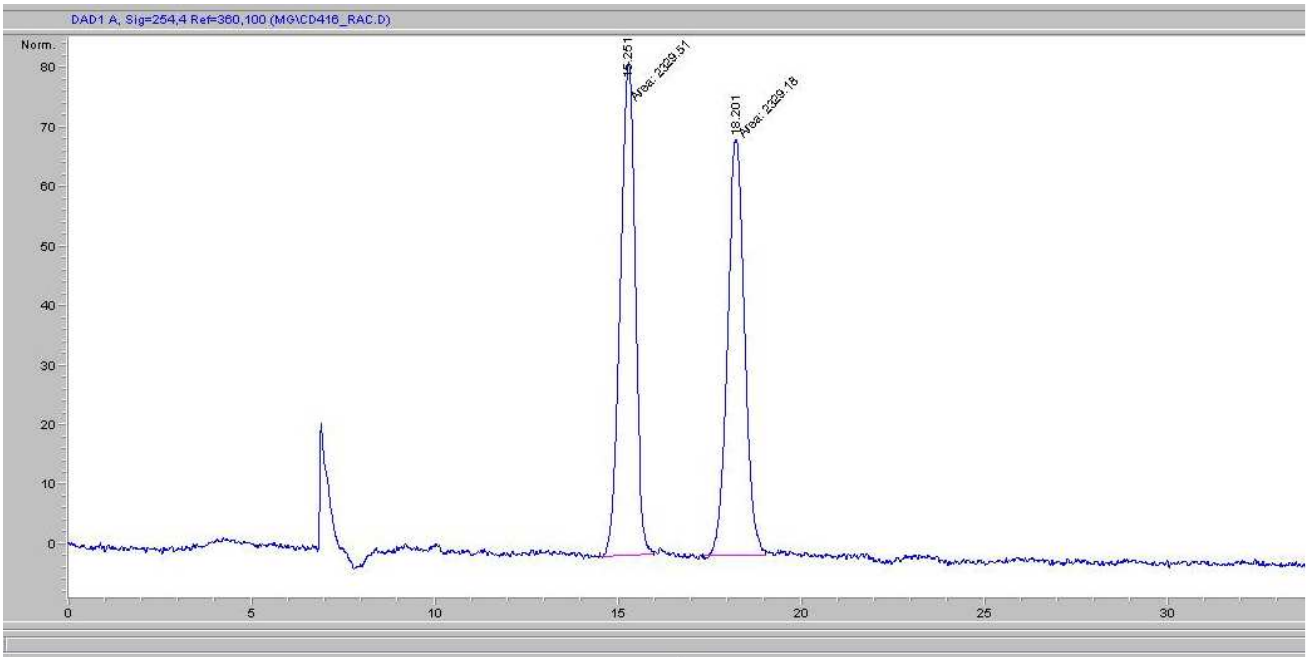

File Information	
LC-File	CD416_RAC.D
File Path	C:\HPCHEM\1\DATA\MG\
Date	21-Nov-20, 01:33:28

#	Time	Area	Height	Width	Area%	Symmetry
1	15.251	2329.5	82.9	0.4683	50.004	1.182
2	18.201	2329.2	69.9	0.5554	49.996	1.059

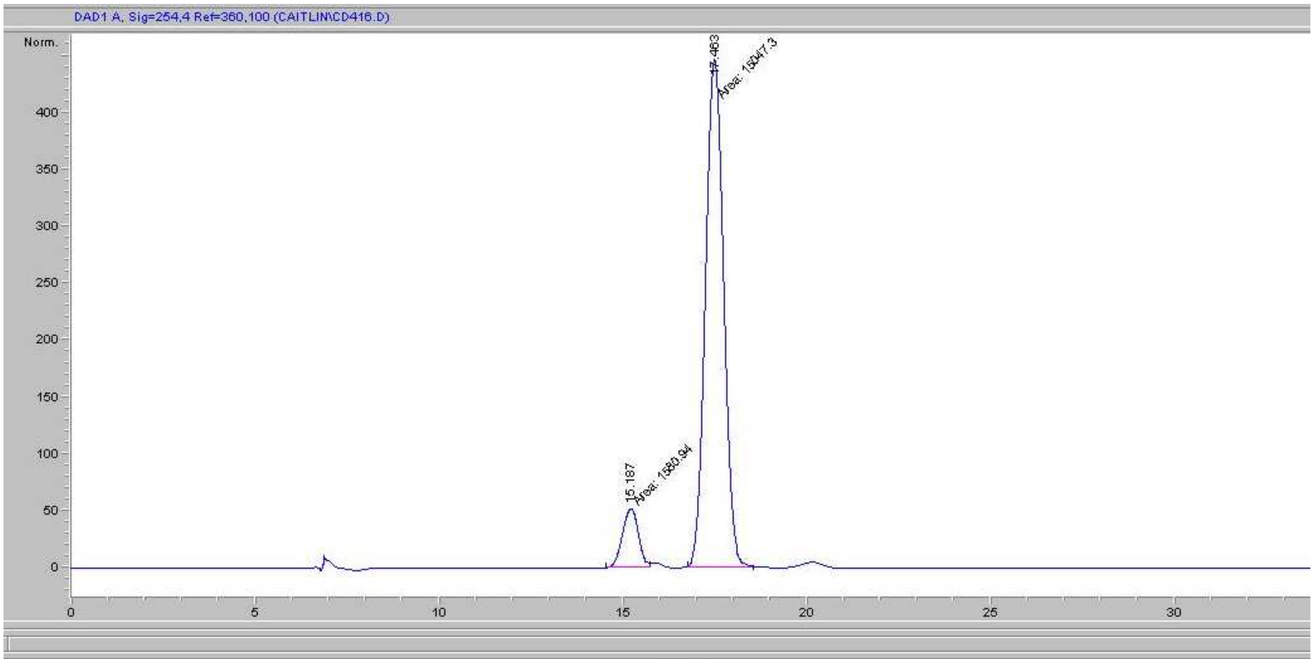

File Information	
LC-File	CD416.D
File Path	C:\HPCHEM\1\DATA\CAITLIN\
Date	05-Oct-20, 18:11:40

#	Time	Area	Height	Width	Area%	Symmetry
1	15.187	1580.9	52.4	0.5028	9.508	1.082
2	17.463	15047.3	446.8	0.5613	90.492	0.968

2-(tert-butyl) 3-ethyl 6-methyl (2R,6S)-6-(1-methyl-1H-indol-2-yl)-1-tosyl-1,2,5,6-tetrahydropyridine-2,3,6-tricarboxylate (3d)

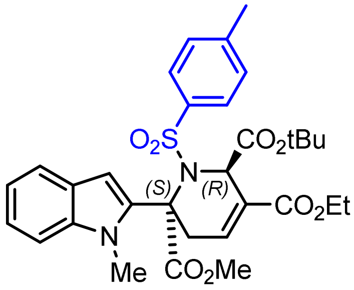

***(R,S)*-3d**

Off-white foam, 91% yield. Pent/EtOAc (6:1 to 4:1) **^{1}H-NMR (500 MHz - $CDCl_3$):** δ 7.88 (bs/d, 2H), 7.52 (dt, J = 7.8, 1.0 Hz, 1H), 7.29 (d, J = 8.2 Hz, 2H), 7.20 – 7.11 (m, 3H), 7.05 (ddd, J = 7.9, 6.5, 1.4 Hz, 1H), 6.46 (s, 1H), 5.92 (s, J = 1.4 Hz, 1H), 4.31 – 4.09 (m, 2H), 3.65 (s, 3H), 3.58 (s, 3H), 3.55 – 3.47 (m, 1H), 3.42 (d, J = 19.9 Hz, 1H), 2.44 (s, 3H), 1.32 (t, J = 7.2 Hz, 3H), 0.79 (bs, 9H); **^{13}C-NMR (126 MHz - $CDCl_3$):** δ 168.87, 166.74, 164.94, 143.35, 139.47, 138.96, 135.13, 131.66, 129.08 (2C), 128.68, 127.35, 126.17, 122.77, 120.83, 120.53, 119.85, 110.06, 106.16, 82.44, 65.07, 61.02, 58.39, 53.14, 33.88, 33.00, 27.16 (3C, tBu), 21.68, 14.32; **HR-MS:** calc. for $[M+H]^+$, $C_{31}H_{37}O_8N_2S$ = 597.22651, found: 597.22631. $[\alpha]^{RT}_D$ = + 88.0° (CH_2Cl_2, c = 1.00); HPLC conditions: CHIRAPAK IC column, DCM:MeOH (100:5)/ *iso*-hexane = 20/80, flow rate = 0.5 mL min^{-1}, minor enantiomer: t_R= 33.58 min; major enantiomer: t_R= 27.00 min, 84% *ee*.

Single enantiomer obtained through purification of ***(S,R)*-3d** using CHIRAPAK IC column, DCM:EtOH (100:2)/*iso*-hexane = 30/70, flow rate = 3 mL min^{-1}. White foam, 15%.

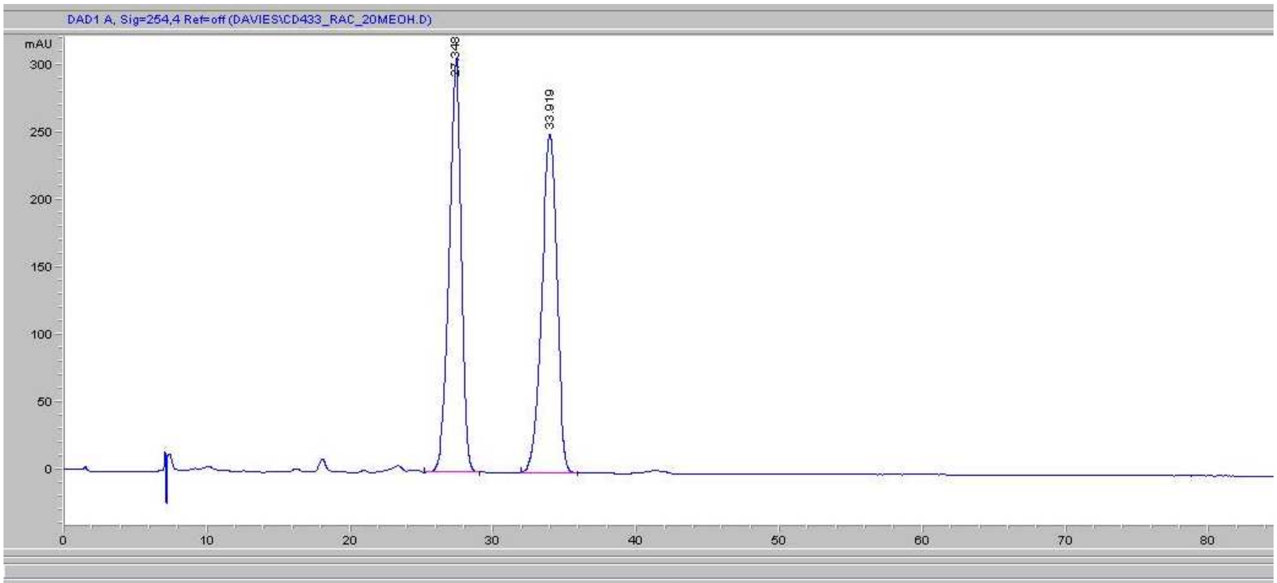

File Information	
LC-File	CD433_RAC_20MEOH.D
File Path	C:\HPCHEM\1\DATA\DAVIES\
Date	19-May-21, 18:01:12

#	Time	Area	Height	Width	Area%	Symmetry
1	27.348	18375.8	306.5	0.9155	49.918	1.261
2	33.919	18436	250.8	1.1538	50.082	1.186

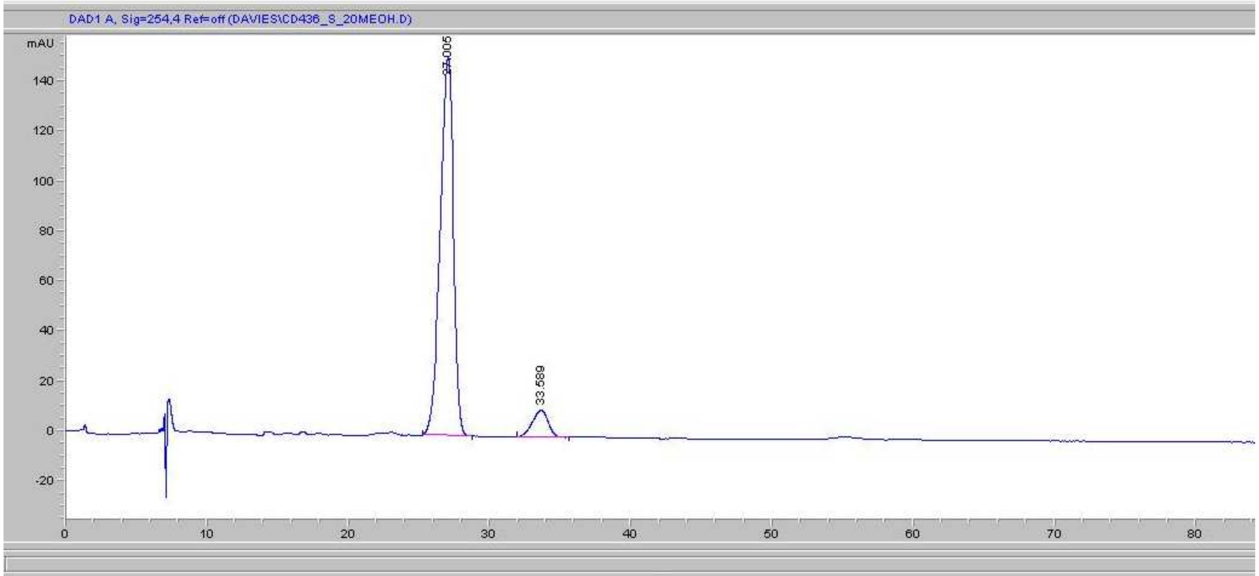

File Information	
LC-File	CD436_S_20MEOH.D
File Path	C:\HPCHEM\1\DATA\DAVIES\
Date	20-May-21, 17:31:09

#	Time	Area	Height	Width	Area%	Symmetry
1	27.005	9889.7	151.1	0.9874	92.207	1.317
2	33.589	835.9	11	0.9744	7.793	1.245

2-(tert-butyl) 3-ethyl 6-methyl (2S,6R)-6-(1-methyl-1H-indol-2-yl)-1-tosyl-1,2,5,6-tetrahydropyridine-2,3,6-tricarboxylate (3d)

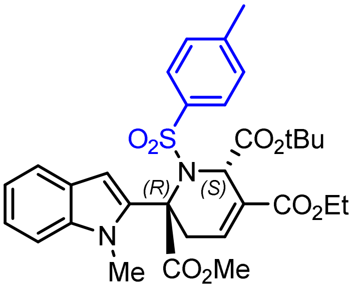

***(S,R)*-3d**

Off-white foam, 93% yield. Pent/EtOAc (6:1 to 4:1). **HR-MS:** calc. for $[M+H]^+$, $C_{31}H_{37}O_8N_2S$ = 597.22651, found: 597.22627. $[\alpha]^{RT}_{D}$ = - 77.0° (CH_2Cl_2, c = 1.00); HPLC conditions: CHIRAPAK IC column, DCM:MeOH (100:5)/ *iso*-hexane = 20/80, flow rate = 0.5 mL min^{-1}, minor enantiomer: t_R= 29.64 min; major enantiomer: t_R= 36.65 min, 84% *ee*.

Single enantiomer purified using CHIRAPAK IC column, DCM:EtOH (100:2)/*iso*-hexane = 30/70, flow rate = 3 mL min^{-1}. White foam, 40%.

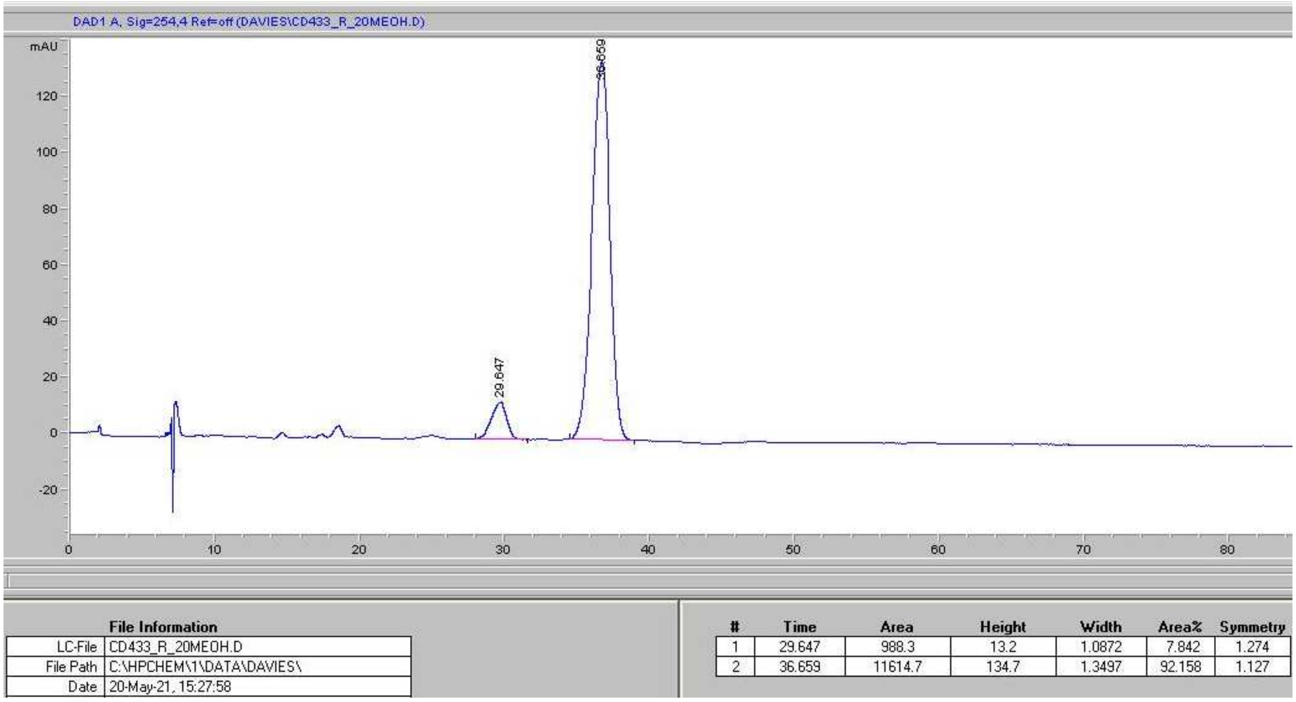

2-(tert-butyl) 3-ethyl (2S,6R)-6-(1-methyl-1H-indol-2-yl)-1-tosyl-1,2,5,6-tetrahydropyridine-2,3-dicarboxylate (3e)

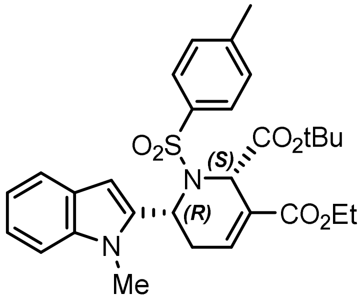

(S,R)-3e

Off-white foam, 52% yield. Pent/EtOAc (6:1 to 5:1) **^{1}H-NMR (700 MHz - $CDCl_3$):** δ 7.81 – 7.77 (m, 2H), 7.43 (dt, *J* = 7.8, 1.0 Hz, 1H), 7.28 (d, *J* = 8.5, 1.0 Hz, 3H), 7.16 (ddd, *J* = 8.3, 7.0, 1.2 Hz, 1H), 7.00 (ddd, *J* = 7.9, 7.0, 0.9 Hz, 1H), 6.85 – 6.81 (m, 1H), 6.26 (s, 1H), 5.55 (d, *J* = 7.3 Hz, 1H), 5.40 (s, 1H), 4.28 – 4.16 (m, 2H), 3.90 (s, 3H), 2.70 (dd, *J* = 19.8, 5.4 Hz, 1H), 2.43 (s, 3H), 2.41 – 2.34 (m, 1H), 1.31 (t, *J* = 7.1 Hz, 3H), 0.74 (s, 9H); **^{13}C-NMR (126 MHz - $CDCl_3$):** δ 166.23, 165.75, 144.47, 138.39, 137.27, 135.00, 134.76, 130.14 (2C), 128.27, 127.77 (2C), 127.05, 122.51, 121.04, 119.72, 109.66, 103.11, 82.62, 61.23, 53.76, 46.58, 30.41, 27.47 (3C, tBu), 26.93, 21.96, 14.63; **HR-MS:** calc. for $[M+H]^+$, $C_{29}H_{35}O_6N_2S$ = 539.22103, found: 539.22061. $[\alpha]^{RT}_{D}$ = - 136.0° (CH_2Cl_2, c = 1.00); HPLC conditions: CHIRAPAK IC column, DCM:EtOH (100:2)/ *iso*-hexane = 30/70, flow rate = 0.5 mL min^{-1}, minor enantiomer: t_R= 27.18 min; major enantiomer: t_R= 24.49 min, 84% *ee*.

Single enantiomer purified using CHIRAPAK IC column, DCM:EtOH (100:2)/*iso*-hexane = 30/70, flow rate = 3 mL min^{-1}. White foam, 52%.

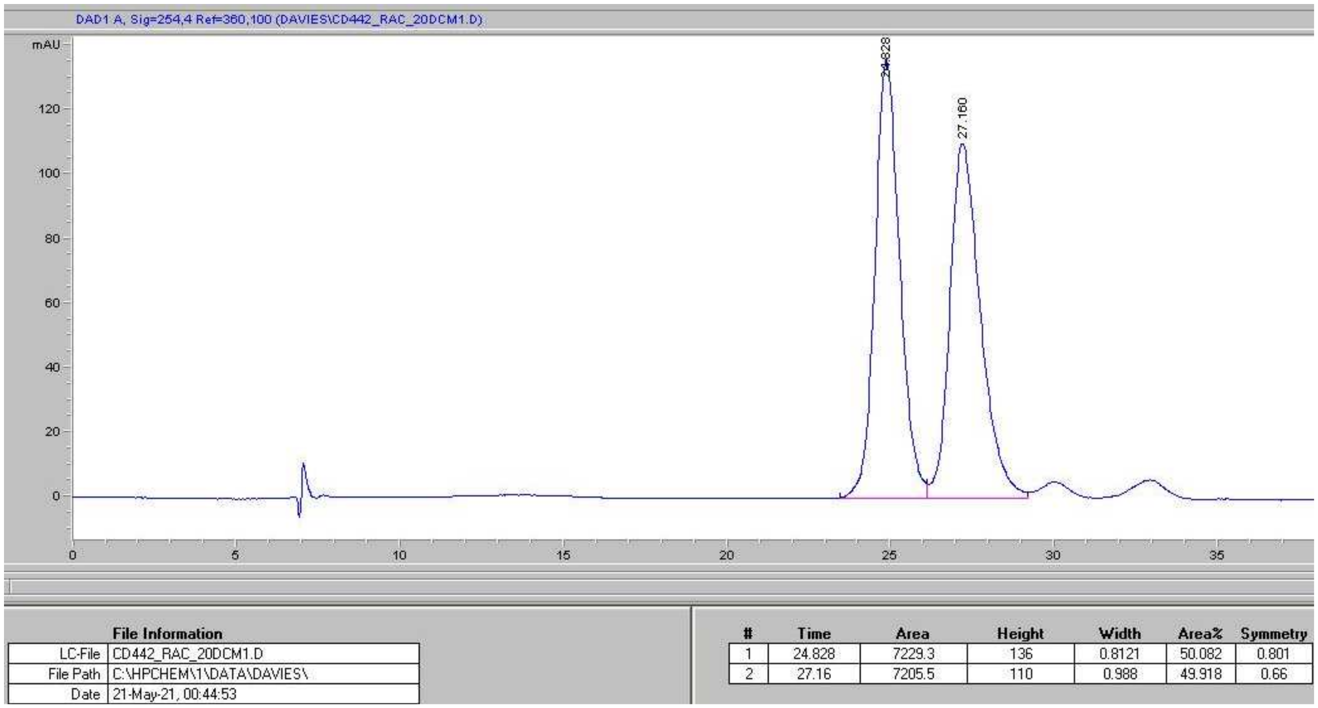

File Information	
LC-File	CD442_RAC_20DCM1.D
File Path	C:\HPCHEM\1\DATA\DAVIES\
Date	21-May-21, 00:44:53

#	Time	Area	Height	Width	Area%	Symmetry
1	24.828	7229.3	136	0.8121	50.082	0.801
2	27.16	7205.5	110	0.988	49.918	0.66

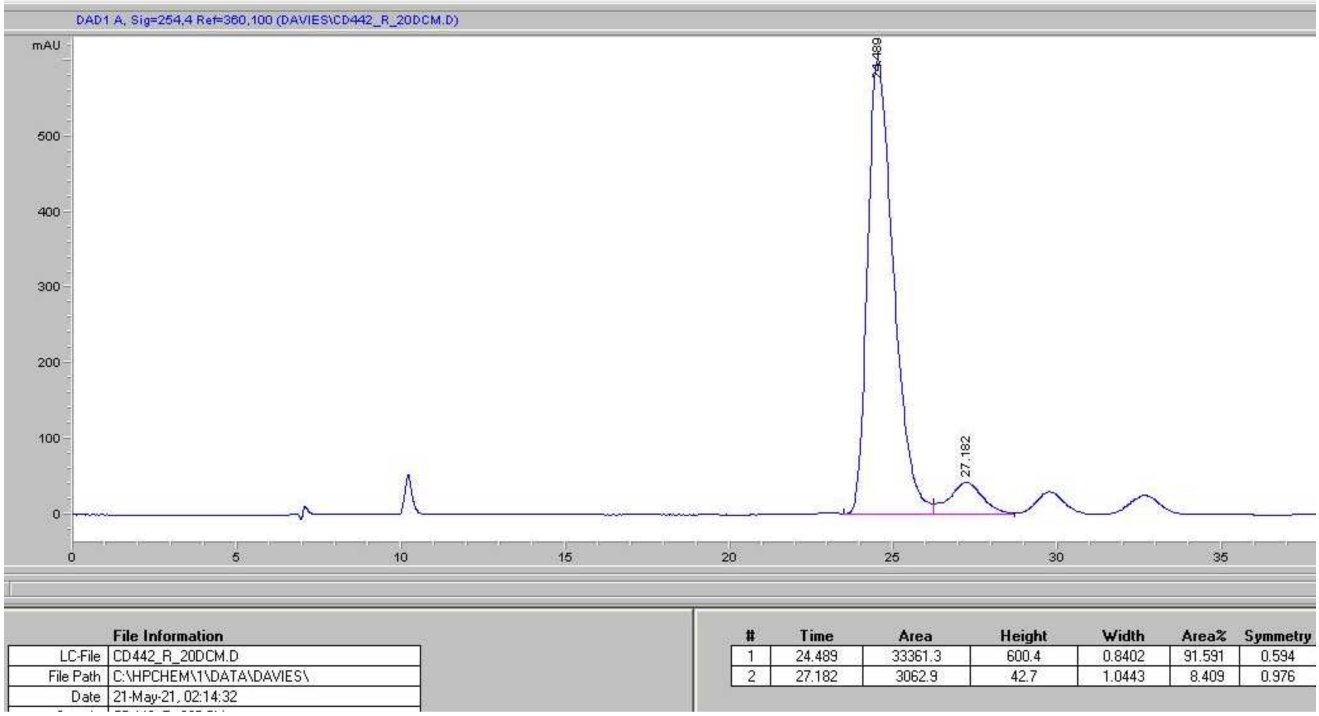

File Information	
LC-File	CD442_R_20DCM.D
File Path	C:\HPCHEM\1\DATA\DAVIES\
Date	21-May-21, 02:14:32
[illegible]	[illegible]

#	Time	Area	Height	Width	Area%	Symmetry
1	24.489	33361.3	600.4	0.8402	91.591	0.594
2	27.182	3062.9	42.7	1.0443	8.409	0.976

2-(tert-butyl) 3-ethyl 6-methyl (2S,6R)-6-(1-methyl-1H-indol-2-yl)-1-(methylsulfonyl)-1,2,5,6-tetrahydropyridine-2,3,6-tricarboxylate (3f)

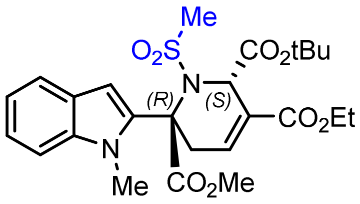

***(S,R)*-3f**

Off-white powder, 88% yield. Pent/EtOAc (5:1) **^{1}H-NMR (500 MHz - $CDCl_3$):** δ 7.54 (dt, *J* = 8.0, 1.0 Hz, 1H), 7.24 (d, *J* = 1.1 Hz, 1H), 7.20 (ddd, *J* = 8.3, 6.8, 1.2 Hz, 1H), 7.16 (ddd, *J* = 5.5, 3.0, 1.5 Hz, 1H), 7.07 (ddd, *J* = 7.9, 6.8, 1.1 Hz, 1H), 6.47 (s, 1H), 5.71 (t, *J* = 1.7 Hz, 1H), 4.23 (dq, *J* = 10.9, 7.1 Hz, 1H), 4.13 (dq, *J* = 11.0, 7.2 Hz, 1H), 3.92 (s, 3H), 3.71 (s, 3H), 3.58 – 3.49 (m, 1H), 3.47 (s, 3H), 3.36 (dt, *J* = 19.6, 2.6 Hz, 1H), 1.30 (t, *J* = 7.1 Hz, 3H), 0.74 (s, 9H).; **^{13}C-NMR (126 MHz - $CDCl_3$):** δ 169.43, 166.90, 165.01, 139.70, 135.48, 131.53, 128.52, 126.27, 123.09, 121.01, 120.10, 110.28, 106.50, 82.65, 65.14, 61.13, 58.12, 53.46, 43.94, 33.95, 33.55, 27.24 (3C, tBu), 14.40; **HR-MS:** calc. for $[M+H]^+$, $C_{25}H_{33}O_8N_2S$ = 521.19521, found: 521.19471. $[\alpha]^{RT}_{D}$ = - 215.0° (CH_2Cl_2, c = 1.00); HPLC conditions: CHIRAPAK IC column, DCM:MeOH (100:5)/ *iso*-hexane = 20/80, flow rate = 0.5 mL min^{-1}, minor enantiomer: t_R= 28.76 min; major enantiomer: t_R= 36.13 min, 84% *ee*.

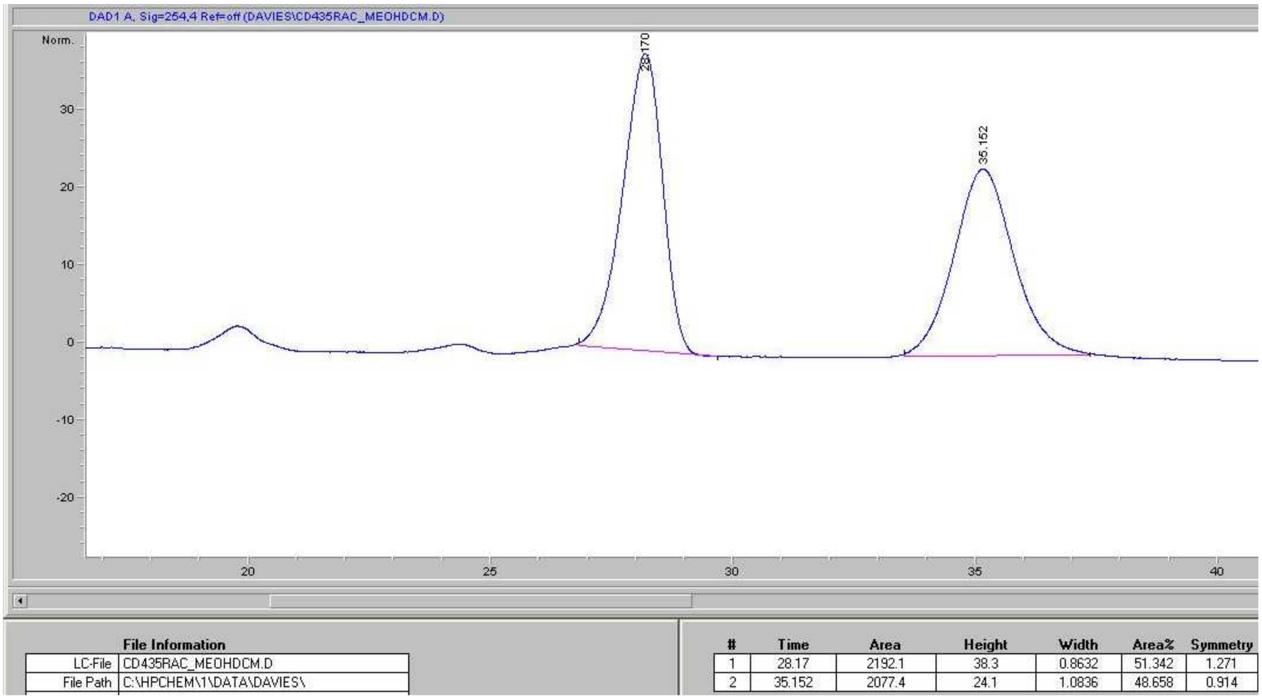

File Information	
LC-File	CD435RAC_MEOHDCM.D
File Path	C:\HPCHEM\1\DATA\DAVIES\

#	Time	Area	Height	Width	Area%	Symmetry
1	28.17	2192.1	38.3	0.8632	51.342	1.271
2	35.152	2077.4	24.1	1.0836	48.658	0.914

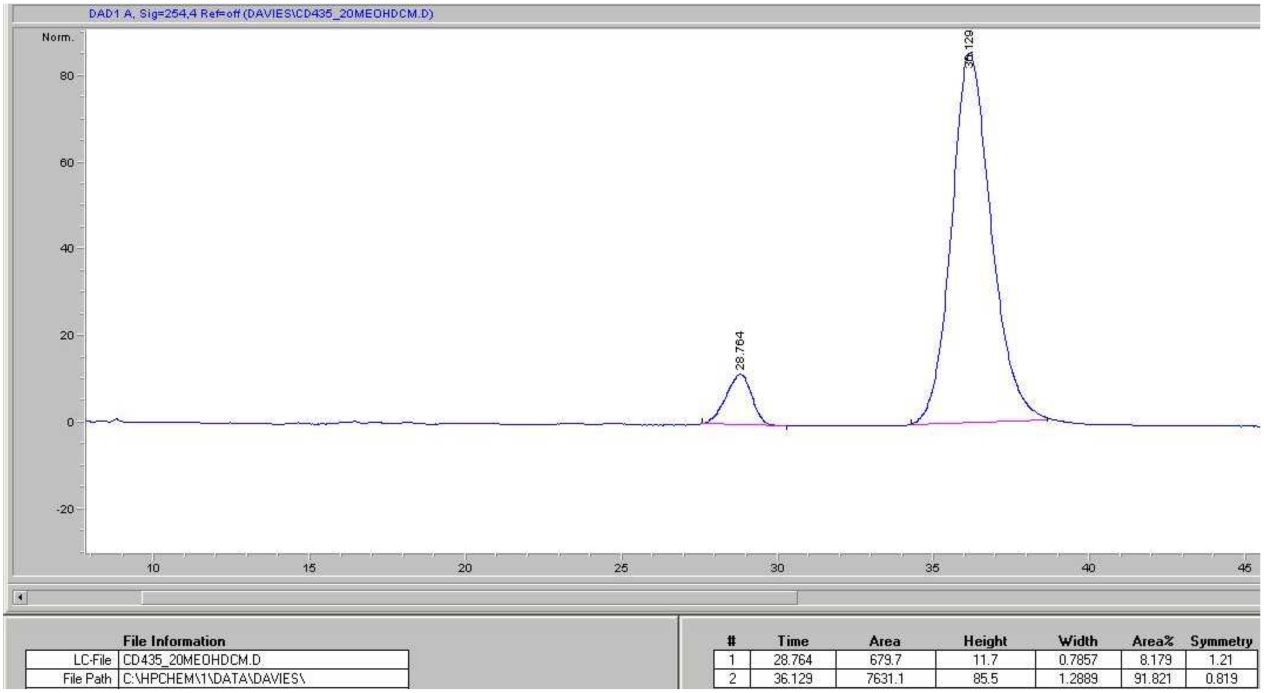

File Information	
LC-File	CD435_20MEOHDCM.D
File Path	C:\HPCHEM\1\DATA\DAVIES\

#	Time	Area	Height	Width	Area%	Symmetry
1	28.764	679.7	11.7	0.7857	8.179	1.21
2	36.129	7631.1	85.5	1.2889	91.821	0.819

2-(tert-butyl) 3-ethyl 6-methyl (2S,6R)-6-(1-methyl-1H-indol-2-yl)-1-(phenylsulfonyl)-1,2,5,6-tetrahydropyridine-2,3,6-tricarboxylate (3g)

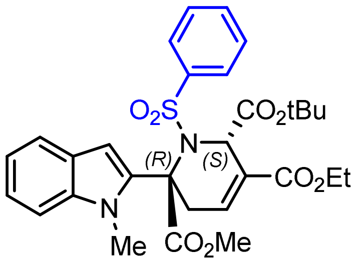

***(S,R)*-3g**

Yellow foam, 70% yield. Pent/EtOAc (5:1 to 4:1) **^{1}H-NMR (500 MHz - $CDCl_3$):** δ 8.01 (s, 2H), 7.57 (d, *J* = 7.4 Hz, 1H), 7.54 – 7.47 (m, 3H), 7.19 – 7.10 (m, 3H), 7.05 (ddd, *J* = 7.8, 6.7, 1.3 Hz, 1H), 6.47 (s, 1H), 5.96 (s, *J* = 1.4 Hz, 1H), 4.26 (dq, *J* = 11.0, 7.2 Hz, 1H), 4.22 – 4.11 (m, 1H), 3.64 (s, 3H), 3.55 (s, 3H), 3.53 – 3.49 (m, 1H), 3.43 (d, *J* = 21.1 Hz, 1H), 1.32 (t, *J* = 7.2 Hz, 3H), 0.80 (s, 9H); **^{13}C-NMR (126 MHz - $CDCl_3$):** δ 169.20, 167.13, 165.27, 142.25, 139.82, 135.50, 132.98, 131.93, 129.10, 128.81 (2C), 128.49, 127.68 (2C), 126.54, 123.20, 121.23, 120.26, 110.45, 106.65, 82.89, 65.55, 61.43, 58.79, 53.51, 33.25, 27.54 (3C, tBu), 14.69; **HR-MS:** calc. for $[M+H]^+$, $C_{30}H_{35}O_8N_2S$ = 583.21086, found: 583.21059. $[\alpha]^{RT}_D$ = - 68.0° (CH_2Cl_2, c = 1.00); HPLC conditions: CHIRAPAK IC column, DCM:MeOH (100:5)/ *iso*-hexane = 20/80, flow rate = 0.5 mL min^{-1}, minor enantiomer: t_R= 23.61 min; major enantiomer: t_R= 28.12 min, 78% *ee*.

Single enantiomer purified using CHIRAPAK IC column, DCM:MeOH (100:5)/*iso*-hexane = 30/70, flow rate = 3 mL min^{-1}. White foam, 33%.

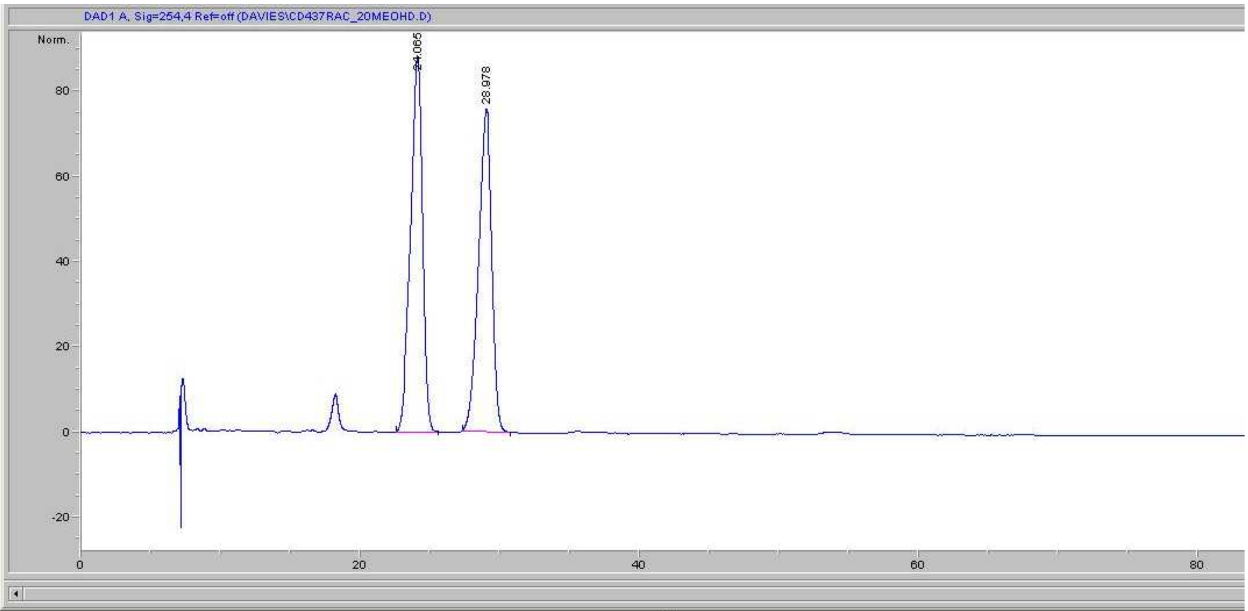

	File Information
LC-File	CD437RAC_20MEOHD.D
File Path	C:\HPCHEM\1\DATA\DAVIES\

#	Time	Area	Height	Width	Area%	Symmetry
1	24.065	5152.8	88.3	0.8446	50.180	1.405
2	28.978	5115.9	75.9	1.0026	49.820	1.301

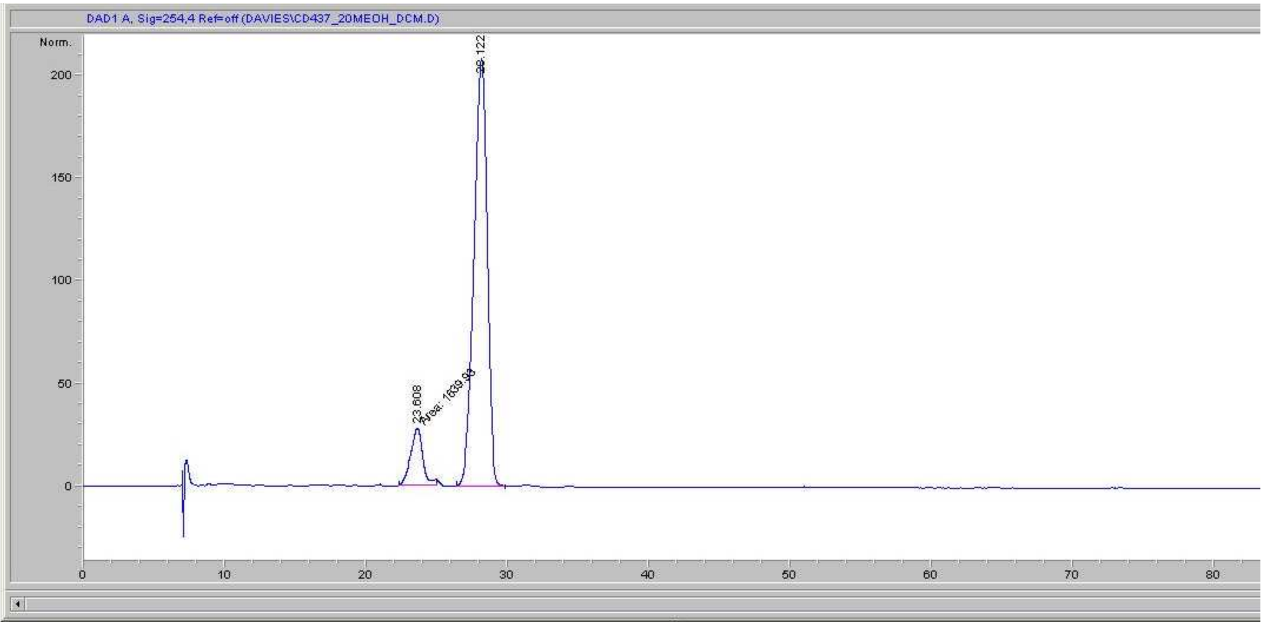

	File Information
LC-File	CD437_20MEOH_DCM.D
File Path	C:\HPCHEM\1\DATA\DAVIES\

#	Time	Area	Height	Width	Area%	Symmetry
1	23.608	1639.9	27.8	0.982	10.976	1.247
2	28.122	13300.7	208.2	0.9712	89.024	1.287

2-(tert-butyl) 3-ethyl 6-methyl (2S,6R)-1-((4-bromophenyl)sulfonyl)-6-(1-methyl-1H-indol-2-yl)-1,2,5,6-tetrahydropyridine-2,3,6-tricarboxylate (3h)

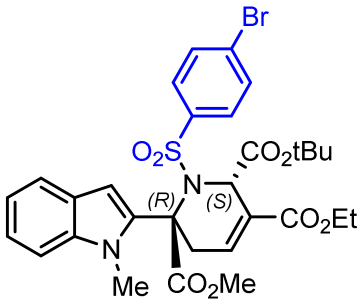

***(S,R)*-3h**

Yellow oil, 75% yield. Pent/EtOAc (6:1 to 5:1) **^{1}H-NMR (500 MHz - CDCl$_3$):** δ 7.97 – 7.72 (m, 2H), 7.60 (d, *J* = 8.1 Hz, 2H), 7.52 (dt, *J* = 7.9, 1.0 Hz, 1H), 7.21 – 7.11 (m, 3H), 7.06 (ddd, *J* = 8.0, 6.7, 1.2 Hz, 1H), 6.47 (s, 1H), 5.96 (s, 1H), 4.26 (dq, *J* = 10.8, 7.1 Hz, 1H), 4.17 (dq, *J* = 10.9, 7.2 Hz, 1H), 3.65 (s, 3H), 3.53 (m, 4H, overlapping of singlet and double doublet), 3.45 – 3.33 (m, 1H), 1.33 (t, *J* = 7.2 Hz, 3H), 0.82 (s, 9H); **^{13}C-NMR (126 MHz - CDCl$_3$):** δ 168.98, 166.95, 164.91, 139.49, 135.20, 131.74 (2C), 131.37, 129.06 (2C), 127.63, 126.25, 123.10, 121.00, 120.12, 110.18, 106.58, 82.82, 65.45, 61.24, 58.59, 53.36, 34.29, 32.93, 27.30 (3C, tBu), 14.44; **HR-MS:** calc. for $[M+H]^+$, $C_{30}H_{34}O_8N_2BrS$ = 661.12138, found: 661.12143 and $C_{30}H_{34}O_8N_2{}^{81}BrS$ = 663.11933, found: 663.11926. $[\alpha]^{RT}_{D}$ = - 53.0° (CH_2Cl_2, c = 1.00); HPLC conditions: CHIRAPAK IA column, DCM:EtOH (100:2)/ *iso*-hexane = 10/90, flow rate = 0.5 mL min^{-1}, minor enantiomer: t_R= 27.90 min; major enantiomer: t_R= 38.35 min, 83% *ee*.

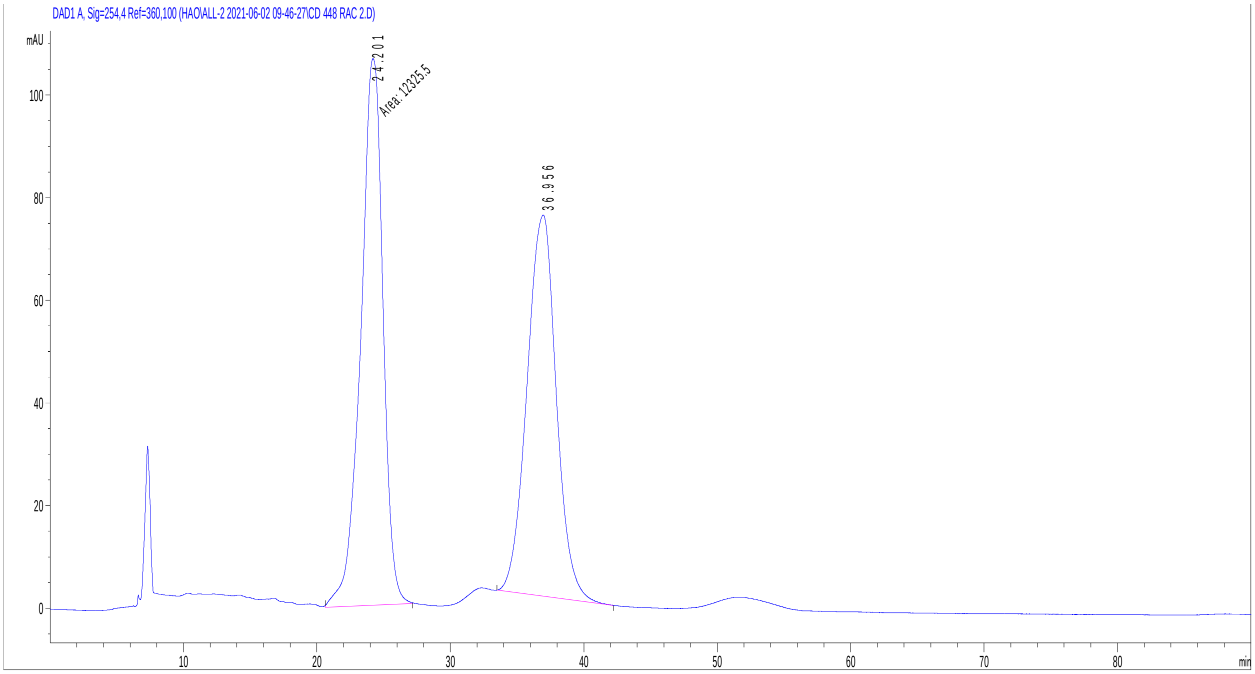

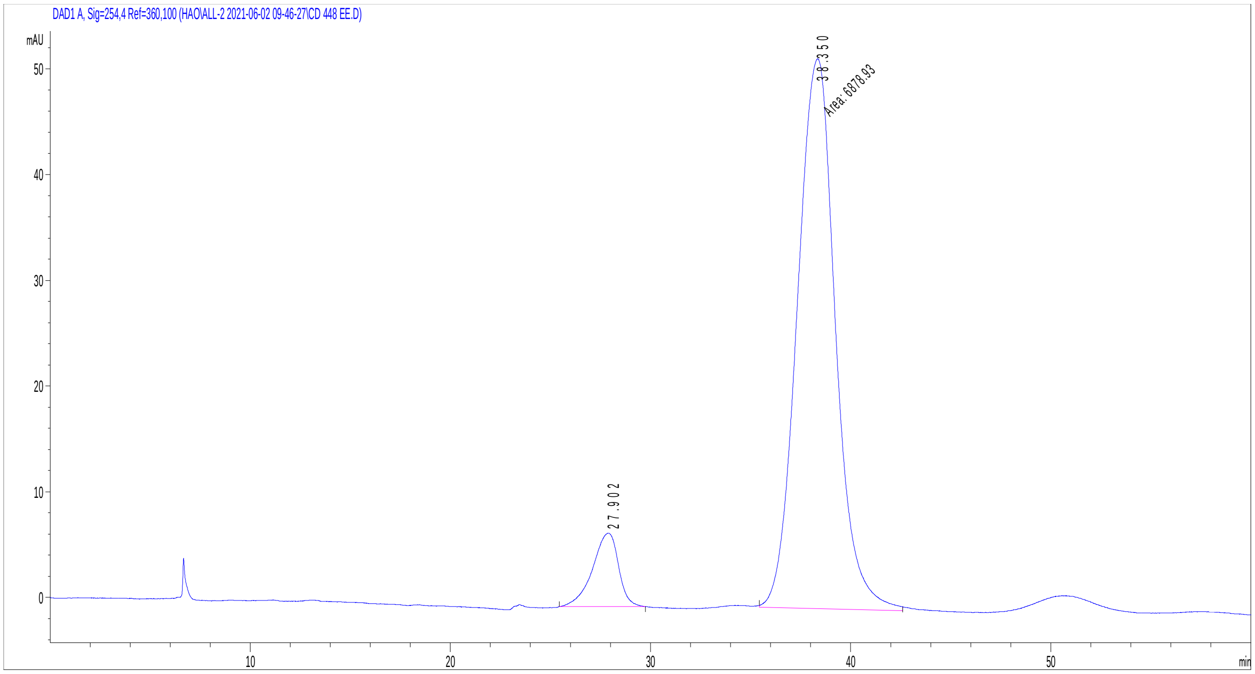

#	Time	Area	Height	Width	Area%	Symmetry
1	27.902	633	7	1.0714	8.427	1.533
2	38.35	6878.9	52	2.2052	91.573	1.076

2-(tert-butyl) 3-ethyl 6-methyl (2S,6R)-6-(1-methyl-1H-indol-2-yl)-1-((3-(trifluoromethyl)phenyl)sulfonyl)-1,2,5,6-tetrahydropyridine-2,3,6-tricarboxylate (3i)

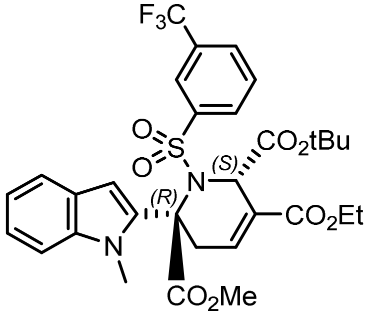

***(S,R)*-3i**

Yellow solid, % yield. Pent/EtOAc (5:1 to 3:1) **^{1}H-NMR (500 MHz - $CDCl_3$):** δ 8.35 (s, 1H), 8.22 – 8.01 (m, 1H), 7.81 (d, *J* = 7.8 Hz, 1H), 7.57 (bs, 1H), 7.52 (d, *J* = 7.9 Hz, 1H), 7.20 – 7.10 (m, 3H), 7.06 (ddd, *J* = 7.9, 6.7, 1.1 Hz, 1H), 6.47 (s, 1H), 6.00 (s, *J* = 1.7 Hz, 1H), 4.23 (ddq, *J* = 47.6, 10.9, 7.1 Hz, 2H), 3.64 (s, 3H), 3.55 (dd, *J* = 19.2, 5.0 Hz, 1H), 3.51 (s, 3H), 3.38 (m, *J* = 23.0, 6.7 Hz, 1H), 1.33 (t, *J* = 7.1 Hz, 4H), 0.85 (s, 9H); **^{13}C-NMR (126 MHz - $CDCl_3$):** δ 168.96, 167.01, 164.82, 139.46, 135.21, 131.30, 131.19, 130.93, 130.68, 129.20, 129.13 (d, *J* = 3.6 Hz), 126.22, 124.87, 124.59, 123.14, 122.42, 121.04, 120.15, 110.16, 106.72, 82.92, 65.59, 61.31, 58.61, 53.33, 34.51, 32.75, 27.30 (3C), 14.42; **^{19}F NMR (470 MHz, $CDCl_3$)** δ -62.76; **HR-MS:** calc. for $[M+H]^+$, $C_{31}H_{34}O_8N_2F_3S$ = 651.19825, found: 651.19815. $[\alpha]^{RT}_D$ = - 61.0° (CH_2Cl_2, c = 1.00); HPLC conditions: CHIRAPAK IC column, IPA/ *iso*-hexane = 10/90, flow rate = 0.5 mL min^{-1}, minor enantiomer: t_R= 32.18 min; major enantiomer: t_R= 23.36 min, 84% *ee*.

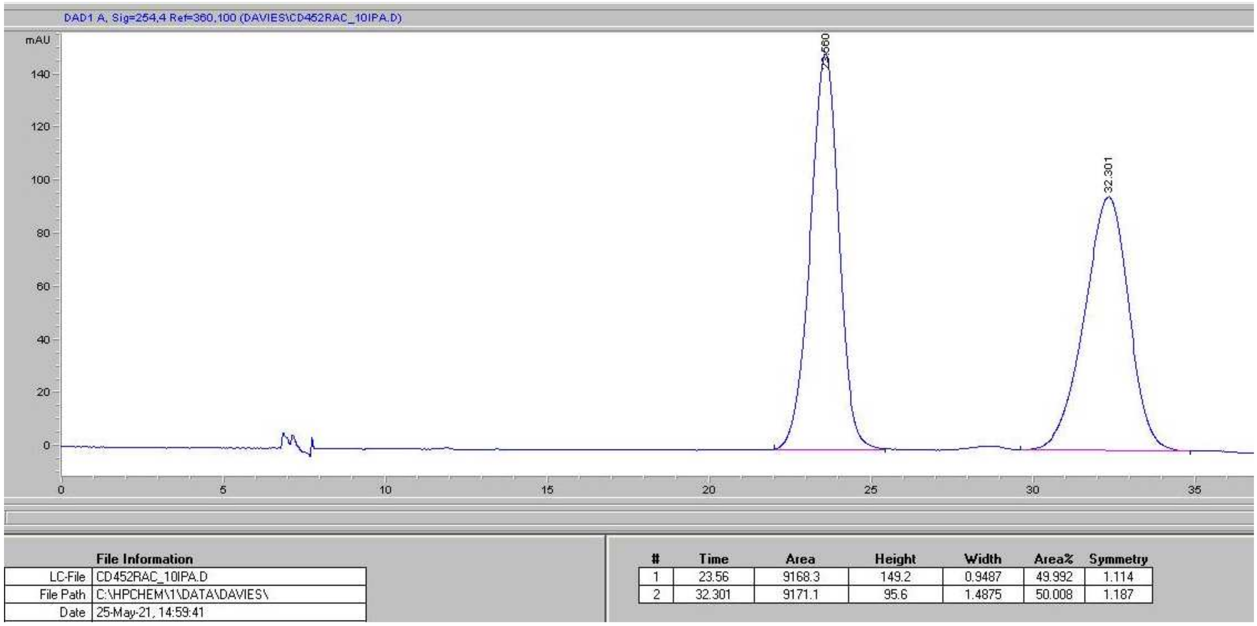

File Information

LC-File	CD452RAC_10IPA.D
File Path	C:\HPCHEM\1\DATA\DAVIES\
Date	25-May-21, 14:59:41

#	Time	Area	Height	Width	Area%	Symmetry
1	23.56	9168.3	149.2	0.9487	49.992	1.114
2	32.301	9171.1	95.6	1.4875	50.008	1.187

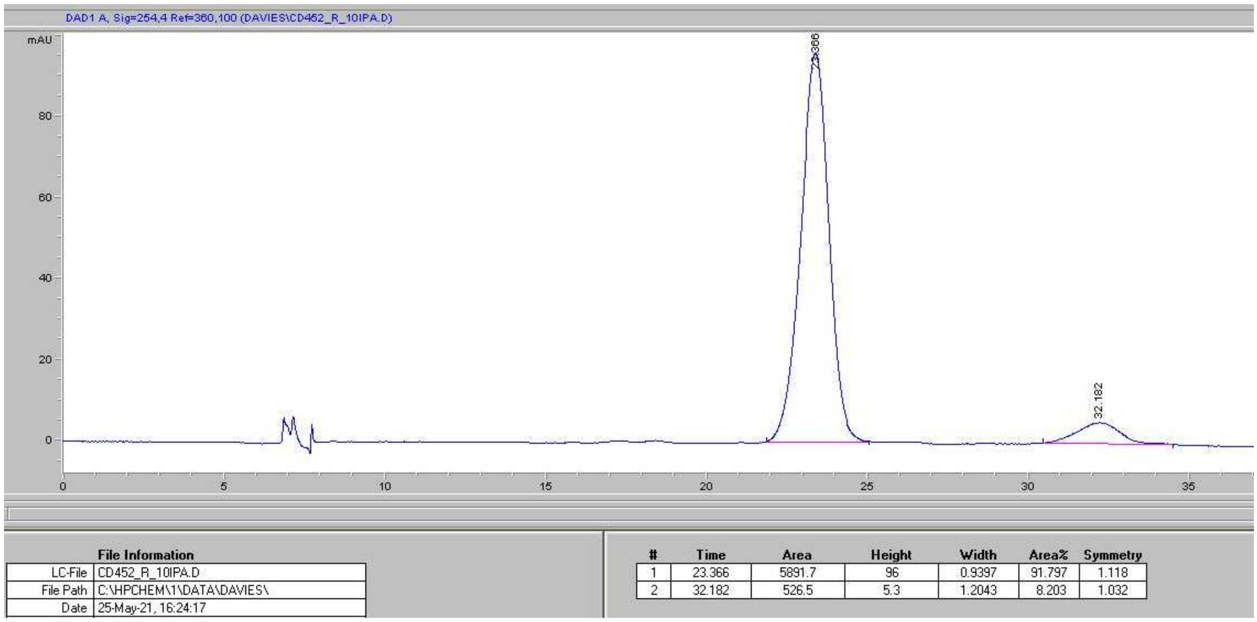

File Information

LC-File	CD452_R_10IPA.D
File Path	C:\HPCHEM\1\DATA\DAVIES\
Date	25-May-21, 16:24:17

#	Time	Area	Height	Width	Area%	Symmetry
1	23.366	5891.7	96	0.9397	91.797	1.118
2	32.182	526.5	5.3	1.2043	8.203	1.032

3-(tert-butyl) 2-ethyl 6-methyl (2S,6R)-6-(1-methyl-1H-indol-2-yl)-1-tosyl-1,2,5,6-tetrahydropyridine-2,3,6-tricarboxylate (3j)

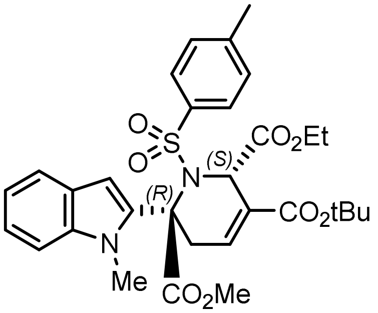

***(S,R)*-3j**

Orange foam, 91% yield. Pent/EtOAc (6:1 to 5:1) **^{1}H-NMR (500 MHz - $CDCl_3$):** δ 7.96 (d, J = 7.9 Hz, 2H), 7.52 (dt, J = 7.9, 1.0 Hz, 1H), 7.33 (d, J = 8.0 Hz, 2H), 7.21 – 7.11 (m, 3H), 7.04 (ddd, J = 7.9, 6.8, 1.1 Hz, 1H), 6.43 (s, 1H), 5.84 (s, 1H), 3.67 (s, 3H), 3.52 (s, 3H), 3.52 – 3.50 (m, 1H), 3.50 – 3.47 (m, 1H), 3.45 – 3.36 (m, 1H), 2.64 (bs, 1H), 2.45 (s, 3H), 1.43 (s, 9H), 0.43 (t, J = 7.1 Hz, 3H); **^{13}C-NMR (126 MHz - $CDCl_3$):** δ 168.74, 167.78, 163.60, 143.69, 139.38, 138.94, 135.80, 131.84, 129.26 (2C), 128.69, 127.50 (2C), 126.29, 123.12, 121.14, 120.03, 109.60, 106.46, 81.42, 65.05, 61.86, 57.75, 53.29, 33.57, 33.02, 28.14 (3C, tBu), 21.83, 12.97; **HR-MS:** calc. for $[M+H]^+$, $C_{31}H_{37}O_8N_2S$ = 597.22651, found: 597.22630. $[\alpha]^{RT}_{D}$ = - 94.0° (CH_2Cl_2, c = 1.00); HPLC conditions: CHIRAPAK IC column, DCM:EtOH (100:2)/ *iso*-hexane = 30/70, flow rate = 0.5 mL min^{-1}, minor enantiomer: t_R= 18.40 min; major enantiomer: t_R= 24.21 min, 90% *ee*.

DAD1 A, Sig=254,4 Ref=360,100 (TESSIER\CD447_RAC_30DCM.D)

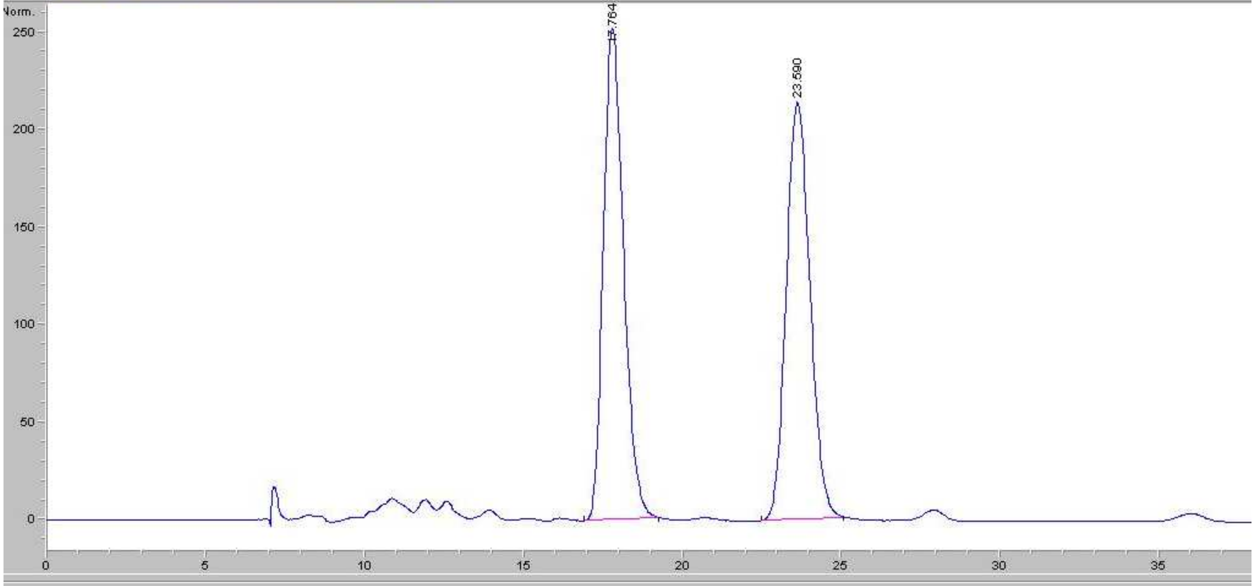

File Information	
LC-File	CD447_RAC_30DCM.D
File Path	C:\HPCHEM\1\DATA\TESSIER\
Date	22-Jan-21, 18:42:11

#	Time	Area	Height	Width	Area%	Symmetry
1	17.764	11183	251.7	0.6884	50.117	0.757
2	23.59	11130.8	214	0.7969	49.883	0.85

DAD1 A, Sig=254,4 Ref=360,100 (TESSIER\CD447_30DCM.D)

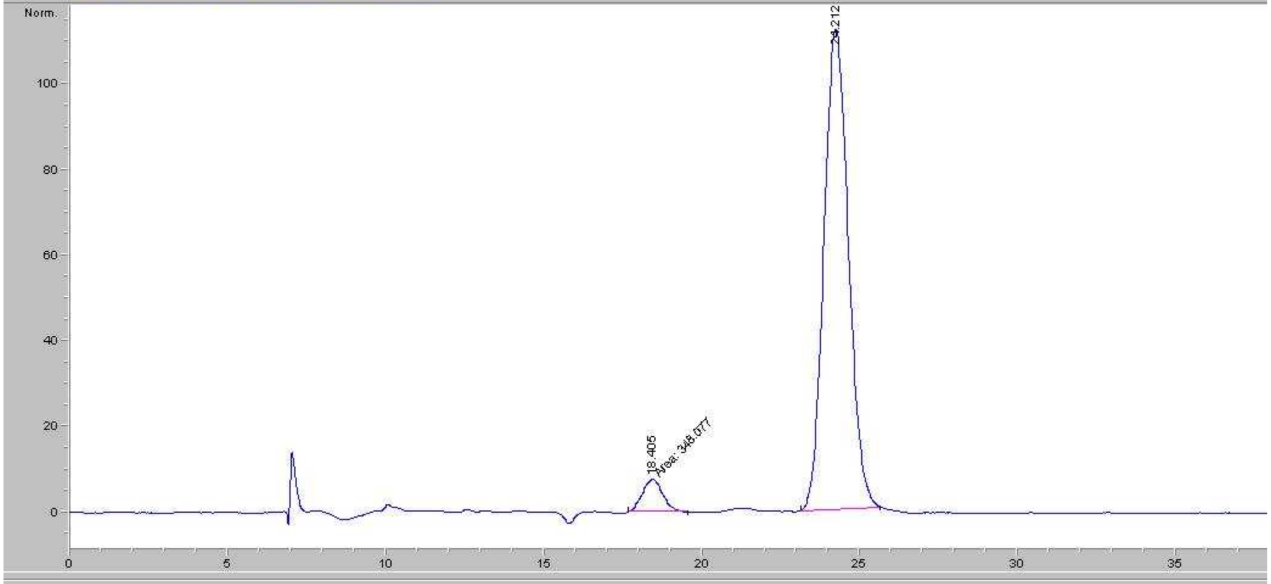

File Information	
LC-File	CD447_30DCM.D
File Path	C:\HPCHEM\1\DATA\TESSIER\

#	Time	Area	Height	Width	Area%	Symmetry
1	18.405	348.1	7.6	0.759	5.431	0.938
2	24.212	6060.8	112.2	0.8263	94.569	0.876

3-(tert-butyl) 2-ethyl (2S,6R)-6-(1-methyl-1H-indol-2-yl)-1-tosyl-1,2,5,6-tetrahydropyridine-2,3-dicarboxylate (3k)

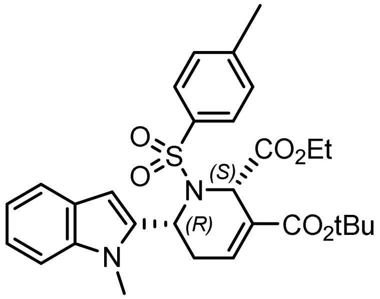

***(S,R)*-3k**

Off-white solid, 68% yield. Pent/EtOAc (5:1 to 3:1) **^{1}H-NMR (700 MHz - $CDCl_3$):** δ 7.80 – 7.77 (m, 2H), 7.44 (d, *J* = 7.8 Hz, 1H), 7.28 (m, *J* = 8.6 Hz, 3H), 7.18 (ddd, *J* = 8.2, 7.0, 1.1 Hz, 1H), 7.02 – 6.99 (m, 1H), 6.81 (m, *J* = 4.4, 1.8 Hz, 1H), 6.25 (s, 1H), 5.57 (d, *J* = 7.5 Hz, 1H), 5.35 (s, 1H), 3.87 (s, 3H), 3.06 – 2.89 (m, 2H), 2.70 (dd, *J* = 19.9, 5.2 Hz, 1H), 2.49 – 2.44 (ddt, 1H), 2.43 (s, 3H), 1.47 (s, 9H), 0.75 (t, *J* = 7.2 Hz, 3H).; **^{13}C-NMR (176 MHz - $CDCl_3$):** δ 167.46, 164.20, 144.33, 138.13, 136.76, 134.70, 134.53, 129.82 (2C), 128.88, 127.68 (2C), 126.45, 122.49, 120.72, 119.58, 109.29, 103.30, 81.33, 61.61, 52.65, 46.41, 30.13, 28.18 (3C), 26.70, 21.75, 13.56; **HR-MS:** calc. for $[M+H]^+$, $C_{29}H_{35}O_6N_2S$ = 539.22103, found: 539.22066. $[\alpha]^{RT}_D$ = - 51.0° (CH_2Cl_2, c = 1.00); HPLC conditions: CHIRAPAK IC column, DCM:EtOH (100:2)/ *iso*-hexane = 30/70, flow rate = 0.5 mL min^{-1}, minor enantiomer: t_R= 32.75 min; major enantiomer: t_R= 24.80 min, 76% *ee*.

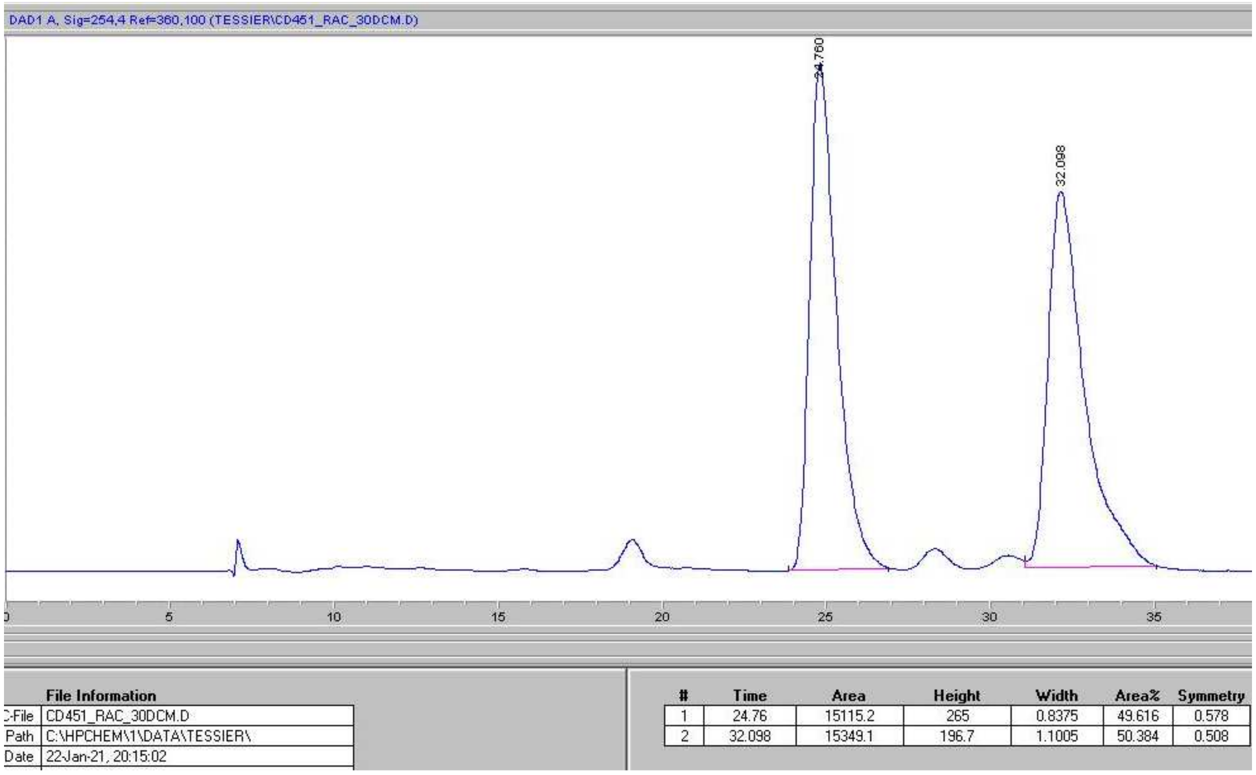

File Information	
C-File	CD451_RAC_30DCM.D
Path	C:\HPCHEM\1\DATA\TESSIER\
Date	22-Jan-21, 20:15:02

#	Time	Area	Height	Width	Area%	Symmetry
1	24.76	15115.2	265	0.8375	49.616	0.578
2	32.098	15349.1	196.7	1.1005	50.384	0.508

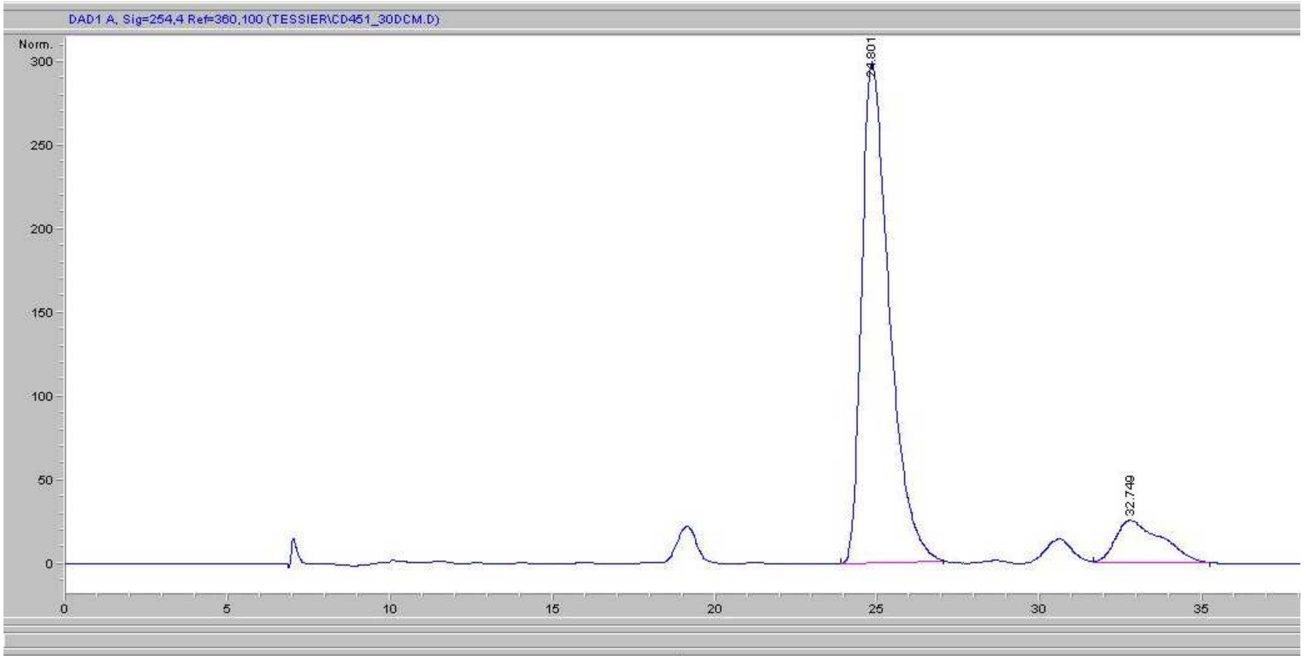

File Information	
LC-File	CD451_30DCM.D
File Path	C:\HPCHEM\1\DATA\TESSIER\
Date	23-Jan-21, 00:07:12

#	Time	Area	Height	Width	Area%	Symmetry
1	24.801	17820.3	299.8	0.8816	87.685	0.546
2	32.749	2502.8	25.4	1.2264	12.315	0.441

3-benzyl 2-(tert-butyl) 6-methyl (2R,6S)-6-(1-methyl-1H-indol-2-yl)-1-((2-nitrophenyl)sulfonyl)-1,2,5,6-tetrahydropyridine-2,3,6-tricarboxylate (3l)

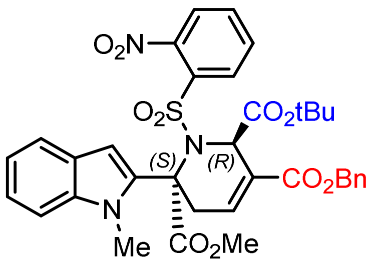

***(R,S)*-3l**

Brown solid, 56% yield. Pent/EtOAc (6:1 to 4:1) **^{1}H-NMR (500 MHz - CDCl$_3$):** δ 8.87 (bs, 1H), 7.79 (t, *J* = 7.5 Hz, 1H), 7.71 (td, *J* = 7.7, 1.3 Hz, 1H), 7.64 (dd, *J* = 7.9, 1.4 Hz, 1H), 7.52 (dd, *J* = 7.9, 1.0 Hz, 1H), 7.42 – 7.30 (m, 5H), 7.25 (d, *J* = 3.4 Hz, 1H), 7.21 – 7.14 (m, 2H), 7.06 (ddd, *J* = 7.9, 5.8, 2.0 Hz, 1H), 6.49 (s, 1H), 6.09 (q, *J* = 1.3 Hz, 1H), 5.20 (dd, 2H), 3.58 (s, 6H), 3.56 – 3.46 (m, 2H), 0.64 (bs, 9H).; **^{13}C-NMR (126 MHz - CDCl$_3$):** 168.55, 166.66, 164.59, 147.82, 139.62, 135.81, 135.48, 133.11, 131.47, 130.66, 130.09, 128.65, 128.59, 128.44, 128.42, 128.24, 126.05, 123.31, 123.26, 120.96, 120.12, 119.41, 110.12, 107.15, 83.12, 66.86, 66.09, 58.03, 53.53, 34.35, 32.85, 26.93 (3C, tBu), 14.17; **HR-MS:** calc. for $[M+H]^+$, $C_{35}H_{36}O_{10}N_3S$ = 690.21159, found: 690.21137. $[\alpha]^{RT}_{D}$ = - 30.0° (CH_2Cl_2, c = 1.00); HPLC conditions: CHIRAPAK IC column, DCM:EtOH (100:2)/*iso*-hexane = 40/60, flow rate = 0.5 mL min^{-1}, minor enantiomer: t_R= 20.36 min; major enantiomer: t_R= 17.05 min, 84% *ee*.

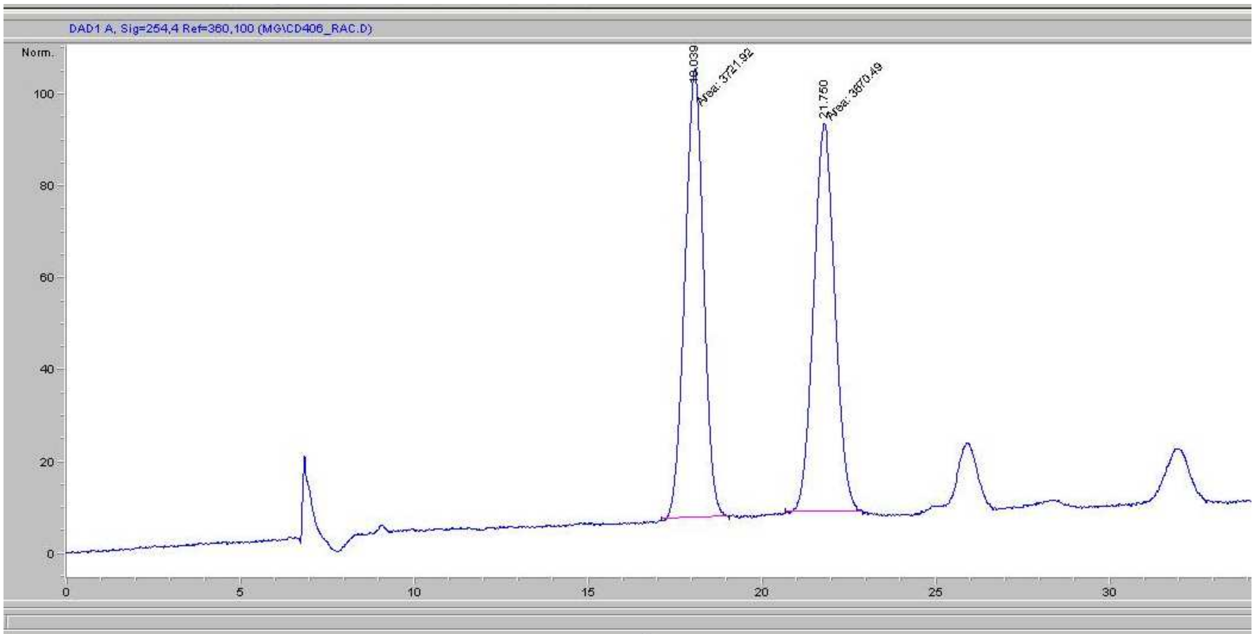

File Information	
LC-File	CD406_RAC.D
File Path	C:\HPCHEM\1\DATA\MG\
Date	20-Nov-20, 22:47:43

#	Time	Area	Height	Width	Area%	Symmetry
1	18.039	3721.9	97.5	0.6361	50.348	1.074
2	21.75	3670.5	84.6	0.7231	49.652	0.89

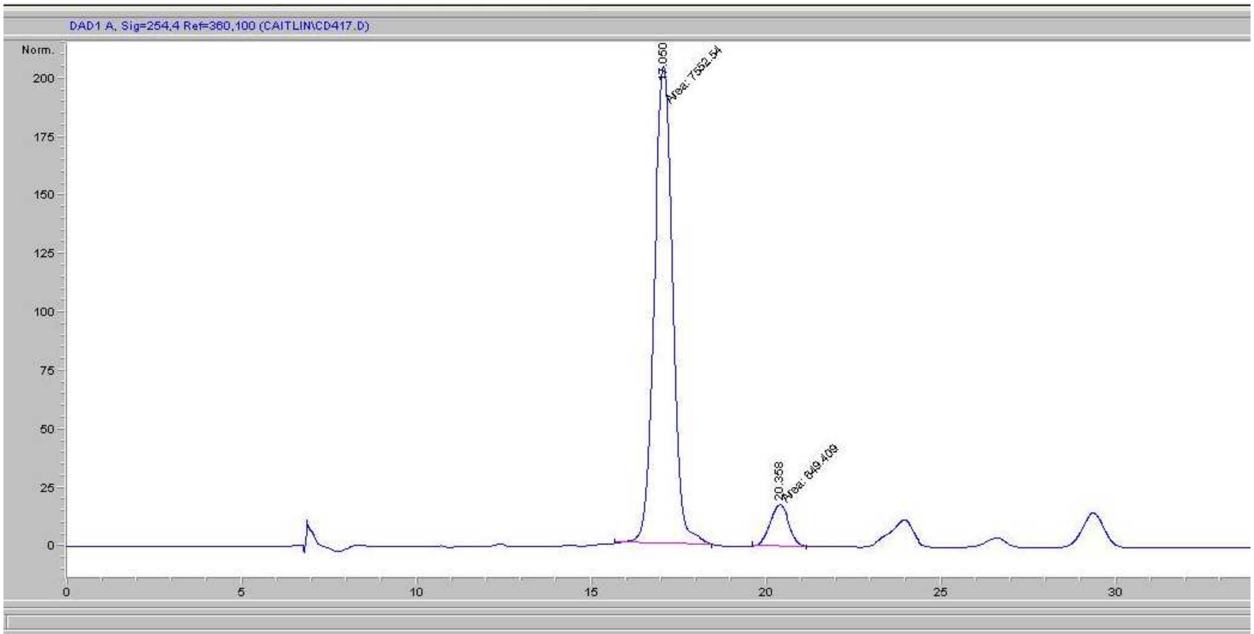

File Information	
LC-File	CD417.D
File Path	C:\HPCHEM\1\DATA\CAITLIN\
Date	05-Oct-20, 14:06:46

#	Time	Area	Height	Width	Area%	Symmetry
1	17.05	7552.5	203.9	0.6174	92.082	1.078
2	20.358	649.4	17.5	0.6181	7.918	0.988

3-benzyl 2-(tert-butyl) 6-methyl (2S,6R)-6-(1-methyl-1H-indol-2-yl)-1-((2-nitrophenyl)sulfonyl)-1,2,5,6-tetrahydropyridine-2,3,6-tricarboxylate (3l)

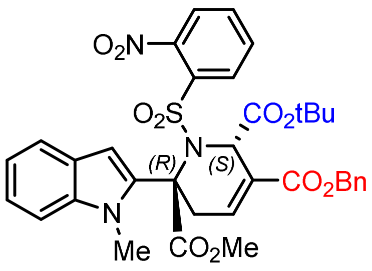

***(S,R)*-3l**

Brown solid, 70% yield. Pent/EtOAc (6:1 to 4:1). **HR-MS:** calc. for $[M+H]^+$, $C_{35}H_{36}O_{10}N_3S$ = 690.21159, found: 690.21131. $[\alpha]^{RT}_{D}$ = + 36.0° (CH_2Cl_2, c = 1.00); HPLC conditions: CHIRAPAK IC column, DCM:EtOH (100:2)/*iso*-hexane = 40/60, flow rate = 0.5 mL min^{-1}, minor enantiomer: t_R= 17.67 min; major enantiomer: t_R= 21.08 min, 82% *ee*.

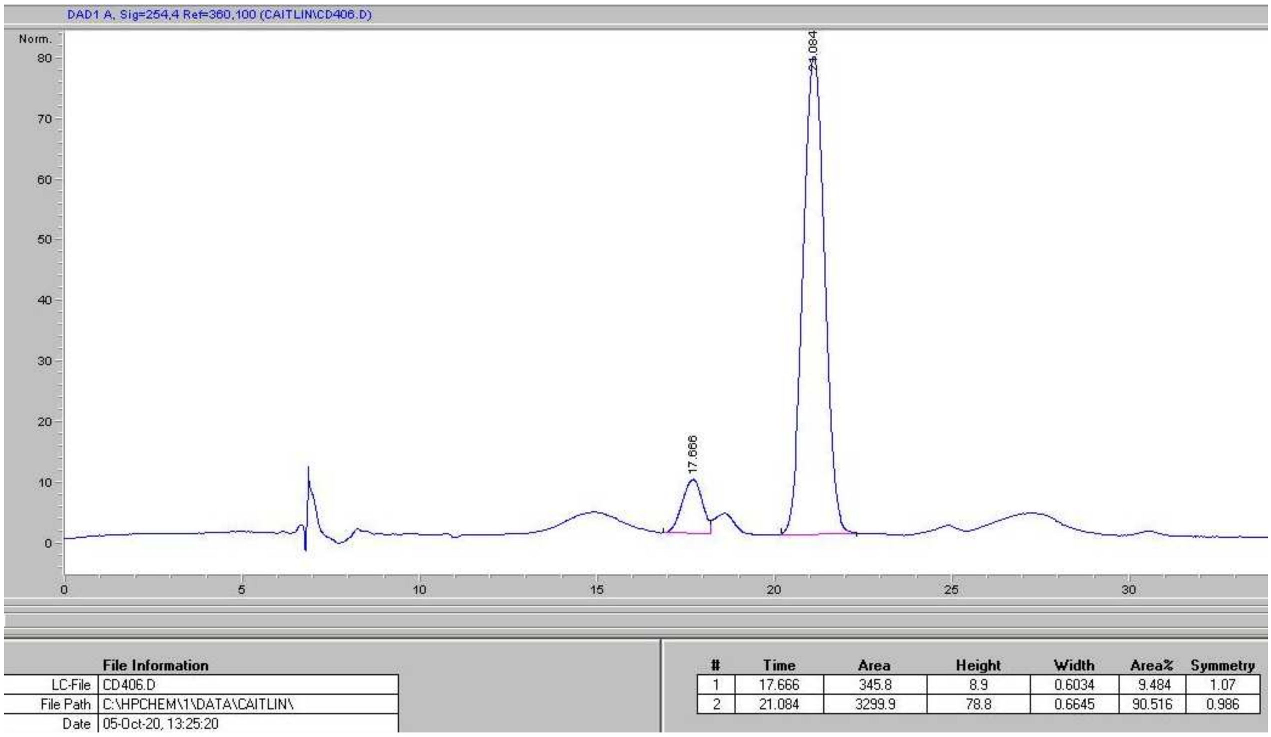

File Information

LC-File	CD406.D
File Path	C:\HPCHEM\1\DATA\CAITLIN\
Date	05-Oct-20, 13:25:20

#	Time	Area	Height	Width	Area%	Symmetry
1	17.666	345.8	8.9	0.6034	9.484	1.07
2	21.084	3299.9	78.8	0.6645	90.516	0.986

3-(tert-butyl) 2-ethyl 6-methyl (2S,6R)-6-(1-methyl-1H-indol-2-yl)-1-((2-nitrophenyl)sulfonyl)-1,2,5,6-tetrahydropyridine-2,3,6-tricarboxylate (3m)

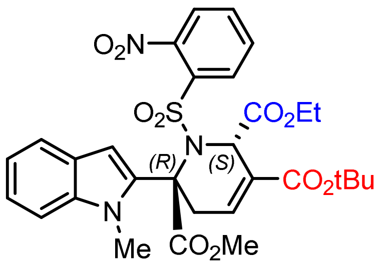

***(S,R)*-3m**

Off-white foam, 68% yield. Pent/EtOAc (5:1 to 4:1) **^{1}H-NMR (500 MHz - $CDCl_3$):** δ 8.89 (bs, 1H), 7.79 (t, 1H), 7.72 (td, J = 7.7, 1.4 Hz, 1H), 7.65 (dd, J = 7.9, 1.4 Hz, 1H), 7.54 (dt, J = 7.9, 1.0 Hz, 1H), 7.25 – 7.15 (m, 3H), 7.07 (ddd, J = 8.0, 6.7, 1.2 Hz, 1H), 6.48 (s, 1H), 5.99 (q, J = 1.3 Hz, 1H), 3.62 (s, 3H), 3.59 – 3.51 (m, 5H), 3.39 (s, 1H), 2.52 (bs, 1H), 1.45 (s, 9H), 0.48 (bs, 3H).; **^{13}C-NMR (126 MHz - $CDCl_3$):** δ 168.54, 167.96, 163.25, 147.90, 139.28, 135.78, 135.47, 133.28, 131.42, 130.65, 130.09, 128.85, 125.97, 123.45, 123.35, 121.14, 120.15, 109.56, 107.43, 81.53, 66.08, 61.89, 57.43, 53.56, 34.05, 32.70, 28.01 (3C, tBu), 12.94; **HR-MS:** calc. for $[M+H]^+$, $C_{30}H_{34}O_{10}N_3S$ = 628.19594, found: 628.19574. $[\alpha]^{RT}_D$ = + 92.0° (CH_2Cl_2, c = 1.00); HPLC conditions: CHIRAPAK IC column, DCM:EtOH (100:2)/*iso*-hexane = 30/70, flow rate = 0.5 mL min^{-1}, minor enantiomer: t_R= 21.52 min; major enantiomer: t_R= 34.92 min, 86% *ee*.

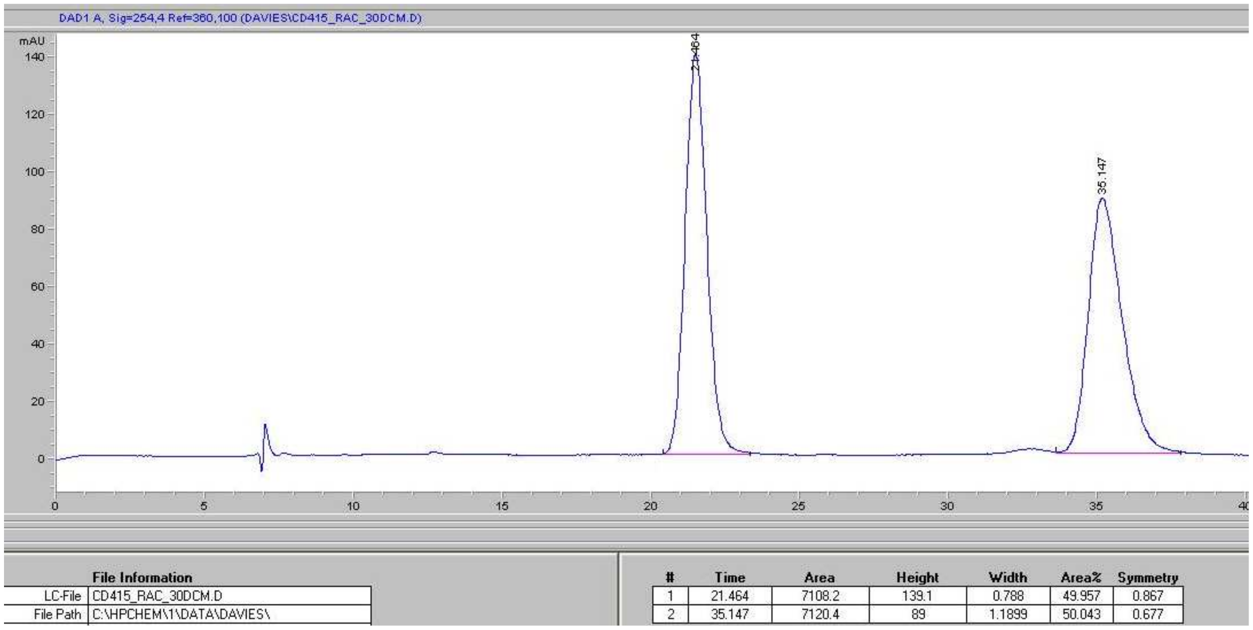

File Information	
LC-File	CD415_RAC_30DCM.D
File Path	C:\HPCHEM\1\DATA\DAVIES\

#	Time	Area	Height	Width	Area%	Symmetry
1	21.464	7108.2	139.1	0.788	49.957	0.867
2	35.147	7120.4	89	1.1899	50.043	0.677

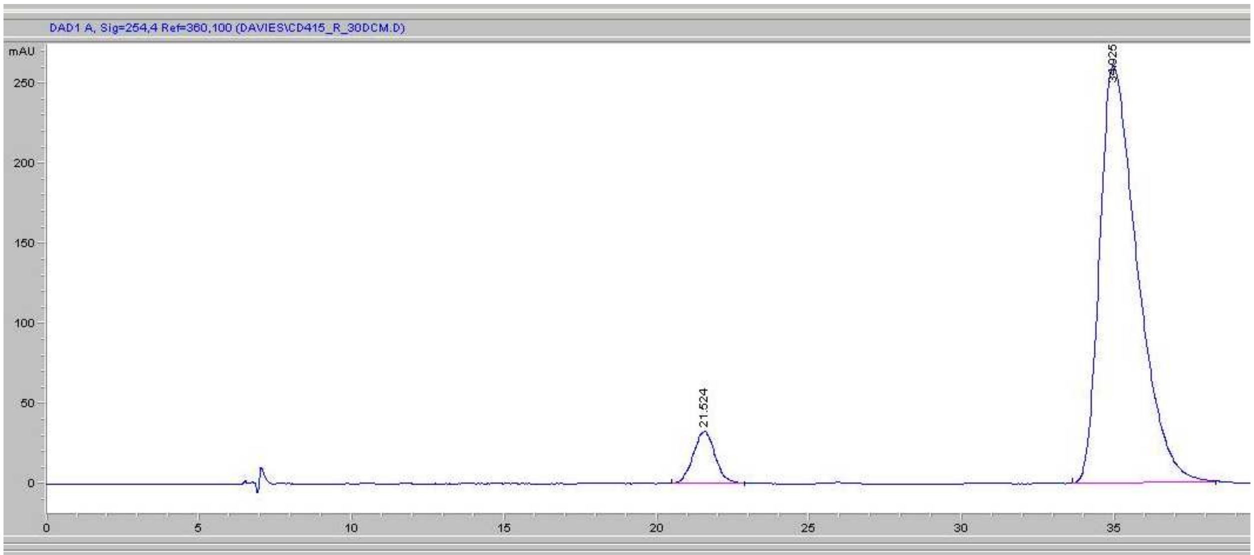

File Information	
LC-File	CD415_R_30DCM.D
File Path	C:\HPCHEM\1\DATA\DAVIES\

#	Time	Area	Height	Width	Area%	Symmetry
1	21.524	1638	32.5	0.7792	6.802	1.003
2	34.925	22443.8	261	1.2754	93.198	0.524

2-benzyl 3-ethyl 6-methyl (2S,6R)-6-(1-methyl-1H-indol-2-yl)-1-((2-nitrophenyl)sulfonyl)-1,2,5,6-tetrahydropyridine-2,3,6-tricarboxylate (3n)

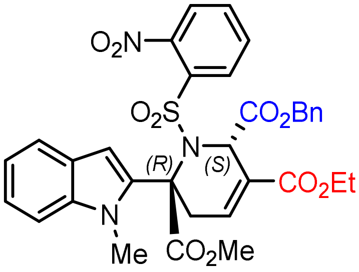

***(S,R)*-3n**

Brown solid, 53 % yield. Pent/EtOAc (6:1 to 4:1) **^{1}H-NMR (500 MHz - $CDCl_3$):** δ 8.86 (s, 1H), 7.78 – 7.71 (m, 2H), 7.66 (dd, 1H), 7.47 (d, 1H), 7.30 – 7.26 (m, 1H), 7.25 – 7.17 (m, 5H), 7.06 (ddd, J = 7.9, 5.0, 3.0 Hz, 1H), 6.78 (d, J = 6.8 Hz, 2H), 6.51 (s, 1H), 6.11 (q, J = 1.3 Hz, 1H), 4.47 (d, J = 12.1 Hz, 1H), 4.05 – 3.94 (m, 1H), 3.92 – 3.81 (m, 1H), 3.63 (s, 3H), 3.59 – 3.56 (m, 5H), 3.08 (s, 1H), 1.04 (t, J = 7.1 Hz, 3H).; **^{13}C-NMR (126 MHz - $CDCl_3$):** δ 168.41, 167.28, 164.12, 147.87, 139.34, 136.58, 135.55, 134.37, 133.31, 131.46, 130.46, 129.95, 128.21, 128.19 (2C), 128.08 (2C), 125.90, 127.17, 123.62, 123.43, 121.33, 120.28, 109.62, 107.62, 67.20, 66.12, 61.10, 57.36,

53.62, 34.03, 32.72, 13.89; **HR-MS:** calc. for $[M+H]^+$, $C_{33}H_{32}O_{10}N_3S$ = 662.18029, found: 662.18000.

$[\alpha]^{RT}_{D}$ = + 46.0° (CH_2Cl_2, c = 1.00); HPLC conditions: CHIRAPAK IC column, DCM:EtOH (100:2)/ *iso*-hexane = 40/60, flow rate = 0.5 mL min^{-1}, minor enantiomer: t_R= 17.28 min; major enantiomer: t_R= 25.65 min, 76% *ee*.

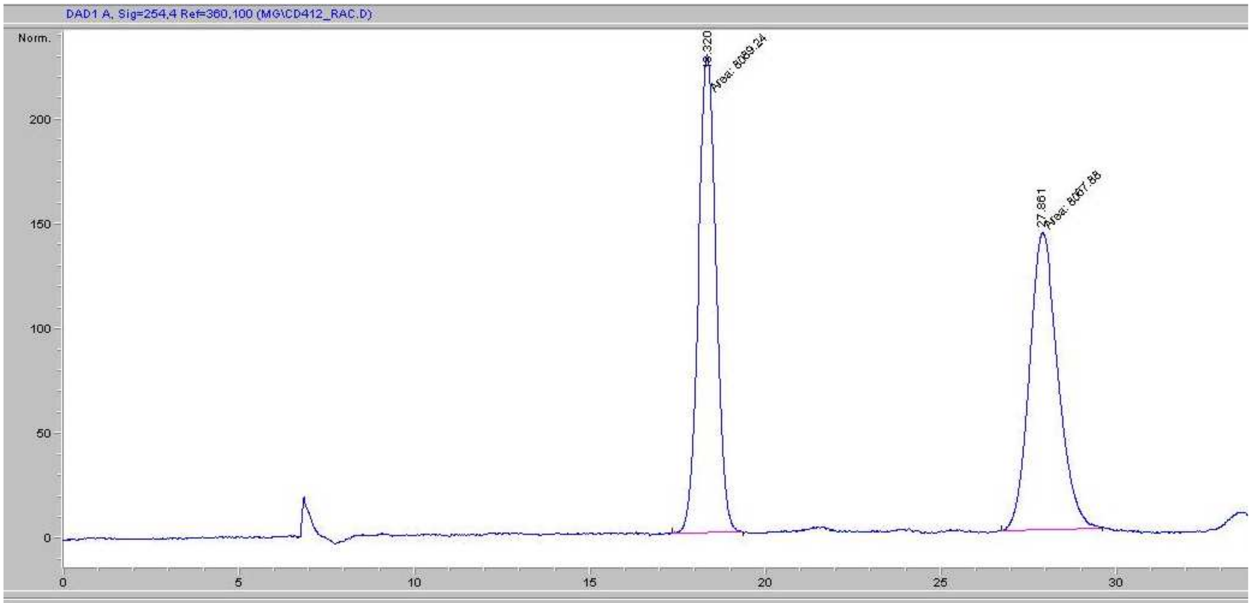

File Information	
LC-File	CD412_RAC.D
File Path	C:\HPCHEM\1\DATA\MG\
Date	21-Nov-20, 00:10:34

#	Time	Area	Height	Width	Area%	Symmetry
1	18.32	8089.2	228.5	0.5901	50.066	1.024
2	27.861	8067.9	142.2	0.9458	49.934	0.792

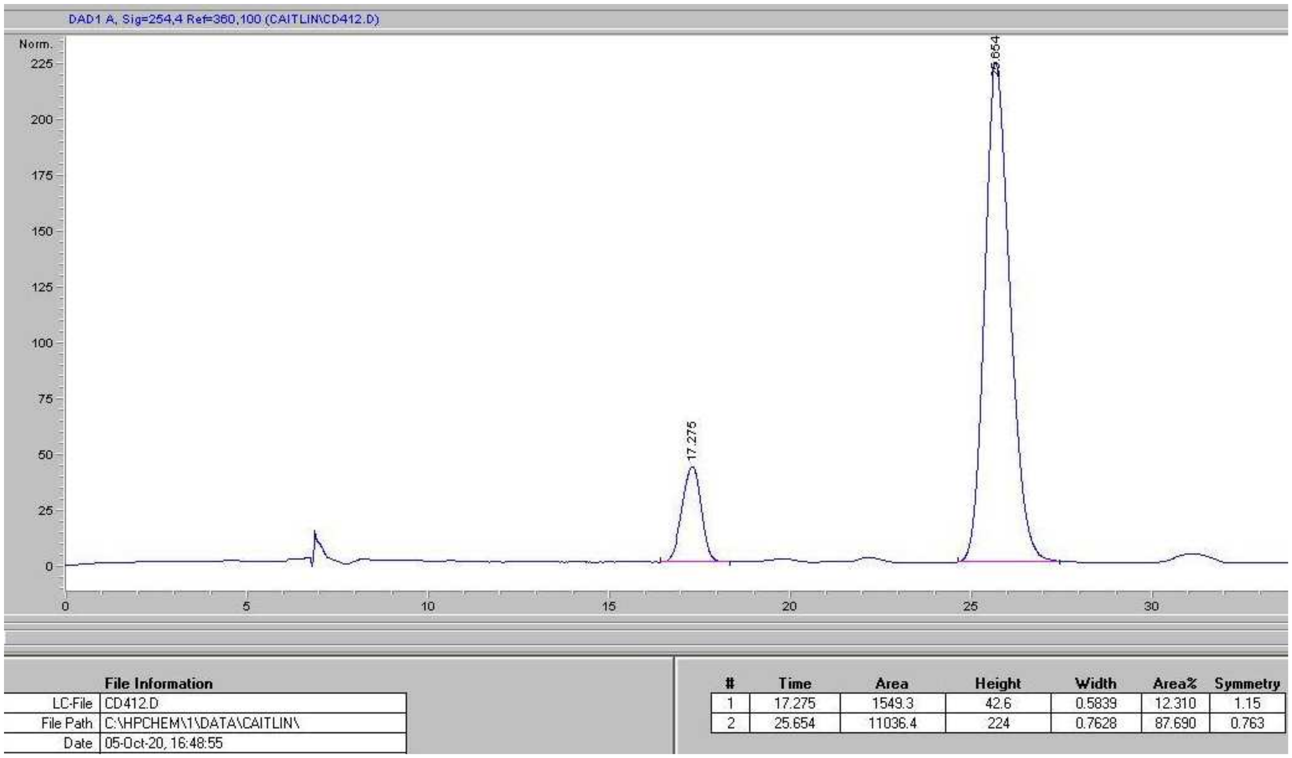

File Information

LC-File	CD412.D
File Path	C:\HPCHEM\1\DATA\CAITLIN\
Date	05-Oct-20, 16:48:55

#	Time	Area	Height	Width	Area%	Symmetry
1	17.275	1549.3	42.6	0.5839	12.310	1.15
2	25.654	11036.4	224	0.7628	87.690	0.763

2,3-di-tert-butyl 6-methyl (2R,6S)-6-(1-methyl-1H-indol-2-yl)-1-((2-nitrophenyl)sulfonyl)-1,2,5,6-tetrahydropyridine-2,3,6-tricarboxylate (3o)

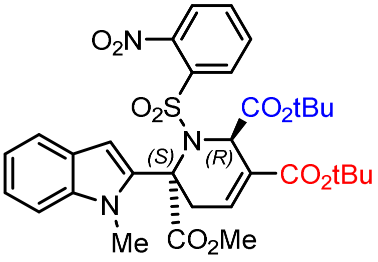

***(R,S)*-3o**

Off-white solid, 62% yield. Pent/EtOAc (5:1 to 4:1) **^{1}H-NMR (500 MHz - $CDCl_3$):** δ 8.80 (bs, 1H), 7.79 – 7.72 (m, 1H), 7.69 (td, *J* = 7.7, 1.4 Hz, 1H), 7.62 (dd, *J* = 7.9, 1.4 Hz, 1H), 7.54 (dd, *J* = 7.9, 1.0 Hz, 1H), 7.23 – 7.16 (m, 2H), 7.12 (t, 1H), 7.08 (ddd, *J* = 7.9, 5.3, 2.7 Hz, 1H), 6.49 (s, 1H), 6.01 (d, *J* = 1.4 Hz, 1H), 3.60 (d, *J* = 10.8 Hz, 3H), 3.57 (s, 3H), 3.52 (d, *J* = 5.0 Hz, 1H), 3.45 (d, *J* = 19.8 Hz, 1H), 1.52 (s, 9H), 0.79 (bs, 9H); **^{13}C-NMR (126 MHz - $CDCl_3$):** δ 169.20, 167.66, 164.04, 148.20, 140.00, 136.14, 135.25, 133.40, 131.76, 131.44, 130.62, 126.46, 123.61, 123.56, 121.31, 120.45, 110.50, 107.43, 83.28, 81.82, 66.57, 58.47, 53.82, 34.97, 33.22, 28.56 (3C, tBu), 27.51 (3C, tBu), 14.54; **HR-MS:** calc. for $[M+H]^+$, $C_{32}H_{38}O_{10}N_3S$ = 656.22724, found: 656.22702. $[\alpha]^{RT}_{D}$ = - 88.0° (CH_2Cl_2, c = 1.00); HPLC conditions: CHIRAPAK IC column, DCM:EtOH

(100:2)/*iso*-hexane = 40/60, flow rate = 0.5 mL min^{-1}, minor enantiomer: t_R= 13.79 min; major enantiomer: t_R= 9.31 min, 86% *ee*.

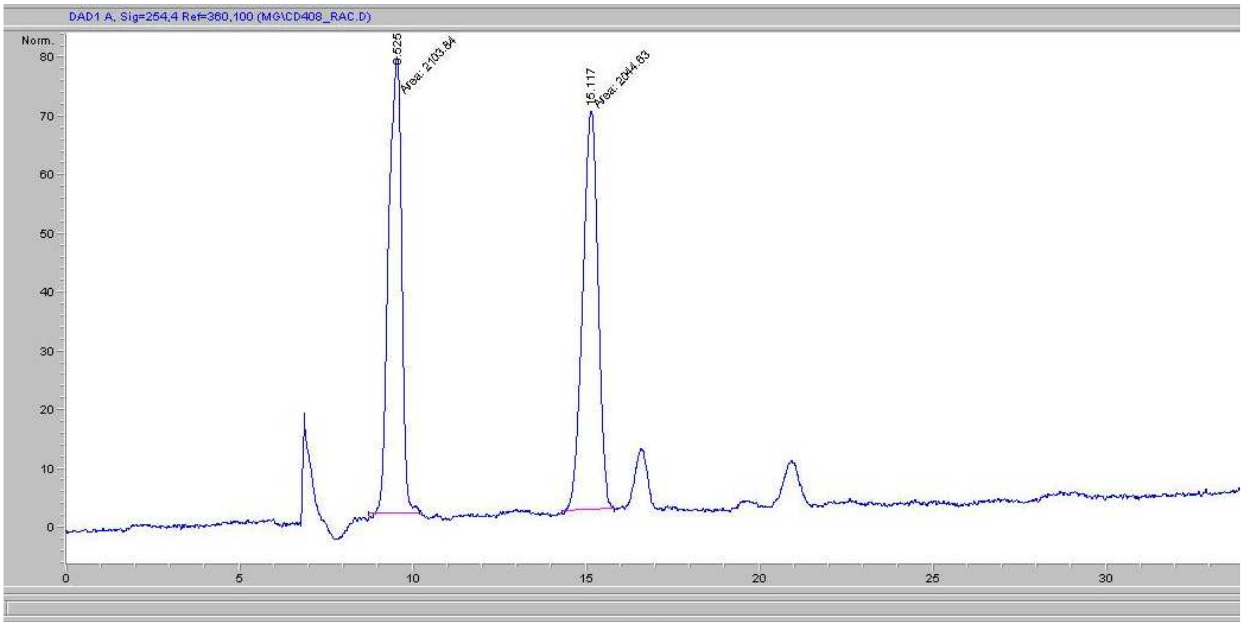

File Information	
LC-File	CD408_RAC.D
File Path	C:\HPCHEM\1\DATA\MG\
Date	20-Nov-20, 23:29:08

#	Time	Area	Height	Width	Area%	Symmetry
1	9.525	2103.8	77.8	0.4504	50.714	1.823
2	15.117	2044.6	68	0.5013	49.286	1.283

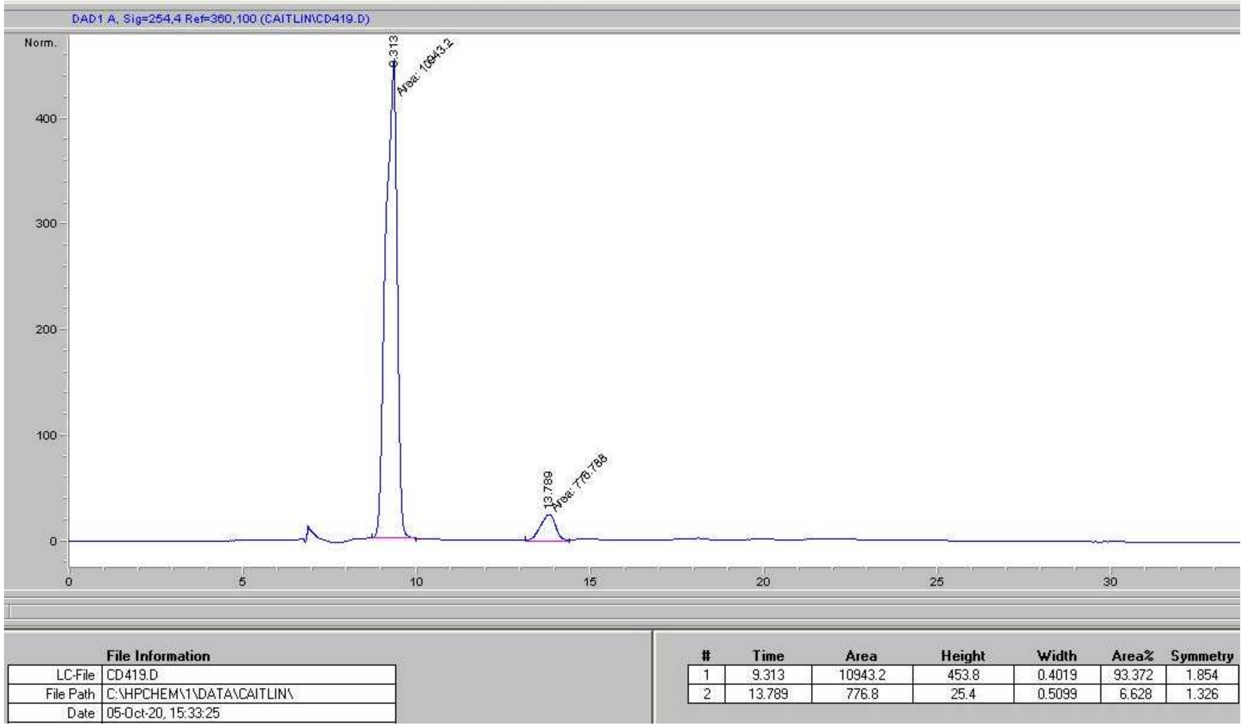

File Information	
LC-File	CD419.D
File Path	C:\HPCHEM\1\DATA\CAITLIN\
Date	05-Oct-20, 15:33:25

#	Time	Area	Height	Width	Area%	Symmetry
1	9.313	10943.2	453.8	0.4019	93.372	1.854
2	13.789	776.8	25.4	0.5099	6.628	1.326

2,3-di-tert-butyl 6-methyl (2S,6R)-6-(1-methyl-1H-indol-2-yl)-1-((2-nitrophenyl)sulfonyl)-1,2,5,6-tetrahydropyridine-2,3,6-tricarboxylate (3o)

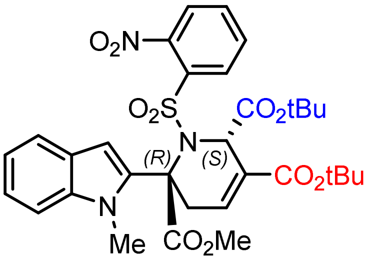

***(S,R)*-3o**

Off-white solid, 71% yield. Pent/EtOAc (5:1 to 4:1). **HR-MS:** calc. for $[M+H]^+$, $C_{32}H_{38}O_{10}N_3S$ = 656.22724, found: 656.22706. $[\alpha]^{RT}_{D}$ = + 83.0° (CH_2Cl_2, c = 1.00); HPLC conditions: CHIRAPAK IC column, DCM:EtOH (100:2)/ *iso*-hexane = 40/60, flow rate = 0.5 mL min^{-1}, minor enantiomer: t_R= 9.39 min; major enantiomer: t_R= 13.96 min, 90% *ee*.

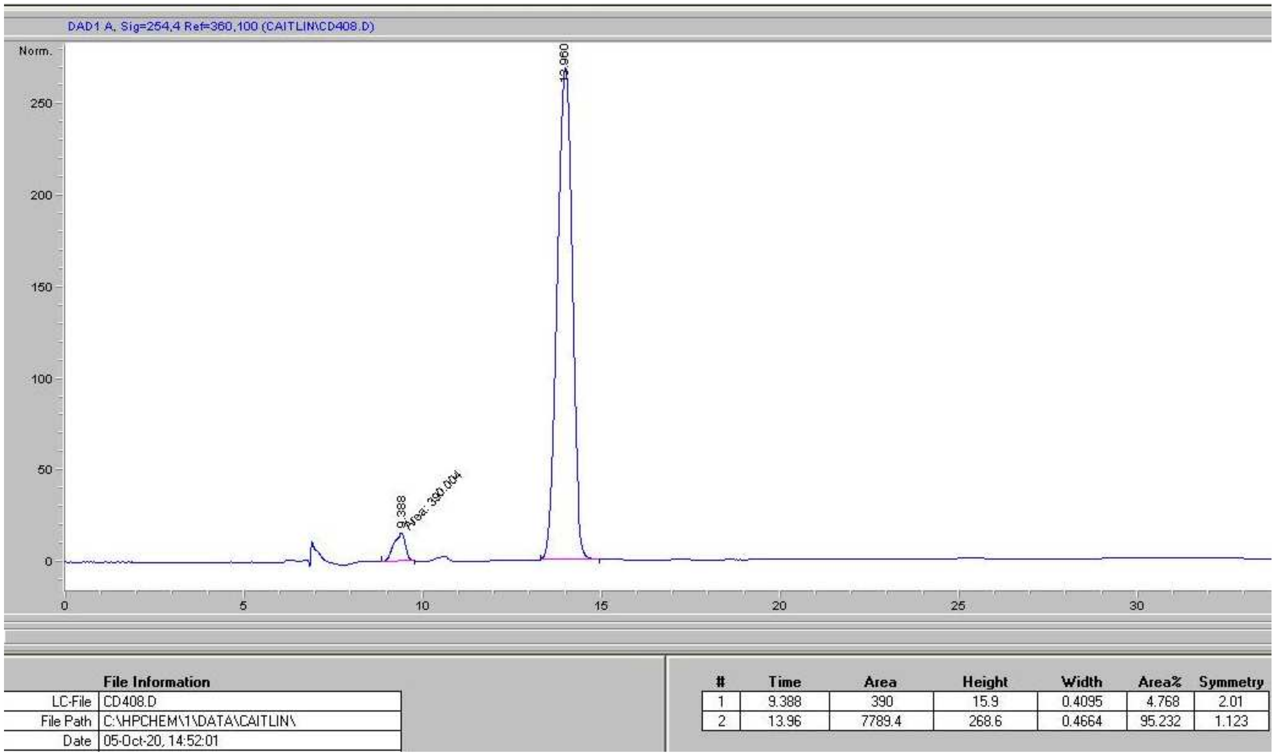

File Information	
LC-File	CD408.D
File Path	C:\HPCHEM\1\DATA\CAITLIN\
Date	05-Oct-20, 14:52:01

#	Time	Area	Height	Width	Area%	Symmetry
1	9.388	390	15.9	0.4095	4.768	2.01
2	13.96	7789.4	268.6	0.4664	95.232	1.123

3-benzyl 2-(tert-butyl) 6-methyl (2S,6R)-6-(1-methyl-1H-indol-2-yl)-1-tosyl-1,2,5,6-tetrahydropyridine-2,3,6-tricarboxylate (3p)

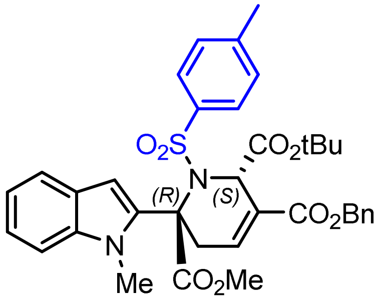

***(S,R)*-3p**

Off-white foam, 58% yield. Pent/EtOAc (7:1) **^{1}H-NMR (600 MHz - $CDCl_3$):** δ 7.87 (bs, 2H), 7.52 – 7.48 (m, 1H), 7.42 – 7.31 (m, 6H), 7.27 (m, 1H), 7.18 – 7.11 (m, 3H), 7.04 (ddd, *J* = 7.9, 6.5, 1.4 Hz, 1H), 6.44 (d, *J* = 0.7 Hz, 1H), 5.93 (q, *J* = 1.4 Hz, 1H), 5.25 – 5.10 (m, 2H), 3.64 (s, 3H), 3.59 (s, 3H), 3.50 (ddd, *J* = 19.5, 5.2, 1.0 Hz, 1H), 3.43 (d, 1H), 2.43 (s, 3H), 0.72 (s, 9H). **^{13}C-NMR (151 MHz - $CDCl_3$):** δ 168.86, 166.60, 164.69, 143.38, 139.49, 138.83, 135.66, 135.62, 131.68, 129.08 (2C), 128.62 (2C),128.42 (2C), 128.38, 128.15, 127.39 (2C), 126.17, 122.77, 120.82, 119.84, 110.05, 106.11, 82.51, 66.72, 65.06, 58.38, 53.13, 33.03, 27.49, 27.08 (3C), 21.65; **HR-MS:** calc. for $[M+H]^+$, $C_{36}H_{39}O_8N_2S$ = 659.24216, found: 659.24406. $[\alpha]^{RT}_{D}$ = - 74.0° (CH_2Cl_2, c = 1.00); HPLC conditions: CHIRAPAK IA column, DCM:EtOH (100:5)/ *iso*-hexane = 15/85, flow rate = 0.5 mL min^{-1}, minor enantiomer: t_R= 20.70 min; major enantiomer: t_R= 27.70 min, 87% *ee*.

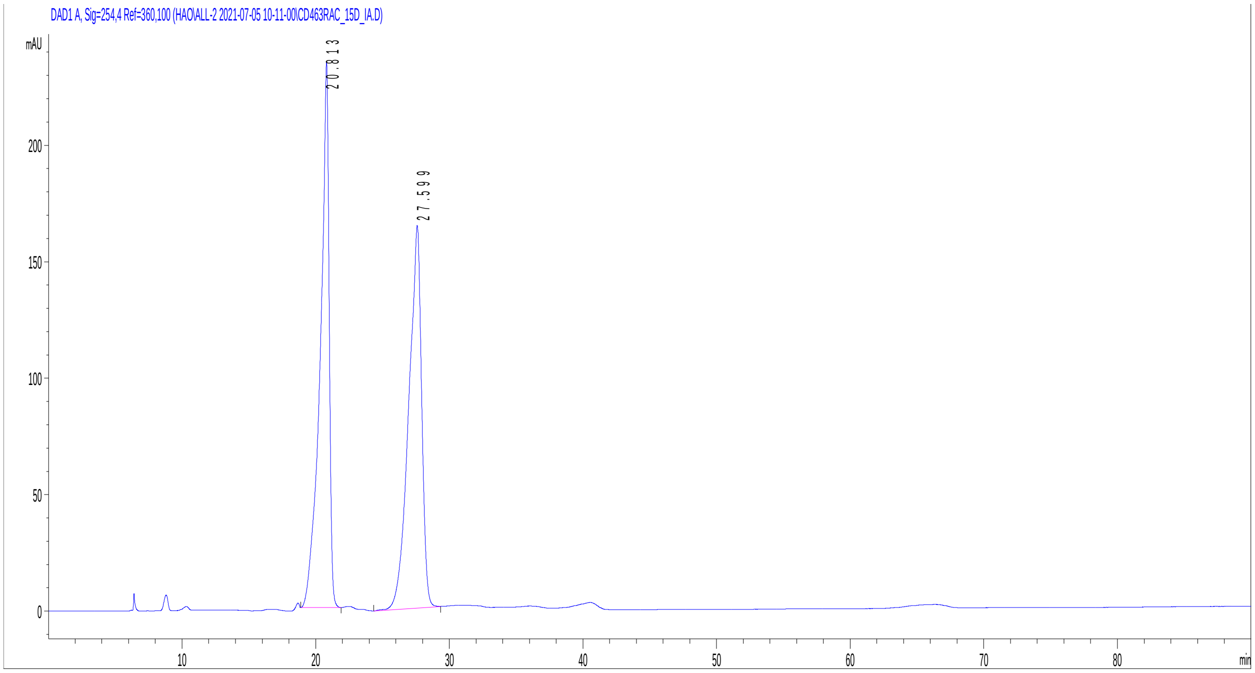

#	Time	Area	Height	Width	Area%	Symmetry
1	20.813	10864.7	234.4	0.6267	49.611	2.355
2	27.599	11035.3	164.2	0.9099	50.389	1.865

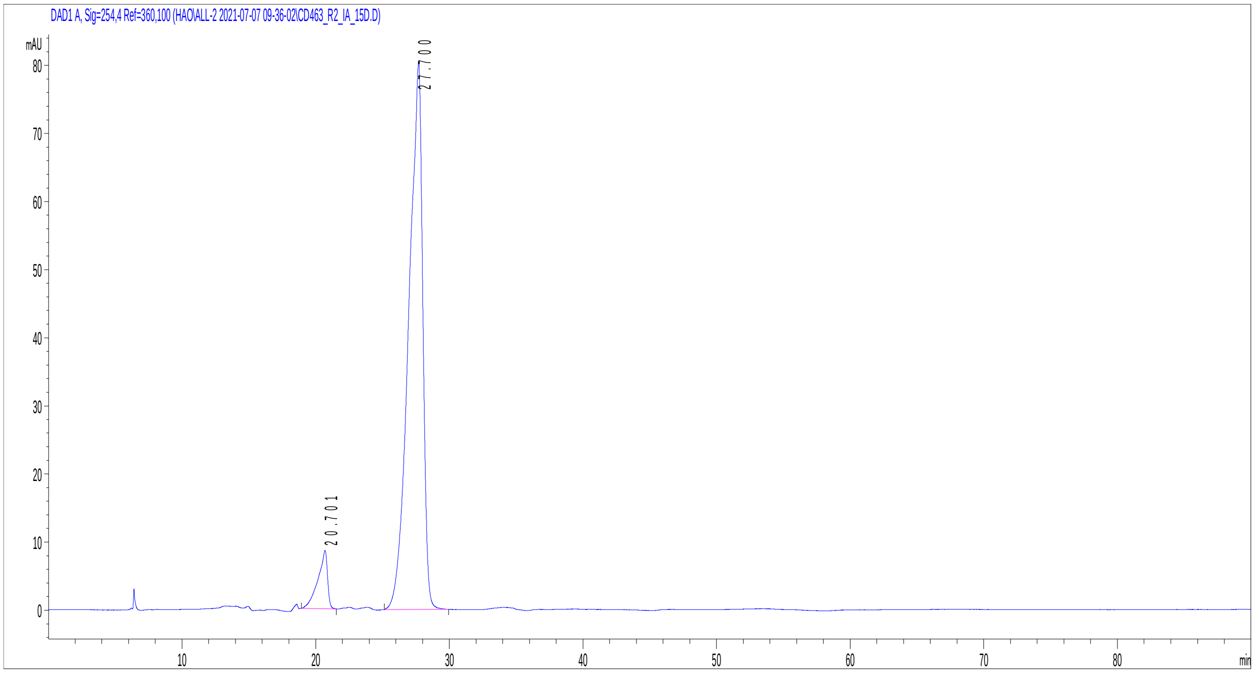

#	Time	Area	Height	Width	Area%	Symmetry
1	20.701	428.2	8.6	0.6686	6.847	2.802
2	27.7	5825.9	80.4	0.9694	93.153	2.209

2-(tert-butyl) 3-ethyl 6-methyl (2S,6R)-1-((4-fluorophenyl)sulfonyl)-6-(1-methyl-1H-indol-2-yl)-1,2,5,6-tetrahydropyridine-2,3,6-tricarboxylate (3q)

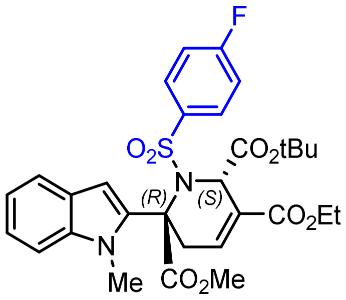

***(S,R)*-3q**

Orange foam, 84% yield. Pent/EtOAc (7:1 to 6:1) **^{1}H-NMR (600 MHz - $CDCl_3$):** δ 8.00 (bs, 2H), 7.52 (d, *J* = 7.9, 1.0 Hz, 1H), 7.20 – 7.10 (m, 5H), 7.06 (ddd, *J* = 7.9, 6.8, 1.1 Hz, 1H), 6.47 (d, *J* = 0.7 Hz, 1H), 5.95 (s, 1H), 4.22 (ddq, *J* = 56.7, 10.9, 7.2 Hz, 2H), 3.66 (s, 3H), 3.54 (dd, *J* = 5.2, 1.0 Hz, 1H), 3.54 (s, 3H), 3.44 – 3.36 (m, 1H), 1.33 (t, *J* = 7.1 Hz, 3H), 0.82 (s, 9H); **^{13}C-NMR (126 MHz - $CDCl_3$):** δ 167.90 (d, *J* = 303.8 Hz), 165.79, 164.80, 164.10, 139.40, 137.93, 135.07, 131.41, 130.25, 130.19, 128.82, 126.16, 122.93, 120.87, 119.97, 115.61, 115.46, 110.04, 106.39, 82.64, 65.31, 61.09, 58.50, 53.19, 34.14, 32.79, 27.18 (3C, tBu), 14.30; **^{19}F NMR (565 MHz, $CDCl_3$)** δ -105.42; **HR-MS:** calc. for $[M+H]^+$, $C_{30}H_{34}O_8N_2FS$ = 601.20144, found: 601.20327. $[\alpha]^{RT}_{D}$ = - 70.0° (CH_2Cl_2, c = 1.00); HPLC conditions: CHIRAPAK IA column, DCM:EtOH (100:2)/ *iso*-hexane = 10/90, flow rate = 0.5 mL min^{-1}, minor enantiomer: t_R= 25.39 min; major enantiomer: t_R= 32.57 min, 84% *ee*.

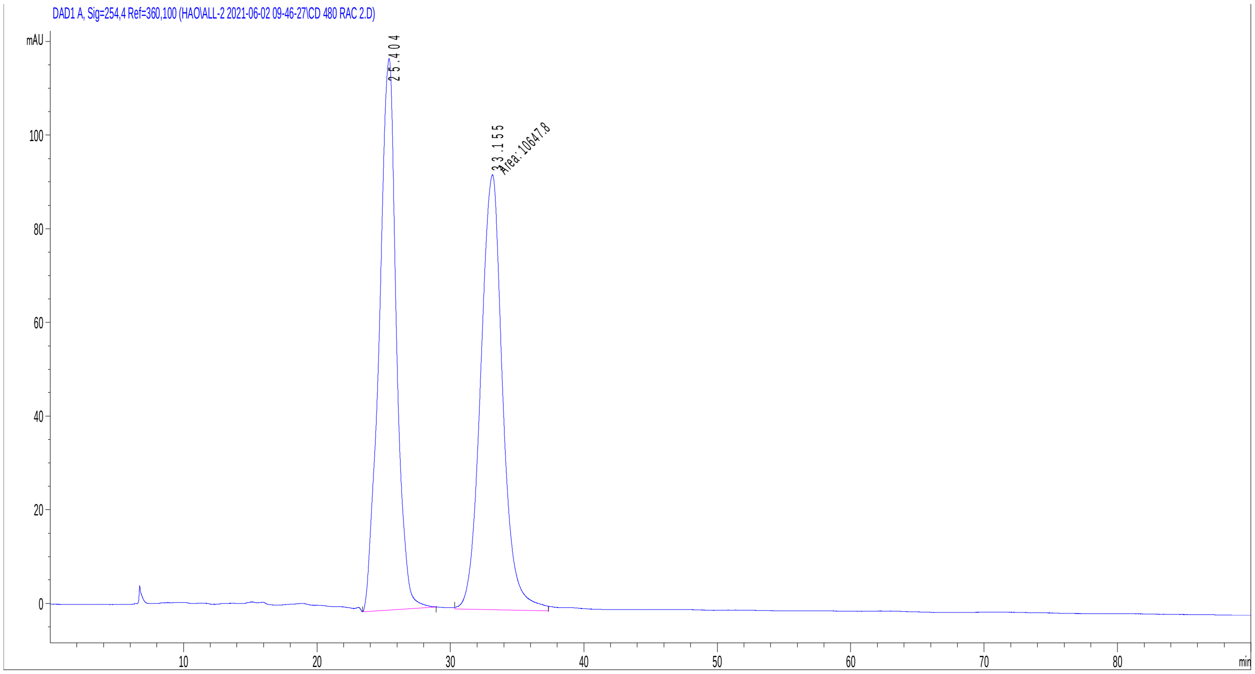

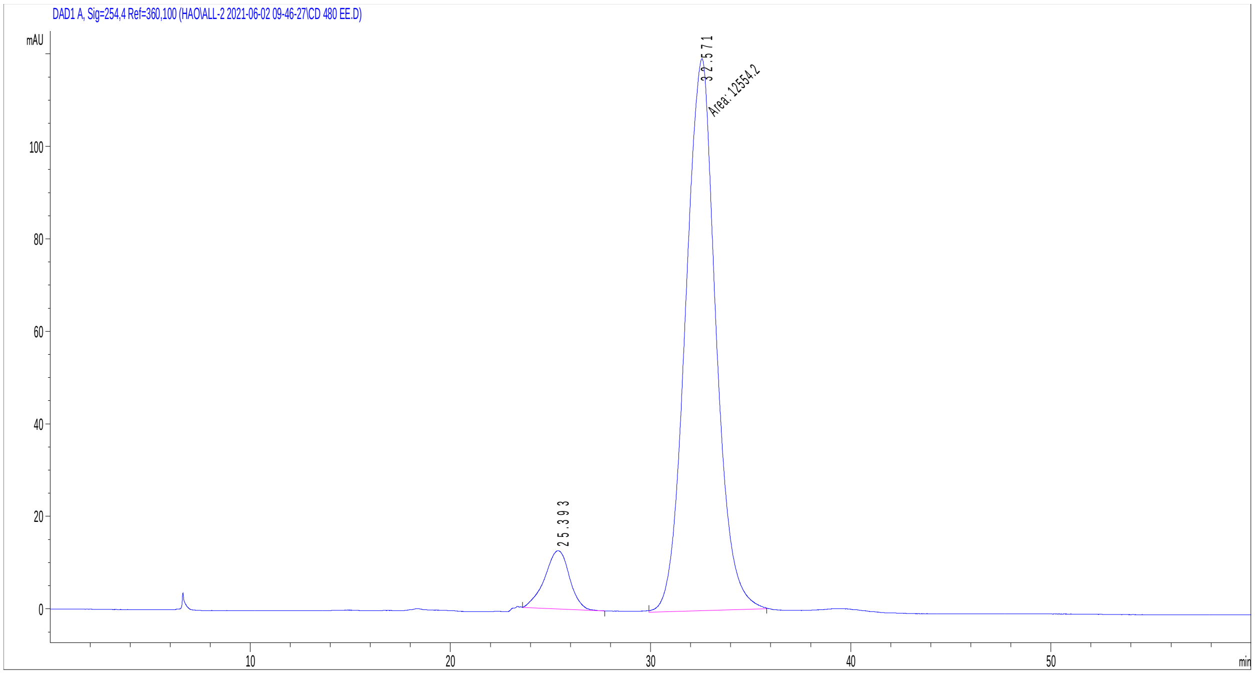

#	Time	Area	Height	Width	Area%	Symmetry
1	25.393	1085.5	12.6	1.0223	7.958	1.19
2	32.571	12554.2	119.4	1.7529	92.042	1.115

3-benzyl 2-(tert-butyl) (2S,6R)-6-(1-methyl-1H-indol-2-yl)-1-((2-nitrophenyl)sulfonyl)-1,2,5,6-tetrahydropyridine-2,3-dicarboxylate (3r)

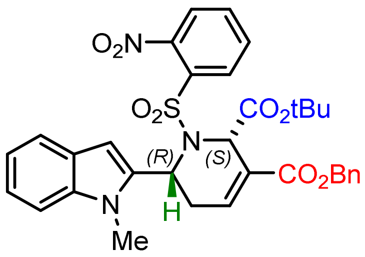

***(S,R)*-3r**

Off-white foam, 54% yield. Pent/EtOAc (6:1 to 5:1) **^{1}H-NMR (500 MHz - $CDCl_3$):** δ 7.90 (dd, *J* = 8.0, 1.3 Hz, 1H), 7.68 (td, *J* = 7.8, 1.3 Hz, 1H), 7.53 (dd, *J* = 8.0, 1.2 Hz, 1H), 7.49 – 7.43 (m, 2H), 7.42 – 7.34 (m, 5H), 7.29 (dd, *J* = 8.5, 0.9 Hz, 1H), 7.17 (ddd, *J* = 8.3, 7.0, 1.2 Hz, 1H), 7.01 (ddd, *J* = 8.1, 7.1, 1.0 Hz, 1H), 6.98 (m, 1H), 6.32 (s, 1H), 5.65 (d, *J* = 7.2 Hz, 1H), 5.60 (m, 1H), 5.26 – 5.18 (m, 2H), 3.87 (s, 3H), 2.87 – 2.79 (m, 1H), 2.67 (ddt, *J* = 20.1, 7.4, 2.5 Hz, 1H), 0.72 (s, 9H); **^{13}C-NMR (126 MHz - $CDCl_3$):** δ 165.41, 165.03, 148.26, 138.09, 135.65, 135.38, 134.15, 133.89, 132.64, 131.65, 130.45, 128.66 (2C), 128.50 (2C), 128.48, 127.53, 126.63, 123.79, 122.41, 120.79, 119.51, 109.45, 103.33, 82.85, 66.84, 54.03, 46.75, 30.15, 27.49, 27.09 (3C); **HR-MS:** calc. for $[M+H]^+$, $C_{33}H_{34}O_8N_3S$ = 632.20611, found: 632.20789. $[\alpha]^{RT}_{D}$ = - 41.0° (CH_2Cl_2, c = 1.00); HPLC conditions: CHIRAPAK IC column, DCM:EtOH (100:2)/ *iso*-hexane = 30/70, flow rate = 0.5 mL min^{-1}, minor enantiomer: t_R= 26.47 min; major enantiomer: t_R= 38.06 min, 76% *ee*.

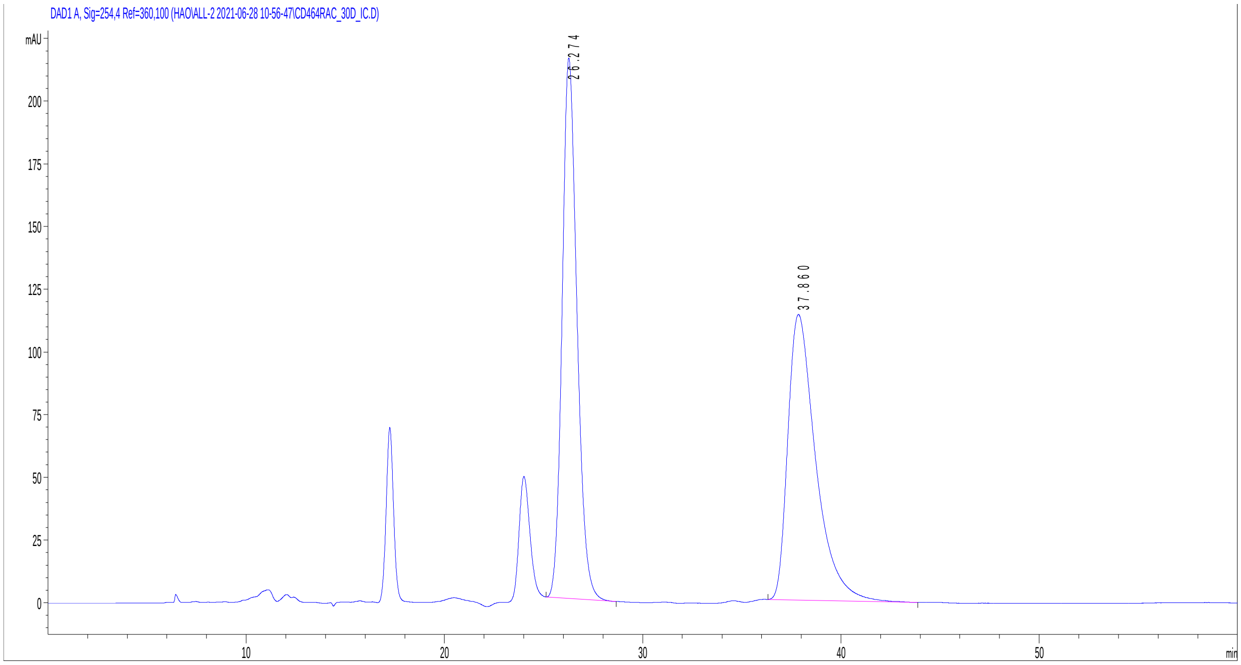

#	Time	Area	Height	Width	Area%	Symmetry
1	26.274	11218.6	215.5	0.7972	50.195	0.774
2	37.86	11131.4	113.8	1.4489	49.805	0.572

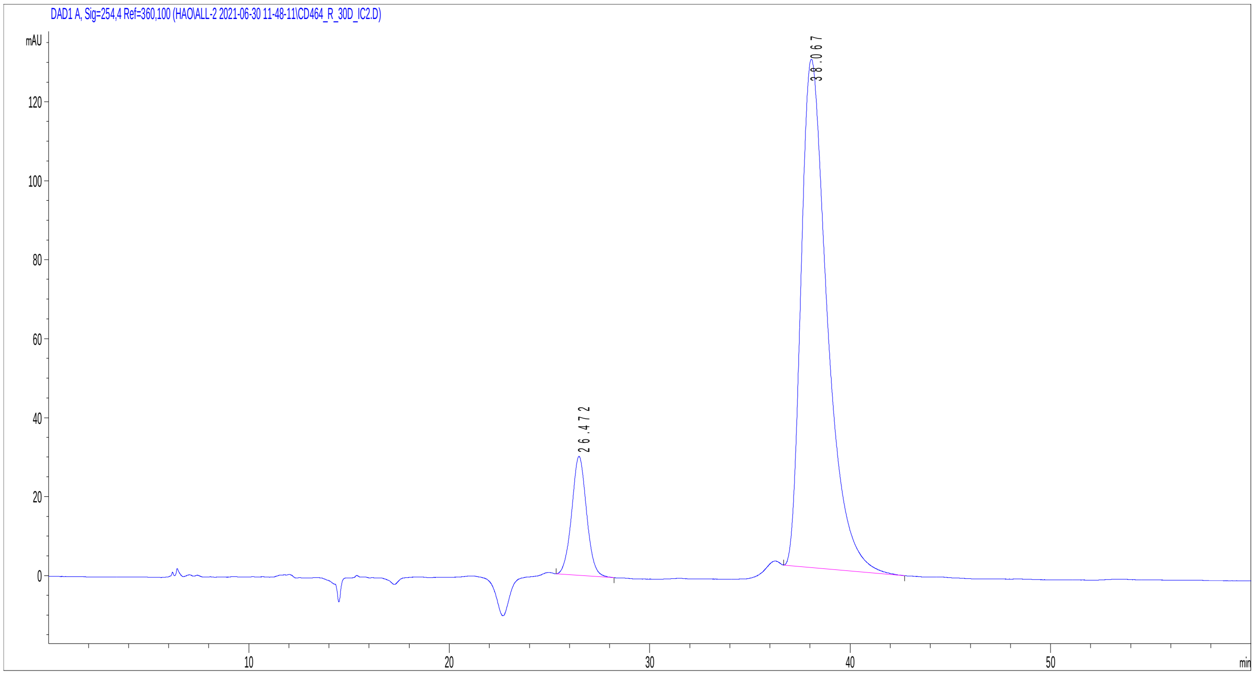

#	Time	Area	Height	Width	Area%	Symmetry
1	26.472	1550.4	30.2	0.7697	11.672	0.921
2	38.067	11732.2	128.8	1.3589	88.328	0.594

3. X-Ray Structure Analyses

The crystal structure of compound *(R,S)*-**3b** was determined using the *Bruker D8 Venture* four-circle diffractometer equipped with a *PHOTON II* CPAD detector by *Bruker AXS GmbH*. The X-ray radiation was generated by the *IµS/IµS* microfocus source Cu (λ = 1.54178 Å) from *Incoatec GmbH* equipped with HELIOS mirror optics and a single-hole collimator by *Bruker AXS GmbH*. The selected single crystal of *(R,S)*-**3b** was covered with an inert oil (perfluoropolyalkyl ether) and mounted on the *MicroMount* from *MiTeGen*. The APEX 3 Suite (v.2018.7-2) software integrated with SAINT (integration) and SADABS (adsorption correction) programs by *Bruker AXS GmbH* were used for data collection. The processing and finalization of the crystal structure were performed using the Olex2 program[10]. The crystal structures were solved by the ShelXT[11] structure solution program using the Intrinsic Phasing option, which were further refined by the ShelXL[12] refinement package using Least Squares minimization. The non-hydrogen atoms were anisotropically refined. The C-bound H atoms were placed in geometrically calculated positions, and a fixed isotropic displacement parameter was assigned to each atom according to the riding-model: C–H = 0.95–1.00 Å with Uiso(H) = 1.5Ueq(CH_3) and 1.2Ueq(CH_2, CH) for other hydrogen atoms. The O-bound H atom was placed free due to the hydrogen bonding. The crystallographic data for the structure of *(R,S)*-**3b** has been published as supplementary publication number 2144567 (*(R,S)*-**3b**) in the Cambridge Crystallographic Data Centre. A copy of these data can be obtained for free by applying to CCDC, 12 Union Road, Cambridge CB2 IEZ, UK, fax: 144-(0)1223-336033 or e-mail: deposit@ccdc.cam.ac.uk.

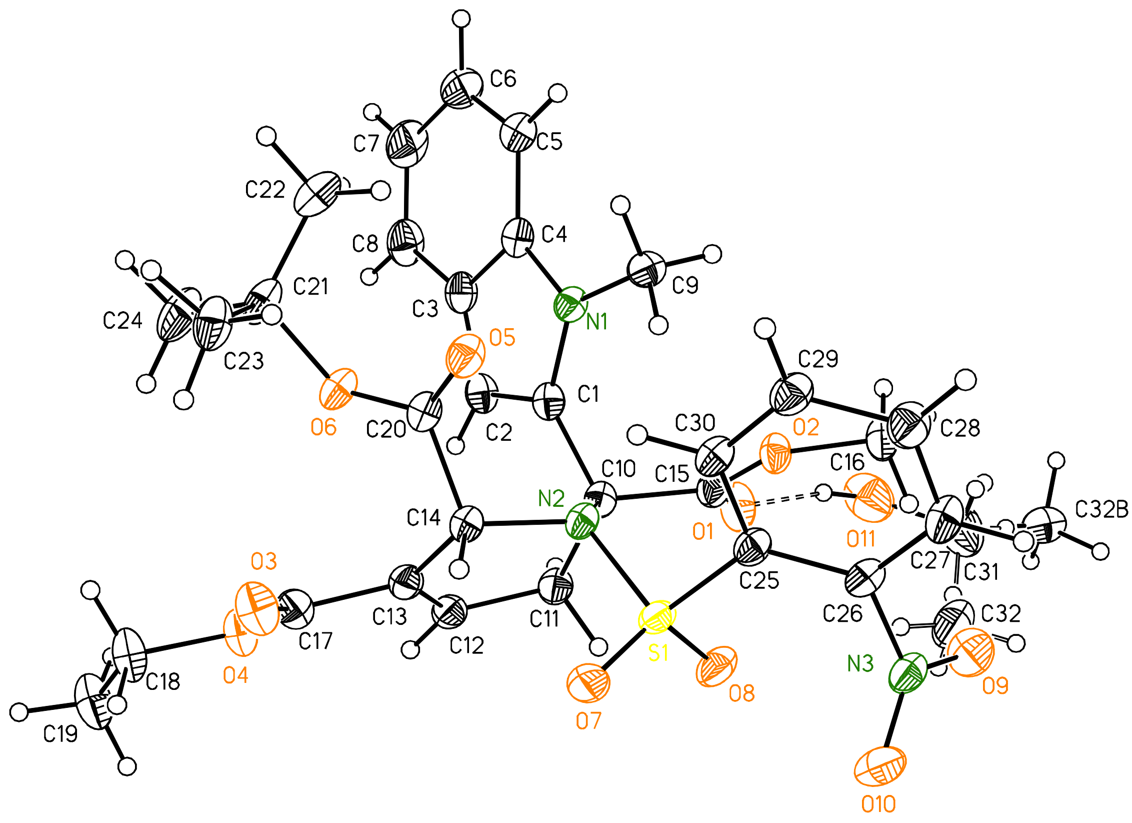

Figure S14. Ortep plot of the molecular structure in the crystal of compound *(R,S)*-**3b**[13]. The displacement ellipsoids are drawn at 50% probability level. Numbering scheme of hydrogen atoms are omitted for clarity.

Table S2. Crystallographic data of compound *(R,S)*-**3b**.

Compound	*(R,S)*-**3b**
Empirical formula	$C_{32}H_{39}N_3O_{11}S$
Formula weight	673.72
Temperature/K	100.0
Crystal system	orthorhombic
Space group	$P2_12_12_1$
a/Å	9.2130(3)
b/Å	15.3432(4)
c/Å	23.2044(4)
α/°	90
β/°	90
γ/°	90
Volume/Å^3	3280.10(15)
Z	4
ρ_{calc}g/cm^3	1.364
μ/mm^{-1}	1.431
F(000)	1424.0
Crystal size/mm^3	0.267 × 0.109 × 0.056
Radiation	CuKα (λ = 1.54178)
2Θ range for data collection/°	6.906 to 159.432
Index ranges	–11 ≤ h ≤ 11, –19 ≤ k ≤ 19, –29 ≤ l ≤ 29
Reflections collected	52330
Independent reflections	7042 [R_{int} = 0.0354, R_{sigma} = 0.0195]
Data/restraints/parameters	7042/0/446
Goodness-of-fit on F^2	1.038
Final R indexes [I>=2σ (I)]	R_1 = 0.0267, wR_2 = 0.0685
Final R indexes [all data]	R_1 = 0.0275, wR_2 = 0.0691
Largest diff. peak/hole / e Å^{-3}	0.2/–0.32
Flack parameter	–0.012(5)

4. Biological Methods

Cell culture

Mammalian cell lines were maintained at 37°C and 5% CO_2 in a humidified atmosphere and sub-cultivated twice a week. All cell lines were tested regularly for mycoplasma contamination and were always free of mycoplasma. BxPC-3 cells (DSMZ#760) were grown in RPMI-1640 medium supplemented with 10% FBS, 2 mM L-glutamine, 1 mM sodium pyruvate, 4.5 g/L glucose, 10 mM HEPES and 1.5 g/L $NaHCO_3$. HeLa cells (DSMZ#57) were cultivated in DMEM supplemented with 10% FBS, 4.5 g/L glucose, 4 mM L-glutamine 1 mM sodium pyruvate, 1% non-essential amino acids, 3.7 g/L $NaHCO_3$. SKOV-3 cells (ATCC#HTB-77) were maintained in modified McCoy's 5A medium supplemented with 10% FBS, 1.5 mM L-glutamine and 2.2 g/L $NaHCO_3$.

Kynurenine (Kyn) assays

High-throughput Kyn assay

Automated screening for modulators of Kyn levels in BxPC-3 cells was performed as published earlier.[2] Briefly, BxPC-3 cells (1,000 cells/well) were seeded in black 1536-well plates prior to incubation for 24 h and subsequent treatment with compounds, 380 µM L-Trp and 50 ng/mL IFN-γ for 48 h. Afterwards, trichloroacetic acid was added to a final concentration of 7% (v/v), the plates were incubated for 10 min at 37°C and centrifuged for 10 min at 1620 x g. Kyn was detected by addition of 17.5 µM Kyn sensor[14] in assay buffer (excitation: 535 nm, emission: 595 nm). In total, 157,332 compounds were screened at a concentration of 7.1 µM. Compounds which showed no cytotoxicity and reduced Kyn levels by ≥70% were subjected to IC_{50} determination.

Manual Kyn assay

For manual testing, BxPC-3, HeLa or SKOV-3 cells (20,000 cells/well, 5,000 cells/well or 6,250 cells/well, respectively) were seeded in 96-well plates (BxPC-3 and HeLa) or 384-well plates (SKOV-3) in medium without phenol red. After 24 h, the IDO1 pathway was induced by addition of 50 ng/mL (BxPC-3 and HeLa) or 17.6 ng/mL IFN-γ (SKOV-3) and 380 µM (BxPC-3), 164.15 µM (HeLa) or 450 µM L-Trp (SKOV-3) with simultaneous treatment with the compounds at the indicated concentrations followed by incubation for 48 h. For the hemin competition assay, hemin was added together with IFN-γ, L-Trp and the compounds. Subsequently, trichloroacetic acid was added to a final concentration of 7% (v/v), the plates were incubated for 15 min at room

temperature and centrifuged for 10 min at 1800 x g. Afterwards, an equal volume of freshly prepared 2% (w/v) *p*-DMAB in glacial acetic acid (Ehrlich reagent) was added prior to measuring the absorbance at 492 nm and 650 nm on the Spark® Multimode Microplate Reader (Tecan, AT). To determine the Kyn levels, the absorbance of the Kyn-*p*-DMAB adduct A_{492} was subtracted by the background absorbance A_{650} and normalized to the DMSO control. Data analysis was performed using a non-linear regression curve fit and GraphPad Prism 9.0 (GraphPad Software, Inc, US) to generate dose-response curves and obtain IC_{50} values.

Kyn assay in IDO1-HEK293T cells

HEK293T cells (25,000 cells/well) were reverse-transfected with pCMV3-IDO1 (1 µg/96-well plate, Sino Biological, CN) using Lipofectamine™ 2000 (Invitrogen, US) prior to incubation for 20 h. Subsequently, cells were treated with 500 µM L-Trp and the compounds for 24 h. Thereafter, trichloroacetic acid was added to a final concentration of 7% (v/v), the plates were incubated for 15 min at room temperature and centrifuged for 10 min at 1800 x g. Afterwards, an equal volume of freshly prepared 2% (w/v) *p*-DMAB in glacial acetic acid (Ehrlich reagent) was added prior to measuring the absorbance at 492 nm and 650 nm on the Spark® Multimode Microplate Reader (Tecan, AT). To determine the Kyn levels, the absorbance of the Kyn-*p*-DMAB adduct A_{492} was subtracted by the background absorbance A_{650} and normalized to the DMSO control. Data analysis was performed using a non-linear regression curve fit and GraphPad Prism 9.0 (GraphPad Software, Inc, US) to generate dose-response curves and obtain IC_{50} values.

in vitro Kyn assay

Recombinant human His-IDO1 (rhIDO1) was expressed in *E. coli* and purified as published previously.[15] To detect direct inhibition, 1 µM rhIDO1 was incubated with compounds for 40 min at 37°C in 50 mM potassium phosphate buffer (16.9 mM K_2HPO_4, 33.1 mM, KH_2PO_4, pH 6.5). Subsequently, 10 mM ascorbic acid, 10 µM methylene blue, 2 mM L-Trp and 100 µg/mL catalase were added and samples were incubated for 60 min at room temperature or 37°C. Trichloroacetic acid was added to a final concentration of 7% (v/v) and samples were incubated for 30 min at 70°C. Afterwards, an equal volume of freshly prepared 2% (w/v) *p*-DMAB in glacial acetic acid (Ehrlich reagent) was added prior to measuring absorbance at 492 nm and 650 nm on the Spark® Multimode Microplate Reader (Tecan, AT). To determine the Kyn levels, the absorbance of the Kyn-*p*-DMAB adduct A_{492} was subtracted by the background absorbance A_{650} and normalized to the DMSO control. Data analysis was performed using a non-linear regression curve fit and

GraphPad Prism 9.0 (GraphPad Software, Inc, US) to generate dose-response curves and obtain IC_{50} values.

IDO1 promoter reporter gene assay

HEK293T cells (25,000 cells/well) were reverse-transfected with pXPG-IDO1[16] (4 µg/96-well plate, kindly provided by Gina M. Doody, Leeds, UK) and pRL-TK (300 ng/96-well plate, Promega, US) using Lipofectamine™ 2000 (Invitrogen, USA) prior to incubation for 24 h. Afterwards, cells were treated with 50 ng/mL IFN-γ and the compounds for 48 h. Luminescence generated by the Fluc reporter and the control reporter Rluc was measured using the Dual-Glo ® Luciferase Assay System (Promega, US) on the Spark® Multimode Microplate Reader (Tecan, AT). To determine the *IDO1* promoter activity, Fluc signals were divided by Rluc signals and normalized to the values of the DMSO control. Data analysis was performed using a non-linear regression curve fit and GraphPad Prism 9.0 (GraphPad Software, Inc, US) to generate dose-response curves and obtain IC_{50} values.

Trp uptake assay

BxPC-3 cells (30,000 cells/well) were seeded in 96-well plates in Trp-free RPMI 1640 medium and incubated for 48 h prior addition of 50 ng/mL IFN-γ. Trp starvation was continued for another 24 h. Afterwards, medium was exchanged for Trp-free medium containing the control inhibitors 5 mM L-Leu (inhibitor of system-L amino acid transporters (LAT)), 1 mM 1-methyl-L-tryptophan (inhibitor of tryptophanyl-tRNA synthetase (TrpRS)) and compounds and cells were incubated for 30 min. Subsequently, 50 µM L-Trp was added and samples were incubated for another 30 min. The supernatant was transferred to a new plate, trichloroacetic acid was added to a final concentration of 7% (v/v) and plates were incubated for 15 min at room temperature. Samples were centrifuged for 10 min at 1800 x g and L-Trp was quantified by HPLC-MS/MS using the LTQ Velos Pro and Dionex HPLC (Thermo Fisher Scientific, US). Data analysis was performed with Xcalibur™ (Thermo Fisher Scientific, US) and represented using GraphPad Prism 9.0 (GraphPad Software, Inc, US).

RNA purification and RT-qPCR

HeLa cells (250,000 cells/well) were seeded in 6-well plates and incubated for 24 h prior addition of 50 ng/mL IFN-γ and compounds. After 24 h, RNA was extracted using the RNeasy Plus Mini

Kit (QIAGEN, DE) following the manufacturer’s procedure. DNA was removed on column by DNase digestion with RNase-free DNase Set (QIAGEN, DE) according to the manufacturer’s instructions. Following the manufacturer’s protocol, cDNA templates were synthesized from 800 ng total RNA using the QuantiTect Reverse Transcription Kit (QIAGEN, DE).

The expression levels of the *IDO1* gene and the reference *GAPDH* were assessed by real-time quantitative PCR (qPCR). Therefore, 120 ng cDNA was amplified using 500 nM of gene-specific primers and SsoAdvanced Universal SYBR Green Supermix (Bio-Rad Laboratories, DE) in a total volume of 10 µL for 50 cycles using the CFX96 Touch™ Real-Time PCR Detection System (Bio-Rad Laboratories, DE). Relative *IDO1* expression levels were calculated using the $\Delta\Delta C_t$ method with *GAPDH* as reference gene[17] and represented using GraphPad Prism 9.0 (GraphPad Software, Inc, US).

The sequences of the primers for *IDO1* were 5’-GCCTGATCTCATAGAGCTTGGC-3’ (forward) and 5’-TGCATCCCAGAACTAGACGTGC-3’ (reverse). The sequences of the primers for *GAPDH* were 5’-GTCTCCTCTGACTTCAACAGCG-3’ (forward) and 5’-ACCACCCTGTTGCTGTAGCCAA-3’ (reverse).

Immunoblotting

HeLa cells (250,000 cells/well) were seeded in 6-well plates and incubated for 24 h prior to addition of 50 ng/mL IFN-γ and compounds. After 24 h, cells were lysed using Laemmli buffer (8% glycerol, 4.4% 0.5 M TRIS, pH 6.8, 3.1 mM SDS, 4.44 mM DTT, 33.2 mM bromophenol blue) and homogenized by sonication. Protein concentration was determined using the DC Protein Assay (Bio-Rad Laboratories, DE). 40 µg total protein was separated using 10% polyacrylamide gels or Any kD™ Mini-PROTEAN® TGX Stain-Free™ Protein Gels (Bio-Rad Laboratories, DE) under reducing/denaturing conditions in a TRIS/glycine-based system. Proteins were transferred to a PVDF membrane for 60 min at 100 V (Thermo Fisher Scientific, USA) using wet tank transfer (192 mM glycine, 25 mM TRIS, 10% methanol) in a Mini Trans-Blot® Cell (Bio-Rad Laboratories, DE). Afterwards, membranes were blocked with 5% non-fat milk in PBS with 0.1% (v/v) Tween-20 (PBS-T) for 60 min at room temperature followed by overnight incubation at 4°C with the primary antibodies. For detection of IDO1 and the reference protein vinculin, the primary antibodies anti-IDO1 (1:5,000, ab211017, Abcam, UK) and anti-vinculin (1:10,000, V9131, Merck KGaA, DE) in 5% milk in PBS-T were used. Protein bands were visualized with secondary antibodies conjugated to IRDye® Infrared Fluorescent Dyes (1:5,000, LI-COR Biosciences, US)

using the ChemiDoc MP Imaging System (Bio-Rad Laboratories, DE). IDO1 protein levels were normalized to the levels of reference protein vinculin.

In-Cell Western

BxPC-3 cells (10,000 cells/well) were seeded in 96-well plates and incubated for 24 h prior to addition of 50 ng/mL IFN-γ and compounds at indicated concentrations for 48 h. Cells were fixed with 4% paraformaldehyde for 20 min at room temperature followed by permeabilization with 0.5% Triton X-100 for 15 min at room temperature. Afterwards, unspecific binding sites were blocked with 5% non-fat milk in PBS with 0.1% (v/v) Tween-20 (PBS-T) for 60 min at room temperature followed by overnight incubation at 4°C with the primary antibodies. For detection of IDO1 and the reference protein vinculin, the primary antibodies anti-IDO1 (1:3,000, ab211017, Abcam, GBR) and anti-vinculin (1:5,000, V9131, Merck KGaA, DE) in 5% milk in PBS-T were used. For visualization, secondary antibodies conjugated to IRDye® Infrared Fluorescent Dyes (1:500, LI-COR Biosciences, US) were used (Bio-Rad Laboratories, DE). Images were acquired with the Odyssey® CLx Infrared Imaging System (LI-COR Biosciences, US) and processed using Image Studio 5.2 (LI-COR Biosciences, US). IDO1 protein levels were normalized to the levels of reference protein vinculin).

nanoDSF

11 µM rhIDO1 was incubated with compounds at indicated concentrations for 60 min at 37°C in 50 mM potassium phosphate buffer (16.9 mM K_2HPO_4, 33.1 mM, KH_2PO_4, pH 6.5). The thermal protein stability from 20°C to 90°C (1°C/min) was measured by means of the intrinsic tryptophan/tyrosine fluorescence using the Prometheus™ NT.48 (NanoTemper® Technologies, DE). Melting scans, first derivatives of melting scans and melting temperatures were analyzed using PR.ThermControl software (NanoTemper® Technologies, DE). Binding affinities were determined by plotting the melting temperature against the ligand concentration and fitting with a single site ligand binding function using GraphPad Prism 9.0 (GraphPad Software, Inc, US). The isothermal analysis of the nanoDSF data was performed with FoldAffinity[1] with an assumed heat capacity change ΔC_p of zero.

UV/Vis spectrophotometry

10 µM rhIDO1 was incubated with compounds at indicated concentrations for 120 min at 37°C in 50 mM potassium phosphate buffer (16.9 mM K_2HPO_4, 33.1 mM, KH_2PO_4, pH 6.5) using UV-

transparent microplates (UV-STAR®, Greiner AG, AT). Subsequently, absorbance spectra from 250 nm to 550 nm were recorded on the Spark® Multimode Microplate Reader (Tecan, AT).

In-Cell CETSA

SKOV-3 cells (1.25 x 10^6 cells/flask) were seeded in two T75 tissue culture flasks and incubated for 24 h prior to addition of 17.6 ng/mL IFN-γ and 10 µM succinylacetone (SA, heme synthesis inhibitor). After 24 h, cells were treated with the compound (C) or DMSO (V, vehicle) for 15 min at 37°C. Cells were detached with trypsin/EDTA and washed thrice in cold PBS after resuspension in cold PBS. C- and V-treated samples were distributed equally into ten tubes each and subjected to heating at different temperatures in the MasterCycler EpGradient S (Eppendorf SE, DE). Afterwards, NP-40 alternative was added to a final concentration of 0.4% (v/v) and cells were lysed by five consecutive freeze/thaw cycles. Soluble fractions were separated from denatured proteins by centrifugation at 100,000 x g and 4°C for 20 min. Supernatants were transferred to new tubes and subjected to immunoblot analysis.

Stability of Apoxidole-1 in Serum

400 µM of apoxidole-1 was incubated in Gibco® Qualified FBS (Thermo Fisher Scientific, Inc, US) at 37°C for up to 48 h. Samples of 50 µL were taken after 0, 6, 10, 24, 30 and 48 h, 200 µL of DCM was added and the compound was extracted by thorough vortexing for 1 min. Subsequently, the samples were centrifuged at 16,000 x g and 22°C for 15 min. The organic phase was dried with magnesium sulfate and filtered through a HPLC filter prior to HPLC-ESI-MS analysis using the LTQ Fleet (Thermo Fisher Scientific, US), Ultimate 3000 HPLC (Thermo Fisher Scientific, US) and Xcalibur™ software (Thermo Fisher Scientific, US).

Crystallization of apo-IDO1

A truncated version of recombinant human GST-IDO1 (rhIDO1) consisting of amino acids 5-400 of the 403 amino acid-long full-length human IDO1 was expressed in *E. coli* and purified as published previously.[15] The heme cofactor was released from IDO1 by overnight incubation with 2-mercaptoethanesulfonate (MESNA) as described previously[18] and heme occupancy was determined by UV/Vis spectrophotometry.

450 µM of IDO1 protein was mixed with 750 µM apoxidole-1 (2.5% (v/v) DMSO) in 25 mM TRIS-HCl, pH 8.0, 100 µM tris(2-carboxyethyl)phosphine (TCEP) and incubated for 2 h at 42°C. The soluble protein fraction was separated from precipitate by centrifugation at 20,000 x g and 18°C

for 10 min. Protein crystals were obtained from a sitting drop setup (iQ plates, SPT Labtech, UK) by addition of 100 nL of the reservoir solution (40% (v/v) PEG200 in 100 mM MES, pH 6.5) to 100 nL of protein solution and incubation at 20°C. Crystals were harvested after 7 days by addition of reservoir solution as cryoprotectant to the drop prior to plunging of the crystals in liquid nitrogen. Synchrotron X-ray diffraction data was acquired to 1.6 Å from the X10SA beamline at the Swiss Light Source at the Paul Scherrer Institute, CH. The data was processed with XDS[19] and scaled using XSCALE. The structure was solved using a model of IDO1 from pdb 6dpq with Phaser[20] from the Phenix suite[21], followed by repetitive cycles of refinement with phenix.refine and Coot.[22] Figures were created with PyMOL Molecular Graphics System (Version 2.5.2 Schrödinger, LLC).

The integrity of apoxidole-1 in the protein solution was assessed 63 days after the crystallization setup by mixing the crystallization drop with 100 µL of DCM and recording of HPLC-ESI-MS spectra using the LTQ Fleet (Thermo Fisher Scientific, US), Ultimate 3000 HPLC (Thermo Fisher Scientific, US) and Xcalibur™ software (Thermo Fisher Scientific, US).

TDO Activity Assay

Inhibition of tryptophan 2,3-dioxygenase (TDO) was tested using the Universal IDO1/IDO2/TDO Inhibitor Screening Assay Kit (cat# 72035, BPS Bioscience, US). 500 ng His-tagged TDO in TDO Assay Buffer was incubated with Reaction Solution and 50 µM Test Inhibitor (0.5% (v/v) DMSO) for 120 min at 37°C with slow shaking in the dark. Afterwards, absorbance at 321 nm was recorded on the Spark® Multimode Microplate Reader (Tecan, AT), all values were subtracted by the background and normalized to the DMSO control.

IDO2 Activity Assay

Inhibition of indoleamine 2,3-dioxygenase 2 (IDO2) was tested using the Universal IDO1/IDO2/TDO Inhibitor Screening Assay Kit (cat# 72035, BPS Bioscience, US). 10 µg His-tagged IDO2 in IDO2 Assay Buffer was incubated with Complete IDO2 Reaction Solution (1% (v/v) component 2 in component 1) and 50 µM Test Inhibitor (0.5% (v/v) DMSO) for 90 min at 30°C with slow shaking in the dark prior to initiation of the reaction by addition of 20% (v/v) IDO2 Substrate. Samples were incubated for 120 min at 30°C with slow shaking in the dark. Afterwards, absorbance at 321 nm was recorded on the Spark® Multimode Microplate Reader (Tecan, AT), all values were subtracted by the background and normalized to the DMSO control.

5. Author Contributions

C.D. and L.D. contributed equally to this work. C.D. performed all chemical synthesis and analysis of compounds. L.D. performed all cellular assays, protein and mRNA expression analyses, nanoDSF experiments, UV/Vis spectrophotometry, CETSA experiments and biochemical TDO and IDO2 assays. M.G.C. and K.Y. performed reaction optimisation. E.H. established conditions for the Kyn assay and performed the biochemical Kyn assay. R.S. and C.S. contributed to the crystal structure analysis of apoxidole-1 and R.G. solved the protein crystal structure. S.S. adapted the conditions for the Kyn assay to an automated high-throughput format and performed the analysis of high-throughput data. K.K., S.Z. and H.W. designed the research. C.D., L.D., S.Z. and H.W. wrote the manuscript.

6. Supplementary References

[1] a) S. Niebling, O. Burastero, J. Bürgi, C. Günther, L. A. Defelipe, S. Sander, E. Gattkowski, R. Anjanappa, M. Wilmanns, S. Springer, H. Tidow, M. García-Alai, *Scientific Reports* **2021**, *11*, 9572; b) O. Burastero, S. Niebling, L. A. Defelipe, C. Günther, M. García-Alai, A. Struve, *Acta Crystallogr D Biol Crystallogr Struct Biol* **2021**, *77*, 1241.

[2] E. Hennes, P. Lampe, L. Dötsch, N. Bruning, L. M. Pulvermacher, S. Sievers, S. Ziegler, H. Waldmann, *Angew Chem Int Ed Engl* **2021**, *60*, 9869.

[3] a) A. Burhop, S. Bag, M. Grigalunas, S. Woitalla, P. Bodenbinder, L. Brieger, C. Strohmann, A. Pahl, S. Sievers, H. Waldmann, *Adv Sci (Weinh)* **2021**, *8*, e2102042; b) N. Kaiser, D. Corkery, Y. Wu, L. Laraia, H. Waldmann, *Bioorg Med Chem* **2019**, *27*, 2444.

[4] a) L. Kremer, E. Hennes, A. Brause, A. Ursu, L. Robke, H. T. Matsubayashi, Y. Nihongaki, J. Flegel, I. Mejdrova, J. Eickhoff, M. Baumann, R. Nencka, P. Janning, S. Kordes, H. R. Scholer, J. Sterneckert, T. Inoue, S. Ziegler, H. Waldmann, *Angew Chem Int Ed Engl* **2019**, *58*, 16617; b) L. Kremer, C. Schultz-Fademrecht, M. Baumann, P. Habenberger, A. Choidas, B. Klebl, S. Kordes, H. R. Schöler, J. Sterneckert, S. Ziegler, G. Schneider, H. Waldmann, *Angewandte Chemie International Edition* **2017**, *56*, 13021.

[5] F. Wesseler, D. Riege, M. Puthanveedu, J. Halver, E. Müller, J. Bertrand, A. P. Antonchick, S. Sievers, H. Waldmann, D. Schade, *J Med Chem* **2022**, *65*, 3978.

[6] A. Friese, S. Kapoor, T. Schneidewind, S. R. Vidadala, J. Sardana, A. Brause, T. Forster, M. Bischoff, J. Wagner, P. Janning, S. Ziegler, H. Waldmann, *Angew Chem Int Ed Engl* **2019**, *58*, 13009.

[7] a) G. Karageorgis, E. S. Reckzeh, J. Ceballos, M. Schwalfenberg, S. Sievers, C. Ostermann, A. Pahl, S. Ziegler, H. Waldmann, *Nat Chem* **2018**, *10*, 1103; b) D. Trauner, C. Fischer, *Synfacts* **2019**, *15*, 1440.

[8] Y. S. Tran, O. Kwon, *Organic Letters* **2005**, *7*, 4289.

[9] K. Junge, B. Hagemann, S. Enthaler, A. Spannenberg, M. Michalik, G. Oehme, A. Monsees, T. Riermeier, M. Beller, *Tetrahedron-Asymmetry* **2004**, *15*, 2621.

[10] O. V. Dolomanov, L. J. Bourhis, R. J. Gildea, J. A. K. Howard, H. Puschmann, *Journal of Applied Crystallography* **2009**, *42*, 339.

[11] G. Sheldrick, *Acta Crystallographica Section A* **2015**, *71*, 3.

[12] G. Sheldrick, *Acta Crystallographica Section C* **2015**, *71*, 3.

[13] L. Farrugia, *Journal of Applied Crystallography* **1997**, *30*, 565.

[14] E. K. Feuster, T. E. Glass, *Journal of the American Chemical Society* **2003**, *125*, 16174.

[15] T. K. Littlejohn, O. Takikawa, D. Skylas, J. F. Jamie, M. J. Walker, R. J. Truscott, *Protein Expr Purif* **2000**, *19*, 22.

[16] N. A. Barnes, S. J. Stephenson, R. M. Tooze, G. M. Doody, *The Journal of Immunology* **2009**, *183*, 5768.

[17] M. W. Pfaffl, *Nucleic Acids Res* **2001**, *29*, e45.

[18] R. F. Ortiz-Meoz, L. Wang, R. Matico, A. Rutkowska-Klute, M. De la Rosa, S. Bedard, R. Midgett, K. Strohmer, D. Thomson, C. Zhang, M. Mebrahtu, J. Guss, R. Totoritis, T. Consler, N. Campobasso, D. Taylor, T. Lewis, K. Weaver, M. Muelbaier, J. Seal, R. Dunham, W. Kazmierski, D. Favre, G. Bergamini, L. Shewchuk, A. Rendina, G. Zhang, *ChemBioChem* **2021**, *22*, 516.

[19] W. Kabsch, *Acta Crystallogr D Biol Crystallogr* **2010**, *66*, 125.

[20] A. J. McCoy, R. W. Grosse-Kunstleve, P. D. Adams, M. D. Winn, L. C. Storoni, R. J. Read, *J Appl Cryst* **2007**, *40*, 658.

[21] D. Liebschner, P. V. Afonine, M. L. Baker, G. Bunkoczi, V. B. Chen, T. I. Croll, B. Hintze, L.-W. Hung, S. Jain, A. J. McCoy, N. W. Moriarty, R. D. Oeffner, B. K. Poon, M. G. Prisant,

R. J. Read, J. S. Richardson, D. C. Richardson, M. D. Sammito, O. V. Sobolev, D. H. Stockwell, T. C. Terwilliger, A. G. Urzhumtsev, L. L. Videau, C. J. Williams, P. D. Adams, *Acta Crystallogr D Biol Crystallogr* **2019**, *75*, 861.
[22] a) P. V. Afonine, R. W. Grosse-Kunstleve, N. Echols, J. J. Headd, N. W. Moriarty, M. Mustyakimov, T. C. Terwilliger, A. Urzhumtsev, P. H. Zwart, P. D. Adams, *Acta Crystallogr D Biol Crystallogr* **2012**, *68*, 352; b) P. Emsley, B. Lohkamp, W. G. Scott, K. Cowtan, *Acta Crystallogr D Biol Crystallogr* **2010**, *66*, 486.

7. Publication Licenses

49 Spadina Ave. Suite 200
Toronto ON M5V 2J1 Canada
www.biorender.com

Confirmation of Publication and Licensing Rights

December 22nd, 2021
Science Suite Inc.

Subscription:	*Institution*
Agreement number:	*ZH23CK1BVN*
Journal name:	*Angewandte Chemie International Edition*

To whom this may concern,

This document is to confirm that Lara Dötsch has been granted a license to use the BioRender content, including icons, templates and other original artwork, appearing in the attached completed graphic pursuant to BioRender's Academic License Terms. This license permits BioRender content to be sublicensed for use in journal publications.

All rights and ownership of BioRender content are reserved by BioRender. All completed graphics must be accompanied by the following citation: “Created with BioRender.com”.

BioRender content included in the completed graphic is not licensed for any commercial uses beyond publication in a journal. For any commercial use of this figure, users may, if allowed, recreate it in BioRender under an Industry BioRender Plan.

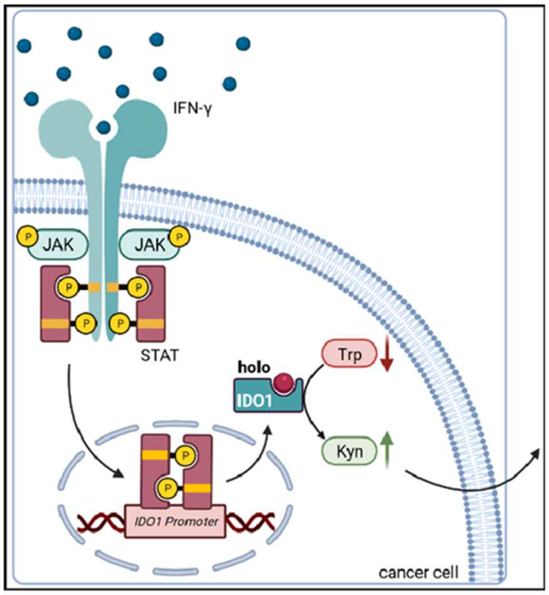

For any questions regarding this document, or other questions about publishing with BioRender refer to our BioRender Publication Guide, or contact BioRender Support at support@biorender.com.

49 Spadina Ave. Suite 200
Toronto ON M5V 2J1 Canada
www.biorender.com

Confirmation of Publication and Licensing Rights

December 22nd, 2021
Science Suite Inc.

Subscription:	*Institution*
Agreement number:	*OA23CK8SLW*
Journal name:	*Angewandte Chemie International Edition*

To whom this may concern,

This document is to confirm that Lara Dötsch has been granted a license to use the BioRender content, including icons, templates and other original artwork, appearing in the attached completed graphic pursuant to BioRender's Academic License Terms. This license permits BioRender content to be sublicensed for use in journal publications.

All rights and ownership of BioRender content are reserved by BioRender. All completed graphics must be accompanied by the following citation: "Created with BioRender.com".

BioRender content included in the completed graphic is not licensed for any commercial uses beyond publication in a journal. For any commercial use of this figure, users may, if allowed, recreate it in BioRender under an Industry BioRender Plan.

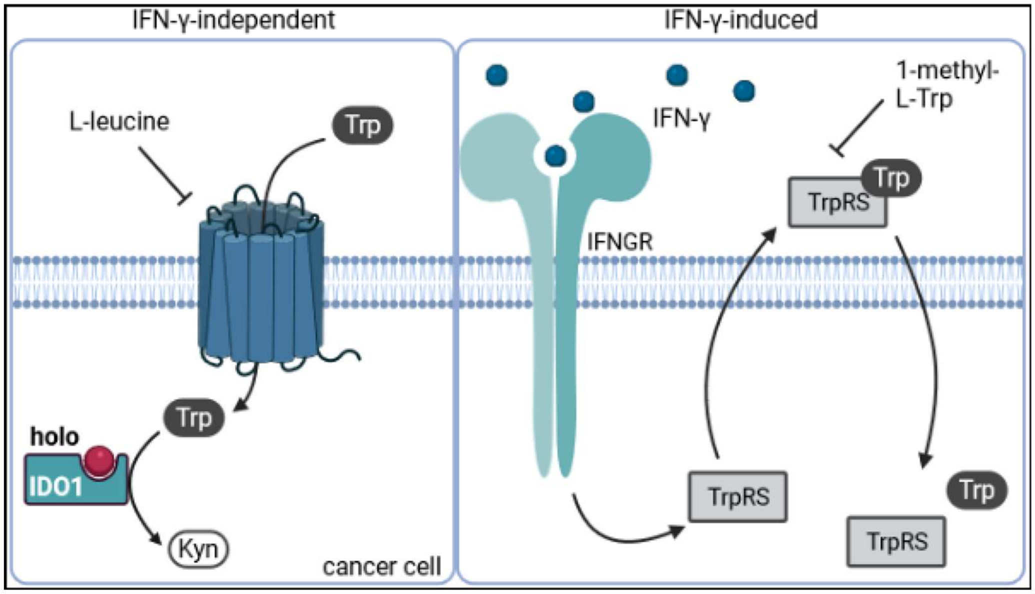

For any questions regarding this document, or other questions about publishing with BioRender refer to our BioRender Publication Guide, or contact BioRender Support at support@biorender.com.

49 Spadina Ave. Suite 200
Toronto ON M5V 2J1 Canada
www.biorender.com

Confirmation of Publication and Licensing Rights

February 25th, 2022
Science Suite Inc.

Subscription:	*Institution*
Agreement number:	*OI23LT5VEZ*
Journal name:	*Angewandte Chemie International Edition*

To whom this may concern,

This document is to confirm that Lara Dötsch has been granted a license to use the BioRender content, including icons, templates and other original artwork, appearing in the attached completed graphic pursuant to BioRender's Academic License Terms. This license permits BioRender content to be sublicensed for use in journal publications.

All rights and ownership of BioRender content are reserved by BioRender. All completed graphics must be accompanied by the following citation: "Created with BioRender.com".

BioRender content included in the completed graphic is not licensed for any commercial uses beyond publication in a journal. For any commercial use of this figure, users may, if allowed, recreate it in BioRender under an Industry BioRender Plan.

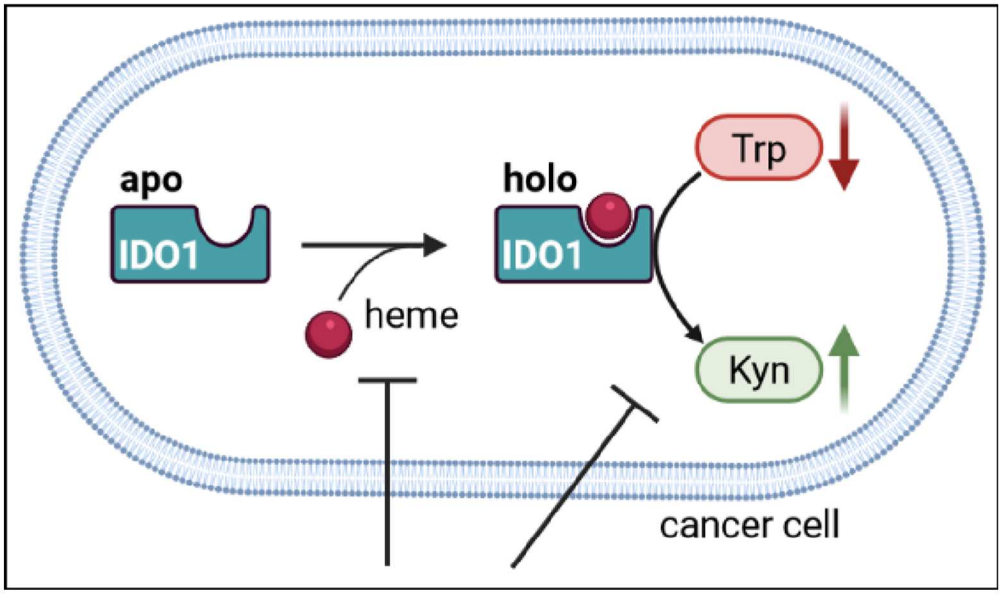

For any questions regarding this document, or other questions about publishing with BioRender refer to our BioRender Publication Guide, or contact BioRender Support at support@biorender.com.

8. NMR Spectra

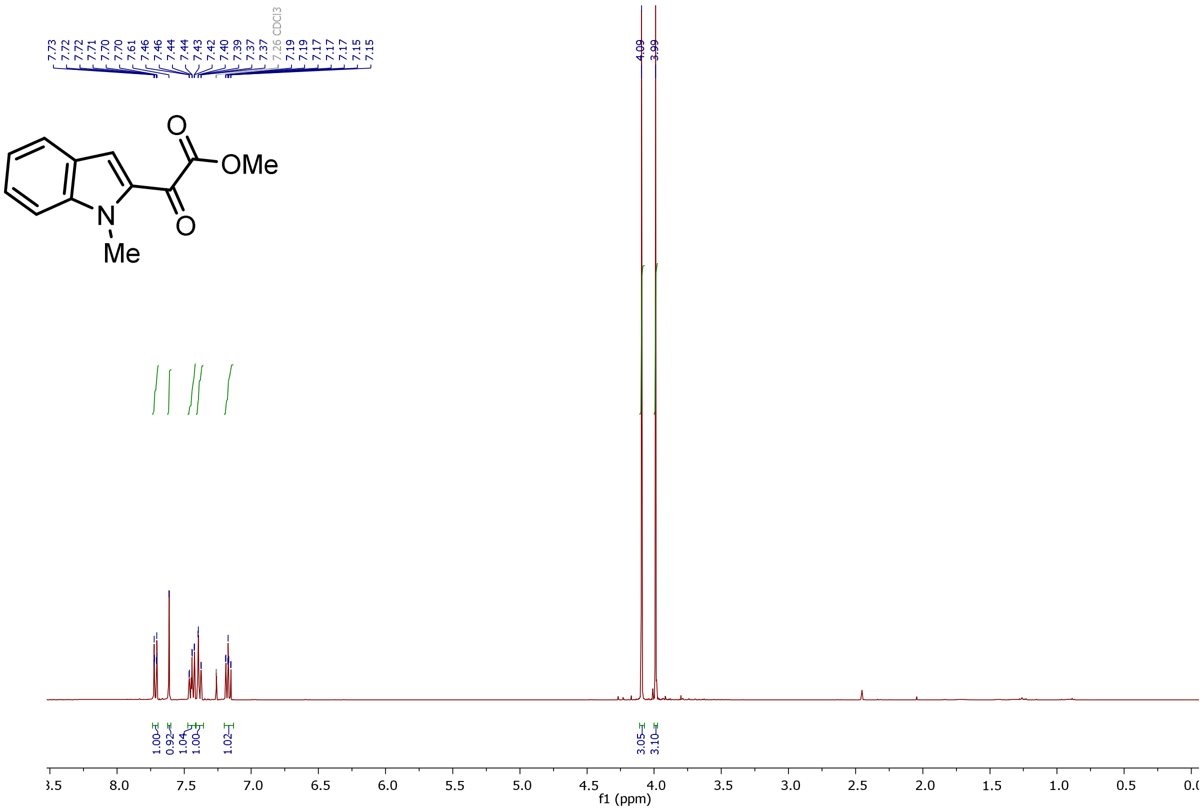

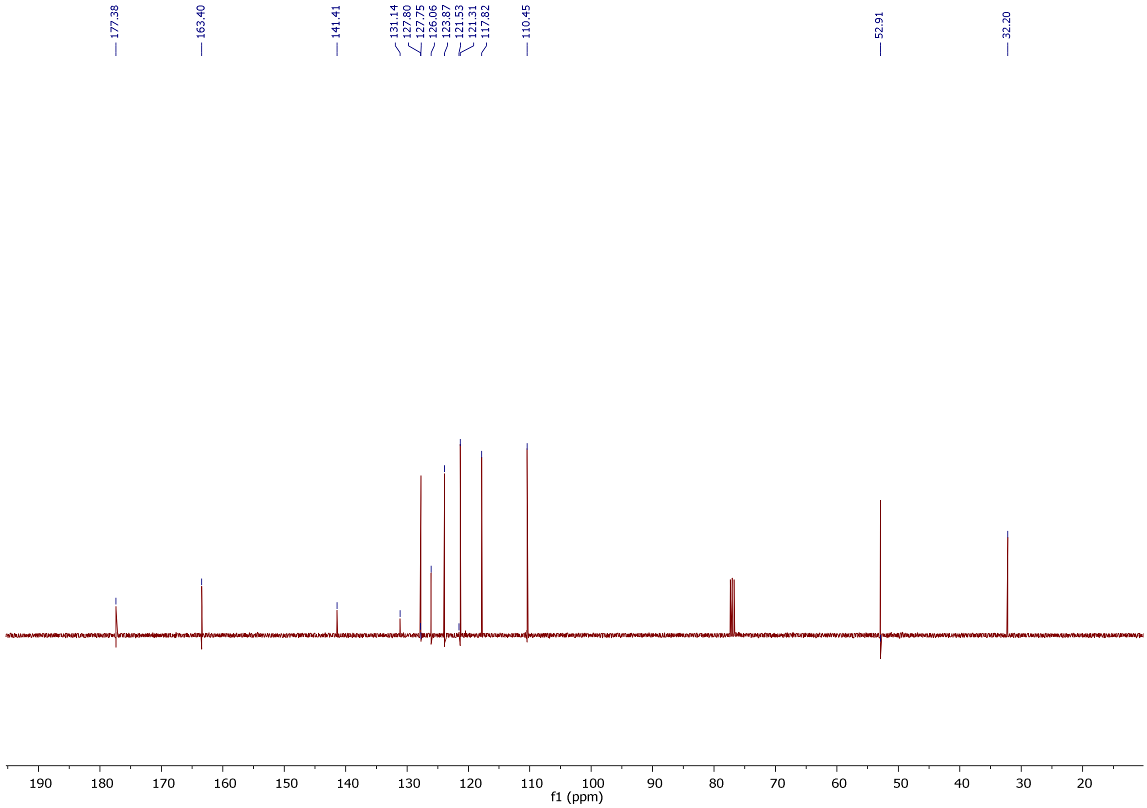
177.38
163.40
141.41
131.14 127.80 127.75 126.06 123.87 121.53 121.31 117.82
110.45
52.91
32.20
190 180 170 160 150 140 130 120 110 100 90 80 70 60 50 40 30 20
f1 (ppm)

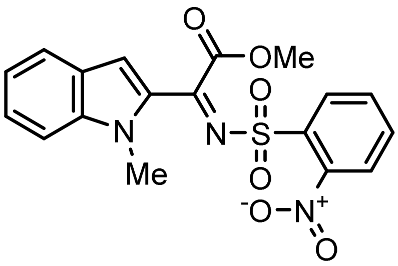
O
OMe
O
N
S
O
N
Me
-O
N+
O

1a

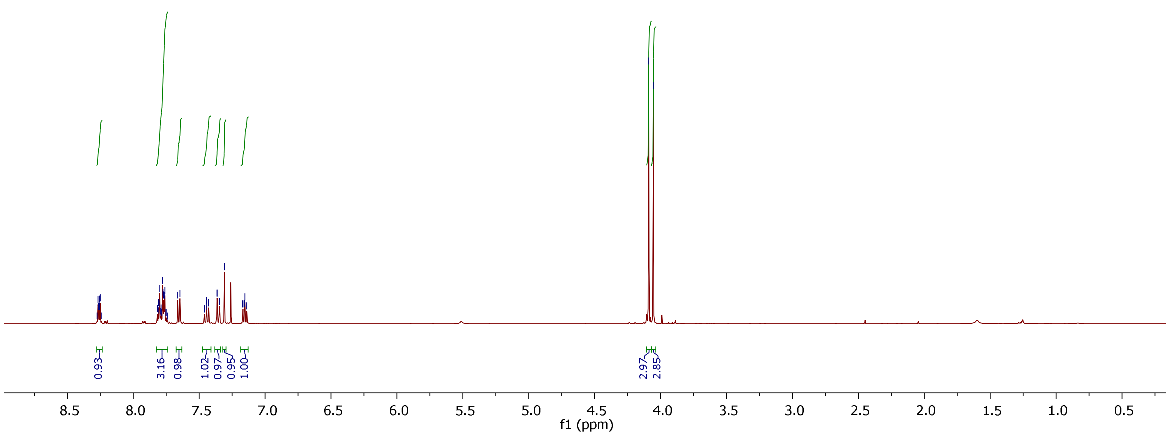
8.27 8.27 8.26 8.26 8.26 8.25 8.24 7.82 7.81 7.81 7.80 7.79 7.79 7.78 7.78 7.77 7.77 7.76 7.76 7.75 7.75 7.74 7.74 7.66 7.65 7.46 7.46 7.45 7.44 7.44 7.43 7.36 7.36 7.35 7.35 7.31 7.17 7.17 7.16 7.15 7.14 7.14
4.09 4.05
0.93 3.16 0.98 1.02 0.97 0.95 1.00
2.97 2.85
8.5 8.0 7.5 7.0 6.5 6.0 5.5 5.0 4.5 4.0 3.5 3.0 2.5 2.0 1.5 1.0 0.5
f1 (ppm)

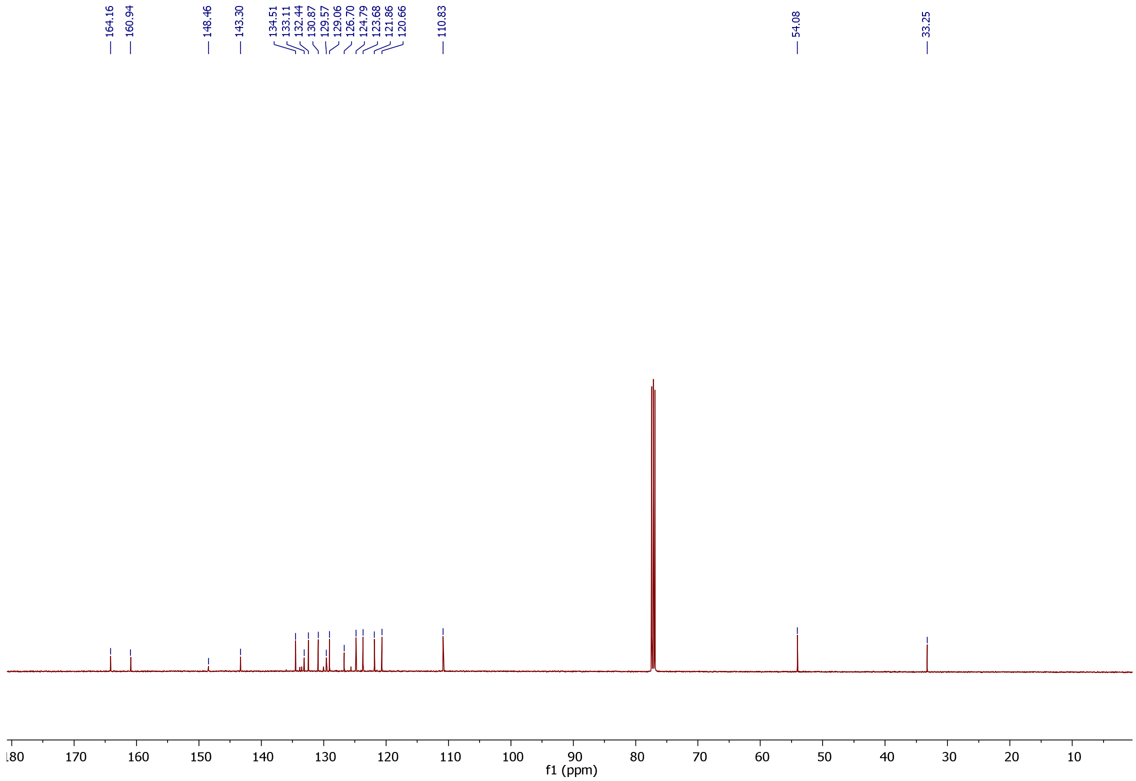
164.16
160.94
148.46
143.30
134.51
133.11
132.44
130.87
129.57
129.06
126.70
124.79
123.68
121.86
120.66
110.83
54.08
33.25
f1 (ppm)

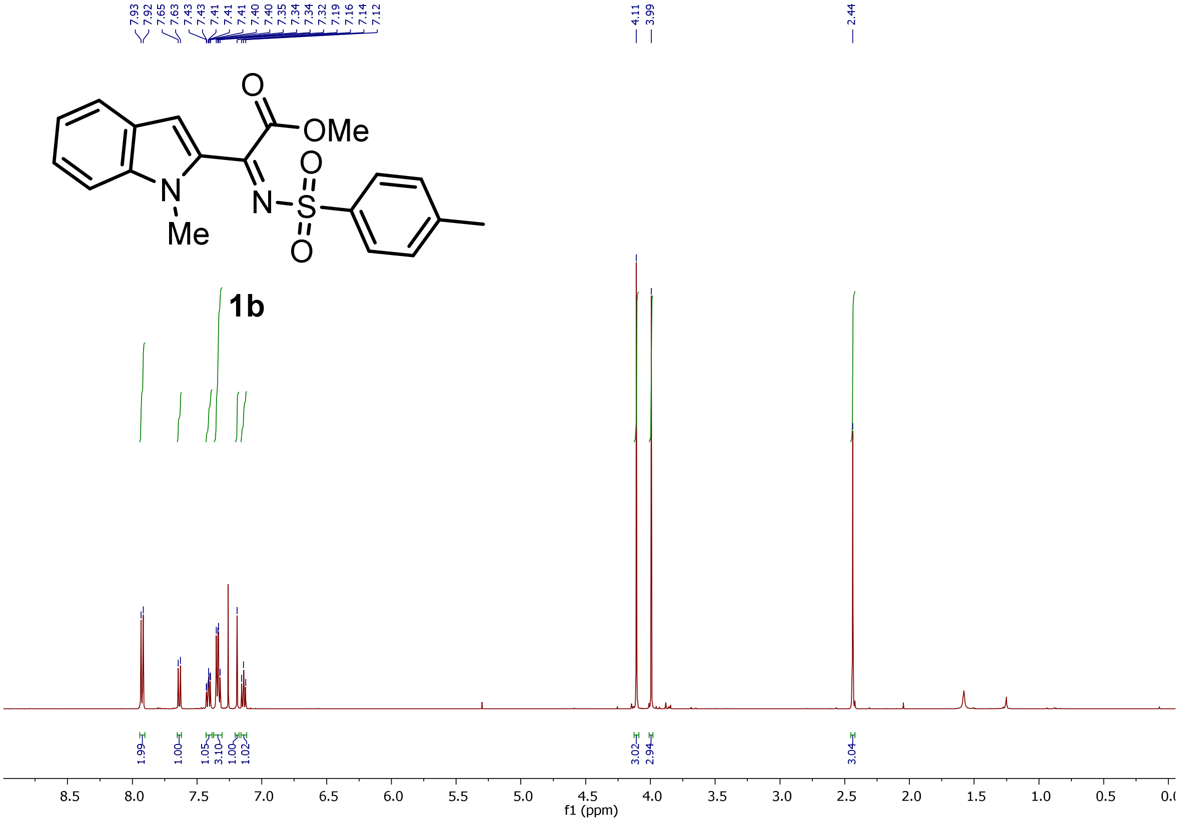
7.93
7.92
7.65
7.63
7.43
7.43
7.41
7.41
7.41
7.40
7.40
7.35
7.34
7.34
7.32
7.19
7.16
7.14
7.12
4.11
3.99
2.44
O
OMe
N
Me
N
S
O
O
1b
1.99
1.00
1.05
3.10
1.00
1.02
3.02
2.94
3.04
f1 (ppm)

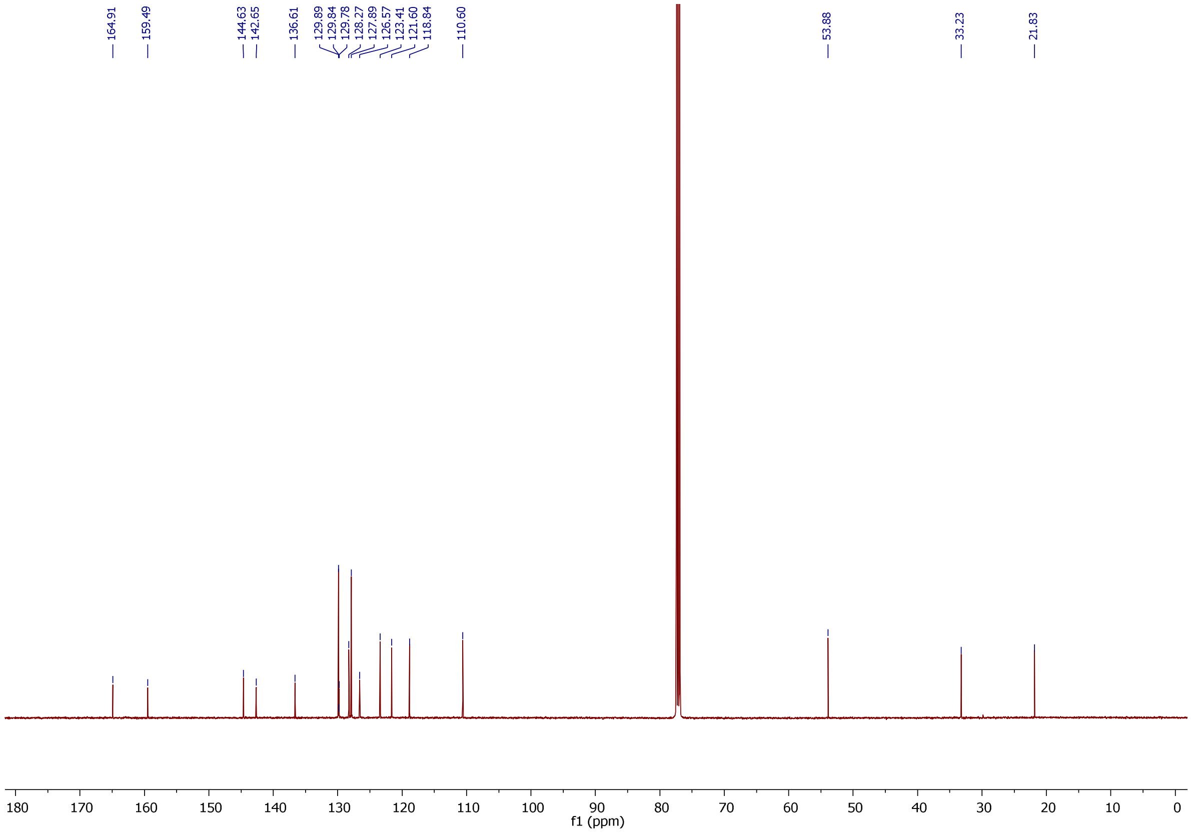
164.91
159.49
144.63
142.65
136.61
129.89
129.84
129.78
128.27
127.89
126.57
123.41
121.60
118.84
110.60
53.88
33.23
21.83
180 170 160 150 140 130 120 110 100 90 80 70 60 50 40 30 20 10 0
f1 (ppm)

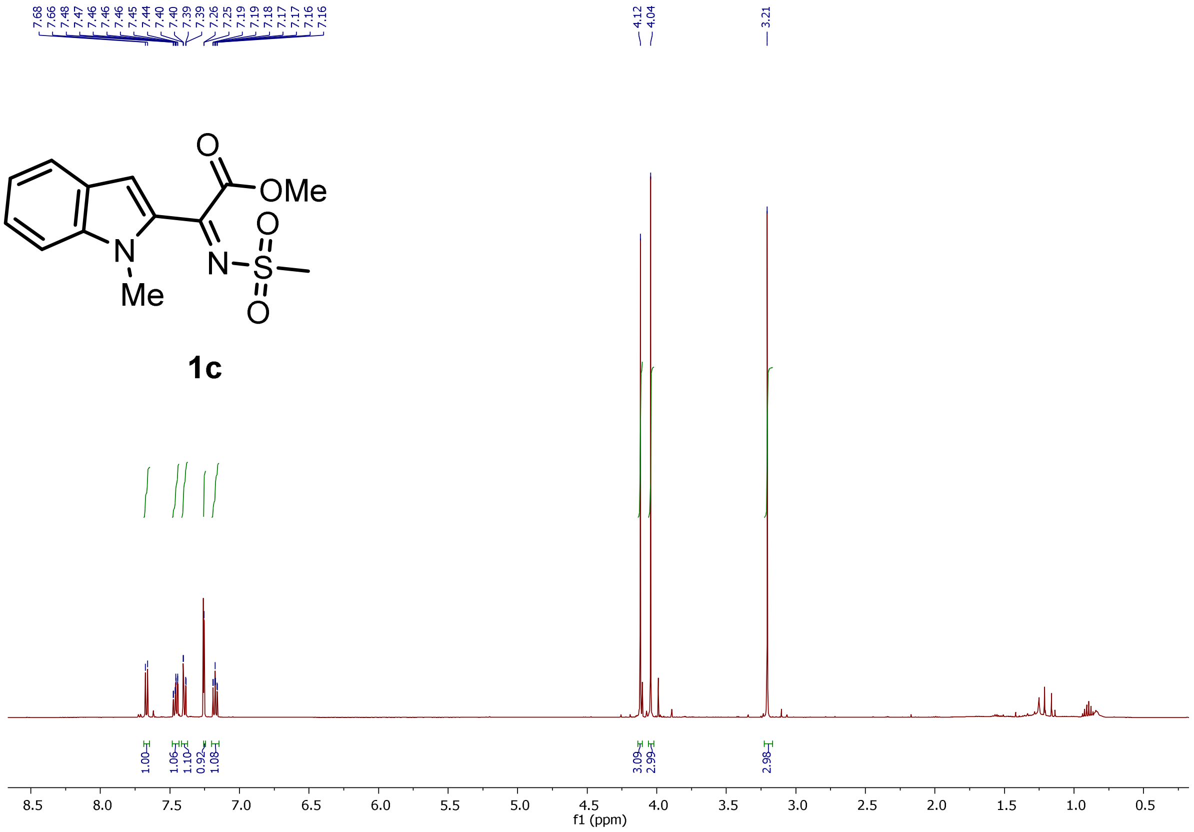
7.68 7.66 7.48 7.47 7.46 7.46 7.46 7.45 7.44 7.40 7.40 7.39 7.39 7.26 7.25 7.19 7.19 7.18 7.17 7.17 7.16 7.16
4.12 4.04
3.21
O
OMe
N
Me
N
S
O
O
1c
1.00
1.06
1.10
0.92
1.08
3.09
2.99
2.98
8.5 8.0 7.5 7.0 6.5 6.0 5.5 5.0 4.5 4.0 3.5 3.0 2.5 2.0 1.5 1.0 0.5
f1 (ppm)

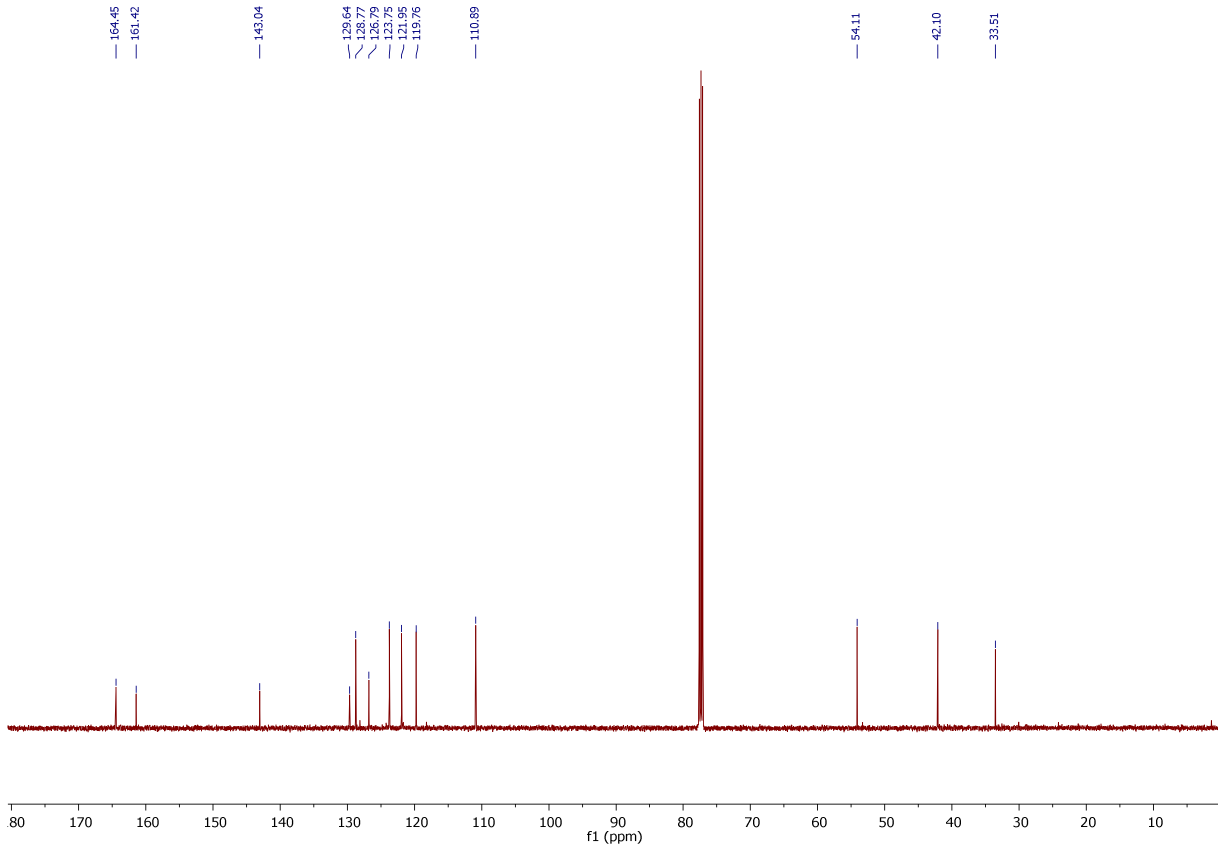
164.45
161.42
143.04
129.64
128.77
126.79
123.75
121.95
119.76
110.89
54.11
42.10
33.51
80
170
160
150
140
130
120
110
100
90
80
70
60
50
40
30
20
10
f1 (ppm)

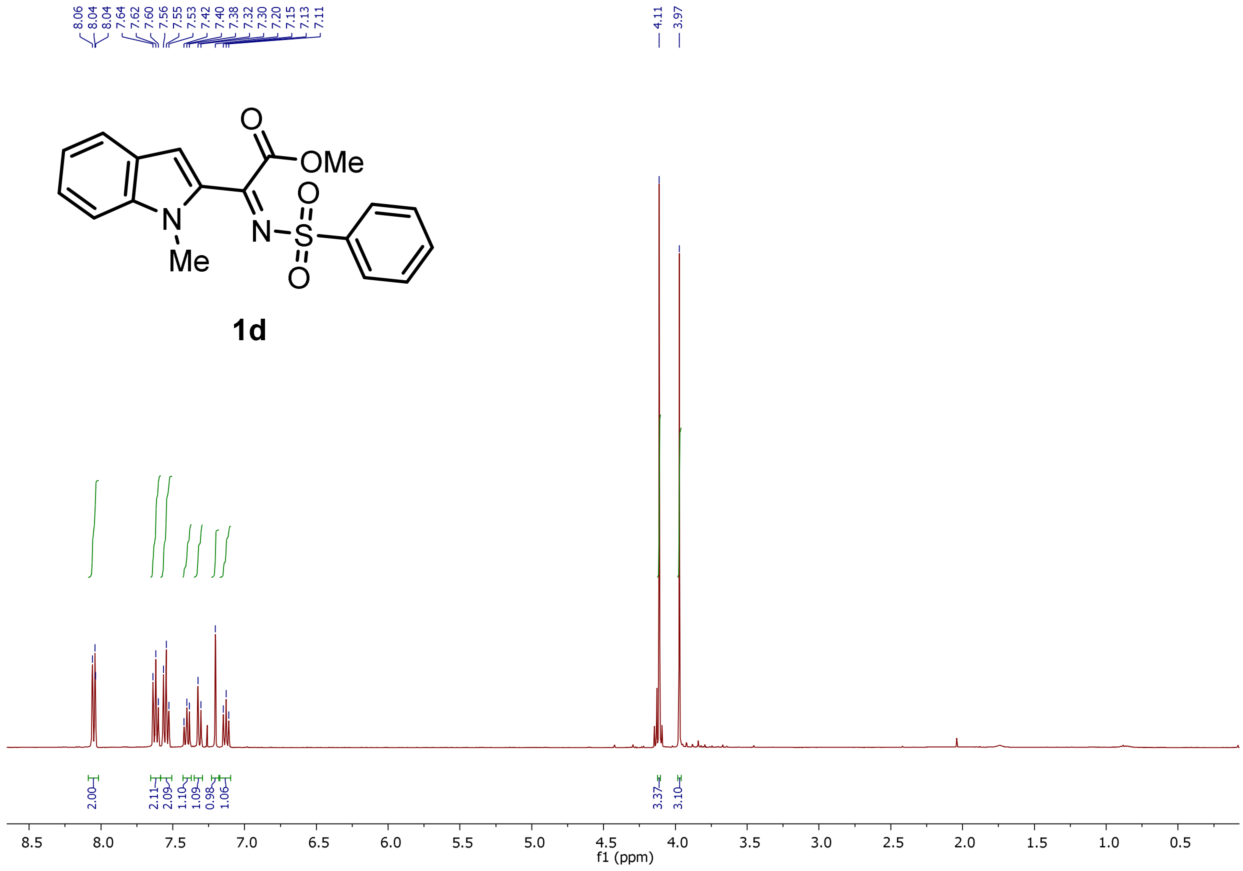
8.06
8.04
8.04
7.64
7.62
7.60
7.56
7.55
7.53
7.42
7.40
7.38
7.32
7.30
7.20
7.15
7.13
7.11
4.11
3.97
OMe
Me
2.00
2.11
2.09
1.10
1.09
0.98
1.06
3.37
3.10
f1 (ppm)

1d

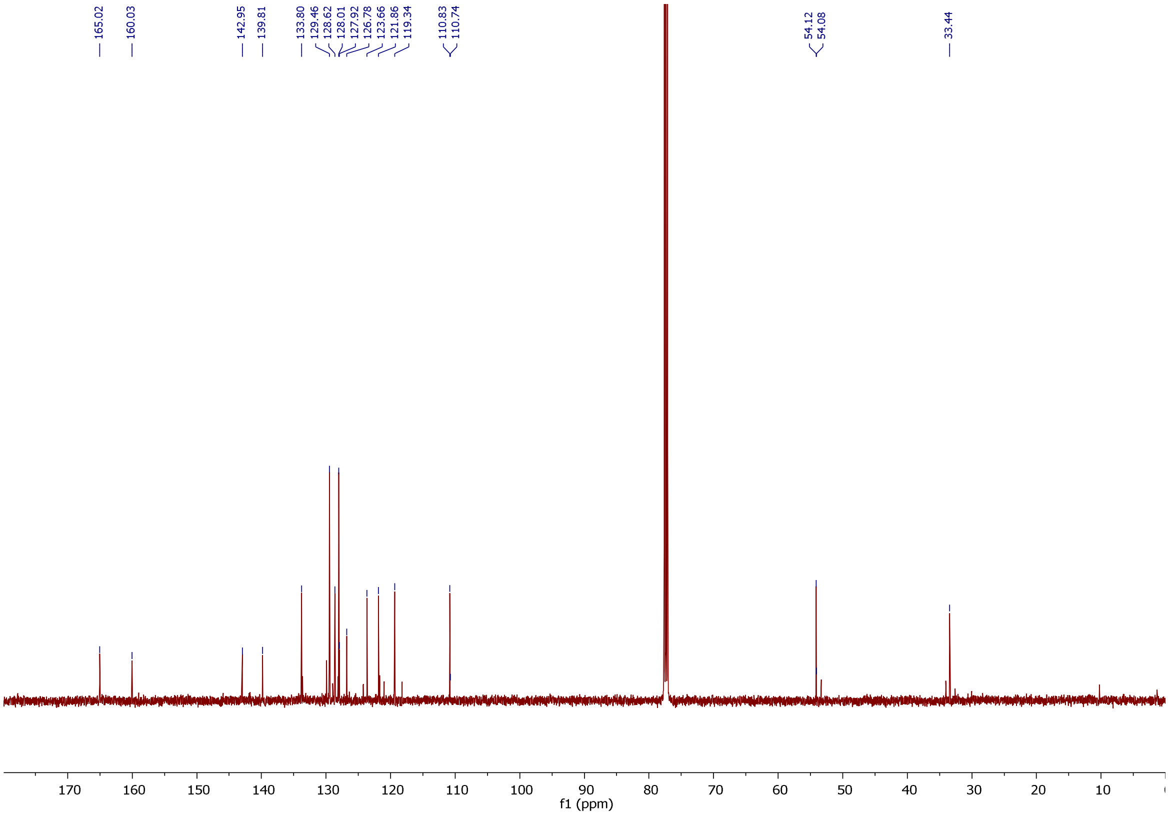
165.02
160.03
142.95
139.81
133.80
129.46
128.62
128.01
127.92
126.78
123.66
121.86
119.34
110.83
110.74
54.12
54.08
33.44
170
160
150
140
130
120
110
100
90
80
70
60
50
40
30
20
10
f1 (ppm)

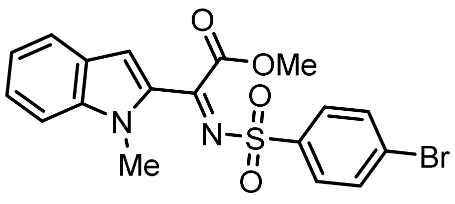
OMe
N
Me
N
S
O
O
O
Br

1e

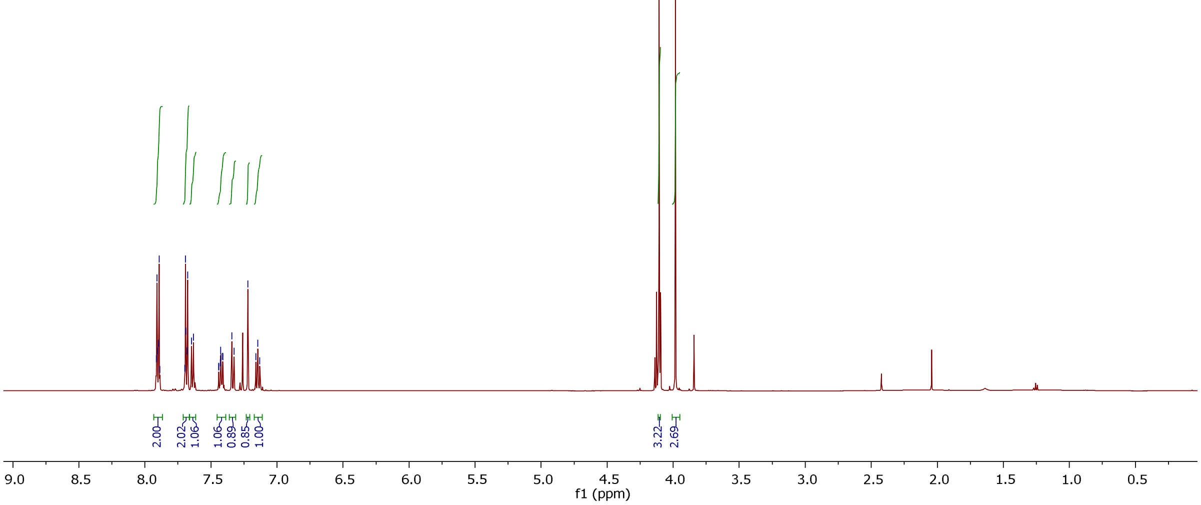
7.91
7.91
7.90
7.89
7.89
7.70
7.69
7.69
7.68
7.68
7.65
7.63
7.44
7.44
7.43
7.43
7.42
7.41
7.41
7.34
7.33
7.22
7.16
7.15
7.13
4.11
3.98
2.00
2.02
1.06
1.06
0.89
0.85
1.00
3.22
2.69
9.0
8.5
8.0
7.5
7.0
6.5
6.0
5.5
5.0
4.5
4.0
3.5
3.0
2.5
2.0
1.5
1.0
0.5
f1 (ppm)

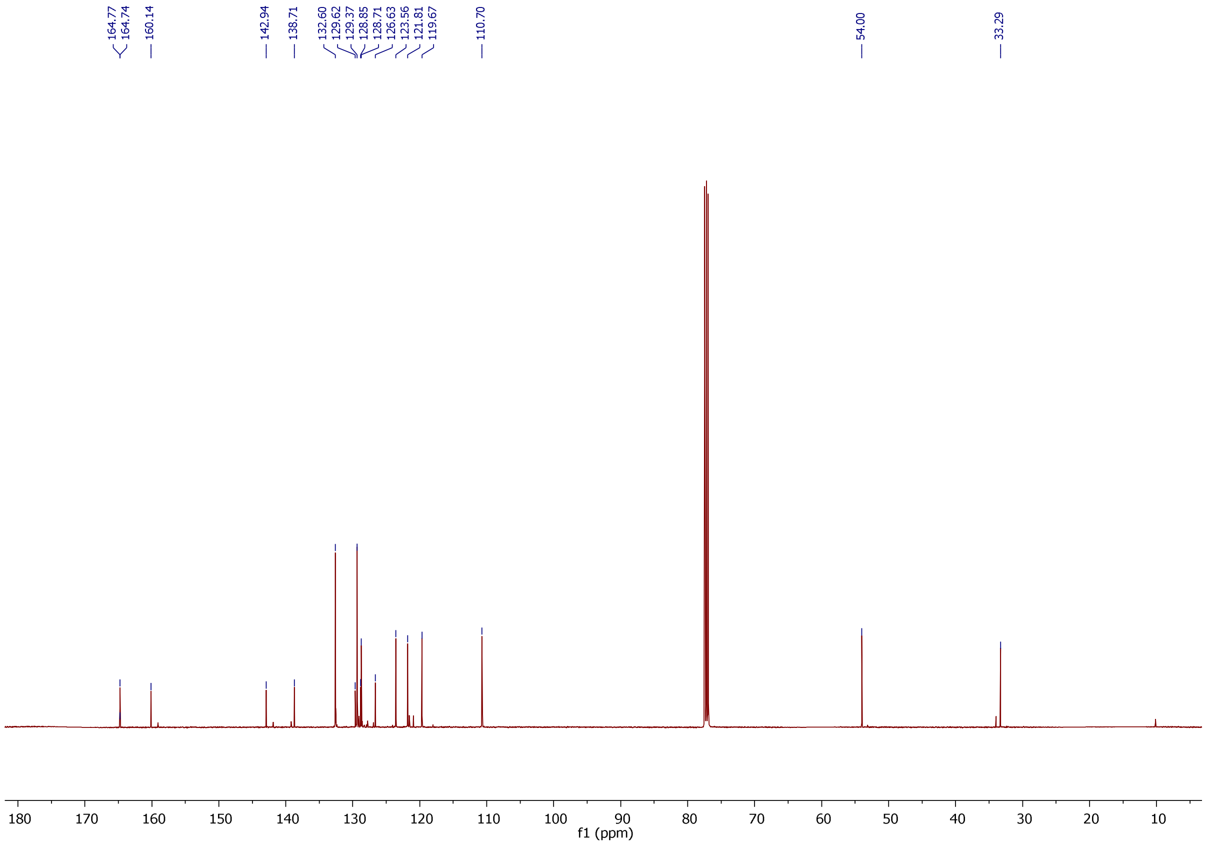
164.77
164.74
160.14
142.94
138.71
132.60
129.62
129.37
128.85
128.71
126.63
123.56
121.81
119.67
110.70
54.00
33.29
180 170 160 150 140 130 120 110 100 90 80 70 60 50 40 30 20 10
f1 (ppm)

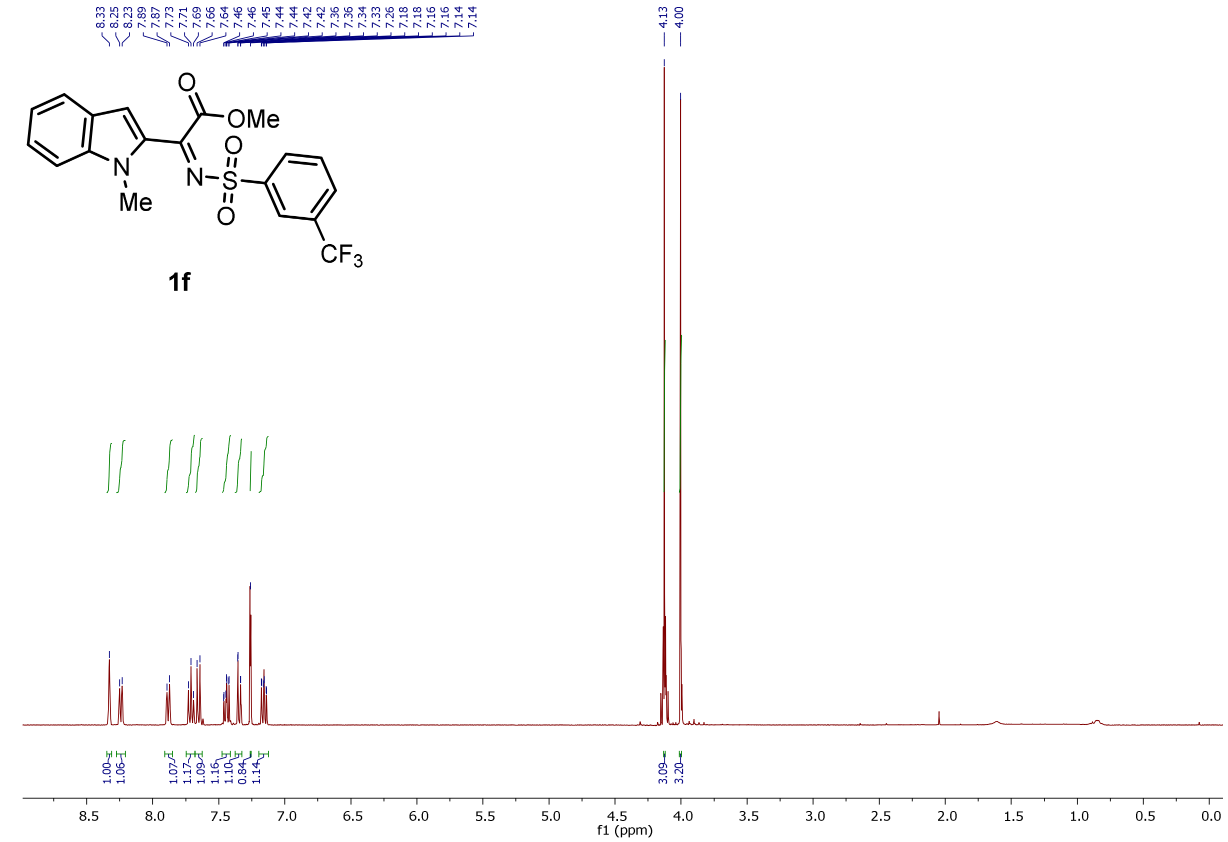

OMe
N
Me
O
S
CF3
1f
f1 (ppm)

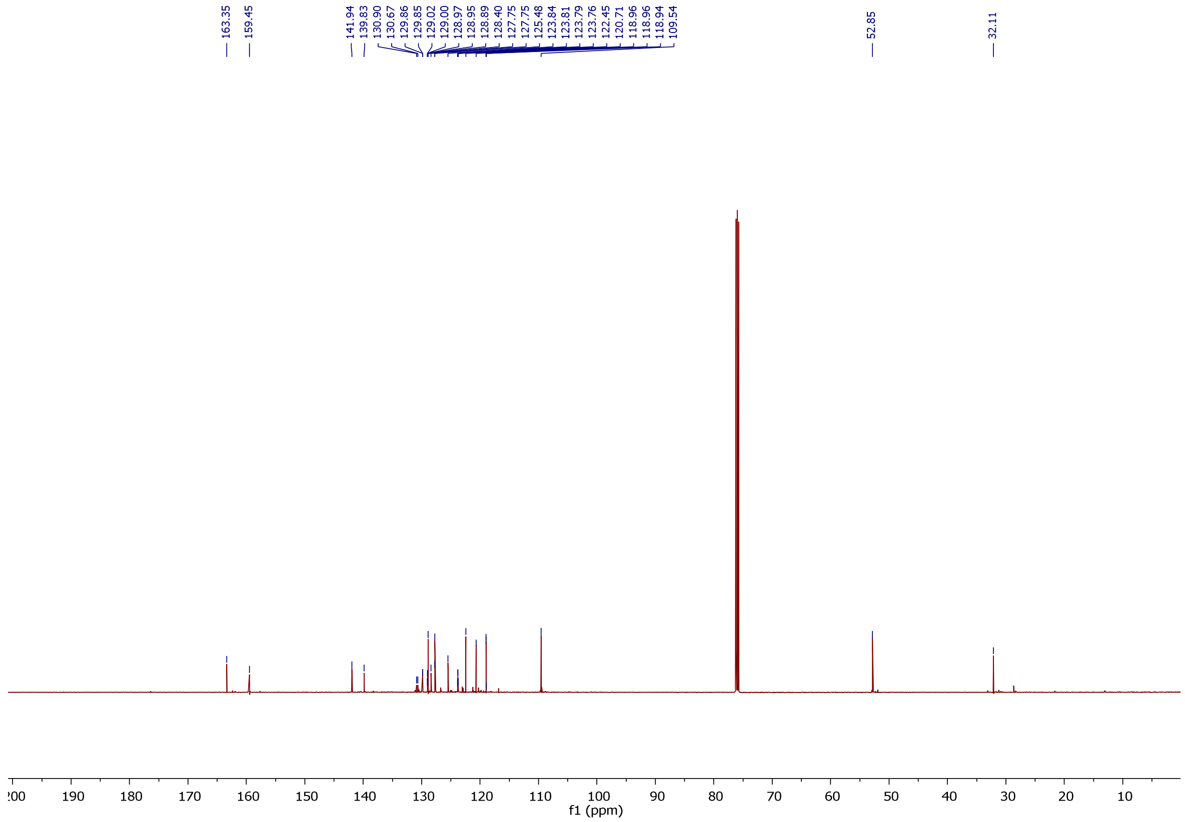
163.35
159.45
141.94
139.83
130.90
130.67
129.86
129.85
129.02
129.00
128.97
128.95
128.89
128.40
127.75
127.75
125.48
123.84
123.81
123.79
123.76
122.45
120.71
118.96
118.96
118.94
109.54
52.85
32.11
200
190
180
170
160
150
140
130
120
110
100
90
80
70
60
50
40
30
20
10
f1 (ppm)

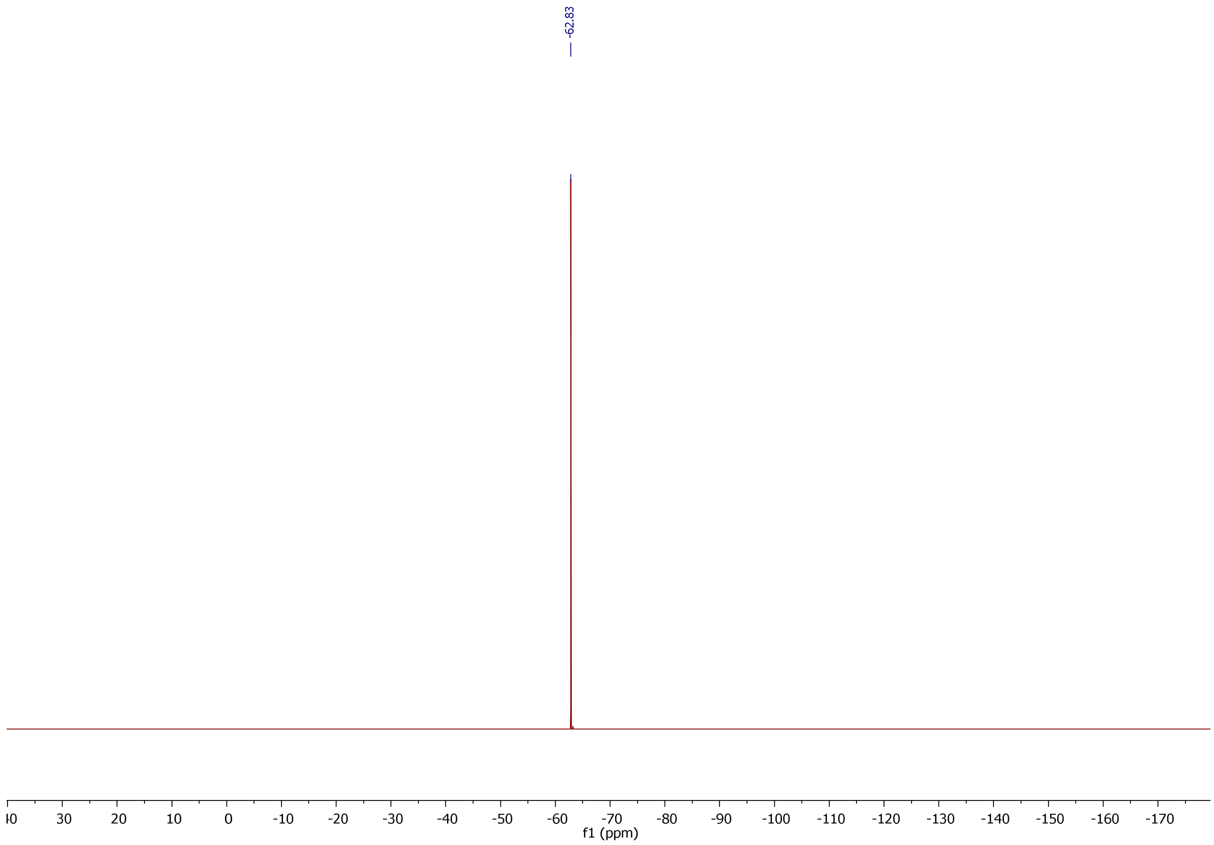
-62.83
40
30
20
10
0
-10
-20
-30
-40
-50
-60
-70
-80
-90
-100
-110
-120
-130
-140
-150
-160
-170
f1 (ppm)

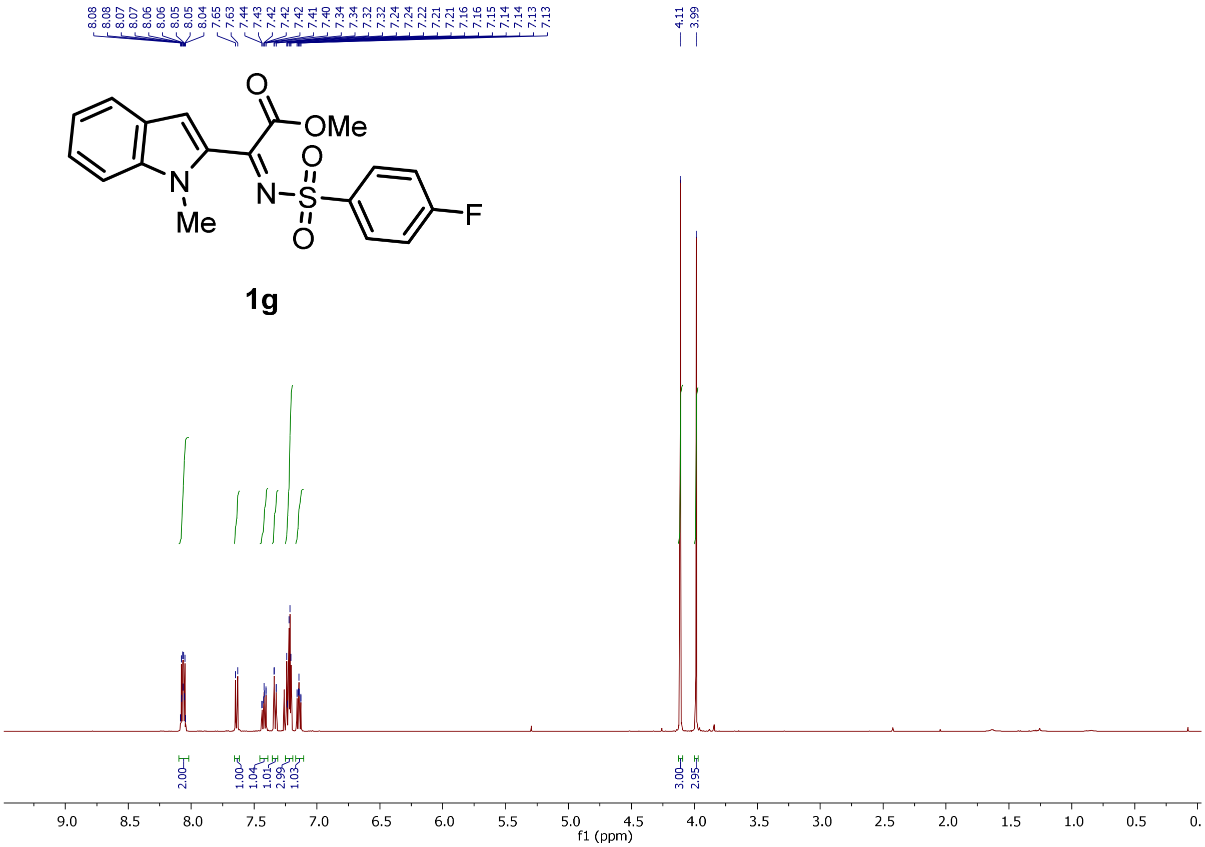

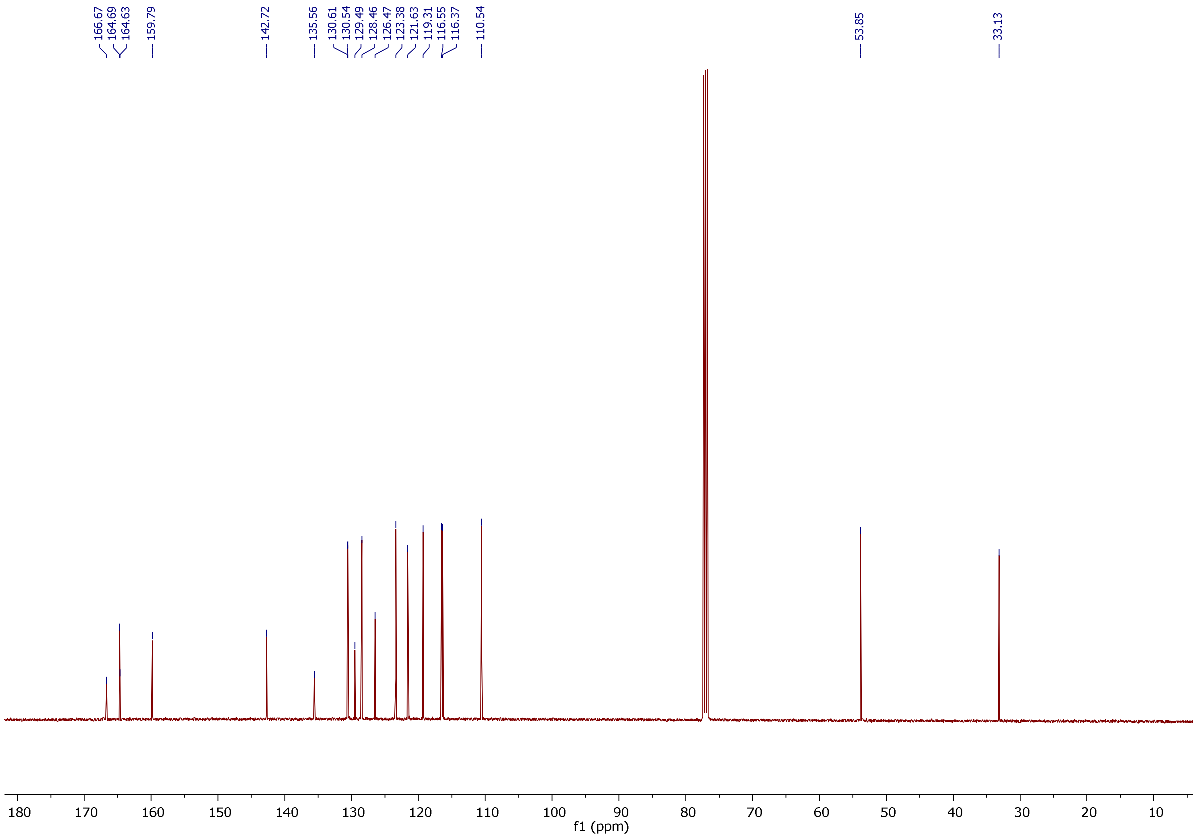
166.67
164.69
164.63
159.79
142.72
135.56
130.61
130.54
129.49
128.46
126.47
123.38
121.63
119.31
116.55
116.37
110.54
53.85
33.13
180 170 160 150 140 130 120 110 100 90 80 70 60 50 40 30 20 10
f1 (ppm)

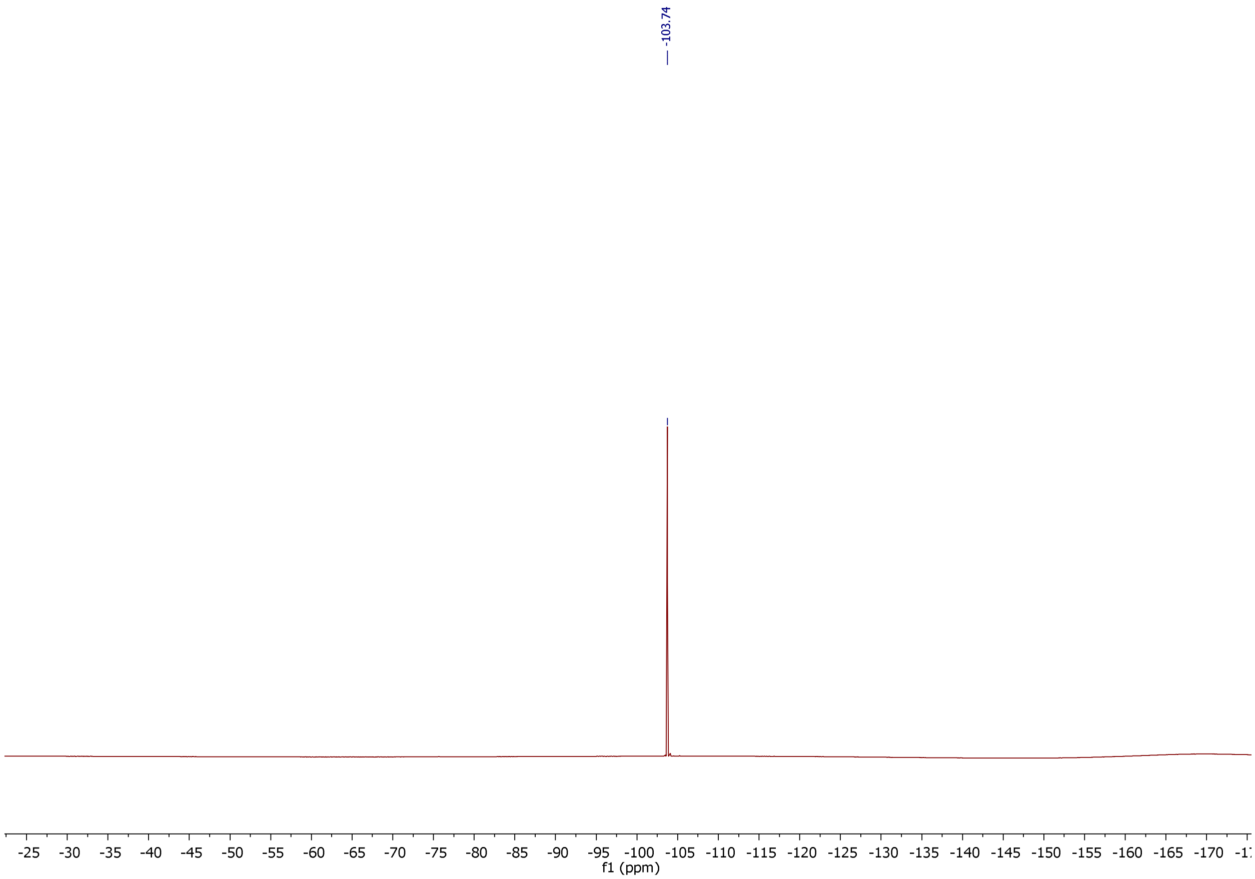
-103.74
-25 -30 -35 -40 -45 -50 -55 -60 -65 -70 -75 -80 -85 -90 -95 -100 -105 -110 -115 -120 -125 -130 -135 -140 -145 -150 -155 -160 -165 -170
f1 (ppm)

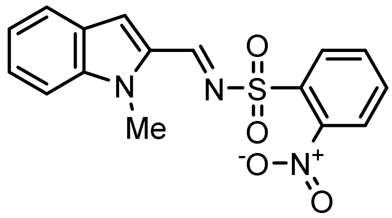

1h

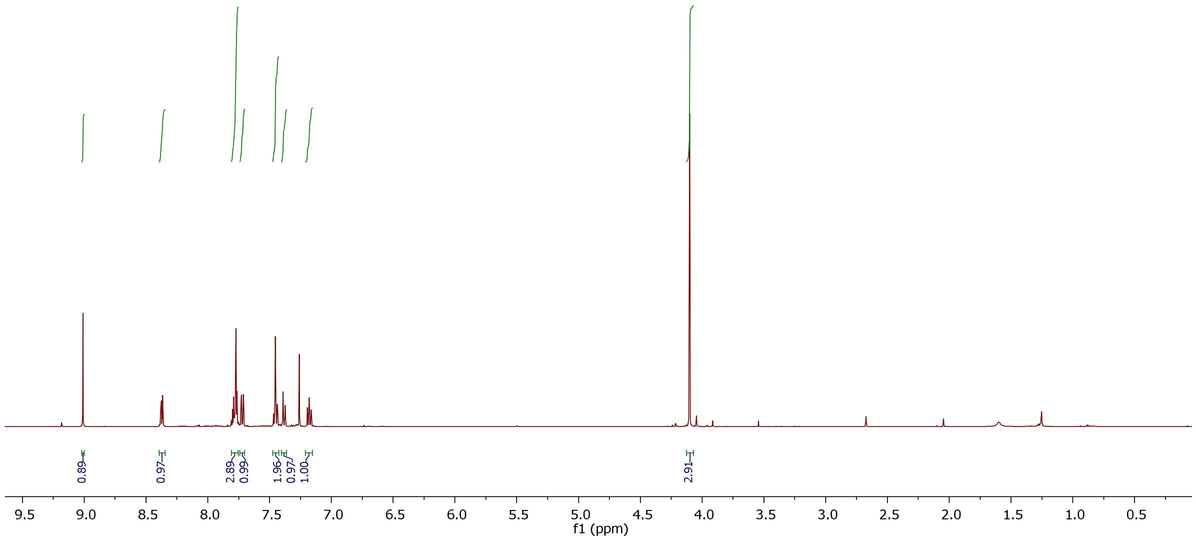
0.89
0.97
2.89
0.99
1.96
0.97
1.00
2.91
9.5
9.0
8.5
8.0
7.5
7.0
6.5
6.0
5.5
5.0
4.5
4.0
3.5
3.0
2.5
2.0
1.5
1.0
0.5
f1 (ppm)

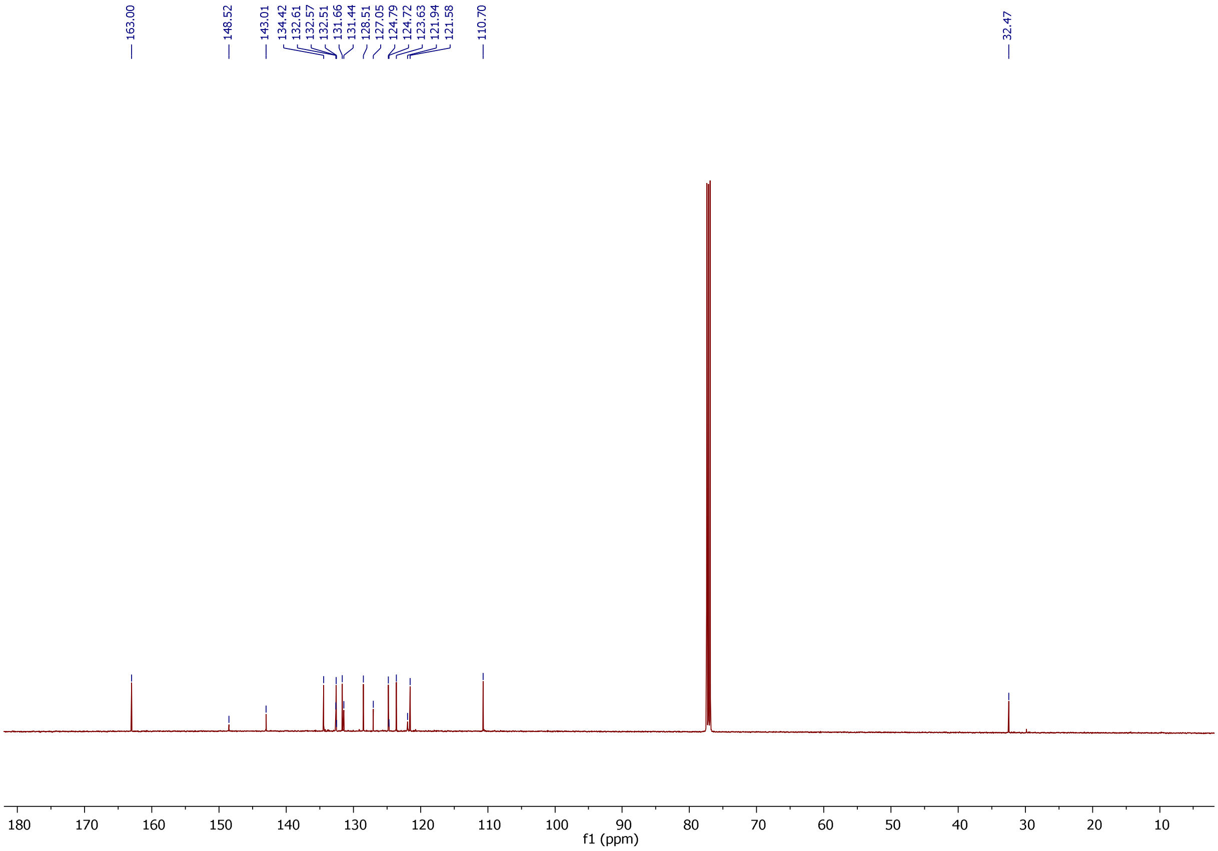
163.00
148.52
143.01
134.42
132.61
132.57
132.51
131.66
131.44
128.51
127.05
124.79
124.72
123.63
121.94
121.58
110.70
32.47
180
170
160
150
140
130
120
110
100
90
80
70
60
50
40
30
20
10
f1 (ppm)

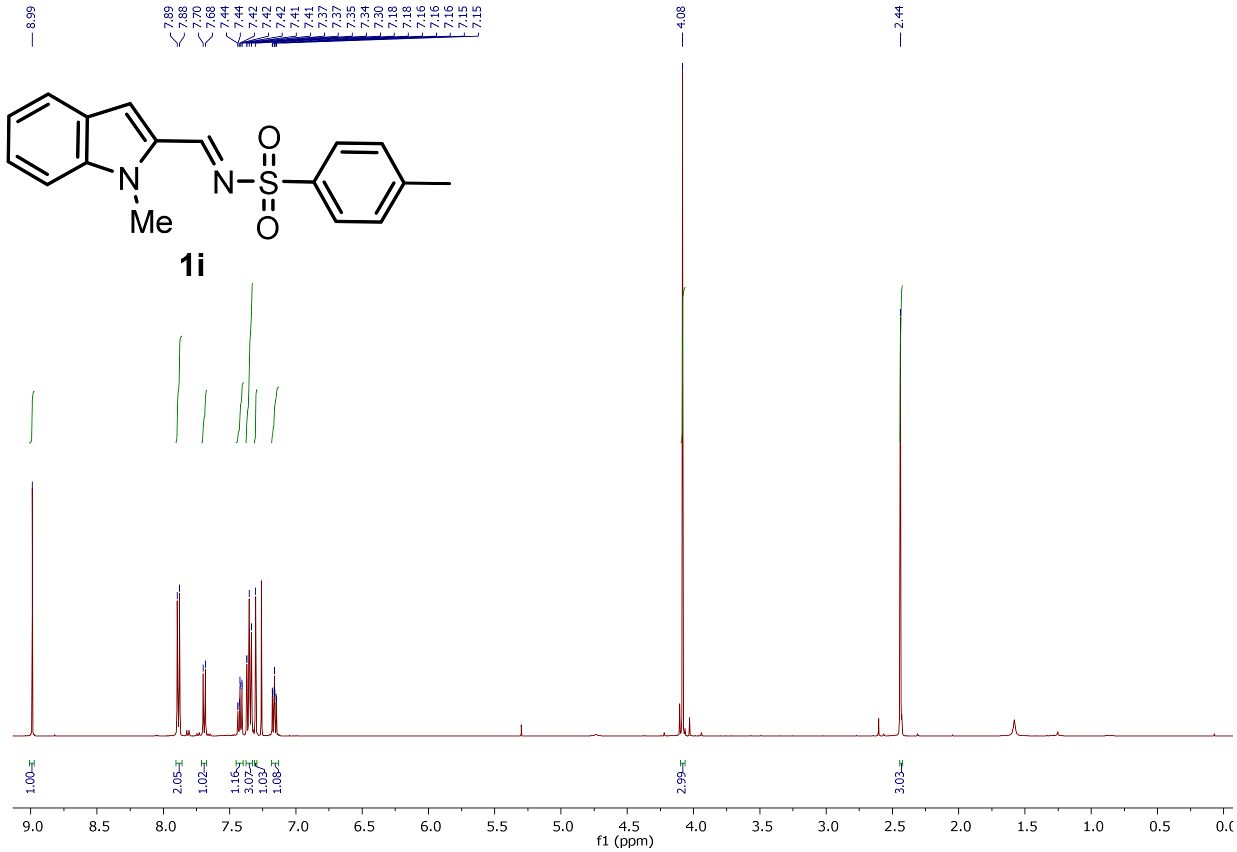
8.99
7.89 7.88 7.70 7.68 7.44 7.44 7.42 7.42 7.42 7.42 7.41 7.41 7.37 7.37 7.35 7.34 7.30 7.18 7.18 7.16 7.16 7.15 7.15
4.08
2.44
N
Me
O
S
O
1i
1.00
2.05
1.02
1.16
3.07
1.03
1.08
2.99
3.03
9.0 8.5 8.0 7.5 7.0 6.5 6.0 5.5 5.0 4.5 4.0 3.5 3.0 2.5 2.0 1.5 1.0 0.5
f1 (ppm)

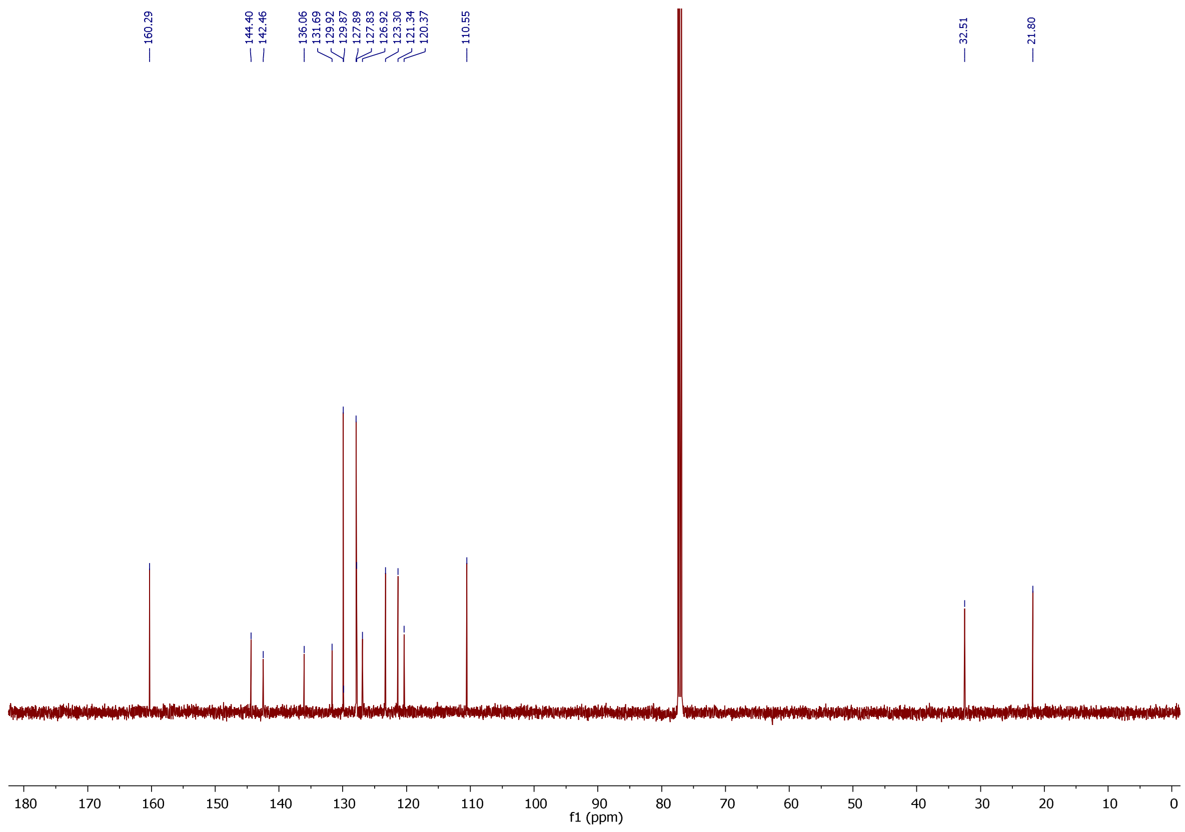
160.29
144.40
142.46
136.06
131.69
129.92
129.87
127.89
127.83
126.92
123.30
121.34
120.37
110.55
32.51
21.80
180
170
160
150
140
130
120
110
100
90
80
70
60
50
40
30
20
10
0
f1 (ppm)

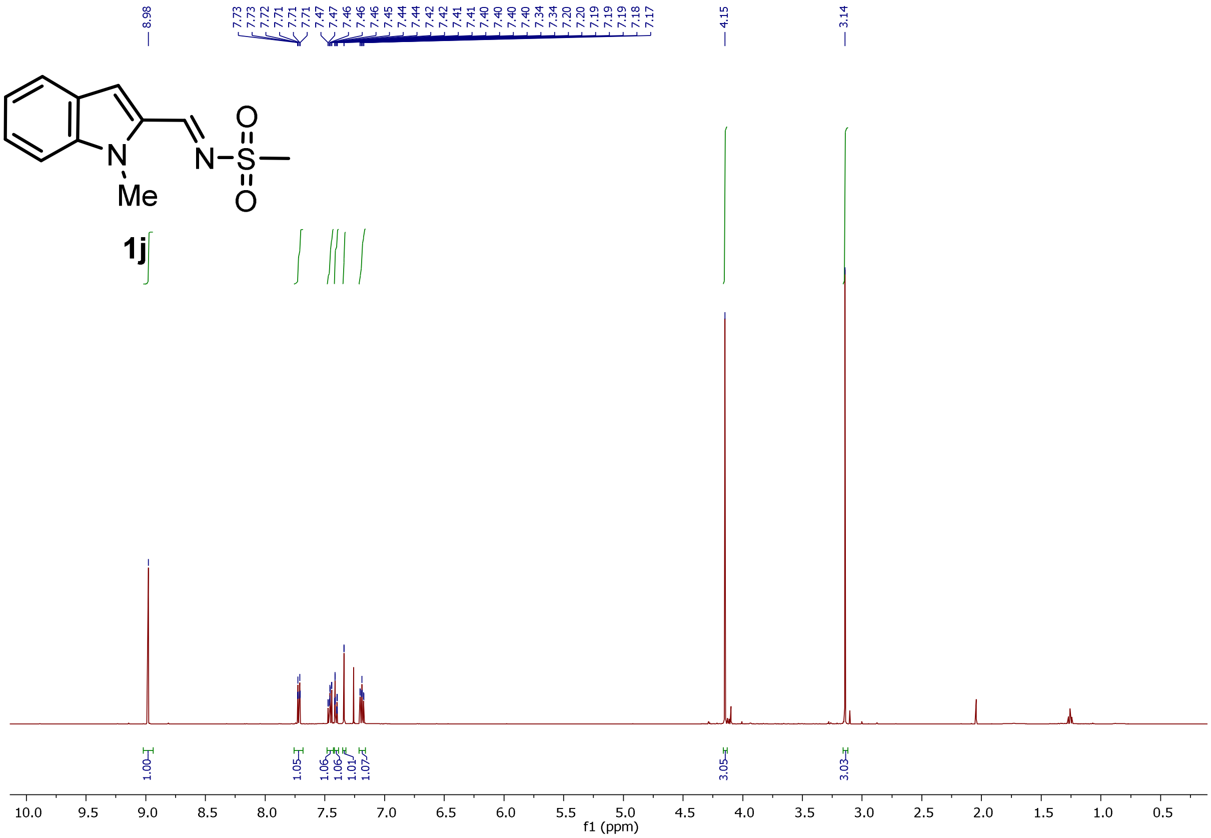
8.98
7.73
7.73
7.72
7.71
7.71
7.71
7.47
7.47
7.46
7.46
7.46
7.45
7.44
7.44
7.42
7.42
7.41
7.41
7.40
7.40
7.40
7.40
7.34
7.34
7.20
7.20
7.19
7.19
7.19
7.18
7.17
4.15
3.14
N
Me
N
S
O
O
1j
1.00
1.05
1.06
1.06
1.01
1.07
3.05
3.03
10.0 9.5 9.0 8.5 8.0 7.5 7.0 6.5 6.0 5.5 5.0 4.5 4.0 3.5 3.0 2.5 2.0 1.5 1.0 0.5
f1 (ppm)

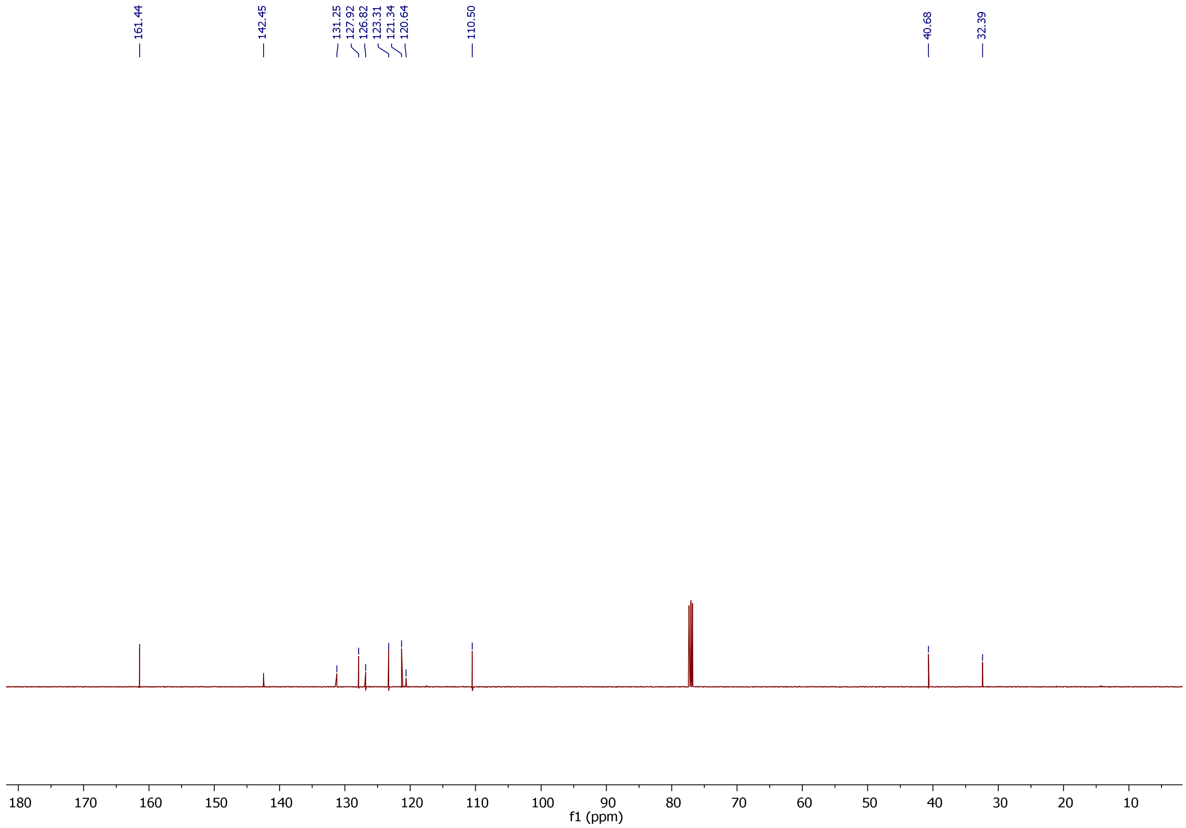
161.44
142.45
131.25
127.92
126.82
123.31
121.34
120.64
110.50
40.68
32.39
180
170
160
150
140
130
120
110
100
90
80
70
60
50
40
30
20
10
f1 (ppm)

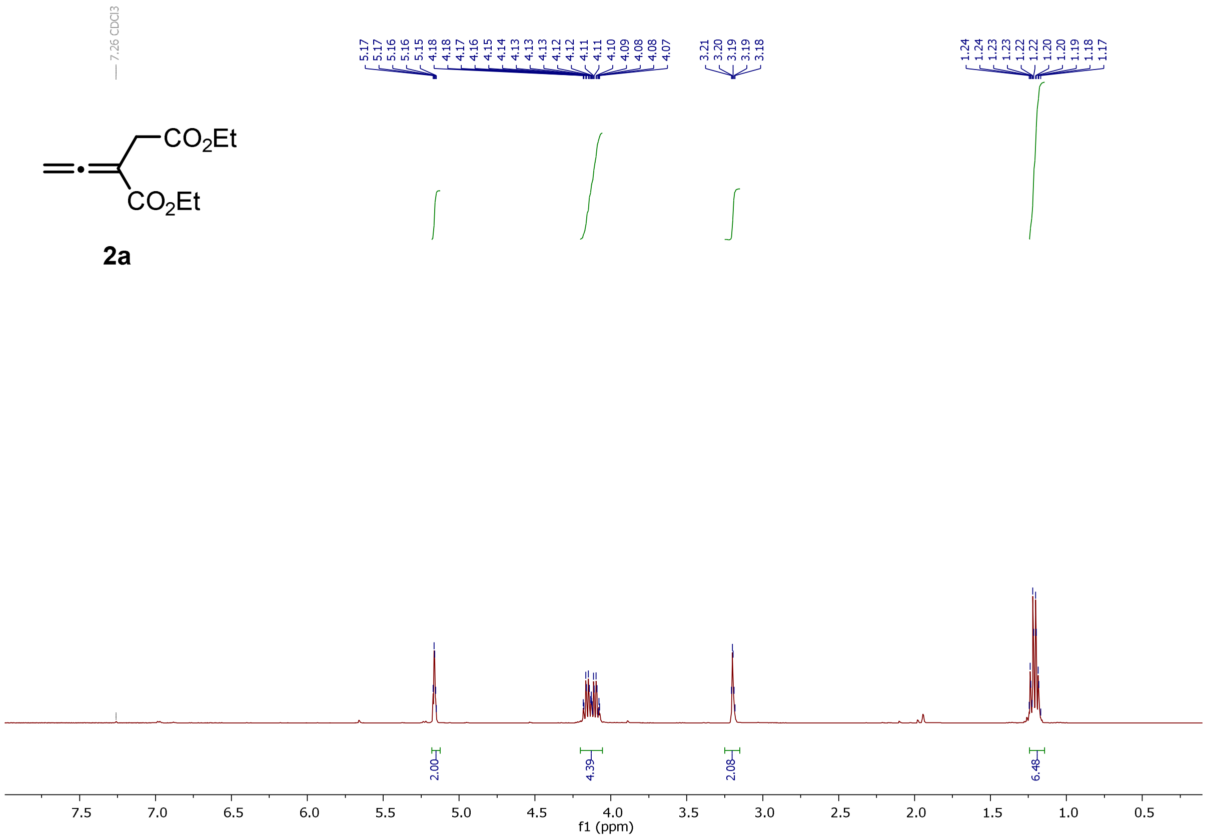
7.26 CDCl3
5.17
5.17
5.16
5.16
5.15
4.18
4.18
4.17
4.16
4.15
4.14
4.13
4.13
4.13
4.12
4.12
4.11
4.11
4.10
4.09
4.08
4.07
3.21
3.20
3.19
3.19
3.18
1.24
1.24
1.23
1.23
1.22
1.22
1.20
1.20
1.19
1.18
1.17
CO2Et
CO2Et
2a
2.00
4.39
2.08
6.48
7.5
7.0
6.5
6.0
5.5
5.0
4.5
4.0
3.5
3.0
2.5
2.0
1.5
1.0
0.5
f1 (ppm)

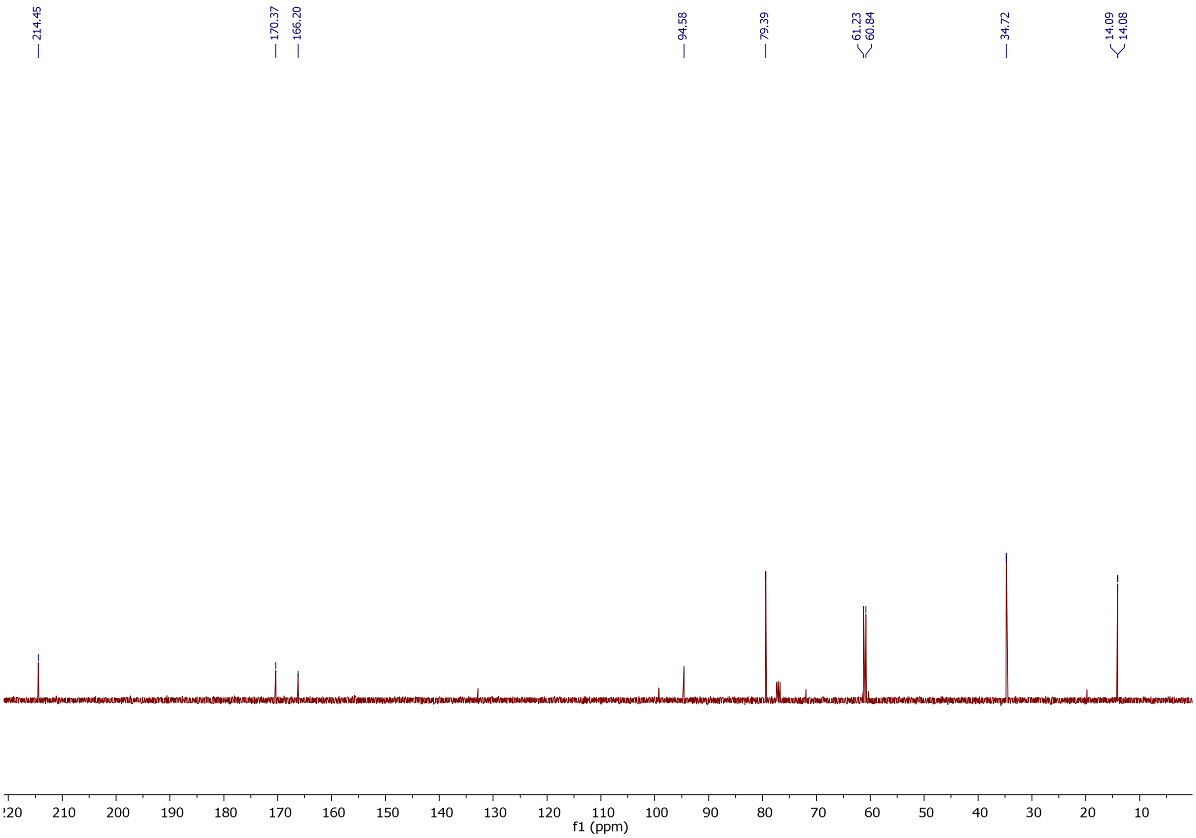
214.45
170.37
166.20
94.58
79.39
61.23
60.84
34.72
14.09
14.08
f1 (ppm)

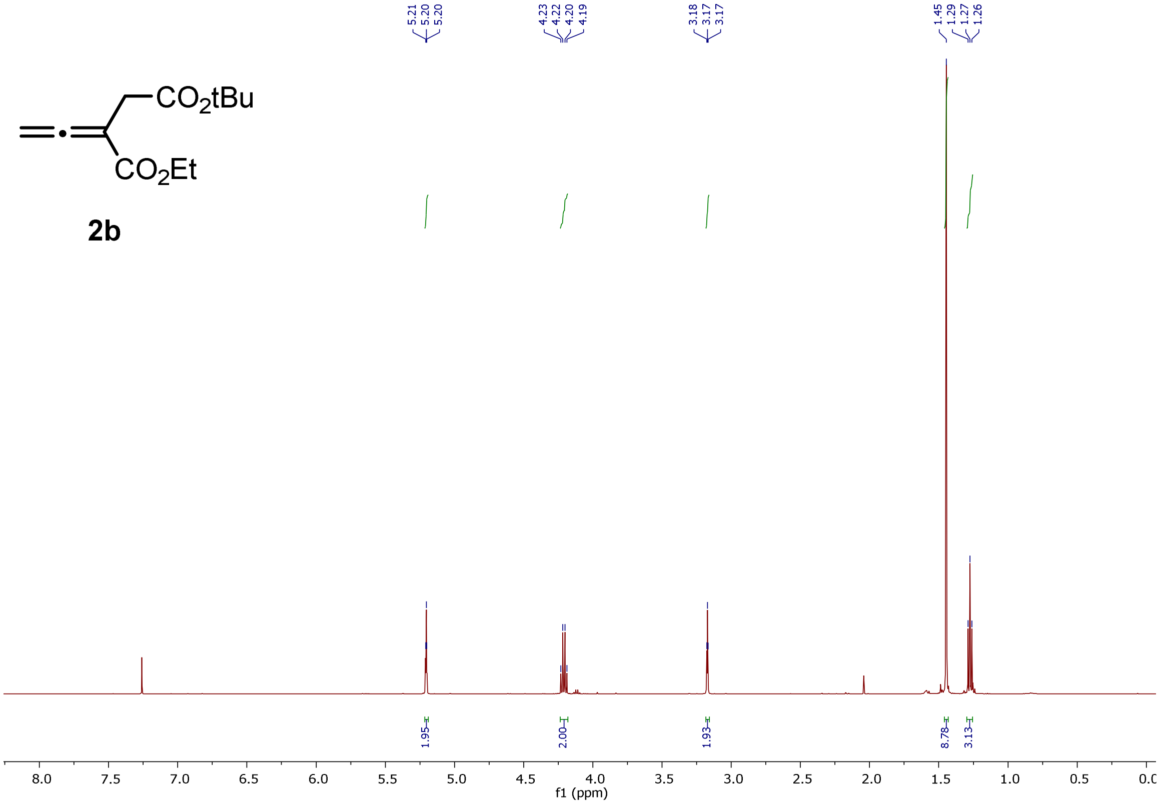
CO2tBu
CO2Et
5.21
5.20
5.20
4.23
4.22
4.20
4.19
3.18
3.17
3.17
1.45
1.29
1.27
1.26
1.95
2.00
1.93
8.78
3.13
f1 (ppm)

2b

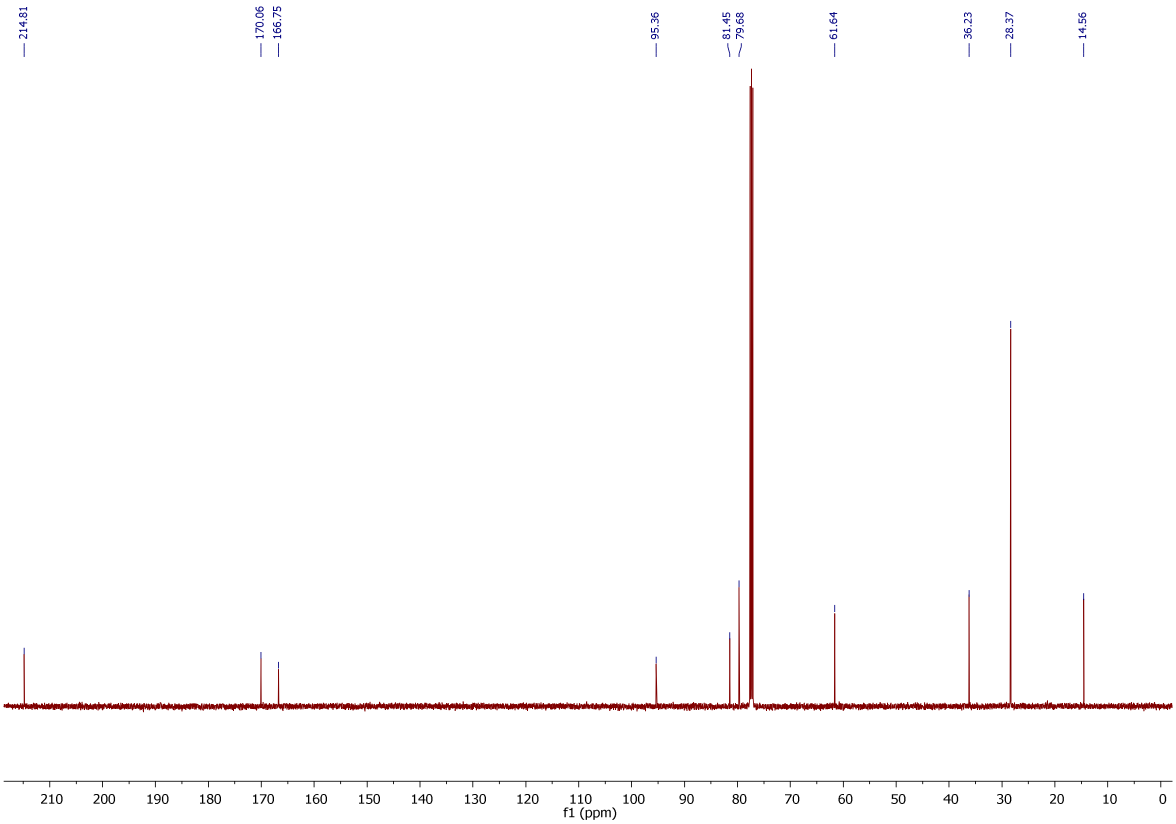
214.81
170.06
166.75
95.36
81.45
79.68
61.64
36.23
28.37
14.56
f1 (ppm)

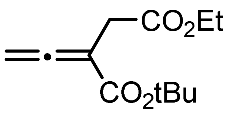
CO2Et
CO2tBu

2c

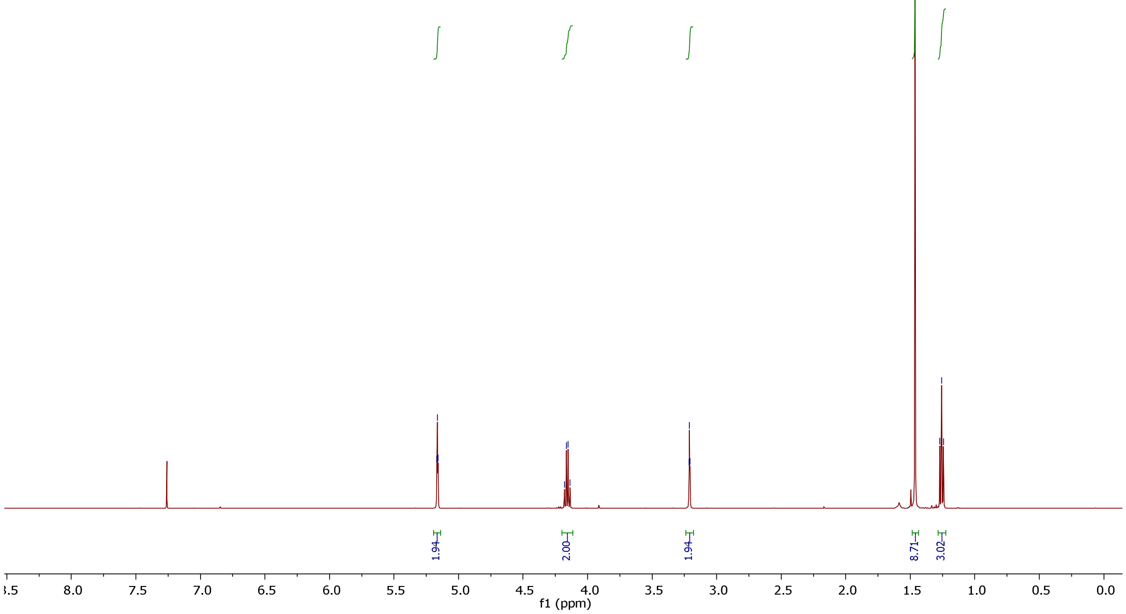
5.17
5.16
5.16
4.18
4.16
4.15
4.14
3.21
3.21
3.21
1.46
1.27
1.26
1.24
1.94
2.00
1.94
8.71
3.02
f1 (ppm)

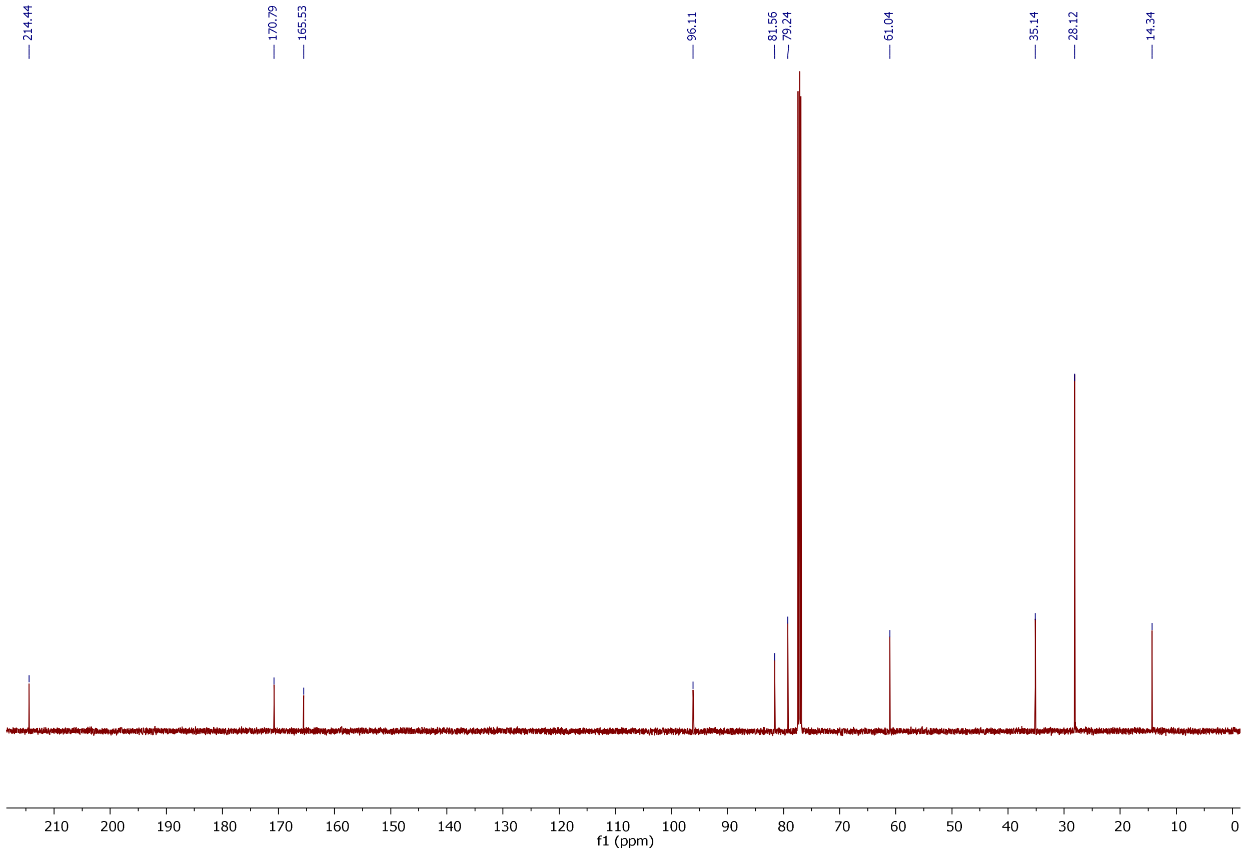
214.44
170.79
165.53
96.11
81.56
79.24
61.04
35.14
28.12
14.34
210 200 190 180 170 160 150 140 130 120 110 100 90 80 70 60 50 40 30 20 10 0
f1 (ppm)

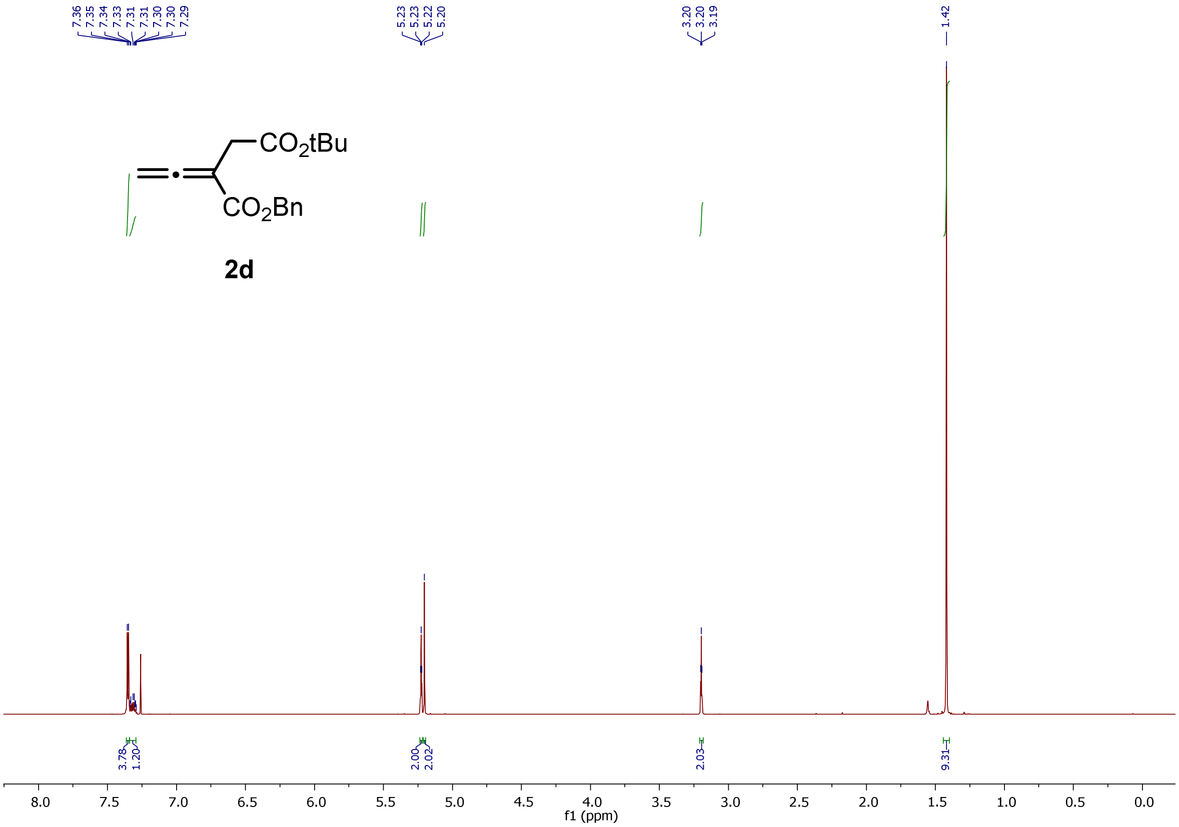
7.36
7.35
7.34
7.33
7.31
7.31
7.30
7.30
7.29
5.23
5.23
5.22
5.20
3.20
3.20
3.19
1.42
CO2tBu
CO2Bn
2d
3.78
1.20
2.00
2.02
2.03
9.31
8.0
7.5
7.0
6.5
6.0
5.5
5.0
4.5
4.0
3.5
3.0
2.5
2.0
1.5
1.0
0.5
0.0
f1 (ppm)

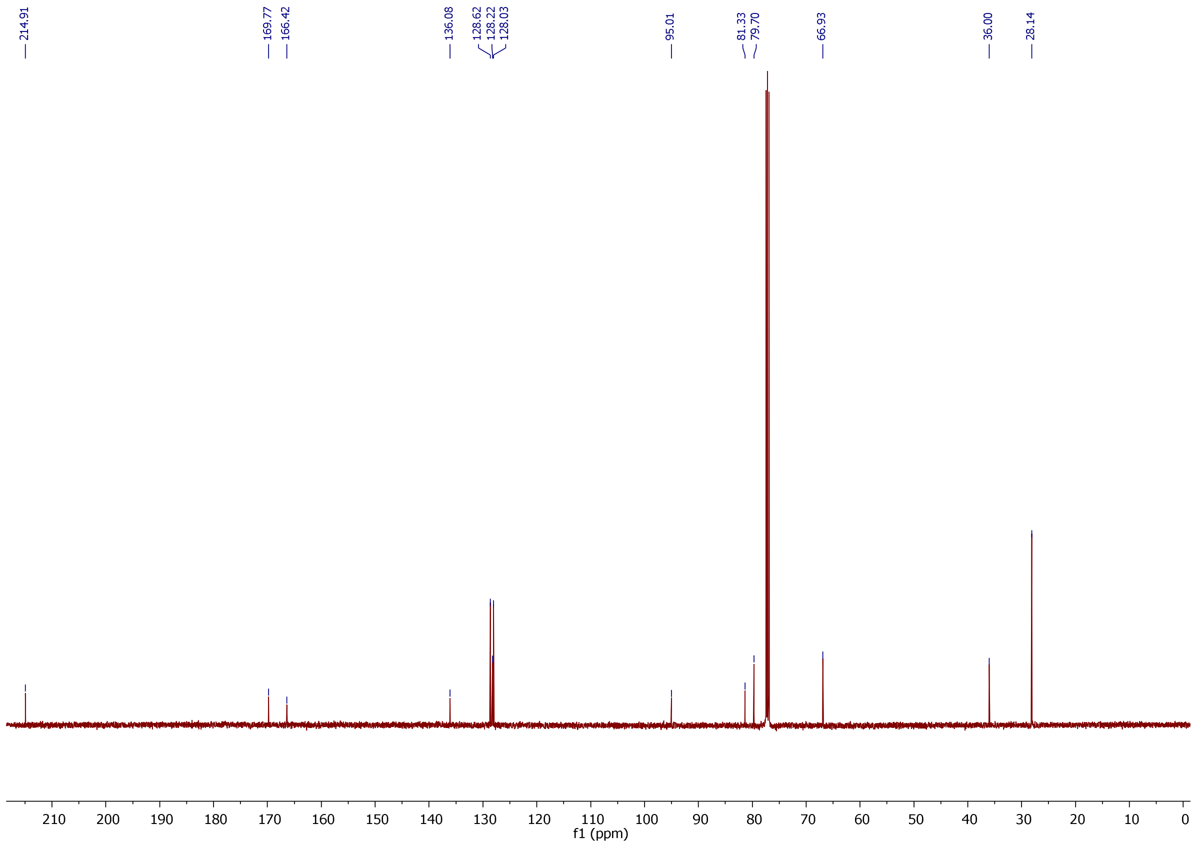

214.91
169.77
166.42
136.08
128.62
128.22
128.03
95.01
81.33
79.70
66.93
36.00
28.14
210 200 190 180 170 160 150 140 130 120 110 100 90 80 70 60 50 40 30 20 10 0
f1 (ppm)

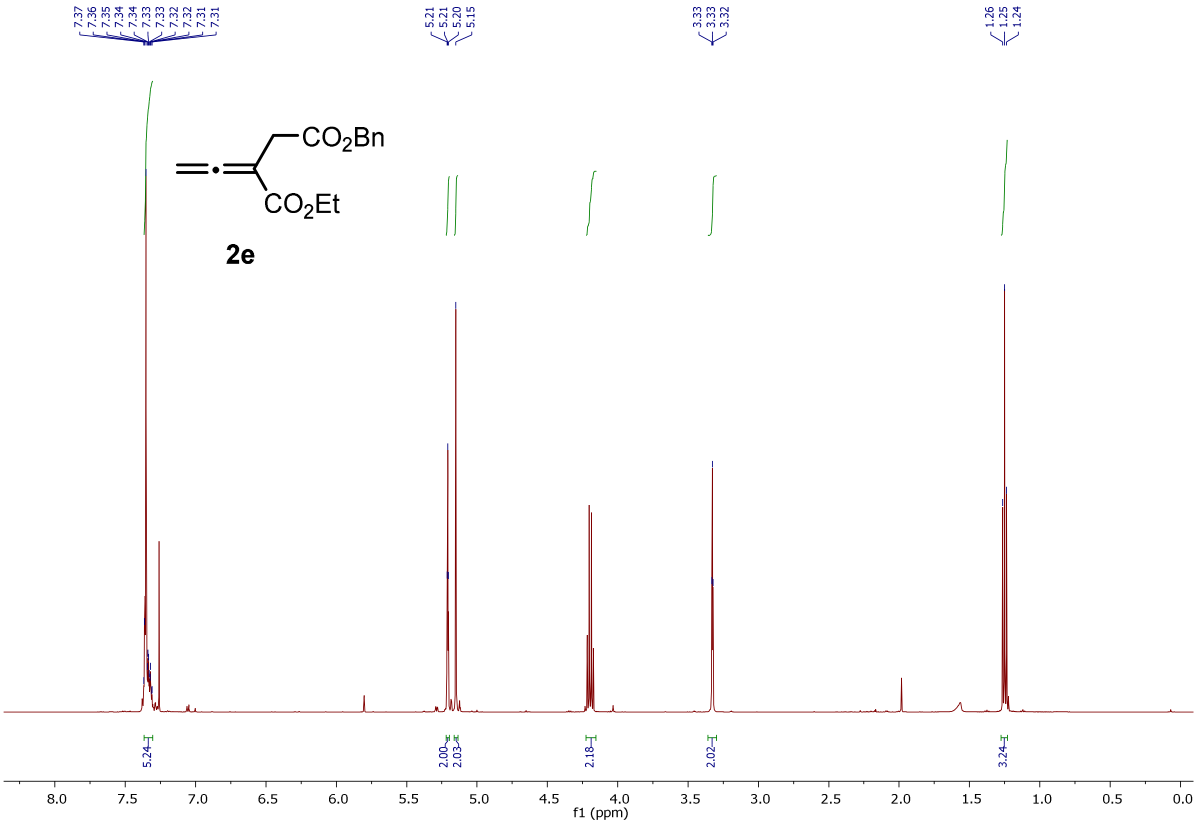
7.37
7.36
7.35
7.34
7.34
7.33
7.33
7.32
7.32
7.31
7.31
5.21
5.21
5.20
5.15
3.33
3.33
3.32
1.26
1.25
1.24
CO2Bn
CO2Et
2e
5.24
2.00
2.03
2.18
2.02
3.24
8.0
7.5
7.0
6.5
6.0
5.5
5.0
4.5
4.0
3.5
3.0
2.5
2.0
1.5
1.0
0.5
0.0
f1 (ppm)

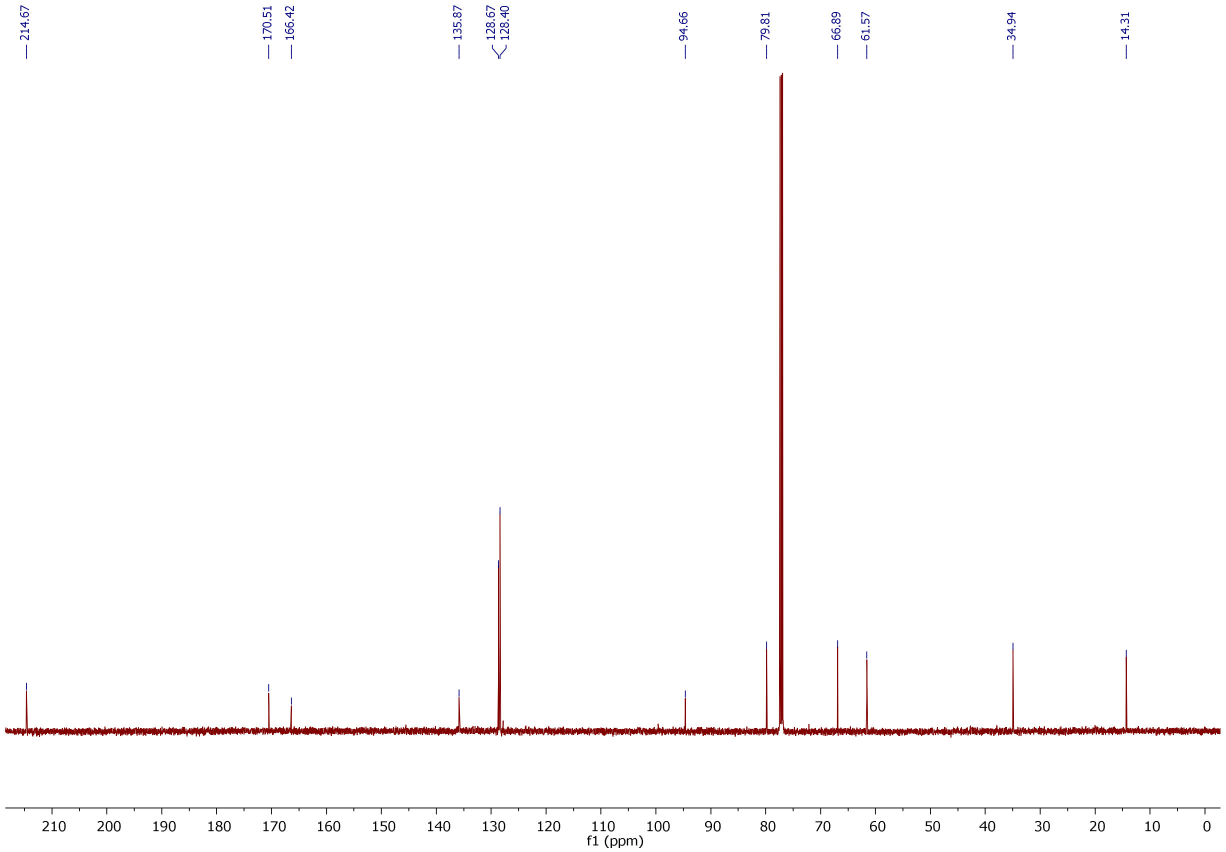
214.67
170.51
166.42
135.87
128.67
128.40
94.66
79.81
66.89
61.57
34.94
14.31
210 200 190 180 170 160 150 140 130 120 110 100 90 80 70 60 50 40 30 20 10 0
f1 (ppm)

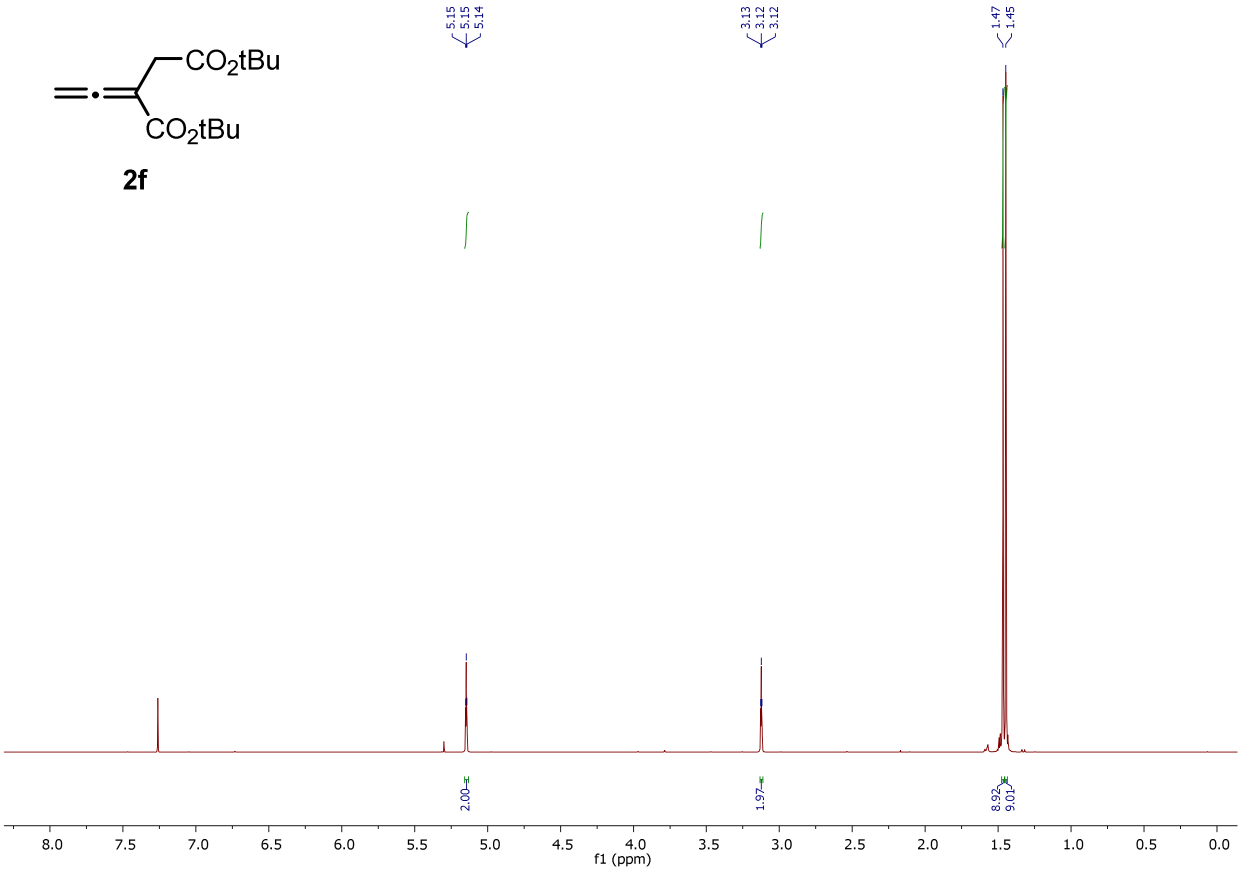
CO2tBu
CO2tBu
2f
5.15
5.15
5.14
3.13
3.12
3.12
1.47
1.45
2.00
1.97
8.92
9.01
8.0 7.5 7.0 6.5 6.0 5.5 5.0 4.5 4.0 3.5 3.0 2.5 2.0 1.5 1.0 0.5 0.0
f1 (ppm)

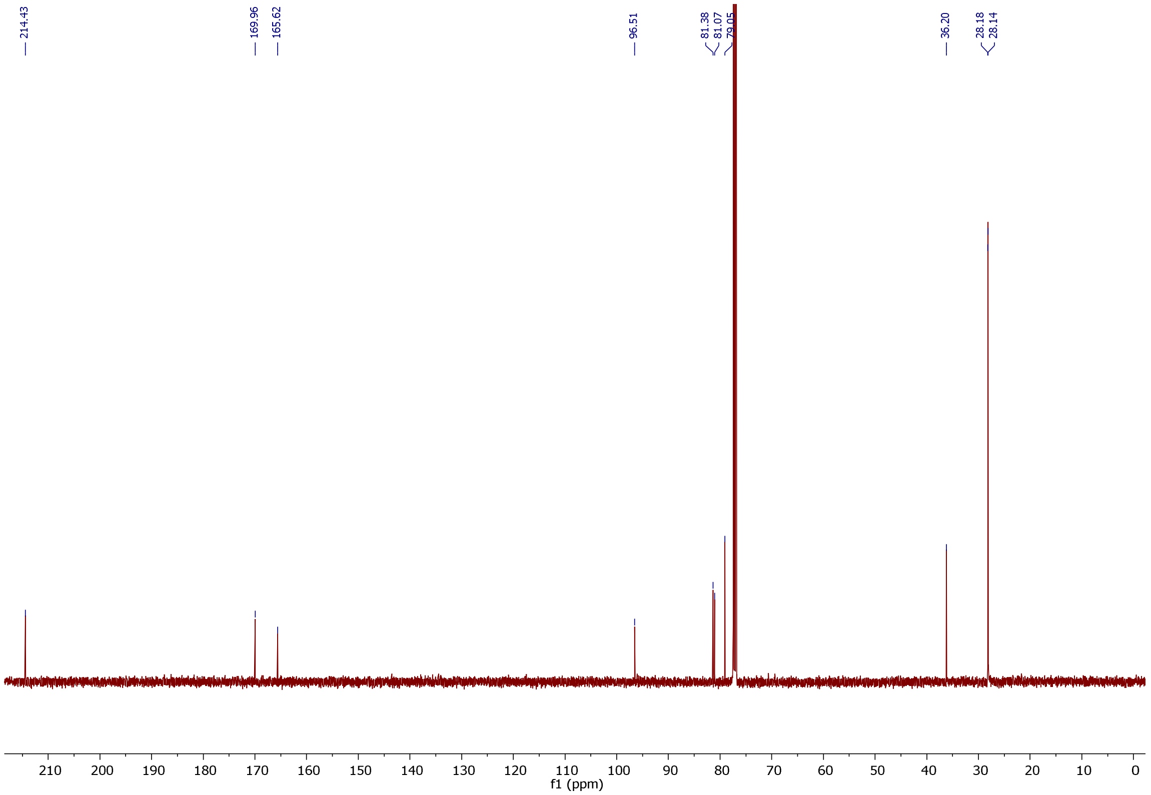

214.43
169.96
165.62
96.51
81.38
81.07
79.05
36.20
28.18
28.14
210 200 190 180 170 160 150 140 130 120 110 100 90 80 70 60 50 40 30 20 10 0
f1 (ppm)

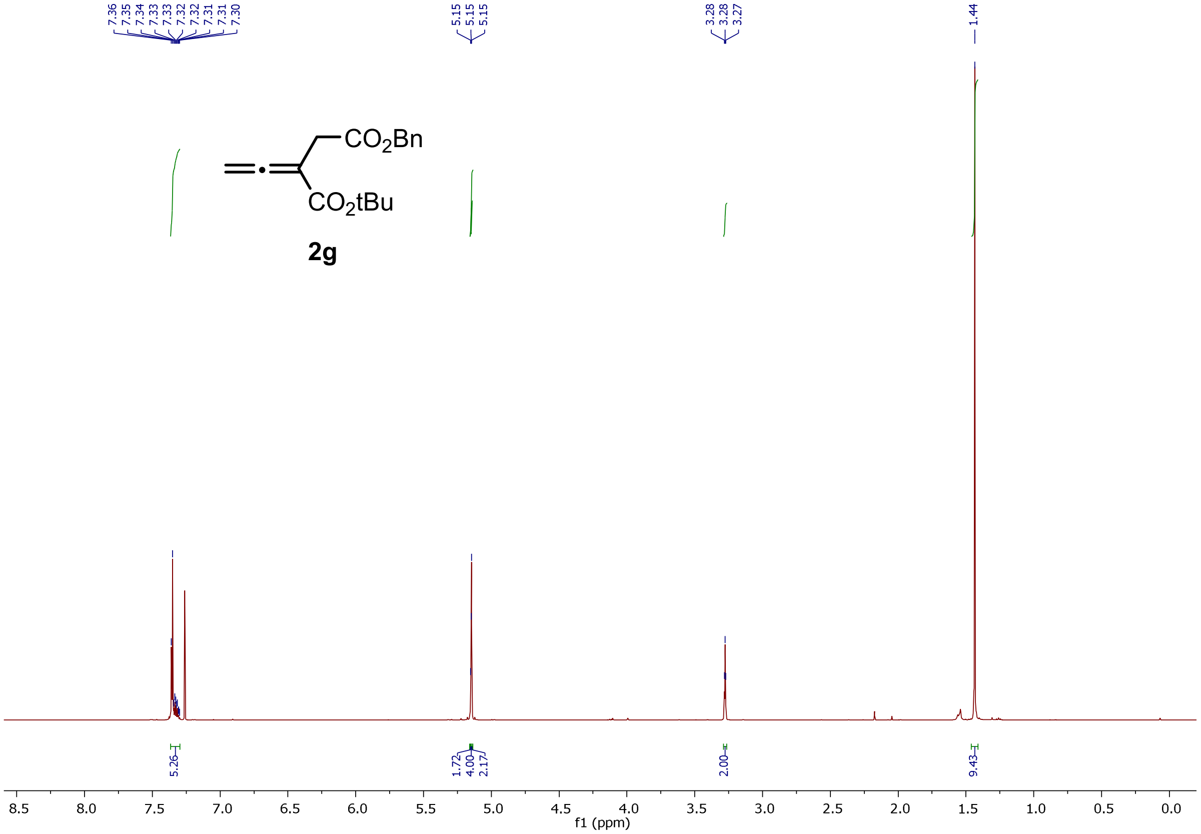

7.36
7.35
7.34
7.33
7.33
7.32
7.32
7.31
7.31
7.30
5.15
5.15
5.15
3.28
3.28
3.27
1.44
CO_2Bn
CO_2tBu
2g
5.26
1.72
4.00
2.17
2.00
9.43
8.5 8.0 7.5 7.0 6.5 6.0 5.5 5.0 4.5 4.0 3.5 3.0 2.5 2.0 1.5 1.0 0.5 0.0
f1 (ppm)

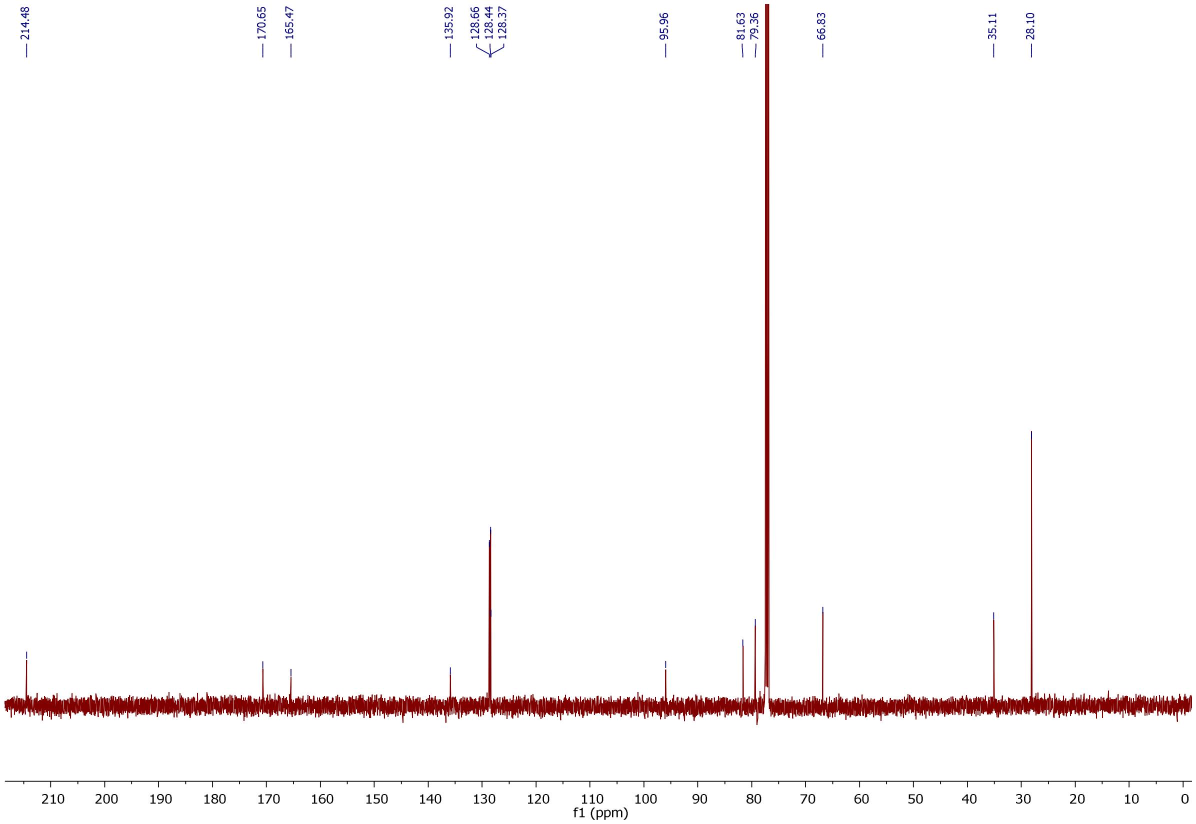
214.48
170.65
165.47
135.92
128.66
128.44
128.37
95.96
81.63
79.36
66.83
35.11
28.10
210 200 190 180 170 160 150 140 130 120 110 100 90 80 70 60 50 40 30 20 10 0
f1 (ppm)

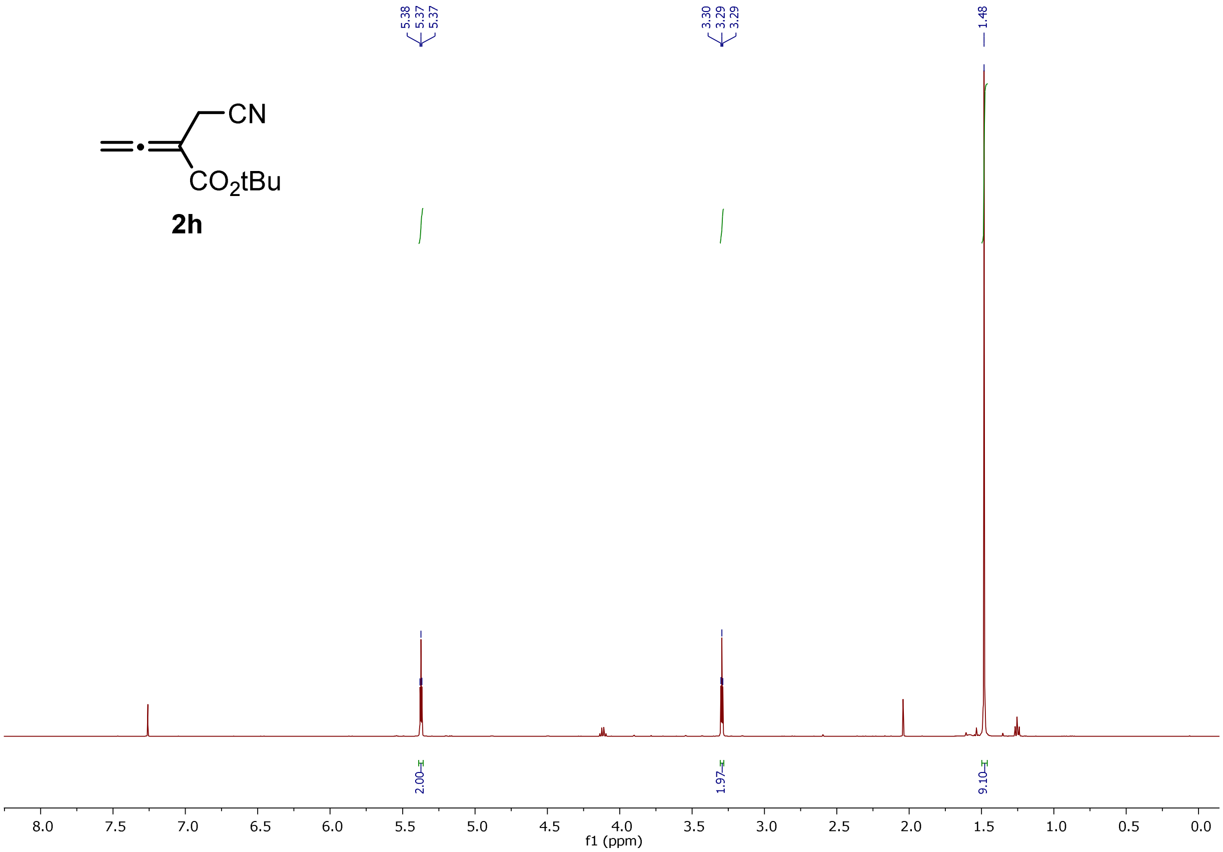

2h
5.38
5.37
5.37
3.30
3.29
3.29
1.48
2.00
1.97
9.10
8.0
7.5
7.0
6.5
6.0
5.5
5.0
4.5
4.0
3.5
3.0
2.5
2.0
1.5
1.0
0.5
0.0
f1 (ppm)

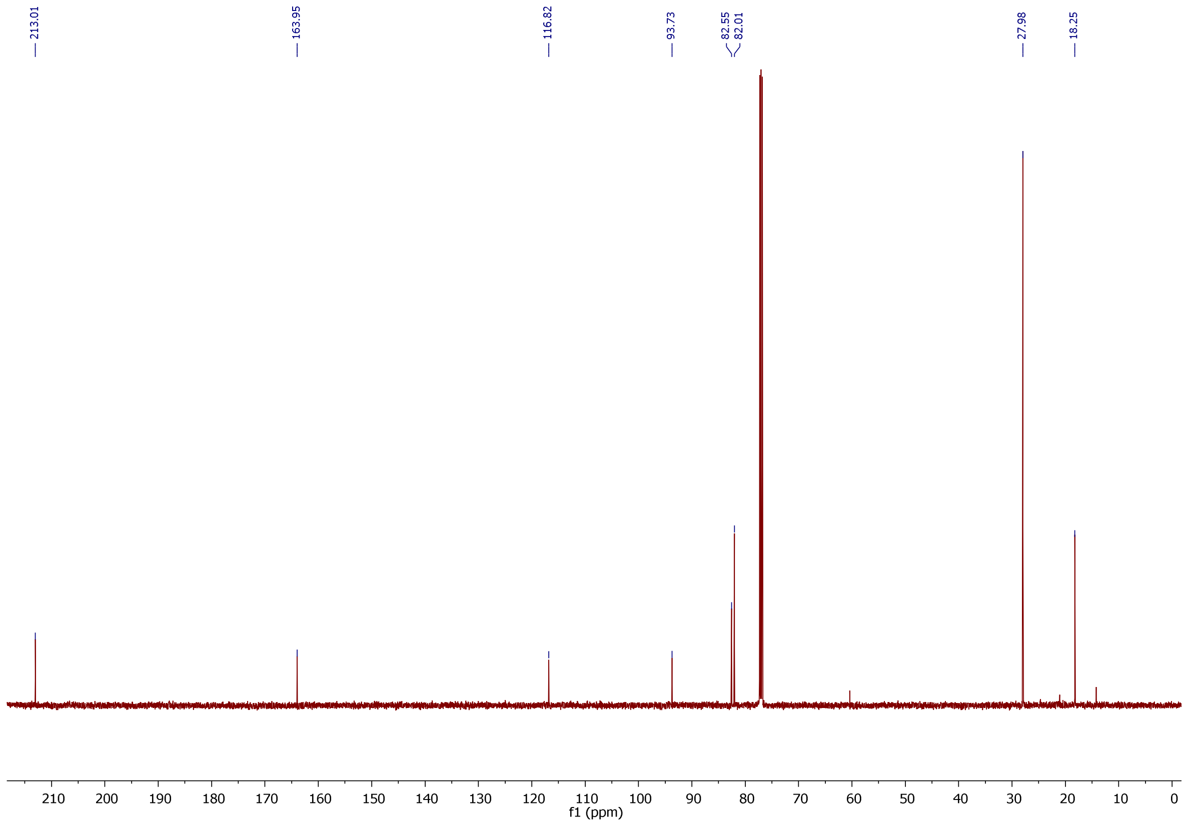
213.01
163.95
116.82
93.73
82.55
82.01
27.98
18.25
210 200 190 180 170 160 150 140 130 120 110 100 90 80 70 60 50 40 30 20 10 0
f1 (ppm)

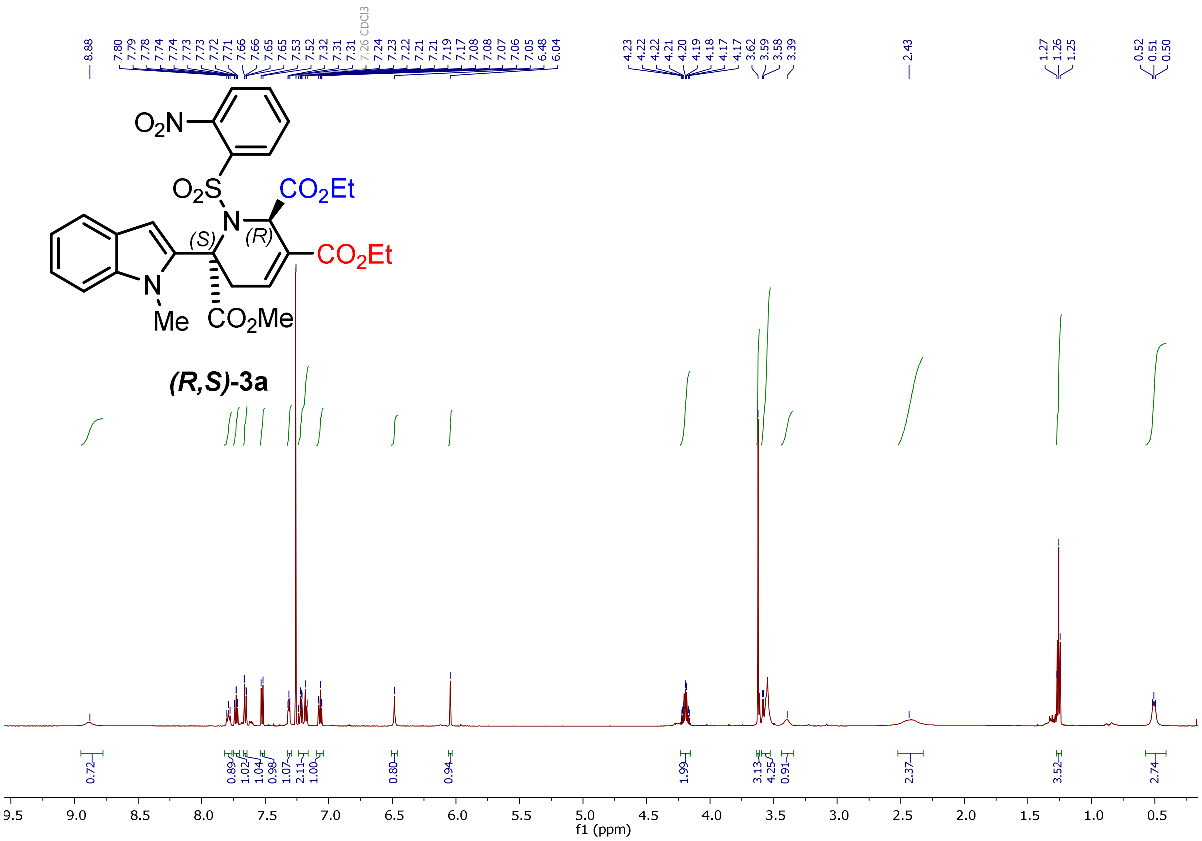
8.88
7.80 7.79 7.78 7.74 7.74 7.73 7.73 7.72 7.72 7.71 7.66 7.66 7.65 7.65 7.53 7.53 7.52 7.32 7.31 7.31 7.26 CDCl3 7.24 7.23 7.22 7.21 7.21 7.19 7.17 7.08 7.08 7.07 7.06 7.05 6.48 6.04
4.23 4.22 4.22 4.21 4.20 4.19 4.18 4.17 4.17 3.62 3.59 3.58 3.39
2.43
1.27 1.26 1.25
0.52 0.51 0.50
O_2N
O_2S
N
CO_2Et
(S)
(R)
CO_2Et
N
Me
CO_2Me
(R,S)-3a
0.72 0.89 1.02 1.04 0.98 1.07 2.11 1.00 0.80 0.94 1.99 3.13 4.25 0.91 2.37 3.52 2.74
9.5 9.0 8.5 8.0 7.5 7.0 6.5 6.0 5.5 5.0 4.5 4.0 3.5 3.0 2.5 2.0 1.5 1.0 0.5
f1 (ppm)

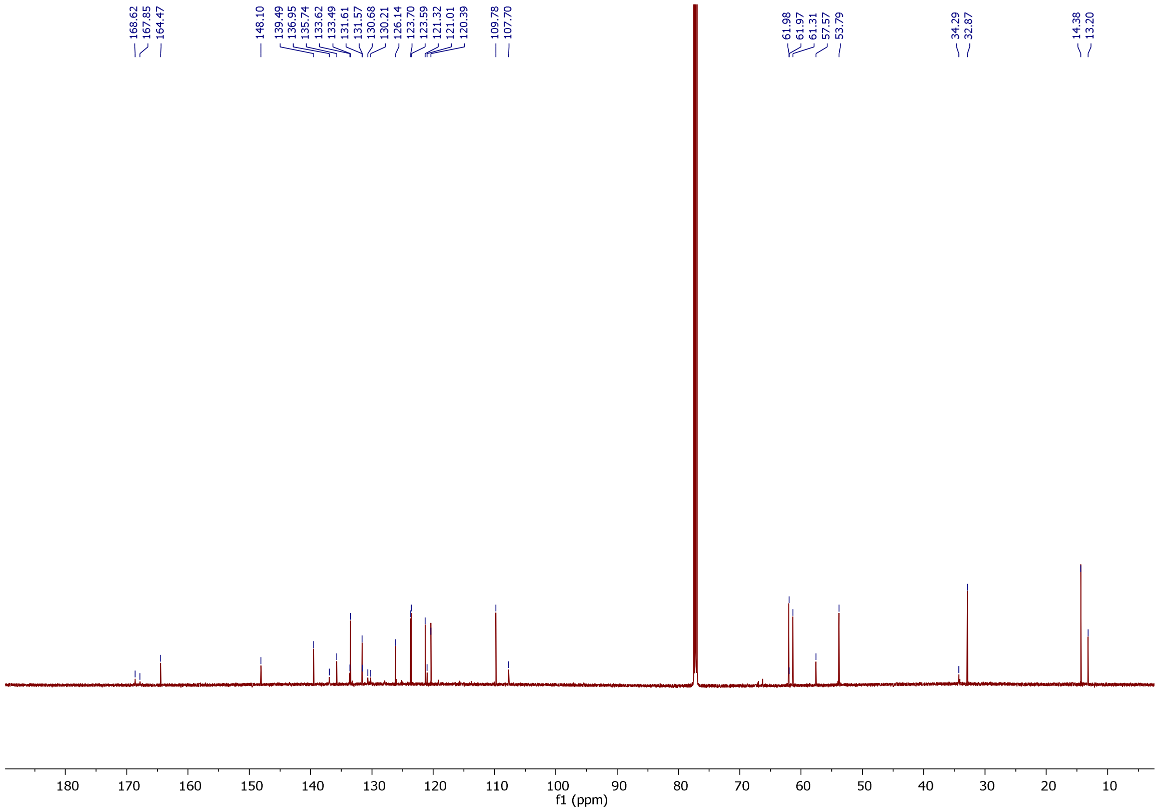
168.62 167.85 164.47
148.10 139.49 136.95 135.74 133.62 133.49 131.61 131.57 130.68 130.21 126.14 123.70 123.59 121.32 121.01 120.39 109.78 107.70
61.98 61.97 61.31 57.57 53.79
34.29 32.87
14.38 13.20
180 170 160 150 140 130 120 110 100 90 80 70 60 50 40 30 20 10
f1 (ppm)

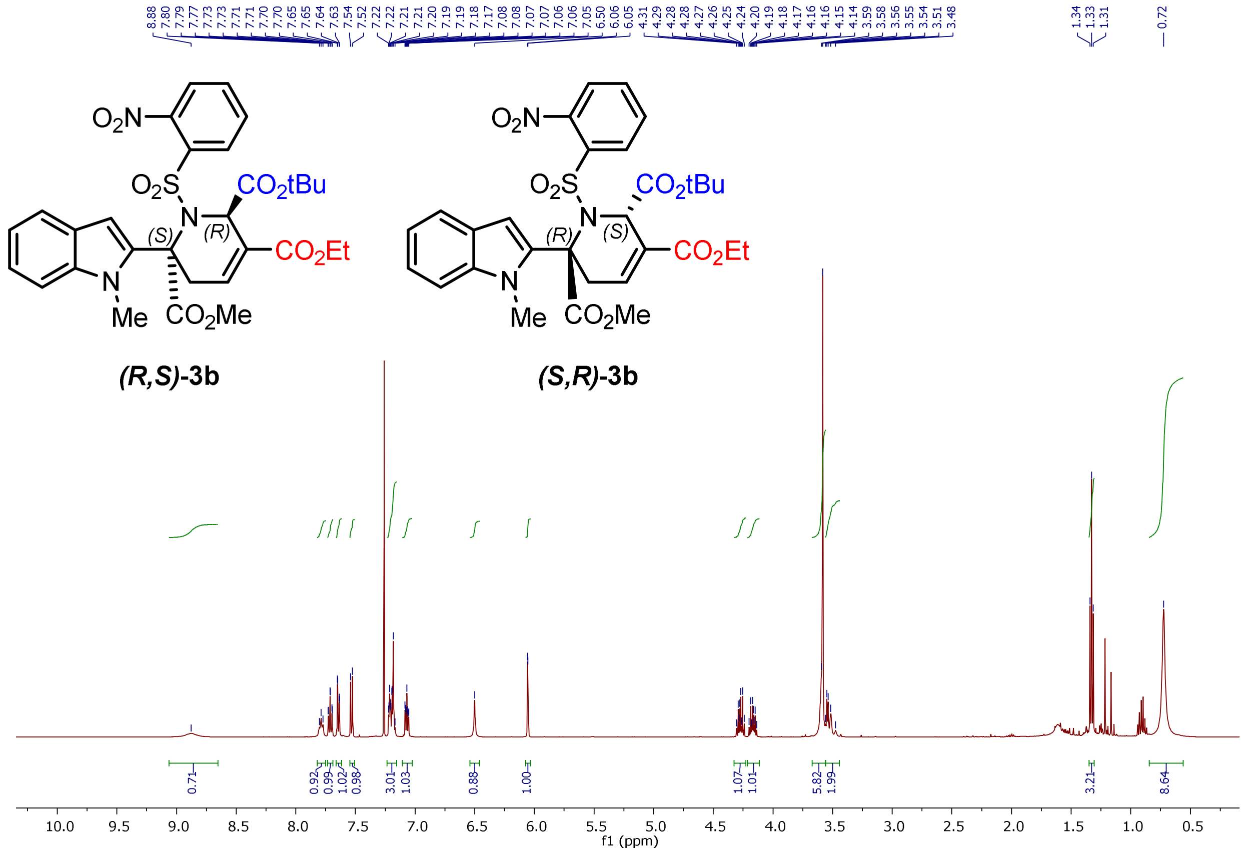
8.88 7.80 7.79 7.77 7.73 7.73 7.71 7.71 7.70 7.70 7.65 7.65 7.64 7.63 7.54 7.52 7.22 7.22 7.21 7.20 7.19 7.19 7.18 7.17 7.08 7.08 7.07 7.07 7.06 7.06 7.05 6.50 6.06 6.05 4.31 4.29 4.28 4.28 4.27 4.26 4.25 4.24 4.20 4.19 4.18 4.17 4.16 4.16 4.15 4.14 3.59 3.58 3.56 3.55 3.54 3.51 3.48
1.34 1.33 1.31
0.72
O2N
O2S
N
CO2tBu
(S)
(R)
CO2Et
N
Me
CO2Me
(R)
(S)
0.71 0.92 0.99 1.02 0.98 3.01 1.03 0.88 1.00 1.07 1.01 5.82 1.99 3.21 8.64
10.0 9.5 9.0 8.5 8.0 7.5 7.0 6.5 6.0 5.5 5.0 4.5 4.0 3.5 3.0 2.5 2.0 1.5 1.0 0.5
f1 (ppm)

(R,S)-3b **(S,R)-3b**

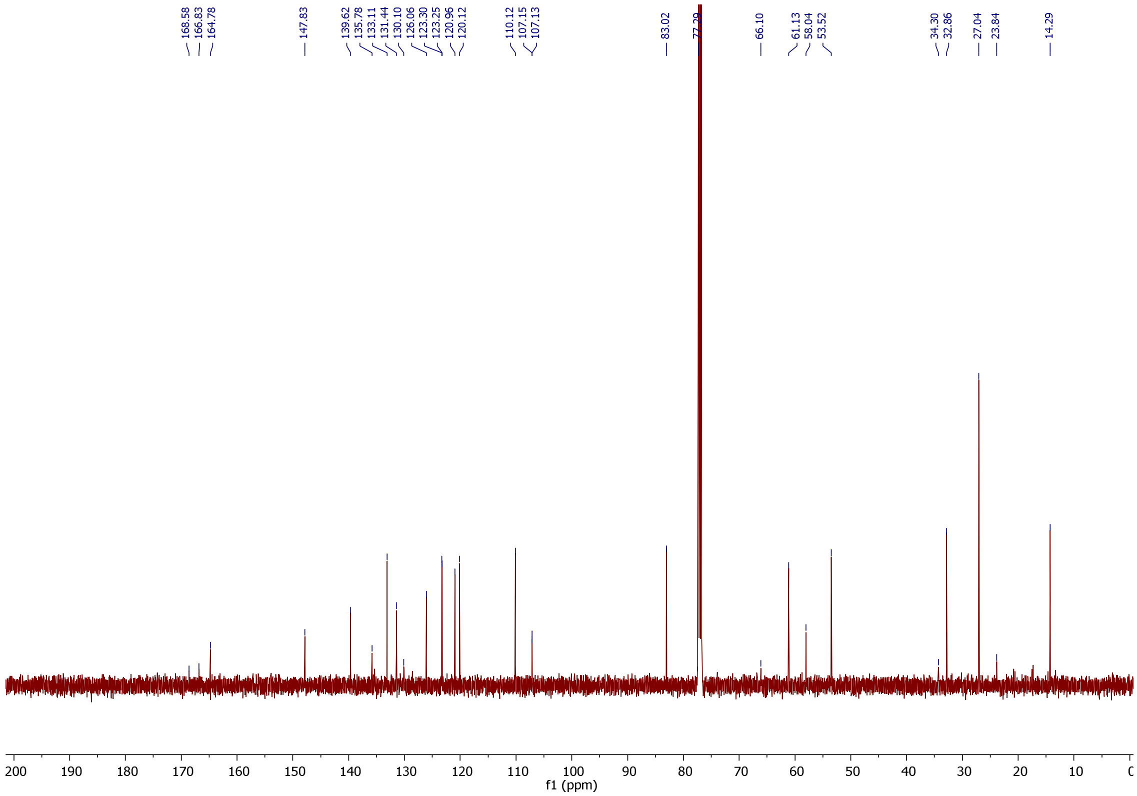
168.58 166.83 164.78
147.83
139.62 135.78 133.11 131.44 130.10 126.06 123.30 123.25 120.96 120.12
110.12 107.15 107.13
83.02
77.29
66.10
61.13 58.04 53.52
34.30 32.86
27.04 23.84
14.29
200 190 180 170 160 150 140 130 120 110 100 90 80 70 60 50 40 30 20 10 0
f1 (ppm)

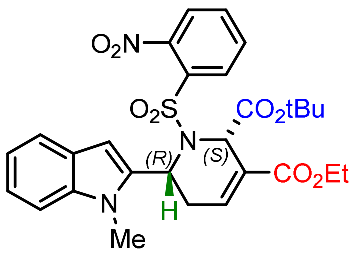

O_2N
O_2S
N
CO_2tBu
(R)
(S)
CO_2Et
N
H
Me

***(S,R)*-3c**

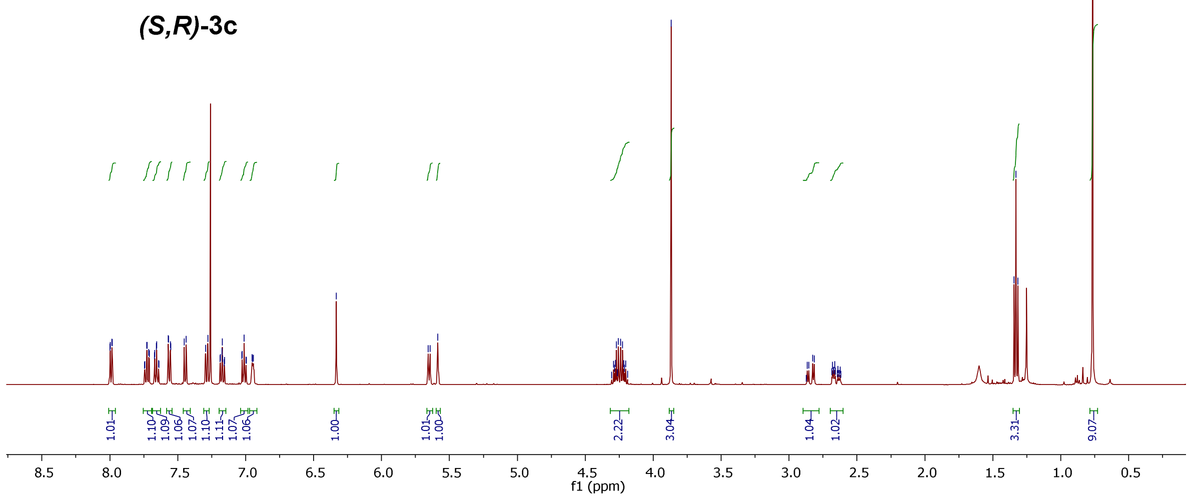

f1 (ppm)

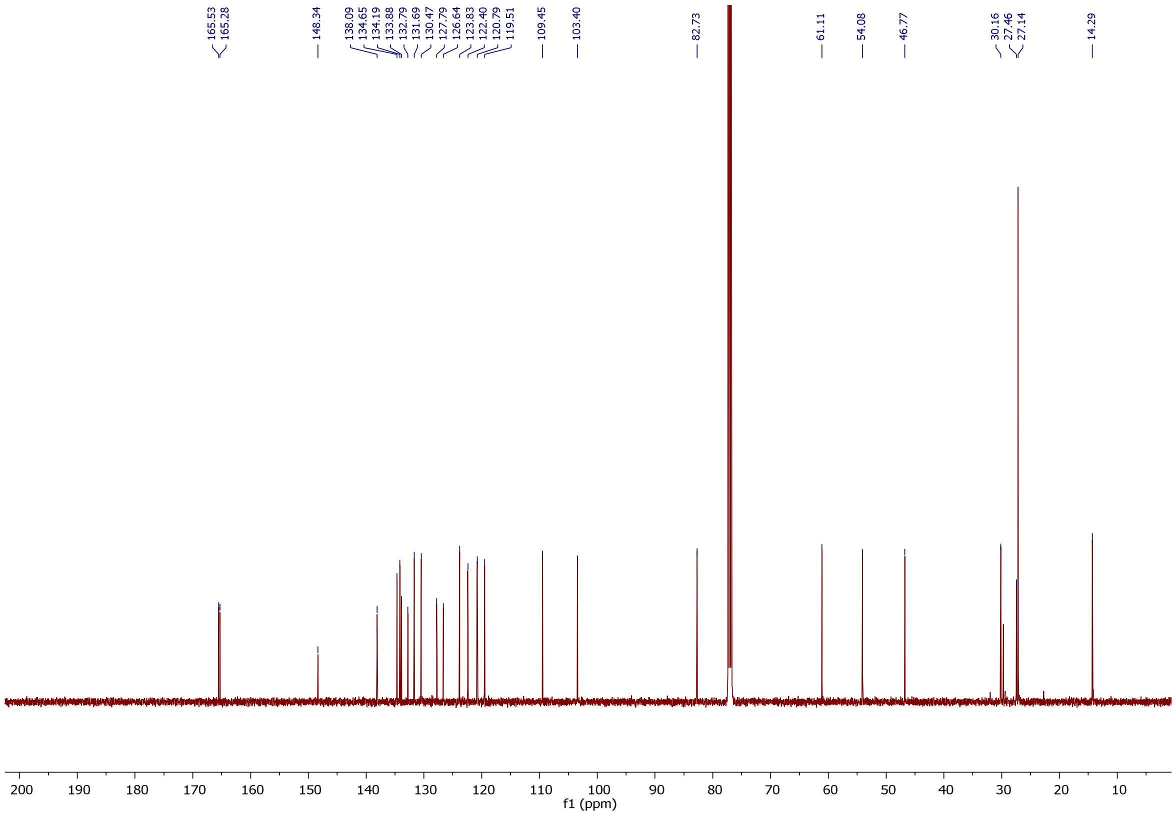

165.53
165.28
148.34
138.09
134.65
134.19
133.88
132.79
131.69
130.47
127.79
126.64
123.83
122.40
120.79
119.51
109.45
103.40
82.73
61.11
54.08
46.77
30.16
27.46
27.14
14.29
f1 (ppm)

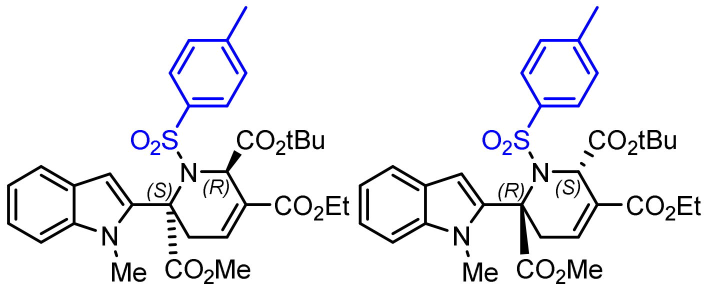
O2S
N
CO2tBu
(S)
(R)
CO2Et
N
Me
CO2Me
O2S
N
CO2tBu
(R)
(S)
CO2Et
N
Me
CO2Me

***(R,S)*-3d** ***(S,R)*-3d**

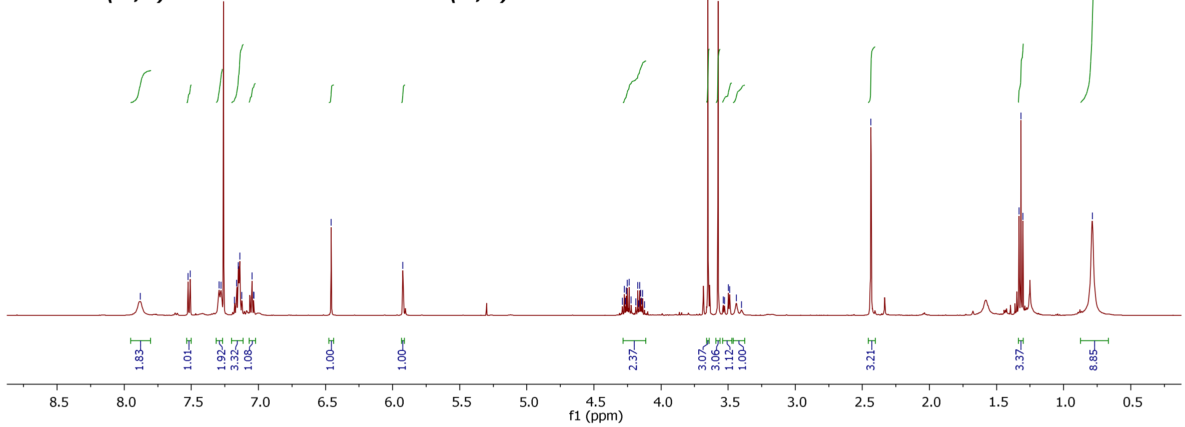
7.88
7.52
7.51
7.29
7.28
7.18
7.16
7.15
7.14
7.12
7.05
7.03
6.46
5.92
4.29
4.27
4.27
4.26
4.25
4.24
4.22
4.19
4.17
4.17
4.16
4.15
4.14
4.14
4.12
3.65
3.58
3.54
3.53
3.50
3.49
3.44
3.40
2.44
1.33
1.32
1.30
0.79
1.83
1.01
1.92
3.32
1.08
1.00
1.00
2.37
3.07
3.06
1.12
1.00
3.21
3.37
8.85
8.5
8.0
7.5
7.0
6.5
6.0
5.5
5.0
4.5
4.0
3.5
3.0
2.5
2.0
1.5
1.0
0.5
f1 (ppm)

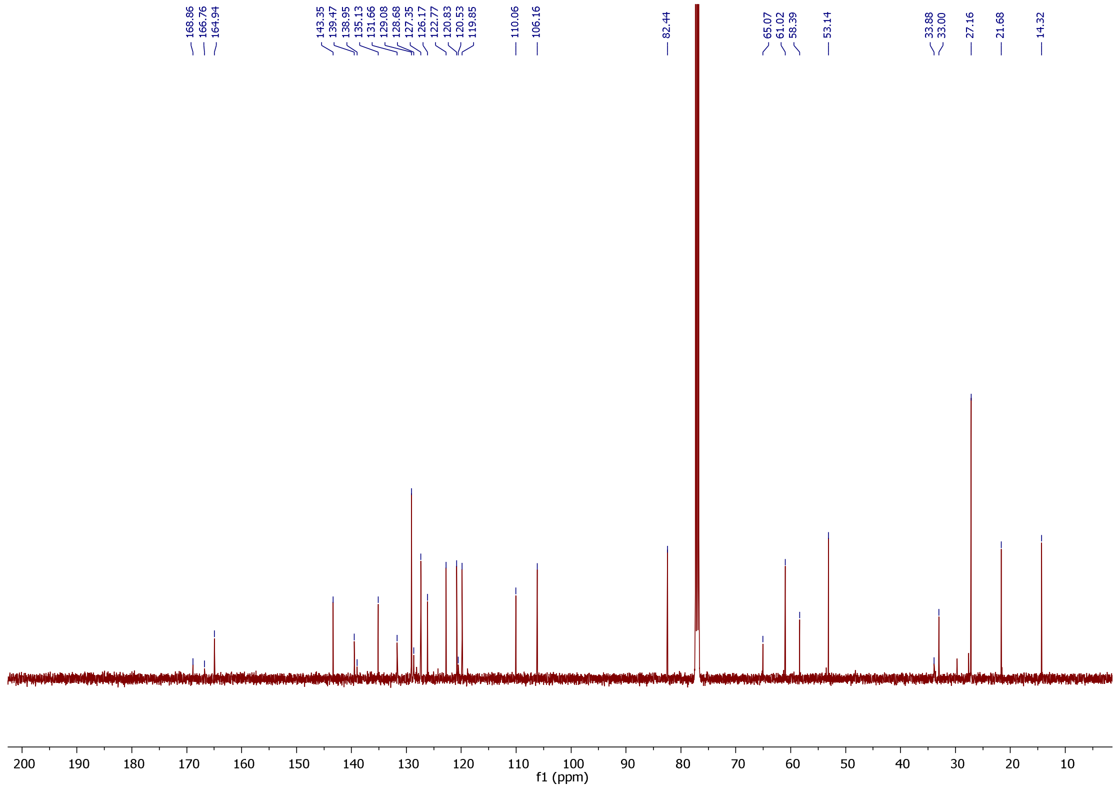
168.86
166.76
164.94
143.35
139.47
138.95
135.13
131.66
129.08
128.68
127.35
126.17
122.77
120.83
120.53
119.85
110.06
106.16
82.44
65.07
61.02
58.39
53.14
33.88
33.00
27.16
21.68
14.32
200
190
180
170
160
150
140
130
120
110
100
90
80
70
60
50
40
30
20
10
f1 (ppm)

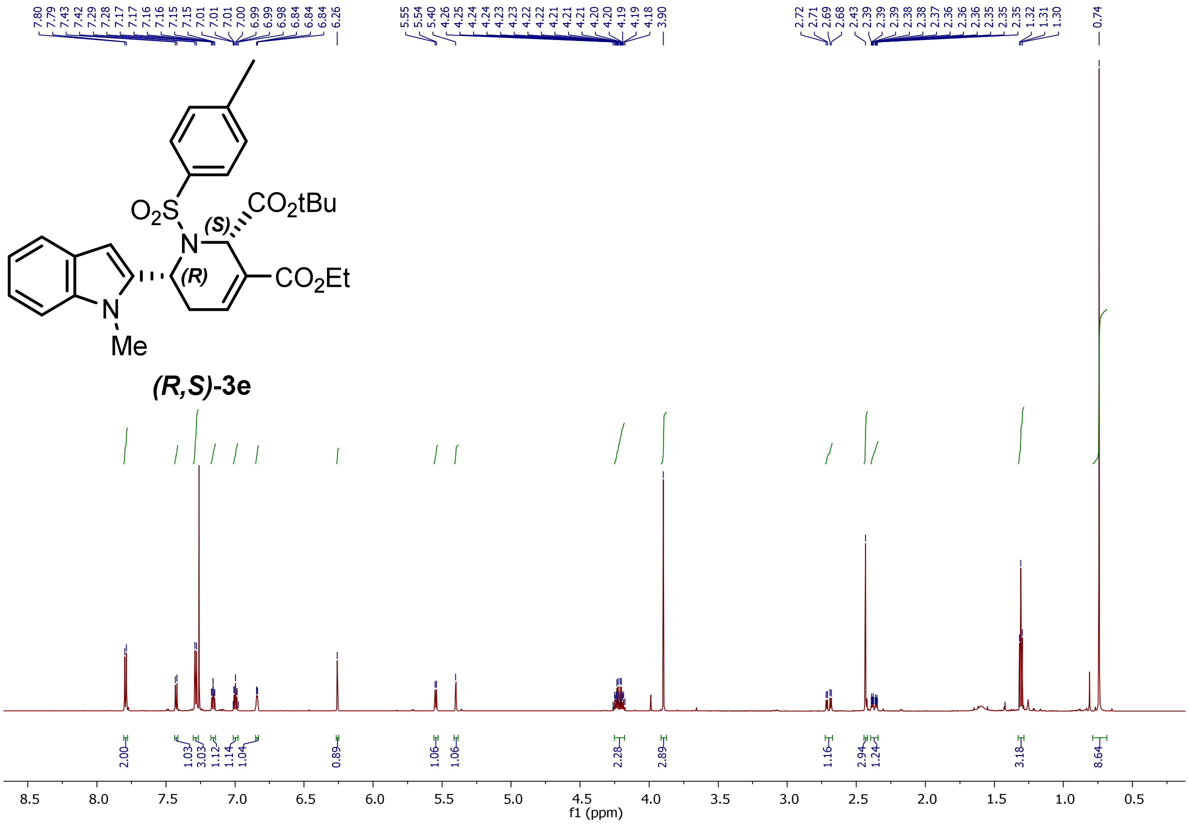

O_2S
N
(S)
CO_2tBu
(R)
CO_2Et
N
Me
(R,S)-3e
f1 (ppm)

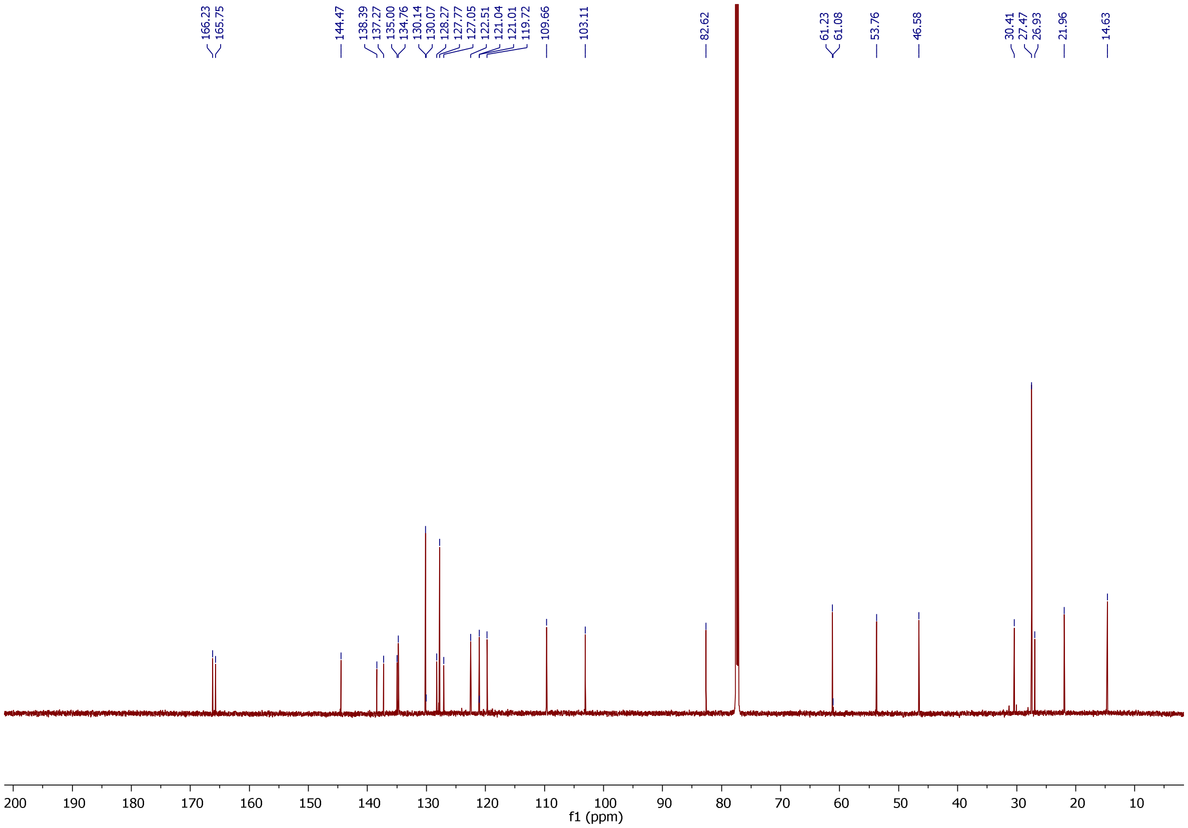

f1 (ppm)

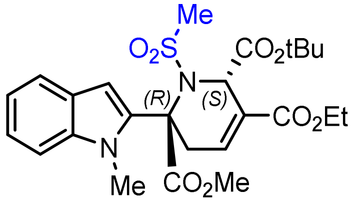

***(S,R)*-3f**

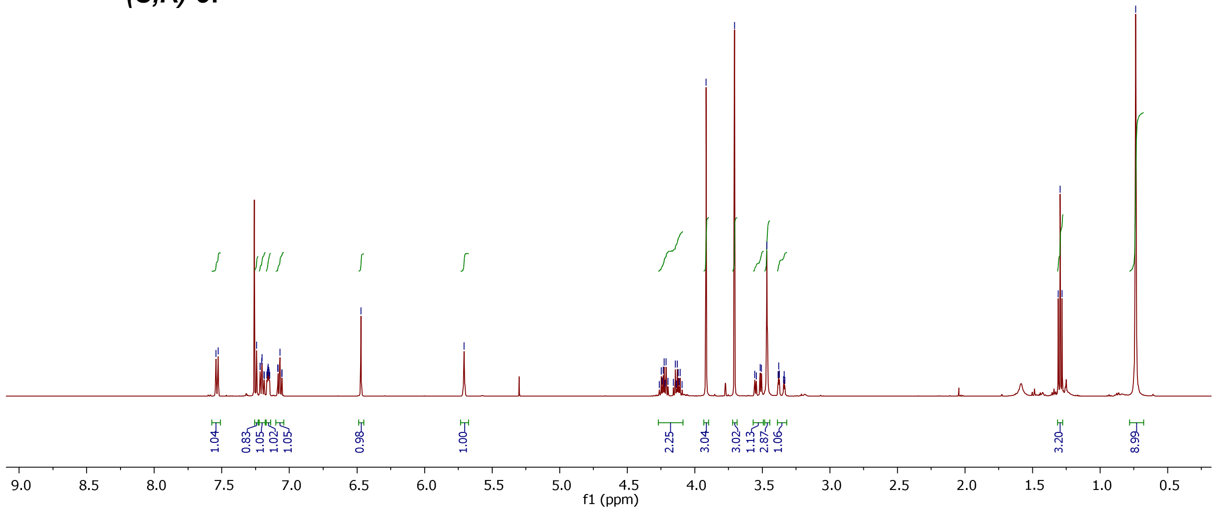

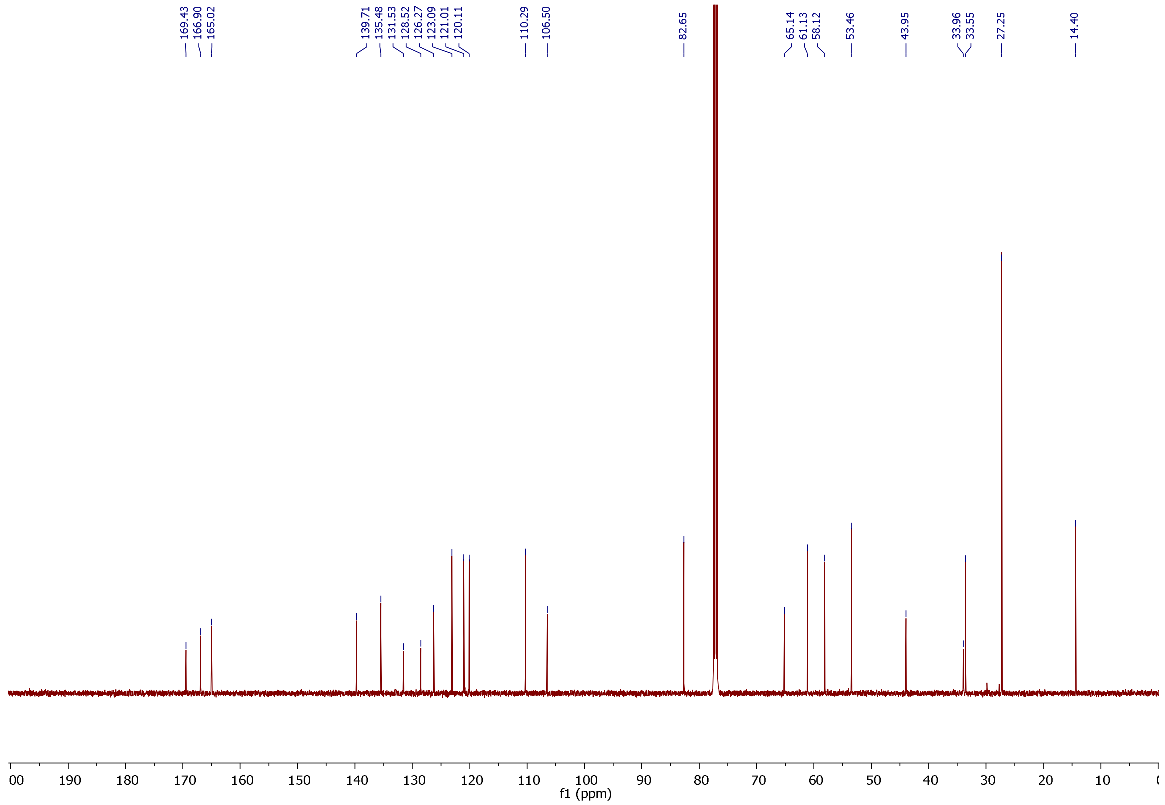

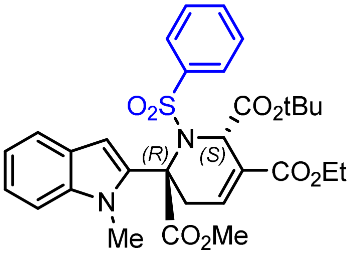

O_2S
N
CO_2tBu
(R)
(S)
CO_2Et
N
Me
CO_2Me

***(S,R)*-3g**

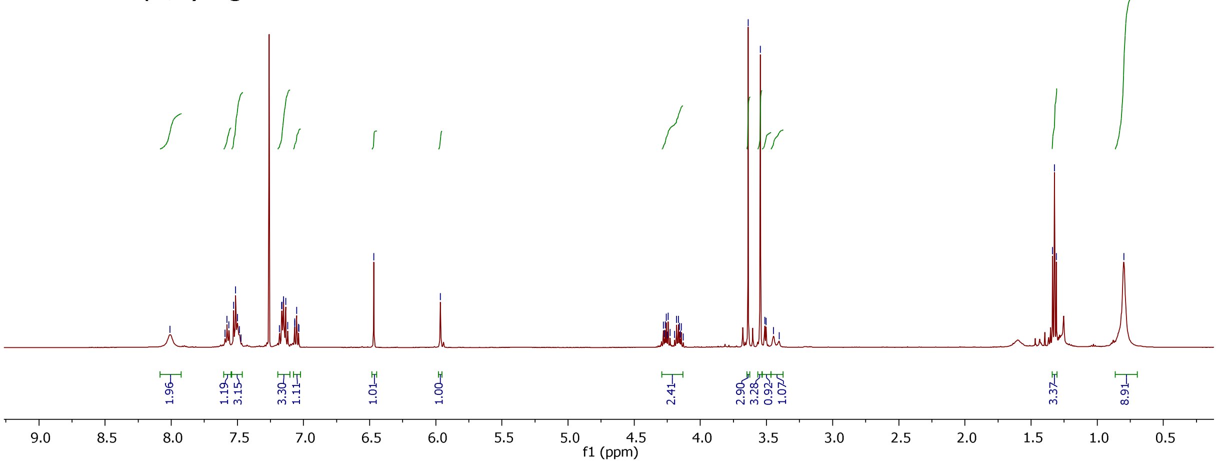

8.01 7.59 7.58 7.56 7.53 7.51 7.50 7.49 7.47 7.18 7.17 7.16 7.15 7.13 7.12 7.07 7.05 7.04 7.04 6.47 5.97
4.28 4.27 4.26 4.25 4.24 4.23 4.19 4.18 4.17 4.16 4.15 4.14 4.13 3.64 3.55 3.51 3.50 3.45 3.40
1.34 1.32 1.31
0.80
1.96 1.19 3.15 3.30 1.11 1.01 1.00 2.41 2.90 3.28 0.92 1.07 3.37 8.91
9.0 8.5 8.0 7.5 7.0 6.5 6.0 5.5 5.0 4.5 4.0 3.5 3.0 2.5 2.0 1.5 1.0 0.5
f1 (ppm)

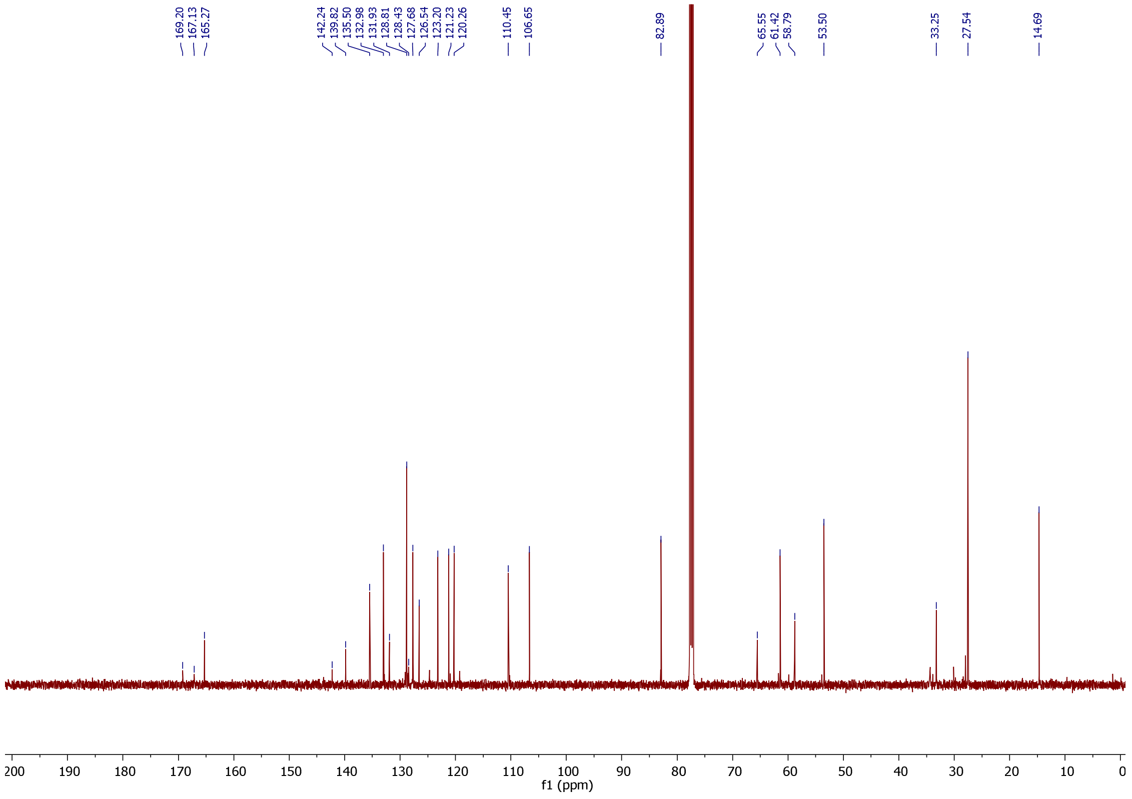

169.20 167.13 165.27
142.24 139.82 135.50 132.98 131.93 128.81 128.43 127.68 126.54 123.20 121.23 120.26
110.45 106.65
82.89
65.55 61.42 58.79
53.50
33.25
27.54
14.69
200 190 180 170 160 150 140 130 120 110 100 90 80 70 60 50 40 30 20 10 0
f1 (ppm)

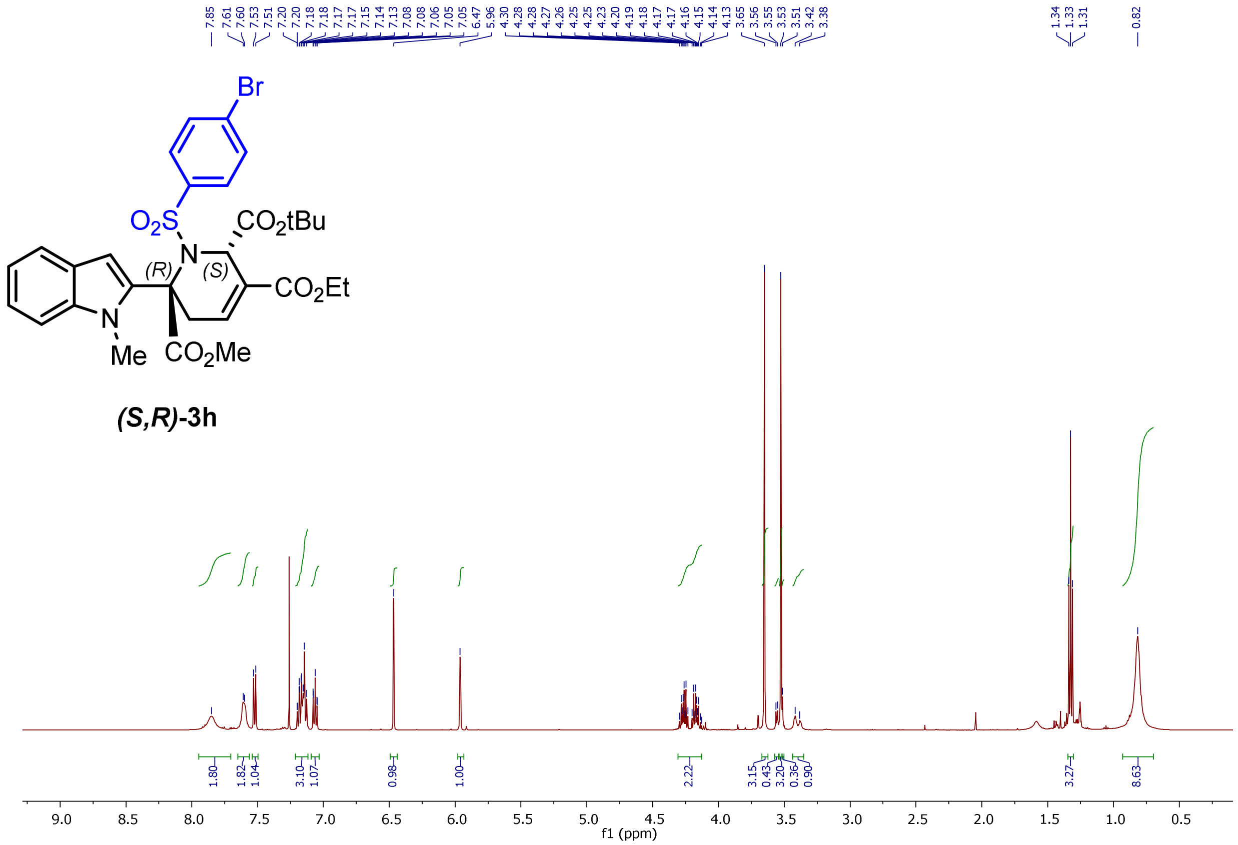

(S,R)-3h

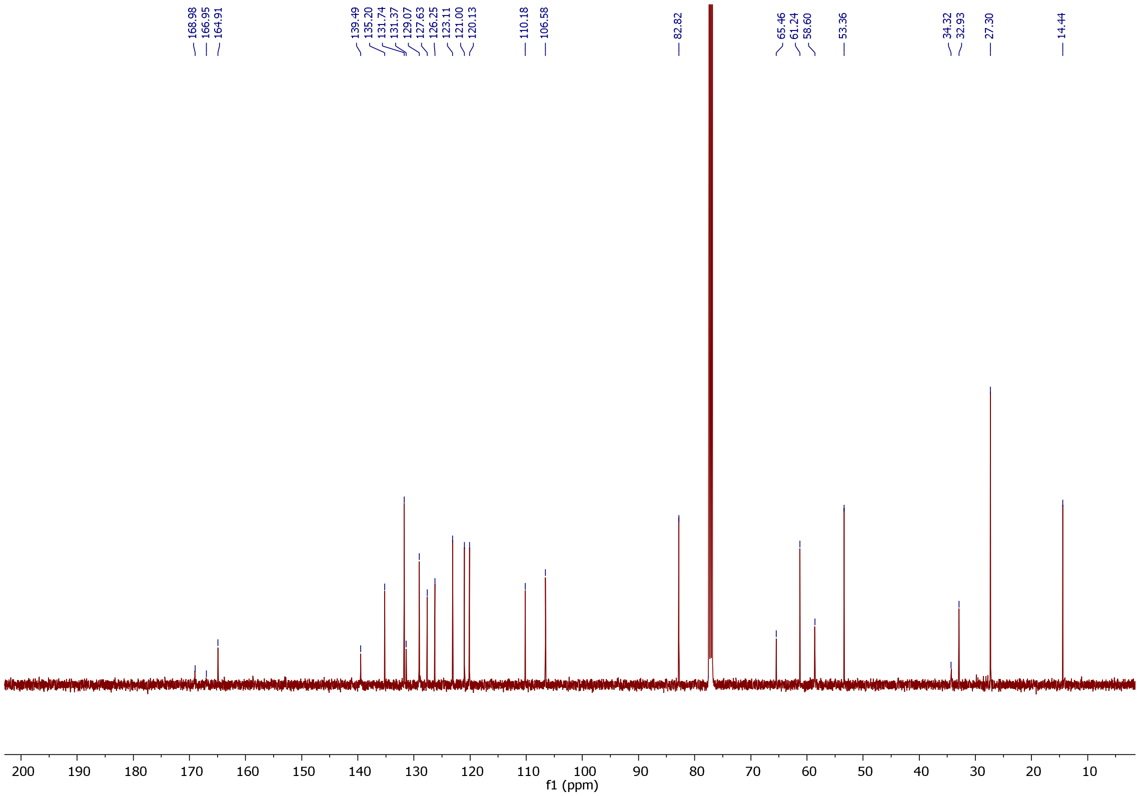

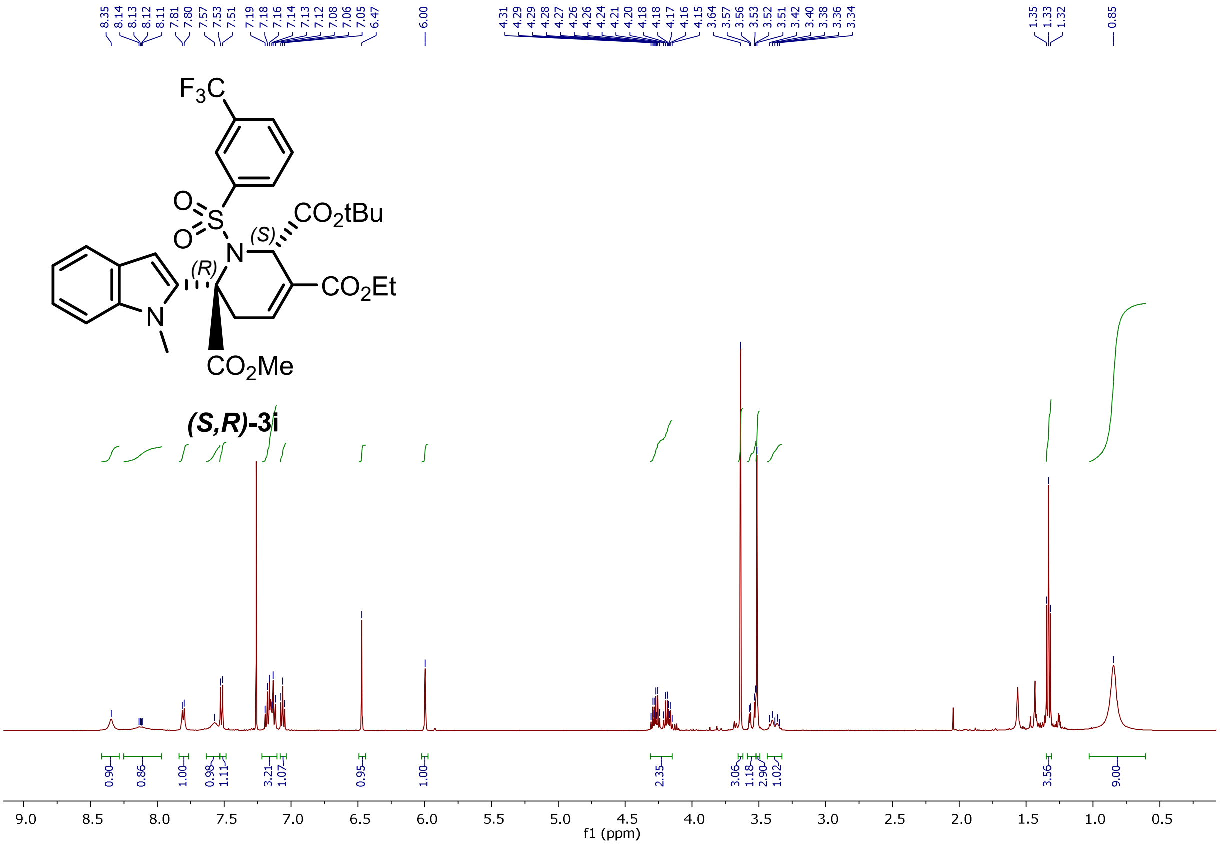

8.35
8.14
8.13
8.12
8.11
7.81
7.80
7.57
7.53
7.51
7.19
7.18
7.16
7.14
7.13
7.12
7.08
7.06
7.05
6.47
6.00
4.31
4.29
4.29
4.28
4.27
4.26
4.26
4.24
4.21
4.20
4.18
4.18
4.17
4.16
4.15
3.64
3.57
3.56
3.53
3.52
3.51
3.42
3.40
3.38
3.36
3.34
1.35
1.33
1.32
0.85
F3C
CO2tBu
CO2Et
CO2Me
(S)
(R)
(S,R)-3i
0.90
0.86
1.00
0.98
1.11
3.21
1.07
0.95
1.00
2.35
3.06
1.18
2.90
1.02
3.56
9.00
9.0 8.5 8.0 7.5 7.0 6.5 6.0 5.5 5.0 4.5 4.0 3.5 3.0 2.5 2.0 1.5 1.0 0.5
f1 (ppm)

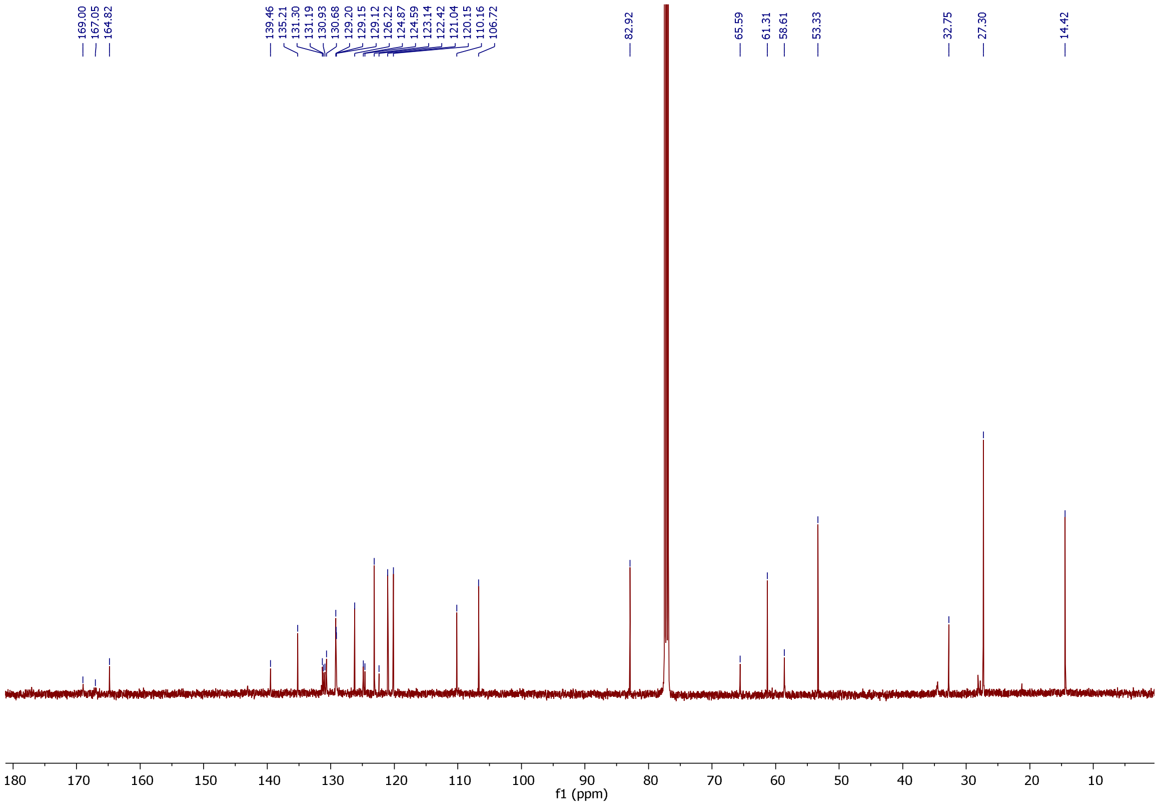

169.00
167.05
164.82
139.46
135.21
131.30
131.19
130.93
130.68
129.20
129.15
129.12
126.22
124.87
124.59
123.14
122.42
121.04
120.15
110.16
106.72
82.92
65.59
61.31
58.61
53.33
32.75
27.30
14.42
180 170 160 150 140 130 120 110 100 90 80 70 60 50 40 30 20 10
f1 (ppm)

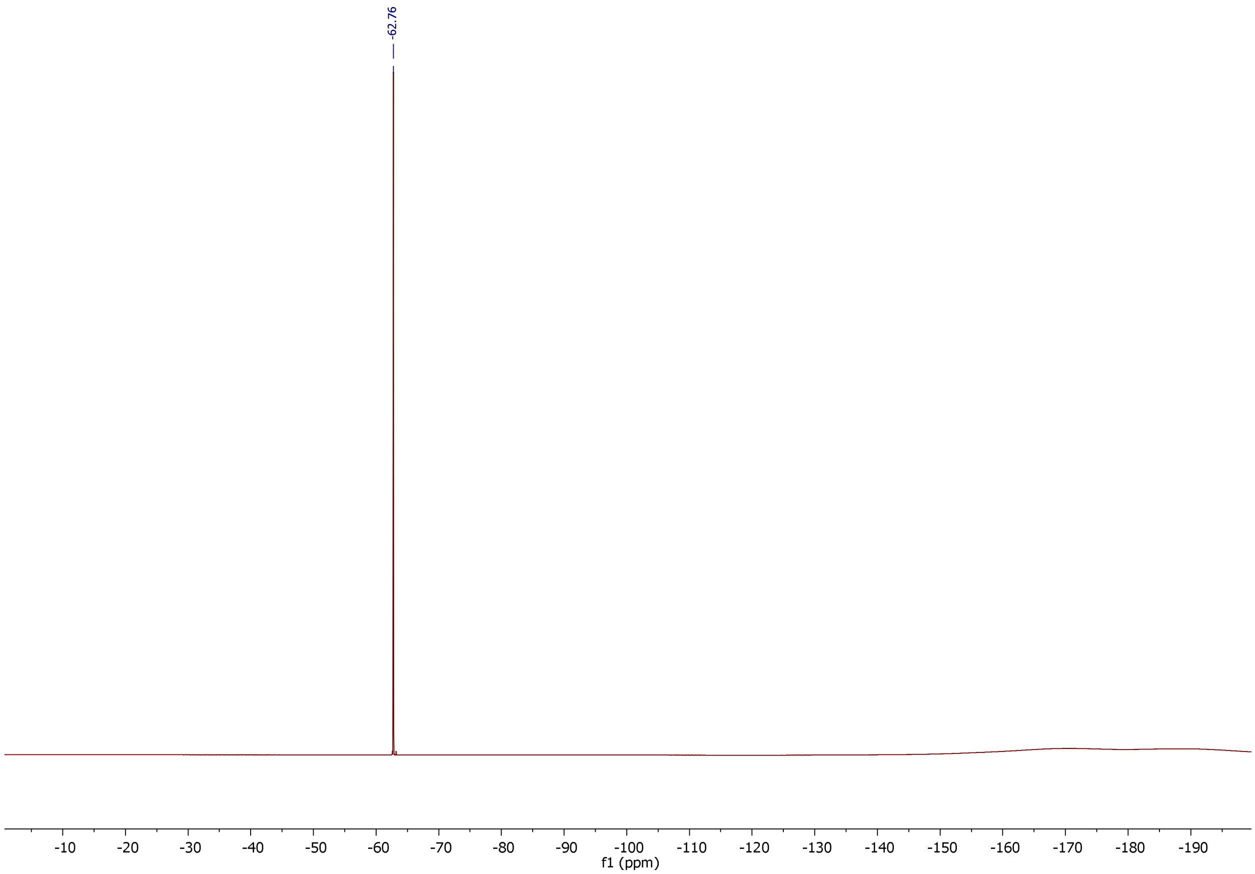
-62.76
-10
-20
-30
-40
-50
-60
-70
-80
-90
-100
-110
-120
-130
-140
-150
-160
-170
-180
-190
f1 (ppm)

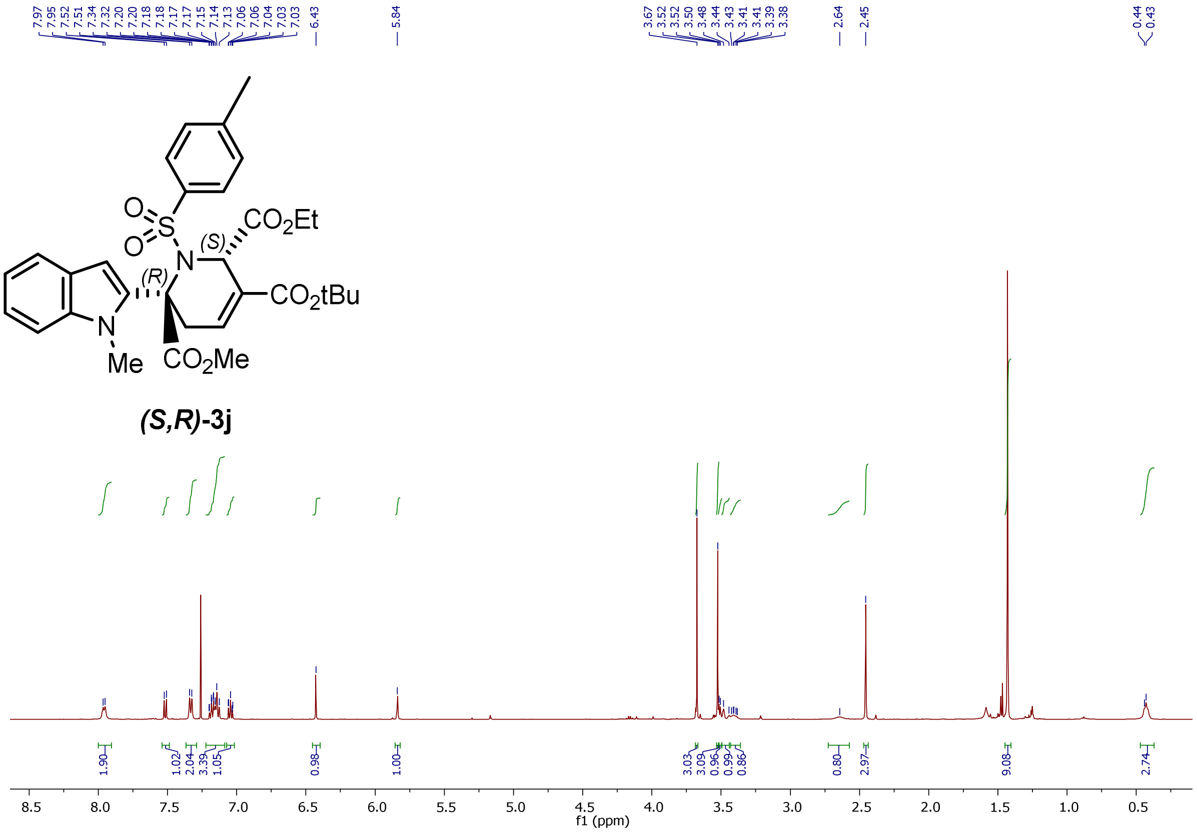
7.97 7.95 7.52 7.51 7.34 7.32 7.20 7.18 7.17 7.17 7.15 7.14 7.13 7.06 7.06 7.04 7.03 7.03 6.43 5.84
3.67 3.52 3.52 3.50 3.48 3.44 3.43 3.41 3.39 3.38 2.64 2.45 0.44 0.43
CO2Et
CO2tBu
CO2Me
(S)
(R)
N
S
O
O
N
Me
(S,R)-3j
1.90 1.02 2.04 3.39 1.05 0.98 1.00 3.03 3.09 0.96 0.99 0.86 0.80 2.97 9.08 2.74
8.5 8.0 7.5 7.0 6.5 6.0 5.5 5.0 4.5 4.0 3.5 3.0 2.5 2.0 1.5 1.0 0.5
f1 (ppm)

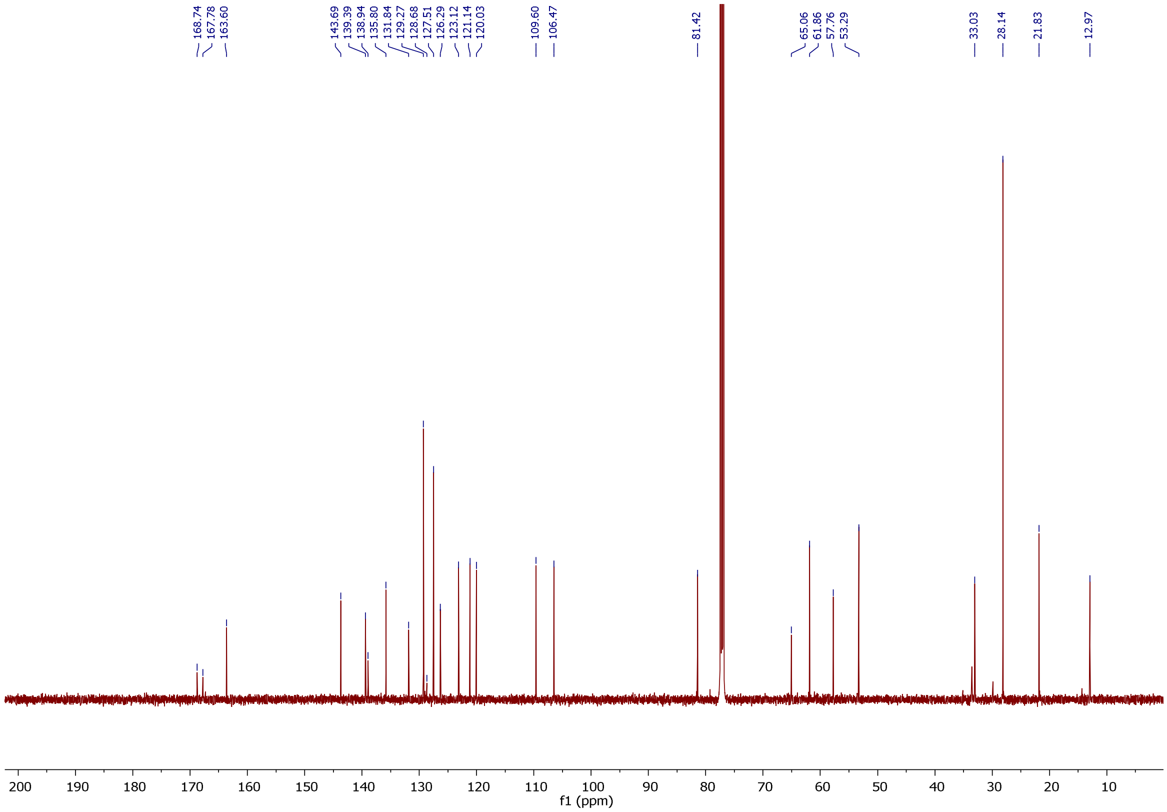
168.74 167.78 163.60 143.69 139.39 138.94 135.80 131.84 129.27 128.68 127.51 126.29 123.12 121.14 120.03 109.60 106.47 81.42 65.06 61.86 57.76 53.29 33.03 28.14 21.83 12.97
200 190 180 170 160 150 140 130 120 110 100 90 80 70 60 50 40 30 20 10
f1 (ppm)

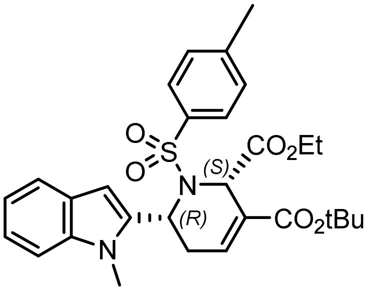

(S,R)-3k

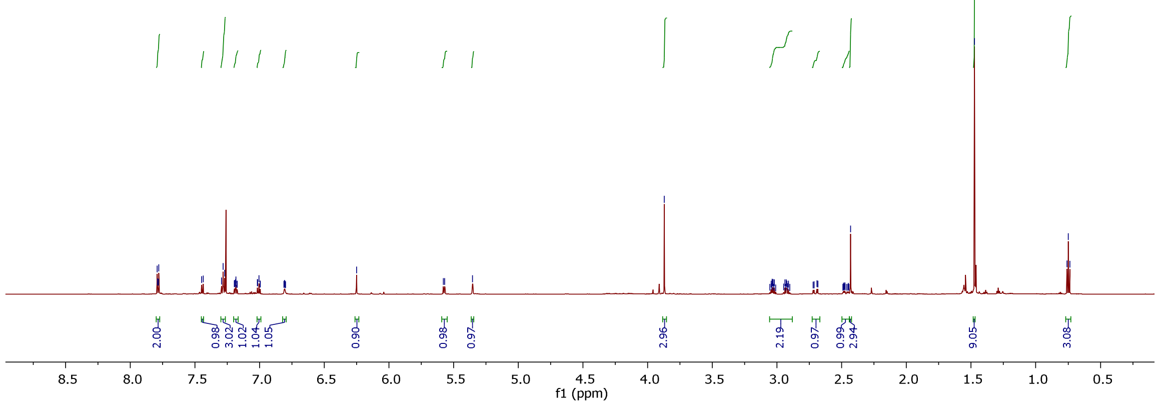

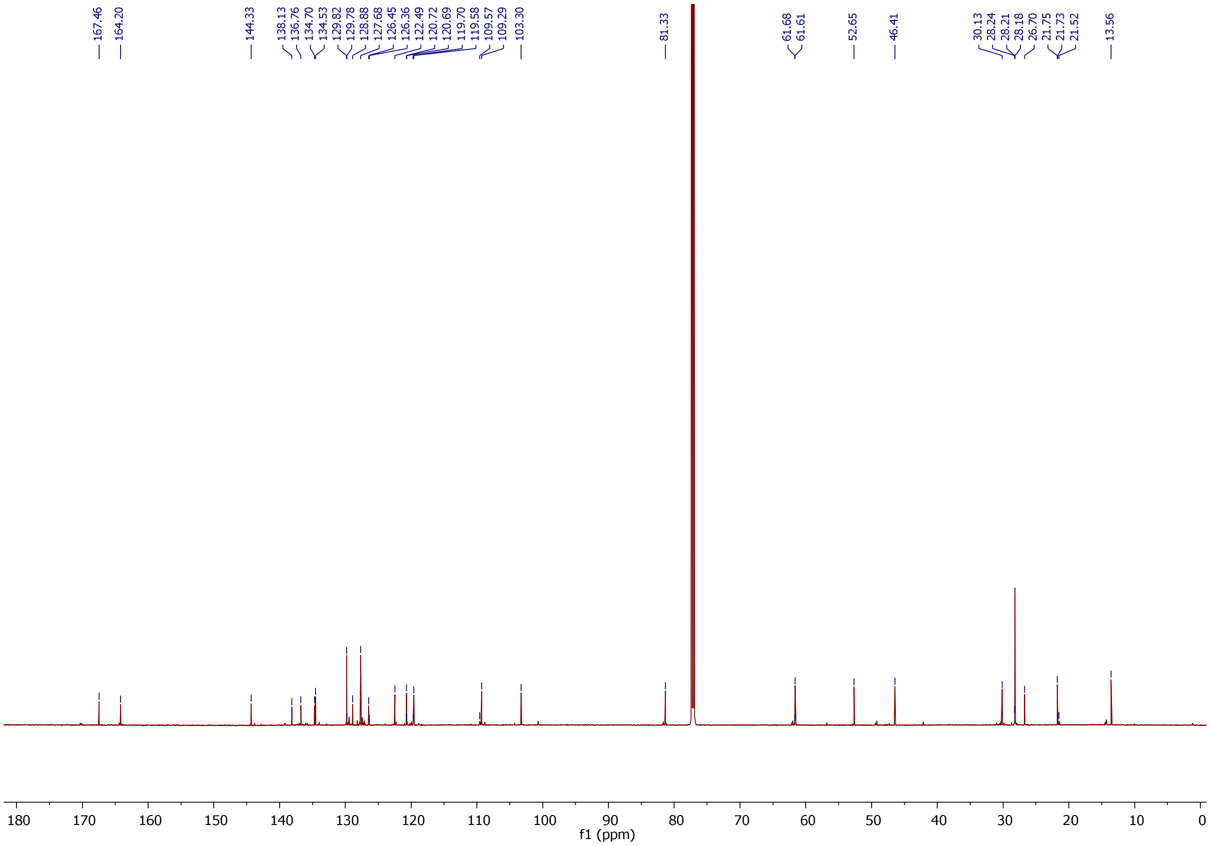
167.46
164.20
144.33
138.13
136.76
134.70
134.53
129.82
129.78
128.88
127.68
126.45
126.36
122.49
120.72
120.69
119.70
119.58
109.57
109.29
103.30
81.33
61.68
61.61
52.65
46.41
30.13
28.24
28.21
28.18
26.70
21.75
21.73
21.52
13.56
f1 (ppm)

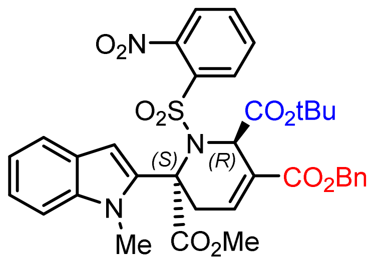
O2N
O2S
CO2tBu
CO2Bn
(S)
(R)
N
Me
CO2Me

***(R,S)*-3l**

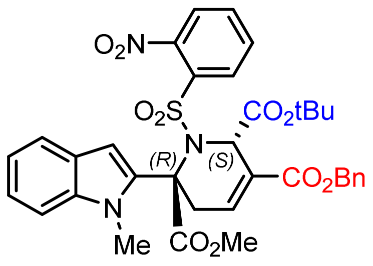
O2N
O2S
CO2tBu
CO2Bn
(R)
(S)
N
Me
CO2Me

***(S,R)*-3l**

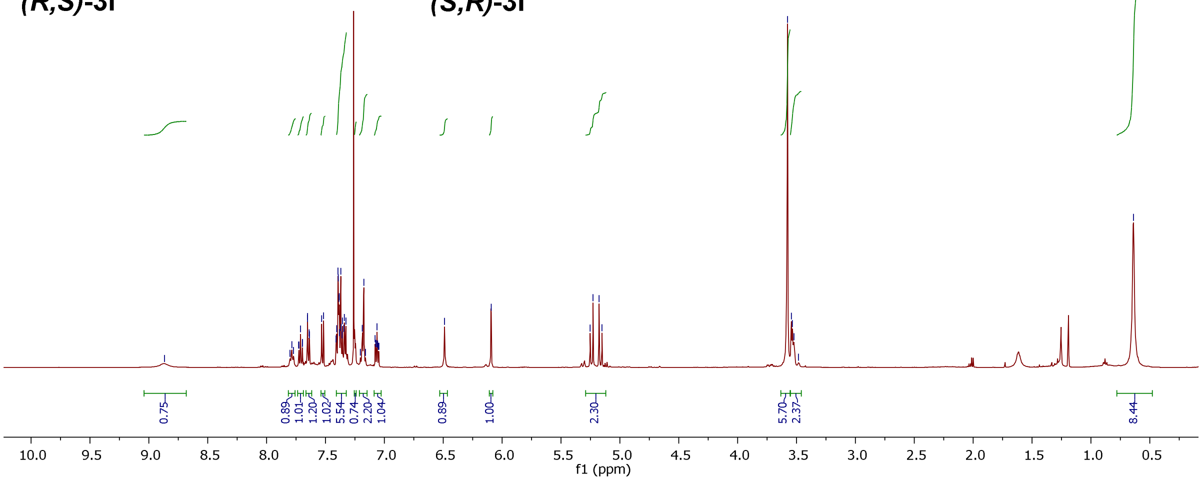
8.87
7.80 7.79 7.77 7.73 7.73 7.71 7.70 7.69 7.65 7.65 7.64 7.64 7.53 7.52 7.41 7.41 7.39 7.39 7.39 7.38 7.38 7.37 7.37 7.36 7.35 7.34 7.34 7.33 7.20 7.19 7.17 7.16 7.08 7.07 7.06 7.06 7.05 7.05 6.49 6.09 5.25 5.23 5.18 5.15 3.58 3.54 3.53 3.52 3.48
0.64
0.75 0.89 1.01 1.20 1.02 5.54 0.74 2.20 1.04 0.89 1.00 2.30 5.70 2.37 8.44
10.0 9.5 9.0 8.5 8.0 7.5 7.0 6.5 6.0 5.5 5.0 4.5 4.0 3.5 3.0 2.5 2.0 1.5 1.0 0.5
f1 (ppm)

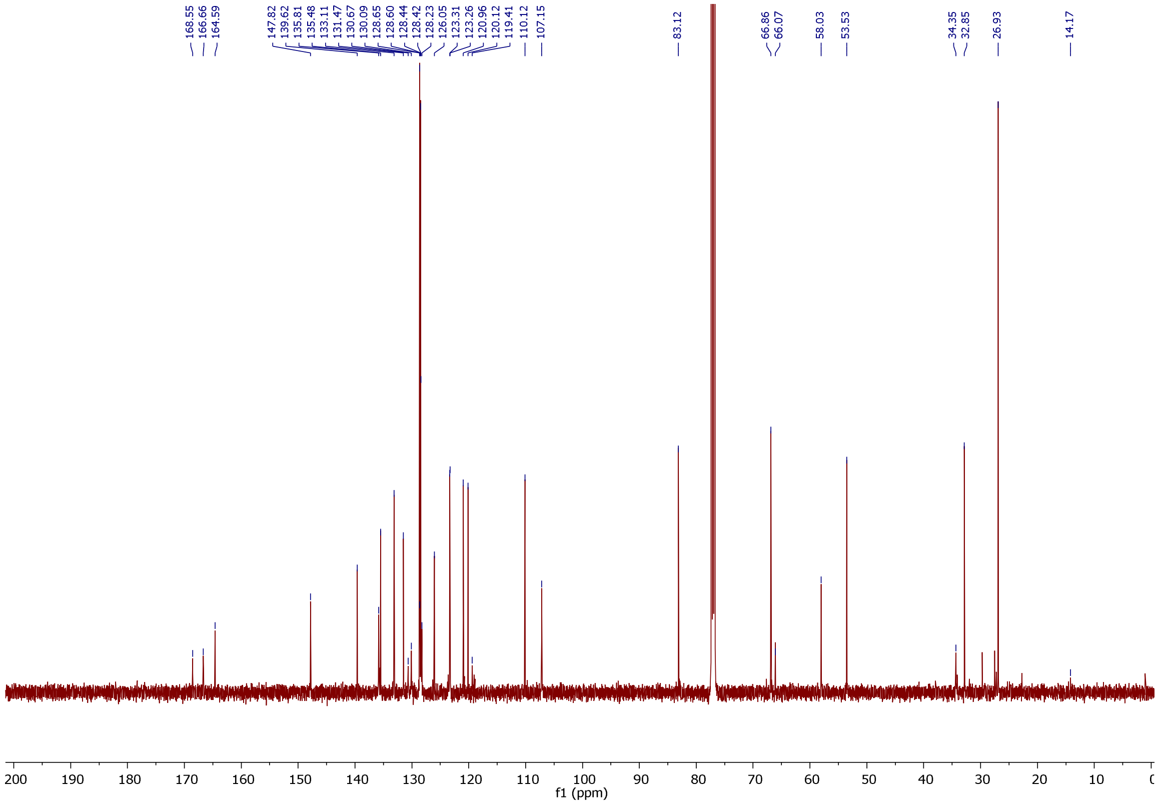
168.55 166.66 164.59
147.82 139.62 135.81 135.48 133.11 131.47 130.67 130.09 128.65 128.60 128.44 128.42 128.23 126.05 123.31 123.26 120.96 120.12 119.41 110.12 107.15
83.12
66.86 66.07
58.03 53.53
34.35 32.85 26.93
14.17
200 190 180 170 160 150 140 130 120 110 100 90 80 70 60 50 40 30 20 10
f1 (ppm)

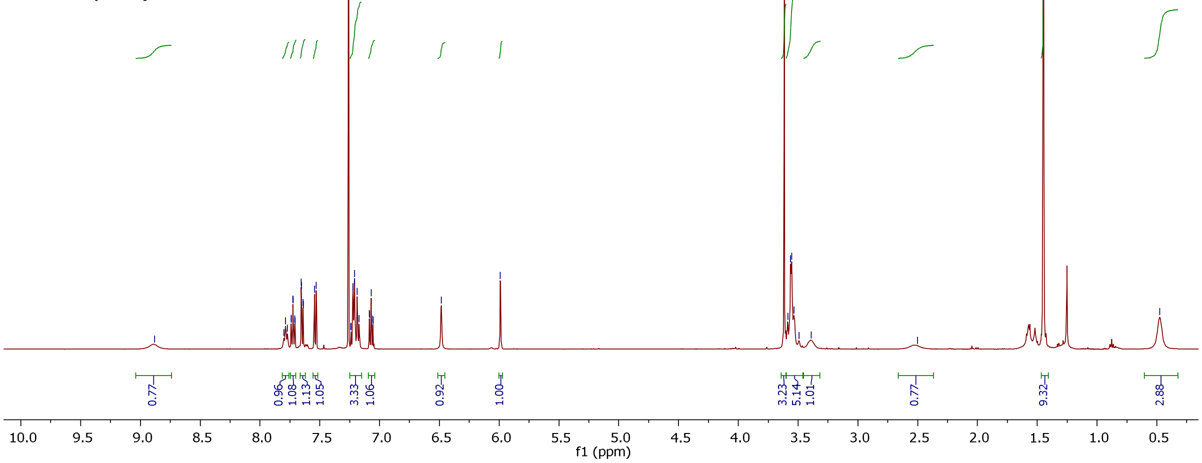
8.88 7.80 7.79 7.77 7.74 7.74 7.72 7.72 7.71 7.71 7.66 7.65 7.64 7.64 7.54 7.53 7.24 7.24 7.22 7.22 7.21 7.21 7.19 7.17 7.09 7.08 7.07 7.06 7.05 6.48 5.99
3.62 3.59 3.56 3.55 3.53 3.49 3.39
2.50
1.45
0.48
0.77 0.96 1.08 1.13 1.05 3.33 1.06 0.92 1.00 3.23 5.14 1.01 0.77 9.32 2.88
f1 (ppm)

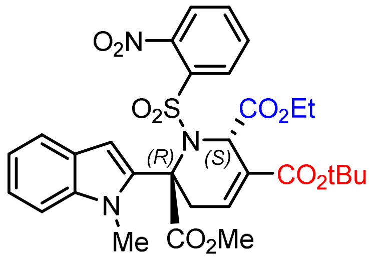
O2N
O2S
N
CO2Et
(R)
(S)
CO2tBu
N
Me
CO2Me

(S,R)-**3m**

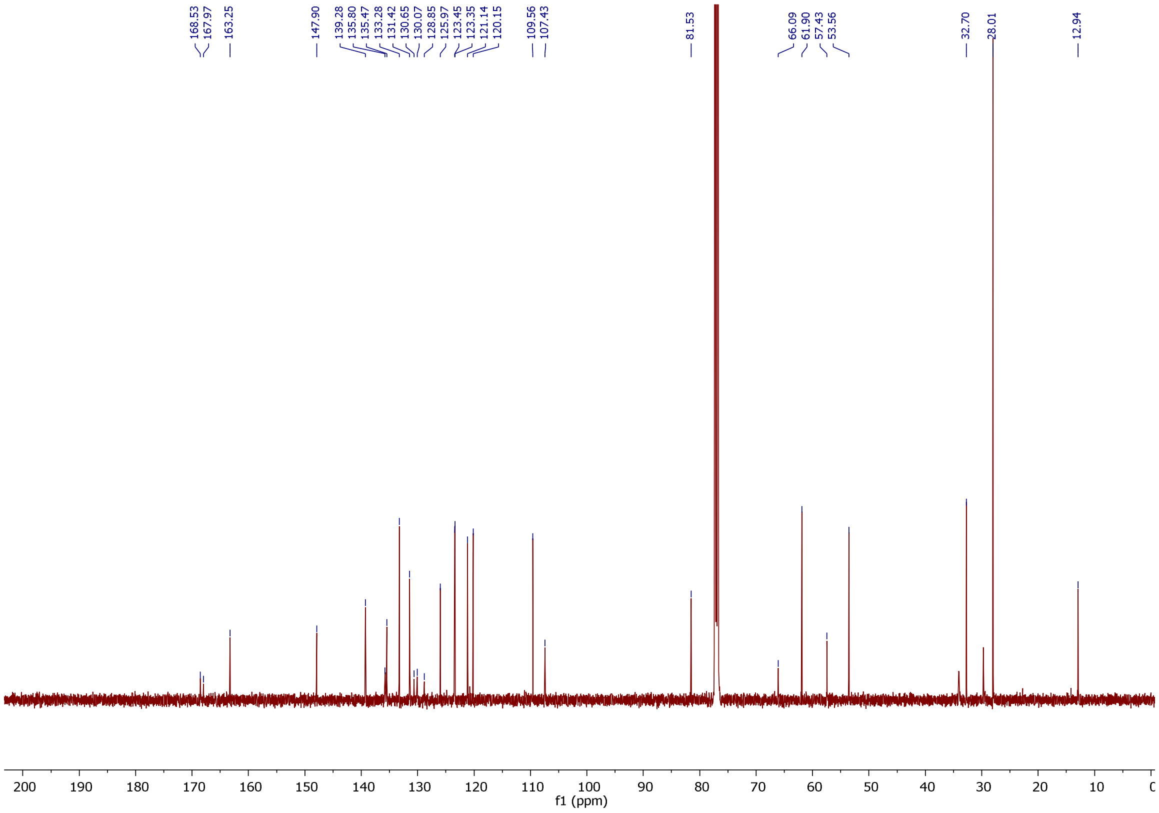
168.53 167.97 163.25 147.90 139.28 135.80 135.47 133.28 131.42 130.65 130.07 128.85 125.97 123.45 123.35 121.14 120.15 109.56 107.43
81.53
66.09 61.90 57.43 53.56
32.70 28.01 12.94
f1 (ppm)

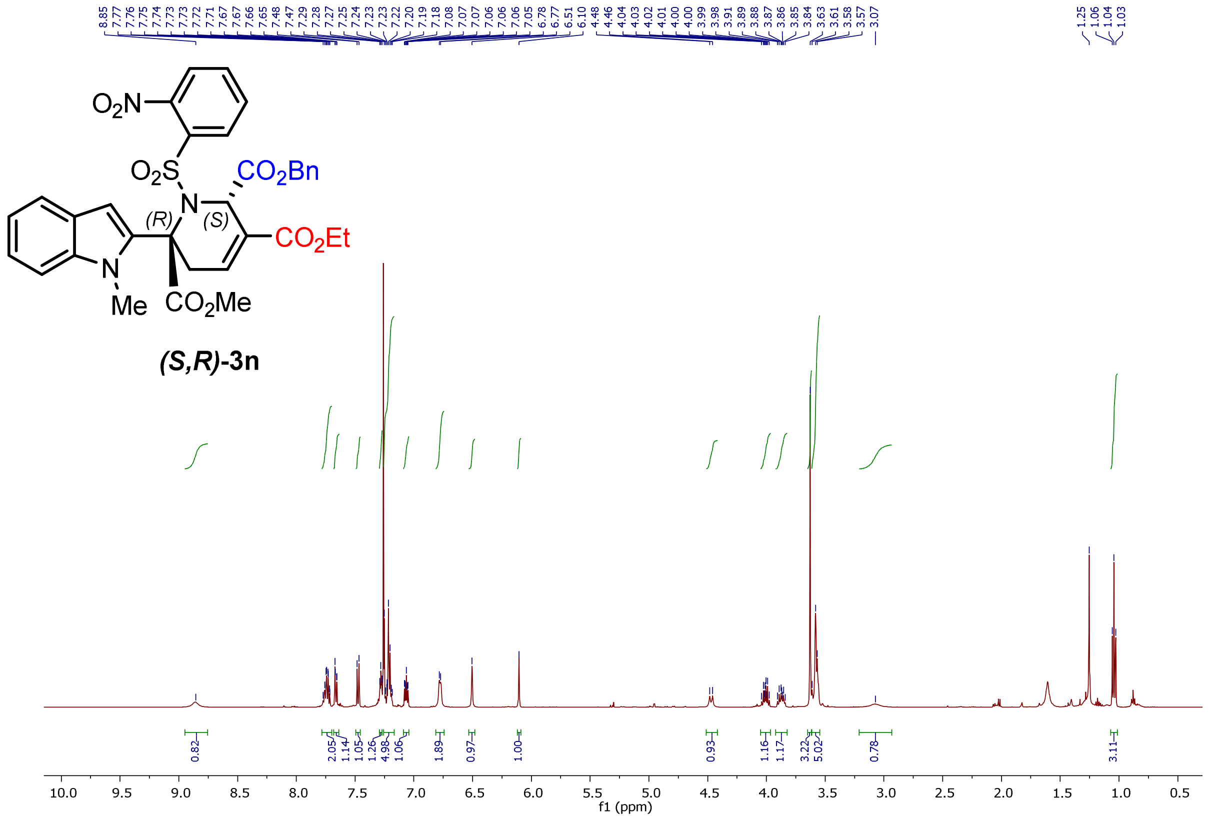

***(S,R)*-3n**

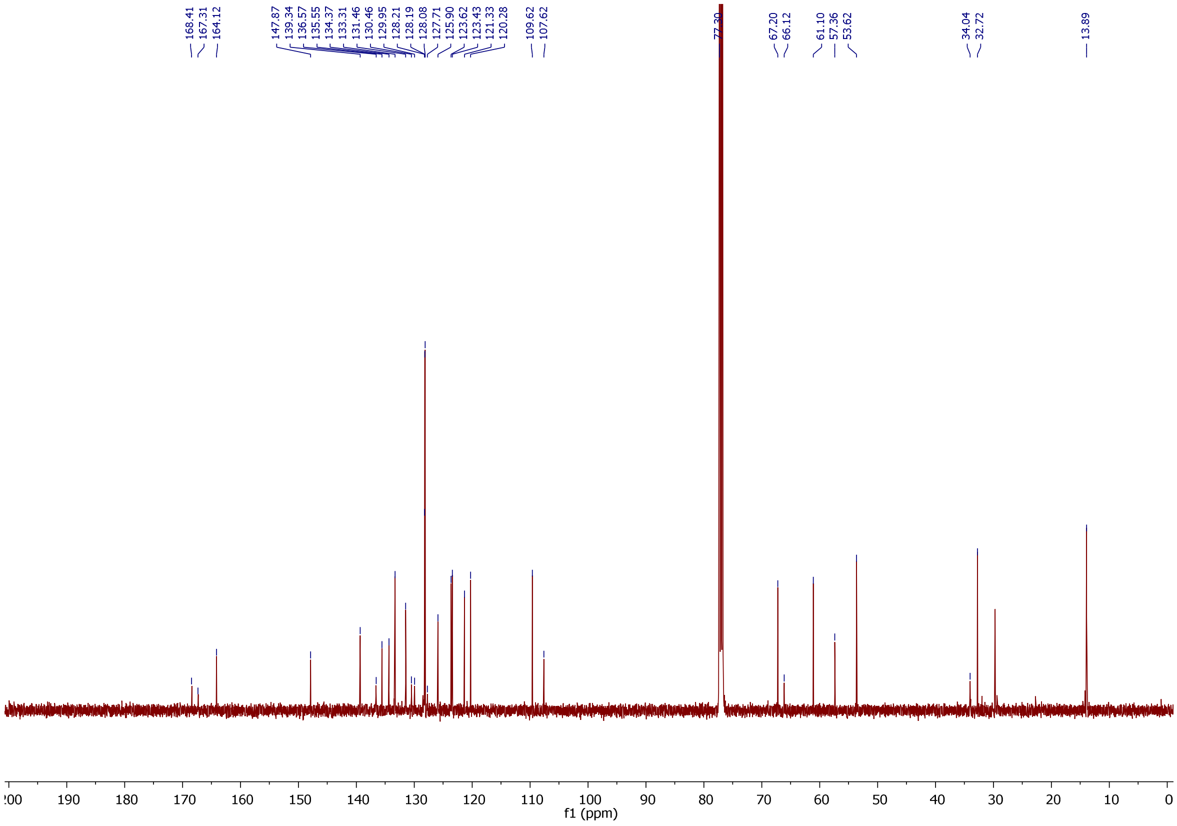

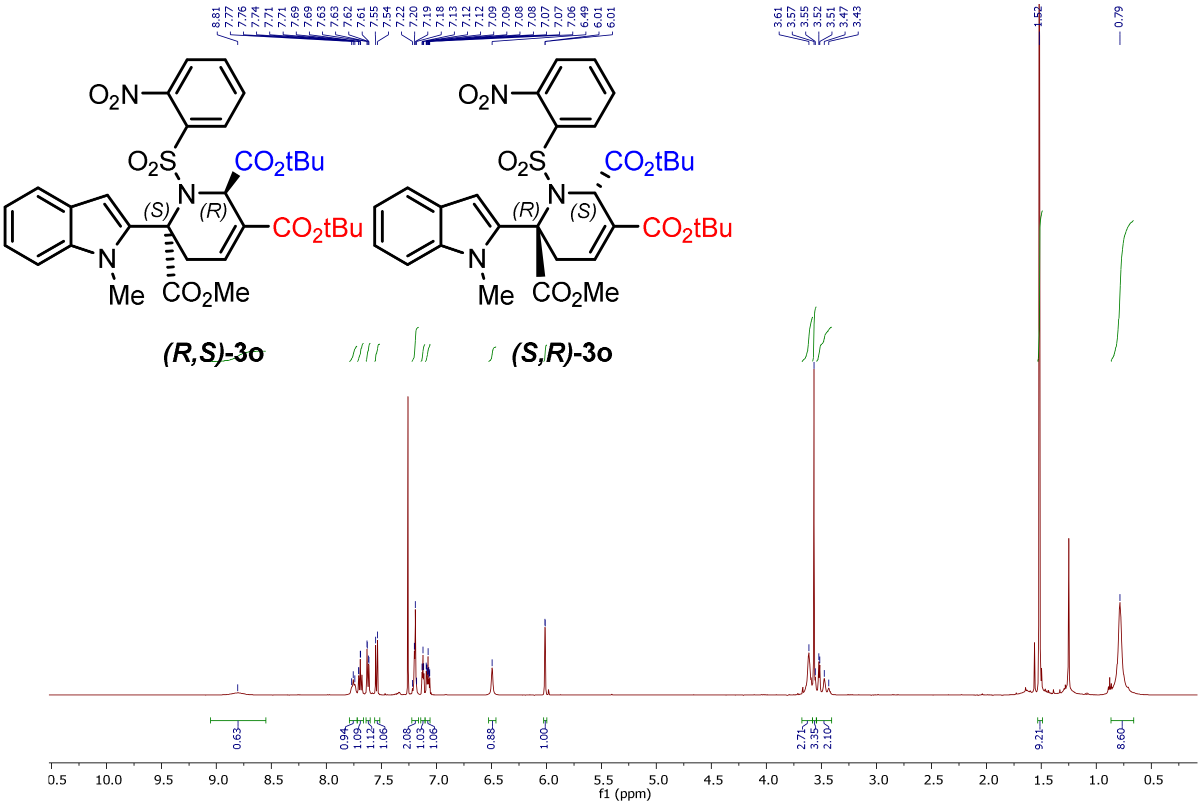
O2N
O2S
N
CO2tBu
CO2tBu
(S)
(R)
N
Me
CO2Me
(R,S)-3o
O2N
O2S
N
CO2tBu
CO2tBu
(R)
(S)
N
Me
CO2Me
(S,R)-3o
8.81 7.77 7.76 7.74 7.71 7.71 7.69 7.69 7.63 7.63 7.62 7.61 7.55 7.54 7.22 7.20 7.19 7.18 7.13 7.12 7.12 7.09 7.09 7.08 7.08 7.07 7.07 7.06 6.49 6.01 6.01
3.61 3.57 3.55 3.52 3.51 3.47 3.43
1.52
0.79
0.63
0.94 1.09 1.12 1.06
2.08 1.03 1.06
0.88
1.00
2.71 3.35 2.10
9.21
8.60
f1 (ppm)

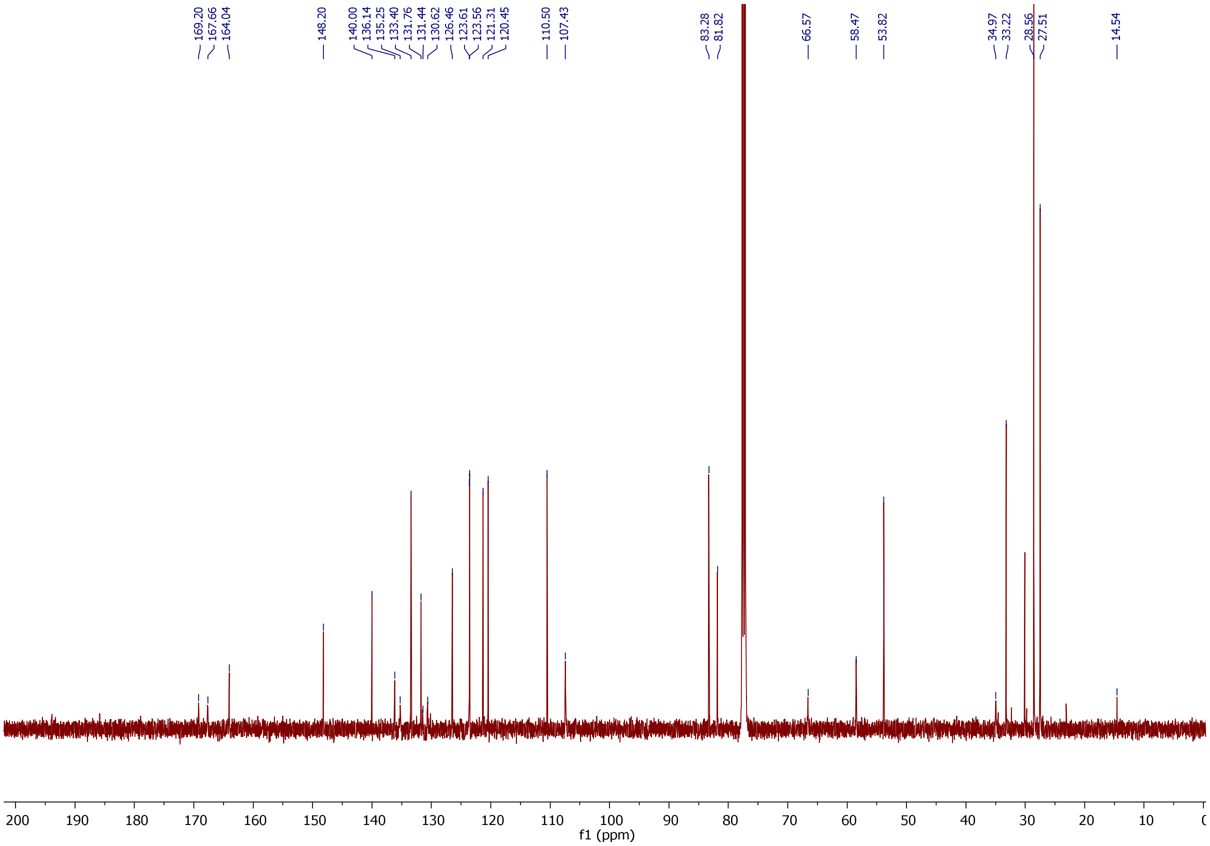
169.20 167.66 164.04
148.20
140.00 136.14 135.25 133.40 131.76 131.44 130.62 126.46 123.61 123.56 121.31 120.45
110.50 107.43
83.28 81.82
66.57
58.47
53.82
34.97 33.22 28.56 27.51
14.54
f1 (ppm)

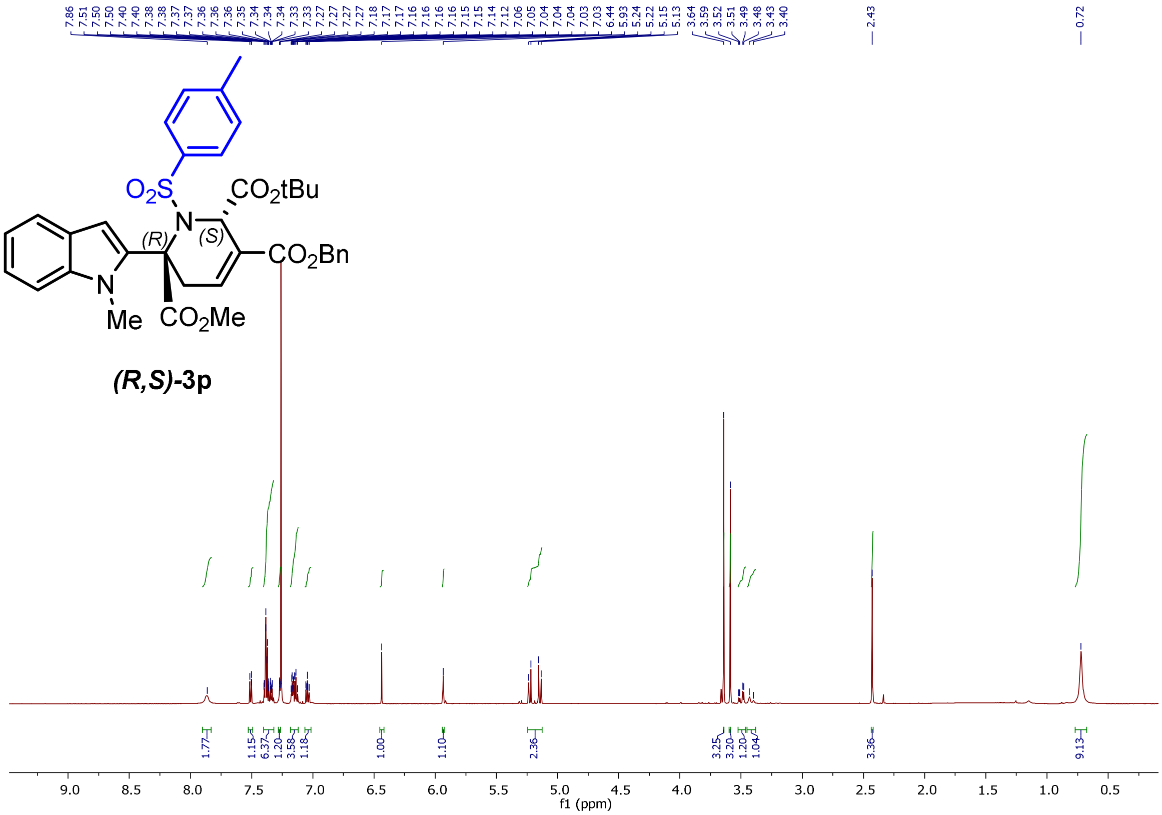

O2S
N
CO2tBu
(R)
(S)
CO2Bn
N
Me
CO2Me
(R,S)-3p
7.86 7.51 7.50 7.40 7.40 7.38 7.38 7.37 7.37 7.36 7.36 7.35 7.34 7.34 7.34 7.33 7.33 7.27 7.27 7.27 7.27 7.18 7.17 7.17 7.16 7.16 7.16 7.15 7.15 7.14 7.12 7.06 7.05 7.04 7.04 7.04 7.03 7.03 6.44 5.93 5.24 5.22 5.15 5.13
3.64 3.59 3.52 3.51 3.49 3.48 3.43 3.40
2.43
0.72
1.77 1.15 6.37 1.20 3.58 1.18 1.00 1.10 2.36 3.25 3.20 1.20 1.04 3.36 9.13
9.0 8.5 8.0 7.5 7.0 6.5 6.0 5.5 5.0 4.5 4.0 3.5 3.0 2.5 2.0 1.5 1.0 0.5
f1 (ppm)

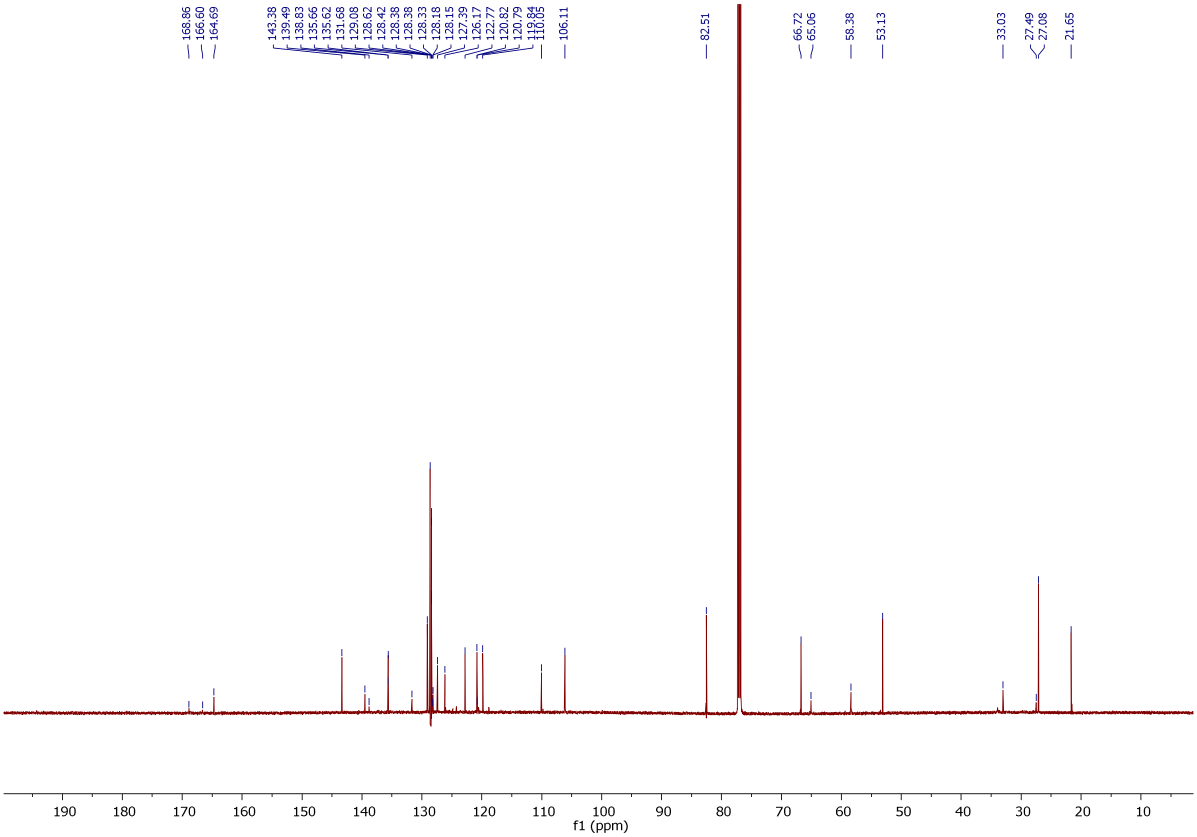

168.86 166.60 164.69
143.38 139.49 138.83 135.66 135.62 131.68 129.08 128.62 128.42 128.38 128.38 128.33 128.18 128.15 127.39 126.17 122.77 120.82 120.79 119.84 110.05 106.11
82.51
66.72 65.06 58.38 53.13
33.03 27.49 27.08 21.65
190 180 170 160 150 140 130 120 110 100 90 80 70 60 50 40 30 20 10
f1 (ppm)

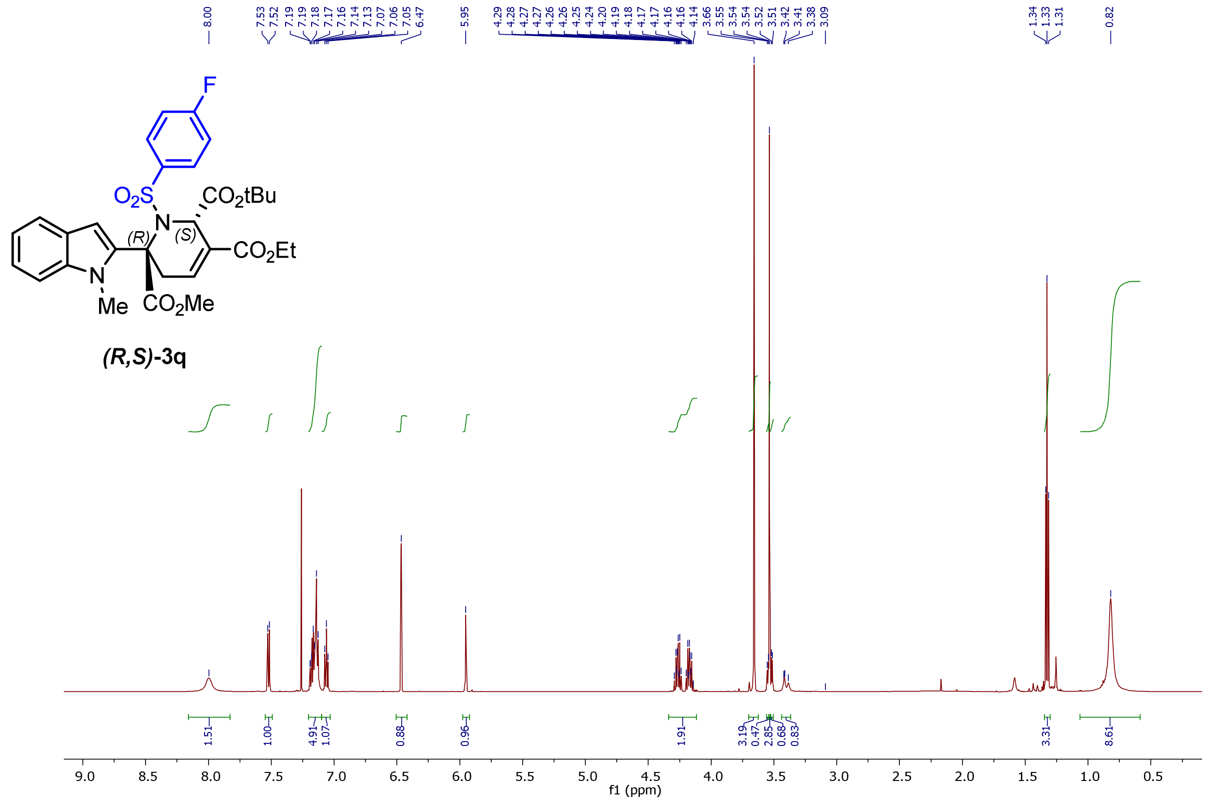
8.00
7.53
7.52
7.19
7.19
7.18
7.17
7.16
7.14
7.13
7.07
7.06
7.05
6.47
5.95
4.29
4.28
4.27
4.27
4.26
4.26
4.25
4.24
4.20
4.19
4.18
4.17
4.17
4.16
4.16
4.14
3.66
3.55
3.54
3.54
3.52
3.51
3.42
3.41
3.38
3.09
1.34
1.33
1.31
0.82
F
O_2S
CO_2tBu
N
(R)
(S)
CO_2Et
N
Me
CO_2Me
(R,S)-3q
1.51
1.00
4.91
1.07
0.88
0.96
1.91
3.19
0.47
2.85
0.68
0.83
3.31
8.61
9.0
8.5
8.0
7.5
7.0
6.5
6.0
5.5
5.0
4.5
4.0
3.5
3.0
2.5
2.0
1.5
1.0
0.5
f1 (ppm)
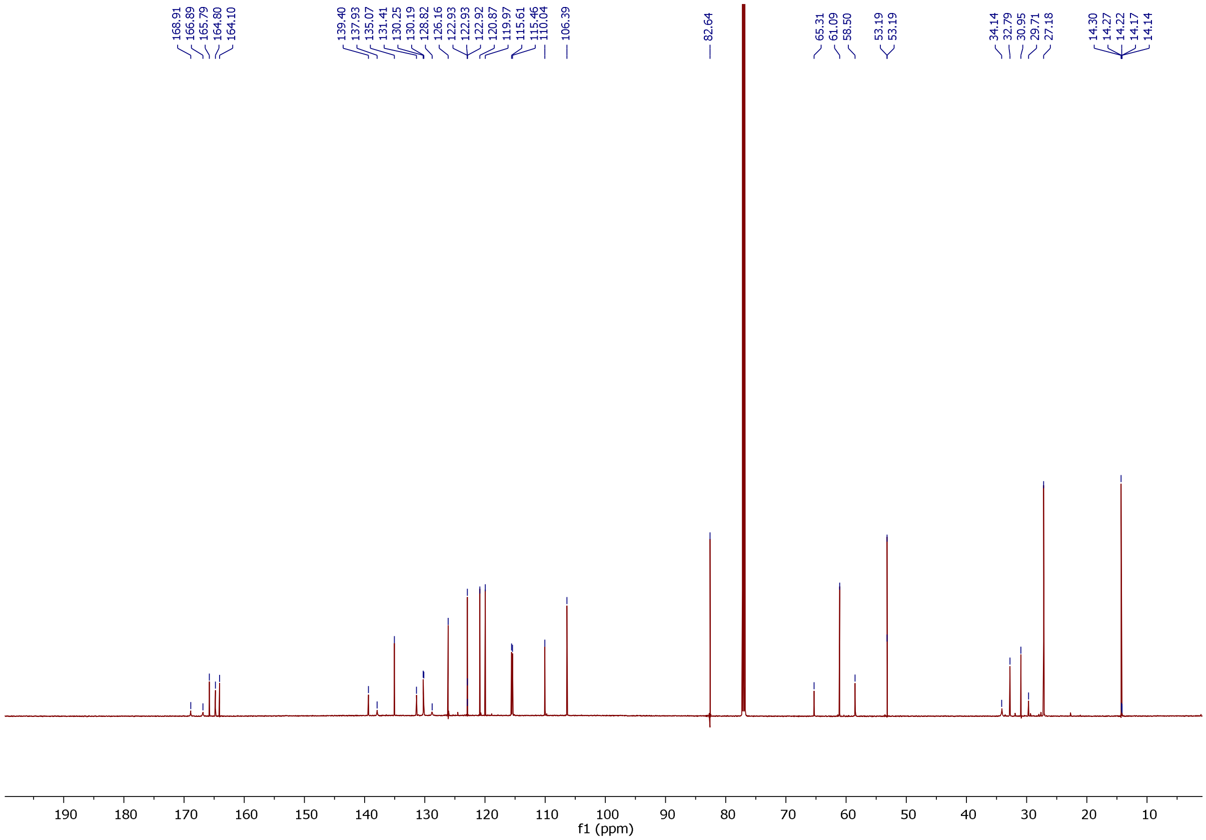
168.91
166.89
165.79
164.80
164.10
139.40
137.93
135.07
131.41
130.25
130.19
128.82
126.16
122.93
122.93
122.92
120.87
119.97
115.61
115.46
110.04
106.39
82.64
65.31
61.09
58.50
53.19
53.19
34.14
32.79
30.95
29.71
27.18
14.30
14.27
14.22
14.17
14.14
190
180
170
160
150
140
130
120
110
100
90
80
70
60
50
40
30
20
10
f1 (ppm)

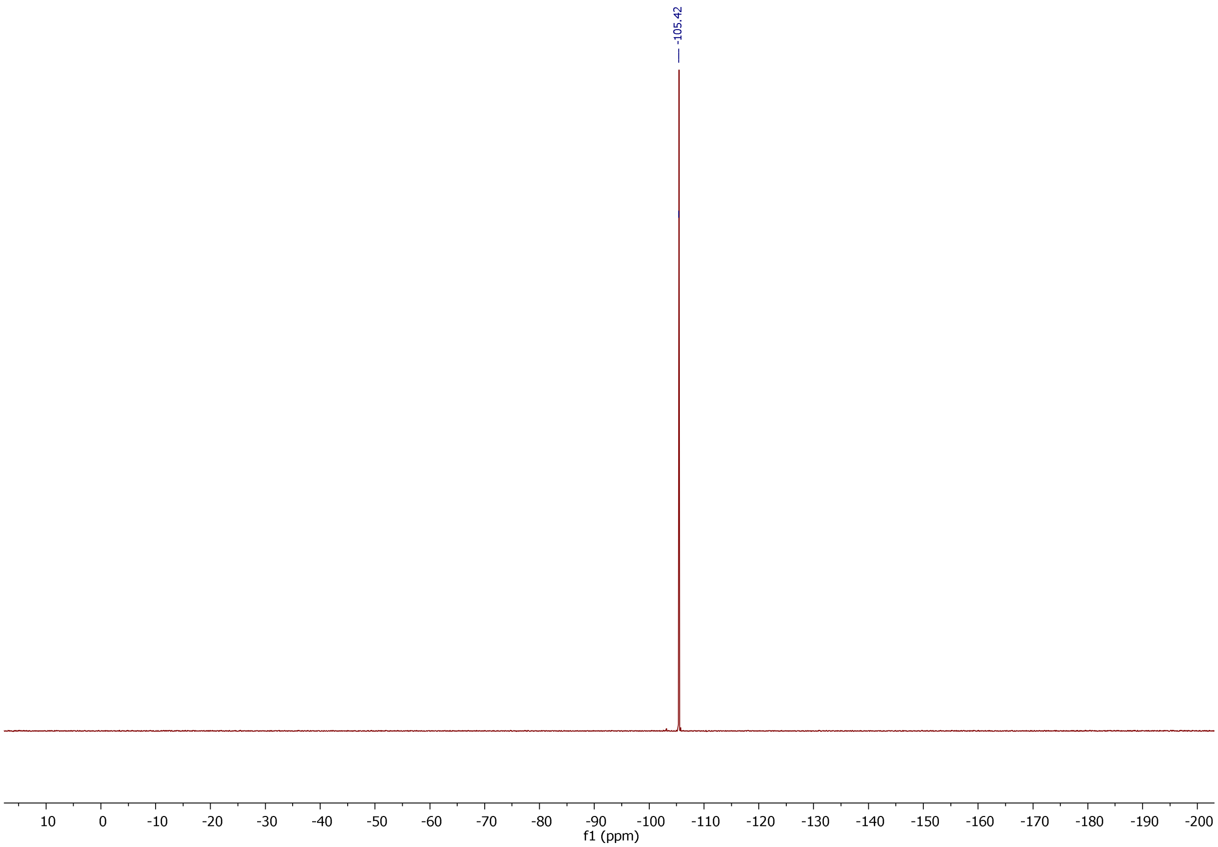
-105.42
10
0
-10
-20
-30
-40
-50
-60
-70
-80
-90
-100
-110
-120
-130
-140
-150
-160
-170
-180
-190
-200
f1 (ppm)

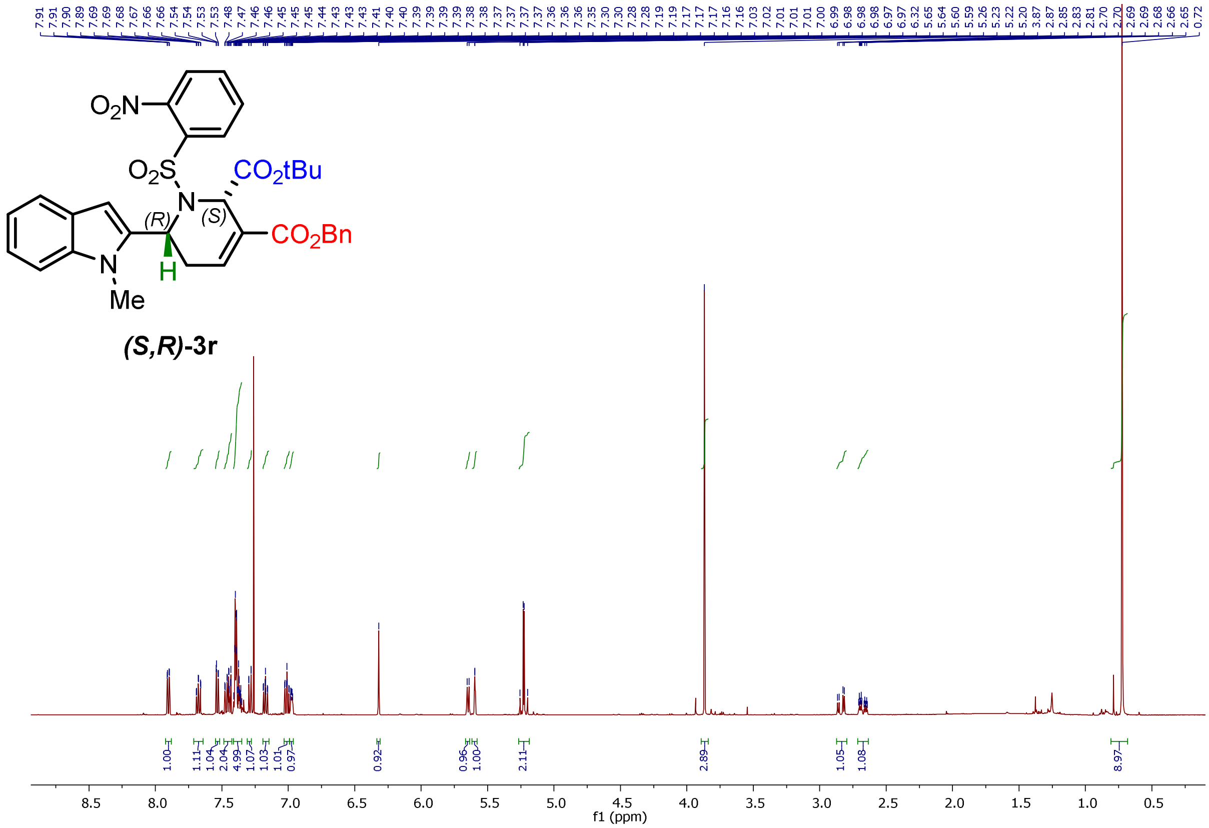

7.91 7.91 7.90 7.89 7.69 7.69 7.68 7.67 7.66 7.66 7.54 7.54 7.53 7.53 7.48 7.47 7.46 7.45 7.45 7.45 7.44 7.43 7.43 7.43 7.41 7.40 7.40 7.39 7.39 7.39 7.39 7.38 7.38 7.37 7.37 7.37 7.37 7.36 7.36 7.36 7.35 7.30 7.30 7.28 7.28 7.19 7.19 7.17 7.17 7.16 7.16 7.03 7.02 7.01 7.01 7.01 7.00 6.99 6.98 6.98 6.98 6.97 6.97 6.32 5.65 5.64 5.60 5.59 5.26 5.23 5.22 5.20 3.87 2.87 2.85 2.83 2.81 2.70 2.70 2.69 2.69 2.68 2.66 2.65 0.72
O2N
O2S
N
CO2tBu
(R)
(S)
CO2Bn
N
H
Me
(S,R)-3r
1.00 1.11 1.04 2.04 4.99 1.07 1.03 1.01 0.97 0.92 0.96 1.00 2.11 2.89 1.05 1.08 8.97
8.5 8.0 7.5 7.0 6.5 6.0 5.5 5.0 4.5 4.0 3.5 3.0 2.5 2.0 1.5 1.0 0.5
f1 (ppm)

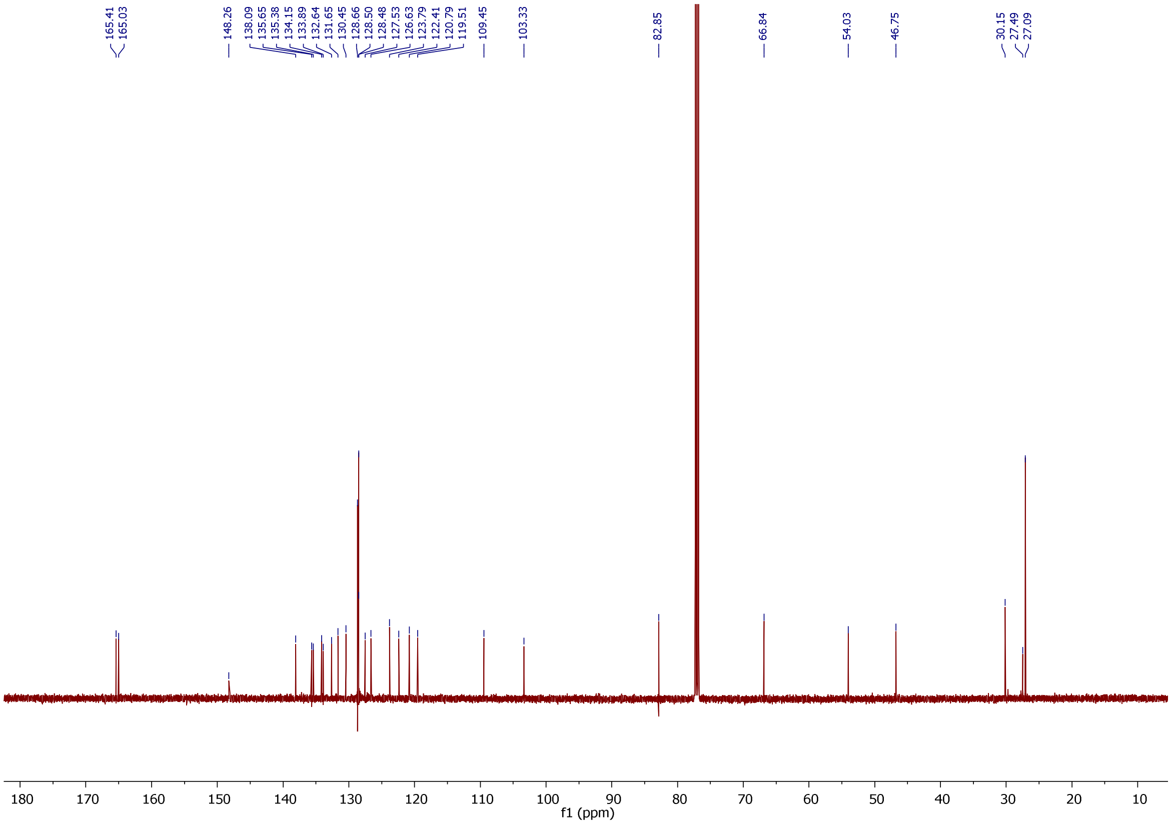

165.41 165.03 148.26 138.09 135.65 135.38 134.15 133.89 132.64 131.65 130.45 128.66 128.50 128.48 127.53 126.63 123.79 122.41 120.79 119.51 109.45 103.33 82.85 66.84 54.03 46.75 30.15 27.49 27.09
180 170 160 150 140 130 120 110 100 90 80 70 60 50 40 30 20 10
f1 (ppm)
